# Transcriptomic and Genetic Associations between Alzheimer’s Disease, Parkinson’s Disease, and Cancer

**DOI:** 10.3390/cancers13122990

**Published:** 2021-06-15

**Authors:** Jaume Forés-Martos, Cesar Boullosa, David Rodrigo-Domínguez, Jon Sánchez-Valle, Beatriz Suay-García, Joan Climent, Antonio Falcó, Alfonso Valencia, Joan Anton Puig-Butillé, Susana Puig, Rafael Tabarés-Seisdedos

**Affiliations:** 1 Biomedical Research Networking Center of Mental Health (CIBERSAM), 28029 Madrid, Spain; jaume.foresmartos@uchceu.es; 2 ESI International Chair@CEU-UCH, Universidad Cardenal Herrera-CEU, CEU Universities, San Bartolomé 55, 46115 Alfara del Patriarca, Spain; beatriz.suay@uchceu.es (B.S.-G.); joan.climentbataller@uchceu.es (J.C.); afalco@uchceu.es (A.F.); 3 Departamento de Matemáticas, Física y Ciencias Tecnológicas, Universidad Cardenal Herrera-CEU, CEU Universities, San Bartolomé 55, 46115 Alfara del Patriarca, Spain; 4 IES Miguel Catalán, 28823 Coslada, Spain; cesarboullosa@gmail.com; 5 Consorcio Hospital General de Valencia, Servicio de Medicina Interna, 46014 Valencia, Spain; darodo@alumni.uv.es; 6 Barcelona Supercomputing Center (BSC), 08034 Barcelona, Spain; jon.sanchez@bsc.es (J.S.-V.); alfonso.valencia@bsc.es (A.V.); 7 Departamento de Producción y Sanidad Animal, Salud Pública Veterinaria y Ciencia y Tecnología de los Alimentos, Universidad Cardenal Herrera-CEU, CEU Universities, C/Tirant lo Blanc 7, 46115 Alfara del Patriarca, Spain; 8 Catalan Institution for Research and Advanced Studies (ICREA), 08010 Barcelona, Spain; 9 Biochemical and Molecular Genetics Service, Hospital Clínic and August Pi i Sunyer Biomedical Research Institute (IDIBAPS), 08036 Barcelona, Spain; japuig@clinic.ub.es; 10 Melanoma Unit, Hospital Clínic, Center for Networked Biomedical Research on Rare Diseases (CIBERER), Carlos III Health Institute (ISCIII), 08036 Barcelona, Spain; spuig@clinic.cat; 11 Dermatology Department, Hospital Clínic and August Pi i Sunyer Biomedical Research Institute (IDIBAPS), 08036 Barcelona, Spain; 12 Teaching Unit of Psychiatry and Psychological Medicine, Department of Medicine, University of Valencia, Blasco-Ibañez 15, 46010 Valencia, Spain; 13 INCLIVA Health Research Institute, 46010 Valencia, Spain

**Keywords:** Alzheimer, Parkinson, comorbidity, transcriptomic, meta-analyses, genetic correlations

## Abstract

**Simple Summary:**

Epidemiological studies have identified a link between neurodegenerative disorders and a reduced risk of overall cancer. Increases and decreases in the risk of site-specific cancers have also been reported. However, it is still unknown whether these associations arise due to shared genetic and molecular factors or are explained by other phenomena (e.g., biases in epidemiological studies or the use of medication). In this study, we aimed to investigate the potential molecular, genetic, and pharmacological links between Alzheimer’s and Parkinson’s diseases and a large panel of 22 cancer types. To examine the overlapping involvement of genes and pathways, we obtained differential gene expression profiles through meta-analyses of post-mortem brain tissues from Alzheimer’s and Parkinson’s disease patients, primary tumors, and tissue-matched controls, and compared them. Genetic similarities were assessed through network-based methods and the computation of genetic correlations. Finally, the potential impact of drugs indicated for each disorder in the identified associations was evaluated using transcriptomic methods. Our research extends previous work in the field by identifying new significant patterns of transcriptomic associations (direct and inverse) between Alzheimer’s disease, Parkinson’s disease, and different site-specific cancers. The results reveal significant genetic correlations between Parkinson’s disease, prostate cancer, and melanoma. In addition, to our knowledge, this is the first time that the role of drugs indicated for the treatment of both sets of disorders has been investigated in the context of their comorbid associations using transcriptomic methods.

**Abstract:**

Alzheimer’s (AD) and Parkinson’s diseases (PD) are the two most prevalent neurodegenerative disorders in human populations. Epidemiological studies have shown that patients suffering from either condition present a reduced overall risk of cancer than controls (i.e., inverse comorbidity), suggesting that neurodegeneration provides a protective effect against cancer. Reduced risks of several site-specific tumors, including colorectal, lung, and prostate cancers, have also been observed in AD and PD. By contrast, an increased risk of melanoma has been described in PD patients (i.e., direct comorbidity). Therefore, a fundamental question to address is whether these associations are due to shared genetic and molecular factors or are explained by other phenomena, such as flaws in epidemiological studies, exposure to shared risk factors, or the effect of medications. To this end, we first evaluated the transcriptomes of AD and PD post-mortem brain tissues derived from the hippocampus and the substantia nigra and analyzed their similarities to those of a large panel of 22 site-specific cancers, which were obtained through differential gene expression meta-analyses of array-based studies available in public repositories. Genes and pathways that were deregulated in both disorders in each analyzed pair were examined. Second, we assessed potential genetic links between AD, PD, and the selected cancers by establishing interactome-based overlaps of genes previously linked to each disorder. Then, their genetic correlations were computed using cross-trait LD score regression and GWAS summary statistics data. Finally, the potential role of medications in the reported comorbidities was assessed by comparing disease-specific differential gene expression profiles to an extensive collection of differential gene expression signatures generated by exposing cell lines to drugs indicated for AD, PD, and cancer treatment (LINCS L1000). We identified significant inverse associations of transcriptomic deregulation between AD hippocampal tissues and breast, lung, liver, and prostate cancers, and between PD substantia nigra tissues and breast, lung, and prostate cancers. Moreover, significant direct (same direction) associations of deregulation were observed between AD and PD and brain and thyroid cancers, as well as between PD and kidney cancer. Several biological processes, including the immune system, oxidative phosphorylation, PI3K/AKT/mTOR signaling, and the cell cycle, were found to be deregulated in both cancer and neurodegenerative disorders. Significant genetic correlations were found between PD and melanoma and prostate cancers. Several drugs indicated for the treatment of neurodegenerative disorders and cancer, such as galantamine, selegiline, exemestane, and estradiol, were identified as potential modulators of the comorbidities observed between neurodegeneration and cancer.

## 1. Introduction

The study of comorbidity (or multimorbidity) is becoming a key topic in biomedical research, and it is especially relevant in the context of population aging [1]. Comorbidities have profound implications for individuals, practitioners, and healthcare systems [2]. As a consequence, the scientific community is devoting increasing efforts to the characterization of relationships between disorders and to the identification of factors that cause these associations [1]. 

Central nervous system (CNS) disorders and cancer are among the leading causes of death and disability worldwide [3,4,5]. Epidemiological data suggest that the cancer incidence and mortality patterns of patients with CNS disorders differ from those of the general population [2]. In particular, several observational studies and meta-analyses have found that individuals diagnosed with neurodegenerative (NDG) disorders, such as Alzheimer’s disease (AD) and Parkinson’s disease (PD), are at a lower risk of subsequent all-cancer incidence and mortality compared to controls [2,6,7,8,9,10,11,12,13,14,15,16,17,18,19,20,21,22,23,24,25,26,27,28,29]. We observed that this apparent anti-cancer protective effect, which we term ‘inverse cancer comorbidity’ (ICC), is produced in many serious CNS and immune disorders [30,31,32]. In addition, a number of associations between AD and PD and site-specific cancers have been reported, including a reduction in the risk of lung cancer [2,10,18,19,20,21,22,23,24,33,34], as well as a decrease in the risks of bladder, colorectal, and prostate cancers [2,11,18,19,20,21,22,23,32,34,35,36,37] and smoking-related cancers in general [38] in individuals with PD. Reduced risks of pancreatic, stomach, liver, blood, and endometrial cancers have also been observed [36,39,40]. By contrast, some studies have suggested that PD patients are at an increased risk of brain cancer and melanoma [17,21,22,23,41,42,43], and others have found an increased risk of these cancer types in the first-degree relatives of PD patients [36,44]. Increases in breast cancer [36,39,40,45,46] have also been reported. Several hypotheses have been proposed to account for these associations, including the presence of systematic biases in observational studies [10,47,48], a lack of adjustment for important confounding variables such as smoking status [49], differential exposure to risk factors [50], the presence of shared alterations in genes and pathways [51,52], the coinciding effects of variants [53], and the potential role of drugs indicated for these disorders [54,55].

The opposite patterns of cell behavior observed in cancer and neurodegeneration, with cancer arising as a consequence of uncontrolled cell proliferation and neurodegeneration resulting from post-mitotic cell death, have prompted researchers to examine the potential molecular bases of their inverse comorbid associations. Early studies revealed that genes upregulated in the substantia nigra in PD patients are biologically linked to cancer, diabetes, and inflammation [56]. More recent works have shown that transcriptomic deregulation patterns in brain tissues derived from AD and PD patients are the opposite of those in lung, prostate, and colorectal cancers. Conversely, patterns of transcriptomic deregulation in AD have been found to be similar to those observed in glioblastomas [51,52]. In addition, at least one study has investigated the involvement of shared genetic variability between AD and a set of site-specific cancers by computing genetic correlations based on GWAS summary statistics and cross-trait LD score regression. The results revealed significant positive genetic correlations between AD and both breast and lung cancers [53]. However, it is still unknown whether these results can be generalized to other cancer types and whether alterations produced by drugs indicated for the treatment of these disorders could be involved in modulating the identified comorbid associations.

To our knowledge, this is the first study to investigate the molecular associations between AD and PD and a panel of 22 site-specific cancers using different approaches. First, we generated differential gene expression profiles for each included disorder through meta-analyses of previously published array data. Then, we compared the AD and PD profiles to those derived from the 22 cancer types and assessed whether they shared patterns of transcriptomic deregulation. Second, Gene Set Enrichment Analyses (GSEAs) were carried out to identify alterations in biological processes and pathways that were common to both neurodegenerative disorders and cancers. A Shiny application was created in order to easily visualize the biological processes and pathways altered in each specific disorder and those jointly deregulated in NDG and cancer. The results were validated using an independent cohort of samples profiled using RNA-seq methods. Gene co-expression network analyses were also performed through the construction of consensus co-expression modules for each disease. Up- and downregulated modules were identified for each disorder and compared between diseases. Third, it has been shown that disease-associated genes, variant genes, and proteins are not randomly distributed in the human interactome [57]. Instead, they tend to interact with each other more than expected by chance, forming connected subgraphs that are known as disease modules. According to this view, a disease is produced by a local perturbation in the underlying disease module, and a comorbidity arises as a consequence of perturbations in overlapping disease modules. In other words, if two disease modules overlap, local perturbations leading to one disease could disrupt pathways involved in the module of the other disease. Therefore, we computed interactome-based overlaps between the disease modules of AD and PD and those derived from the 22 included cancers. To test whether genetic variation is shared between the development of AD, PD, and cancer, we obtained GWAS summary statistics and computed genetic correlations using cross-trait LD score regression [58]. Finally, the potential role of medications indicated for the treatment of AD, PD, and cancer in their reported comorbid associations was assessed using transcriptomic methods and data derived from LINCS L1000.

## 2. Materials and Methods 

### 2.1. Differential Gene Expression Meta-Analyses, Gene Set Enrichment Analyses, Weighted Co-Expression Network Analyses, and Measures of Transcriptomic Association between NDG Disorders and Cancer

Searches for transcriptomic datasets, including tissue-matched case and control samples, were carried out in public repositories (i.e., the Gene Expression Omnibus (GEO), Array Express (AE), and The Cancer Genome Atlas (TCGA)) for AD, PD, and 22 tumor types: acute lymphoblastic leukemia (ALL), acute myeloid leukemia (AML), bladder cancer (BLCA), breast cancer (BRCA), brain cancer (BRNCA), cervical cancer (CERV), cholangiocarcinoma (CHLCA), chronic lymphocytic leukemia (CLL), chronic myeloid leukemia (CML), colorectal cancer (CRCA), diffuse large B-cell lymphoma (DLBCL), follicular lymphoma (FLYMPH), head and neck carcinoma (HANC), kidney cancer (KDNCA), lung cancer (LGCA), liver cancer (LIVCA), ovarian cancer (OVCA), pancreatic cancer (PACA), prostate cancer (PRCA), skin cancer melanoma (SKCM), stomach cancer (STCA), and thyroid cancer (THCA) (see Supplementary Methods 1.1 at Supplementary File S1). The data were preprocessed using standard methods, and sample- and study-level quality control and outlier detection and removal methods were applied (Supplementary Methods 1.2, 1.3, and 1.4). For each disorder, differential gene expression meta-analyses were carried out using all of the available studies and the MetaDE package [59] (Supplementary Methods 1.5). Transcriptomic associations between AD and PD and each included cancer were determined through intersection analyses and the computation of correlations (Supplementary Methods 1.6). The observed associations were validated using additional cohorts of cancers derived from TCGA (Supplementary Methods 1.7). Consensus modules of co-expressed genes were obtained for each disorder and correlated with disease status using the WGCNA package [60], and disease-associated modules were compared between disorders using hypergeometric tests (Supplementary Methods 1.8 and 1.9). Altered biological processes and pathways were identified by applying different types of enrichment methods (Supplementary Methods 1.10).

### 2.2. Human Interactome-Based Overlaps and Cross-Trait LD Score Regression Analyses

Three different human interactomes (Supplementary Methods 1.11) and lists of disease-associated genes and variant genes (Supplementary Methods 1.12) were employed in order to compute measures of network localization and network separation between NDG disorders and cancers (Supplementary Methods 1.13 and 1.14). The presence of shared genetic variability between NDG disorders and cancer was tested by employing GWAS summary statistics data and cross-trait LD score regression [58]. GWAS summary statistics were obtained from public repositories or directly requested from the authors (Supplementary Methods 1.15 and 1.16).

### 2.3. Identification of Drugs Indicated for the Treatment of AD, PD, and Cancer as Potential Modulators of their Comorbidities through LINCS L1000 Analysis

We explored the potential role of indicated medications in the comorbidities observed between NDG disorders and cancer. First, drugs indicated for the treatment of AD, PD, and the 22 studied cancers were identified using MEDI-an, a medication indication repository that gathers information from multiple resources. Then, transcriptomic effects induced by treatment with these drugs were tested using LINCS L1000 data by constructing differential gene expression consensus signatures and computing correlations between profiles induced by drug treatment and those derived from the studied disorders (Supplementary Methods 1.17). 

## 3. Results

### 3.1. Results of Differential Gene Expression Meta-Analyses 

Data from 176 array-based studies were identified in GEO and AE. After quality control and sample- and study-level outlier detection and removal, 144 studies remained, resulting in a total of 15,136 samples divided into 11,359 cases and 3777 controls. Of the 32 excluded studies with SRM values larger than 7, there was one on AML, three on BRNCA, eight on CRCA, two on KDNCA, nine on LGCA, four on LIVCA, and five on PD. Seven studies, which included 226 samples derived from hippocampal samples of 124 AD cases and 102 tissue-matched controls, were selected for analyses. In the case of PD, data from six studies encompassing 149 samples derived from the substantia nigra of 83 cases and 66 controls were selected for further analysis. The number of samples of different site-specific cancers ranged between 139 (CERV) and 1500 (ALL). Supplementary Table S1 shows the number of studies and samples identified for each of the included cancers. The differential gene expression meta-analysis yielded totals of 3341 and 3473 differentially expressed genes (DEGs) with FDR-adjusted *p*-values lower than 0.05 for AD and PD, respectively. The number of DEGs resulting from cancer analyses ranged from 581 in AML to 9757 in BRNCA. Table 1 shows the number of DEGs obtained in each analysis under two significance thresholds (0.05 and 0.01), as well as the number of studies and samples included in each meta-analysis. Supplementary File S2 provides the complete differential gene expression meta-analysis results for all of the included disorders. 

### 3.2. Transcriptomic Associations between Neurodegenerative Disorders and Cancers

Transcriptomic associations between AD and PD and all of the studied cancers were computed by determining whether the number of genes placed at the four possible intersections of significantly upregulated and downregulated genes in each member of a given disease pair was larger than expected by chance (see SM 1.6). The significance of these overlaps was measured using Fisher’s exact tests. 

**Figure 1:**
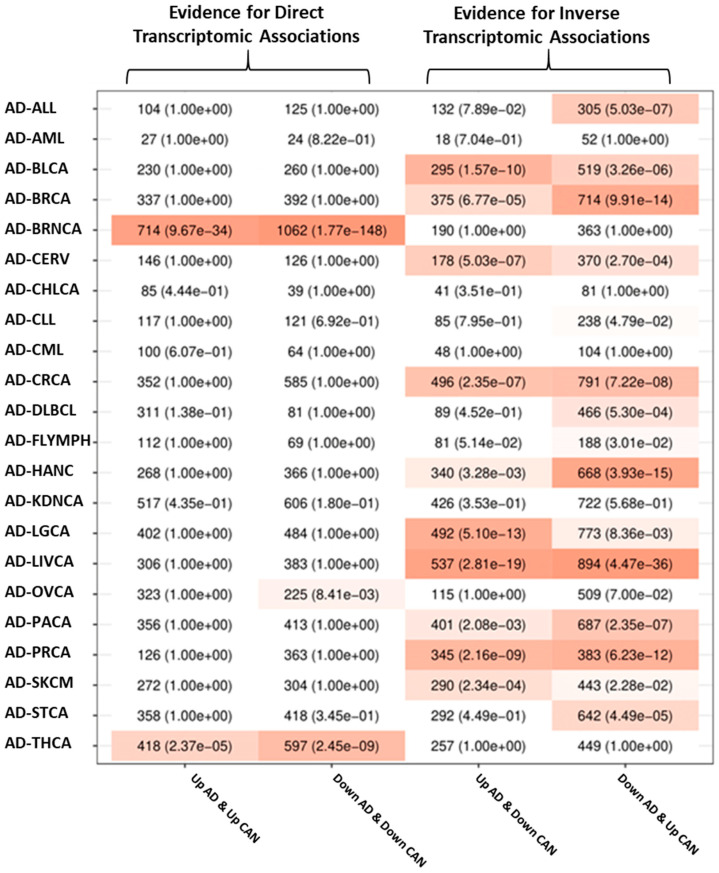
Results of AD and cancer intersection analysis. Each column shows the number of genes at the four possible intersections of up- and downregulated genes in each AD and cancer pair, as well as the FDR-adjusted *p*-values of Fisher’s exact tests. Column 1 shows genes upregulated in both conditions, column 2 shows genes downregulated in both conditions, column 3 shows genes upregulated in AD and downregulated in each cancer, and column 4 shows genes downregulated in AD and upregulated in each cancer.

**Figure 2:**
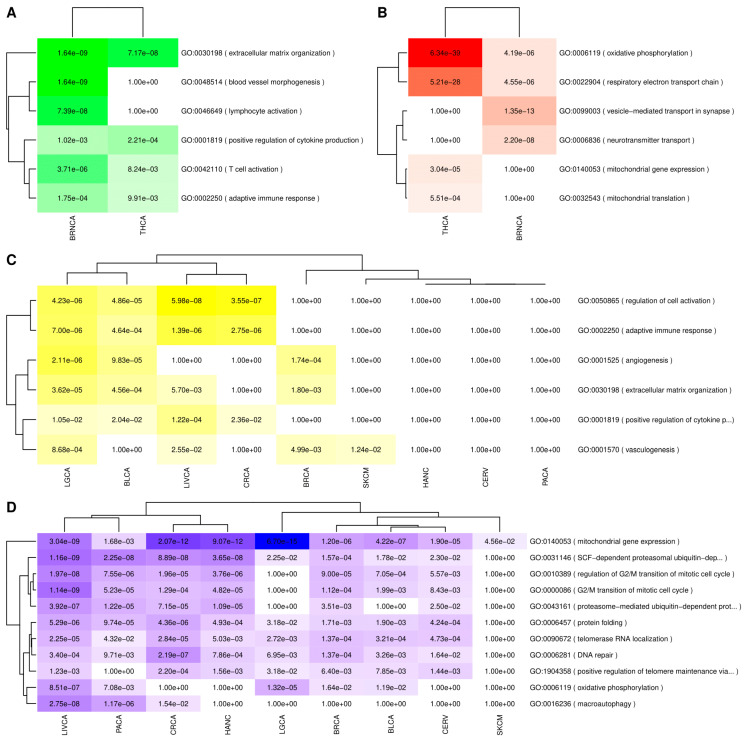
GO overrepresentation analysis results of genes significantly deregulated in AD and cancers. (**A**) Genes upregulated in AD and its SDDCs. (**B**) Genes downregulated in AD and its SDDCs. (**C**) Genes jointly upregulated in AD and downregulated in its ODDCs. (**D**) Genes jointly downregulated in AD and upregulated in its ODDCs. Color hues are proportional to the FDR adjusted p-vales of the overrepresentation results, with darker hues corresponding to lower *p*-values.

**Figure 3:**
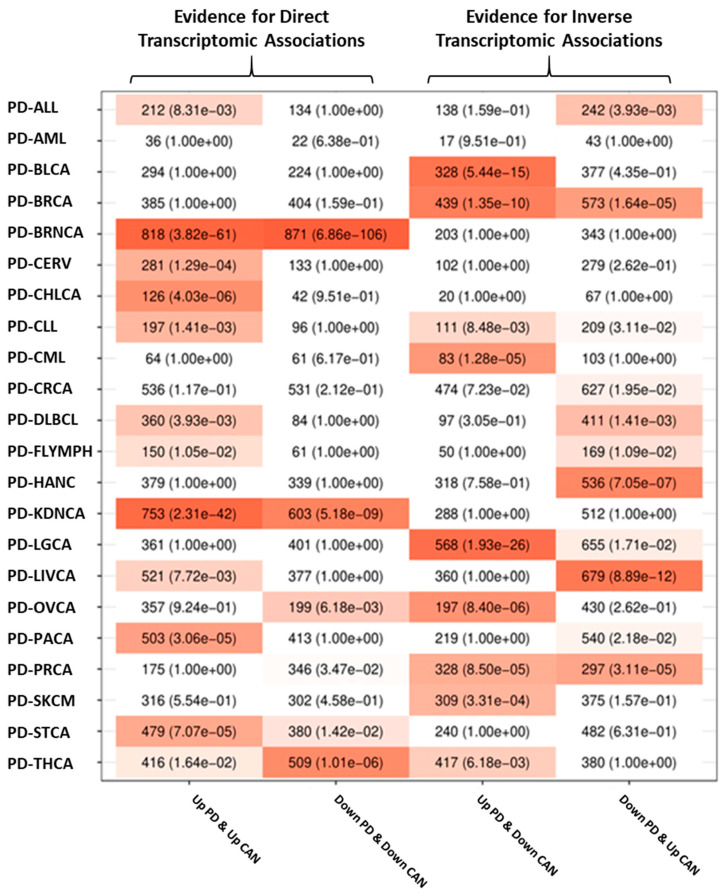
PD and cancer intersection analysis results. Each column shows the number of genes at the four possible intersections formed by up- and downregulated genes in each PD and cancer pair, as well as the FDR-adjusted *p*-values of Fisher’s exact tests. Column 1 shows genes upregulated in both conditions, column 2 shows genes downregulated in both conditions, column 3 shows genes upregulated in PD and downregulated in each cancer, and column 4 shows genes downregulated in PD and upregulated in each cancer.

**Figure 4:**
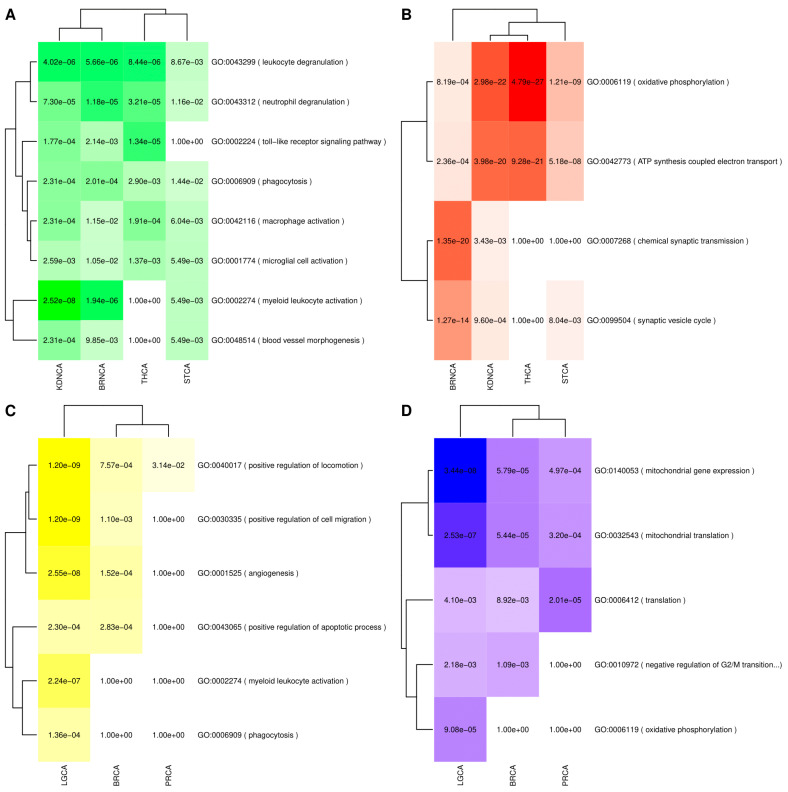
GO overrepresentation analysis results of genes significantly deregulated in PD and cancers. (**A**) Genes upregulated in PD and its SDDCs. (**B**) Genes downregulated in PD and its SDDCs. (**C**) Genes upregulated in PD and downregulated in its ODDCs. (**D**) Genes downregulated in PD and upregulated in its ODDCs. Color hues are proportional to the FDR adjusted p-vales of the overrepresentation results, with darker hues corresponding to lower *p*-values.

Overrepresentation analysis of deregulated genes shared between each disease pair was carried out using Gene Ontology (GO) as the source of gene sets linked to specific biological processes. The significant overrepresented pathways mentioned in the text are presented alongside their GO identifiers and the adjusted *p*-values (p-adj). Figure 1 and Figure 2 show the intersection analysis and overrepresentation analyses results for AD and all the included cancer types, whereas Figure 3 and Figure 4 show the same analyses results for PD. Supplementary Figures S1 and S2 show the correlations computed using the $\hat{\mu }$ values derived from the differential expression profiles of both NDG disorders and all of the included cancers. The complete overrepresentation enrichment analyses results of the genes placed at the significant intersections can be found in Supplementary File S3.

#### 3.2.1. Transcriptomic Associations between AD and Cancer

AD was directly associated with BRNCA and THCA (henceforth, AD’s same-direction deregulated cancers (SDDCs)): the intersections formed by genes that were upregulated and downregulated in both diseases were significant after adjustment by multiple comparisons. Several cancer types were inversely associated with AD, including LGCA, CERV, HANC, CRCA, PACA, BRCA, LIVCA, BLCA, and SKCM. These cancers are termed AD’s opposite-direction deregulated cancers (ODDCs). In these cases, intersections formed by genes upregulated in one disorder and downregulated in the other were both significant. The differential gene expression profiles of BRNCA and THCA were positively correlated with the AD profile (BRNCA r = 0.32, THCA r = 0.11). Negatively associated cancers had Pearson’s correlations of −0.22, −0.17, −0.12, −0.11, −0.1, −0.09, −0.09, −0.08, and −0.05 for LGCA, CERV, HANC, CRCA, PACA, BRCA, LIVCA, BLCA, and SCKM, respectively.

Genes upregulated in both AD and its SDDCs were enriched in functions linked to extracellular matrix organization, and immune system-related processes, such as adaptive immune response, T-cell activation, and positive regulation of cytokine production. Genes upregulated in both AD and BRNCA were also enriched in lymphocyte activation and blood vessel morphogenesis (Figure 2A). 

Genes that were downregulated in both AD and its SDDCs were enriched in biological processes linked to energy production such as, oxidative phosphorylation, and respiratory electron transport chain. Genes jointly downregulated in AD and BRNCA were also enriched in neural-related processes such as, vesicle-mediated transport in synapse and neurotransmitter transport (Figure 2B). 

Genes upregulated in AD and downregulated in its ODDCs were enriched in immune-related processes, including, the regulation of cell activation, adaptive immune response, and the positive regulation of cytokine production, and other processes such as angiogenesis, vasculogenesis, and extracellular matrix organization (Figure 2C). 

Finally, genes downregulated in AD and upregulated in its ODDCs were enriched in biological processes linked to the G2/M transition of the mitotic cell cycle and its regulation. Other processes downregulated in AD and upregulated in its ODDCs were mitochondrial gene expression, oxidative phosphorylation, and proteasomal-related processes, such as SCF-dependent ubiquitin-dependent protein catabolic process, as well as pathways linked to DNA repair, protein folding, and telomere maintenance (Figure 2D). 

#### 3.2.2. Transcriptomic Associations between PD and Cancer

PD was found to present direct transcriptomic associations with BRNCA, KDNCA, THCA, and STCA and inverse transcriptomic associations with LGCA, BRCA, and PRCA, as well as chronic lymphocytic leukemia (CLL) to a minor extent. The differential gene expression profiles of BRNCA, KDNCA, and STCA were positively correlated with the PD profile (BRNCA r = 0.33, KDNCA r = 0.17, STCA r = 0.05), whereas the correlation between Parkinson’s disease and THCA gene expression signatures was negligible (THCA r = 0.03). Among the negatively associated cancers, only LGCA presented a negative correlation with an absolute value higher than 0.1 (LGCA r = −0.13, BRCA r = −0.06, PRCA r = −0.04, CLL r = −0.01).

Upregulated genes at the intersections formed by PD and its SDDCs were enriched in immune-related processes, including leukocyte and neutrophil degranulation, myeloid leukocyte, macrophage, and mircroglia activation, and Toll-like receptor signaling pathway, and in processes linked to phagocytosis and blood vessel morphogenesis (Figure 4A). 

Genes downregulated in both PD and its SDDCs were enriched in processes linked to oxidative phosphorylation and ATP synthesis coupled electron transport. Genes that were downregulated in both PD and BRNA were also heavily enriched in related neuronal processes, such as the chemical synaptic transmission and the synaptic vesicle cycle (Figure 4B). 

Among inverse associations with cancers, genes upregulated in PD and downregulated in its ODDCs were enriched pathways related to the positive regulation of locomotion and cell migration, as well as angiogenesis and the positive regulation of apoptotic processes. Genes upregulated in PD and downregulated in LGCA were also enriched in myeloid leukocyte activation and phagocytosis (Figure 4C). 

Genes that were downregulated in PD and upregulated in inversely associated cancer types were enriched in mitochondrial processes, such as mitochondrial translation and mitochondrial gene expression. In the case of lung cancer, enrichment in oxidative phosphorylation-related genes was also observed. Finally, genes linked to the negative regulation of cell cycle G2/M phase transition were also downregulated in PD and upregulated in lung and breast cancers (Figure 4D). 

### 3.3. Validation of the Intersection Analyses Using an Alternative Set of Cancer Data

The cancer datasets used for validation consisted of 7361 samples comprising 6717 cases and 644 controls from 17 tumor types, including 15 of the cancer types included in the array-based analyses, which were obtained from TCGA. Not all cancer types included in the array-based analyses were available. The data for leukemias, lymphomas, melanoma, and ovarian cancer did not include matched normal tissue. Therefore, differential gene expression analysis could not be carried out for these cancers. In addition, some tumor types were represented by more than one dataset. For instance, lung cancer was divided into two datasets: lung adenocarcinomas and lung squamous cell carcinomas. Supplementary Table S2 shows the study and sample characteristics of the cancer datasets used for validation and the number of DEGs identified in each differential gene expression analysis. The intersection analysis results carried out using the differential gene expression analyses results derived from the alternative source of cancer data (TCGA) and the array-based NDG data served to validate the direct associations observed between AD and brain cancers and the inverse associations between AD and bladder, breast, lung, liver, and prostate cancers in the array-based analyses. The direct associations between PD and brain and kidney cancers were also validated, as were the negative associations between PD and breast, lung, and prostate cancers. Supplementary Figures S3 and S4 show the intersection analysis results obtained using independent cohorts of cancer samples derived from TCGA for AD and PD, respectively.

### 3.4. Pathways and Consensus Co-Expression Modules Deregulated in Both Neurodegenerative Disorders and Cancer

#### 3.4.1. Gene Set Enrichment Analyses (GSEA) Results

Gene Set Enrichment Analysis was carried out for each disorder using the full list of genes ordered by their z-scores (computed by Choi’s meta-analysis method) as input. Compared to traditional overrepresentation enrichment analysis methods, GSEA makes use of the complete profile and is able to detect small but consistent changes in the expression of genes linked to specific biological processes. The presented results include the Normalized Enrichment Score (NES) and the adjusted *p*-value (p-adj). GSEA, carried out using the Hallmark molecular signature database, showed that genes upregulated in neurodegenerative disorders tended to be enriched in immune system-related processes, including the inflammatory response (AD: NES = 2.12, p-adj = 4.46 × 10^−8^, PD: NES = 1.99, p-adj = 8.68 × 10^−7^), allograft rejection (AD: NES = 2.09, p-adj = 7.03 × 10^−8^, PD: NES = 1.44, p-adj = 7.95 × 10^−3^), and IL6-JAK-STAT3 signaling (AD: NES = 2.19, p-adj = 4.69 × 10^−6^, PD: NES = 2.09, p-adj = 1.26 × 10^−5^), among others. Downregulated genes were enriched in several processes, including oxidative phosphorylation (AD: NES = −3.37, p-adj = 2.50 × 10^−9^, PD: NES = −3.09, p-adj = 5.00 × 10^−9^), protein secretion (AD: NES = −2.31, p-adj = 4.14 × 10^−8^, PD: NES = −2.37, p-adj = 6.87 × 10^−9^), mTORC1 signaling (AD: NES = −2.19, p-adj = 6.78 × 10^−9^, PD: NES = −1.9, p-adj = 1.67 × 10^−6^), and MYC targets (AD: NES = −2.54, p-adj = 2.50 × 10^−9^, PD: NES = −1.98, p-adj = −1.98). The canonical pathway (C2) and GO analyses showed that altered expression patterns in additional biological processes were present in AD and PD brains, including the downregulation of genes linked to cell cycle checkpoints (AD: NES = −2.16, p-adj = 5.07 × 10^−9^, PD: NES = −1.88, p-adj = 5.27 × 10^−6^), the proteasome (AD: NES = −2.64, p-adj = 7.66 × 10^−9^, PD: NES = −2.59, p-adj = 7.41 × 10^−9^), and autophagy (AD: NES = −2.19, p-adj = 2.24 × 10^−6^, PD: NES = −1.84, p-adj = 4.88 × 10^−4^), among others.

**Figure 5:**
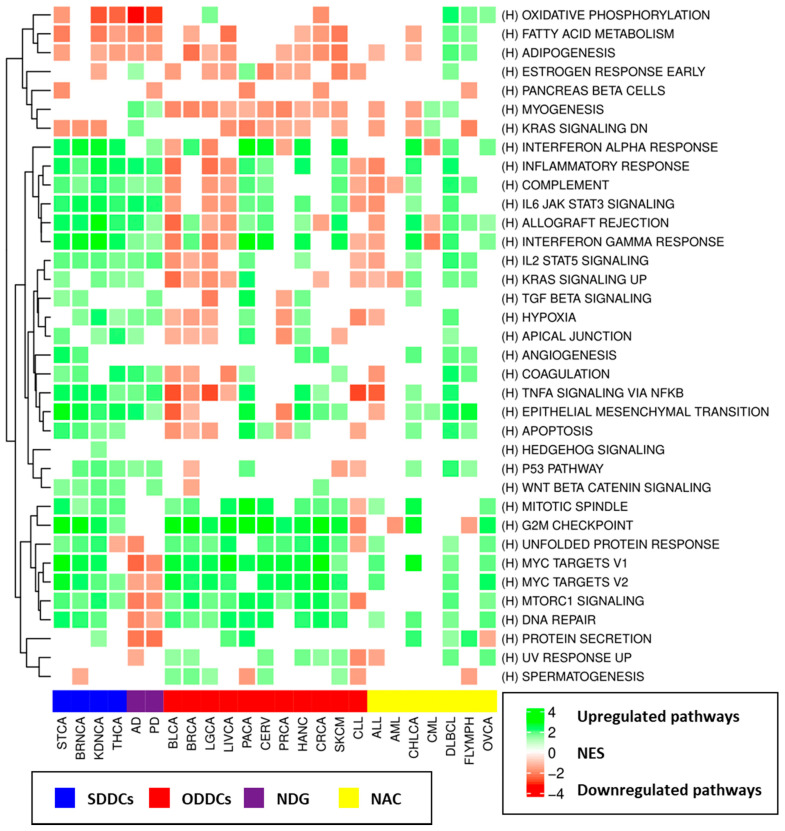
Hallmark molecular signatures in GSEA enrichment results for all studied disorders. Green and red cells indicate the upregulation and downregulation of pathways, respectively, with color intensity being proportional to NES values. Cancers showing direct (same direction) transcriptomic associations of deregulation with AD and/or PD (SDDCs) are coded in blue in the annotation bar below the heatmap, whereas cancers exhibiting inverse (opposite-direction) associations of transcriptomic deregulation with AD and/or PD (ODDCs) are coded in red. NDGs are annotated using purple. The yellow bar indicates cancers with no transcriptomic associations with AD or PD, termed as no associated cancers (NAC).

The most frequently upregulated hallmark gene sets in cancers were MYC targets v1, E2F targets, and MYC targets v2, which were upregulated in 17, 17, and 16 site-specific cancers, respectively. mTORC1 signaling and G2M checkpoints were upregulated in 16 cancers, and DNA repair, glycolysis, unfolded protein response, and E2F targets were upregulated in 15 cancer types. The most common downregulated pathways in cancer were bile acid metabolism, which was downregulated in 13 site-specific cancers, and KRAS signaling. Figure 5 summarizes the GSEA enrichment analysis results with the up- and downregulation status for all studied disorders and the Hallmark gene sets, and Supplementary Files S4–S6 provide the complete GSEA results for all studied disorders and all employed sources of molecular signatures.

Pathways that were upregulated in both AD and its SDDCs were linked to immune function, including cytokine–cytokine receptor interaction, interleukin 4 and interleukin 13 signaling, IL6-JAK-STAT3 signaling, inflammatory response, and allograft rejection, as well as other processes, such as extracellular matrix organization, epithelial–mesenchymal transition, and coagulation. Pathways that were downregulated in both AD and its SDDCs differed between THCA and BRNCA. While pathways that were downregulated in both AD and BRNCA were mainly linked to neuronal-related processes, such as the neuronal system and transmission across chemical synapses, those that were downregulated in both AD and THCA were mainly linked to oxidative phosphorylation and the citric acid cycle.

Pathways that were upregulated in AD and downregulated in its ODDCs included myogenesis, KRAS signaling, allograft rejection, and the complement cascade, among others (downregulated in at least three ODDCs), whereas pathways that were downregulated in AD and upregulated in its ODDCs included MYC targets, mTORC1 signaling, cell cycle checkpoints, DNA repair, unfolded protein response, proteasome, and stabilization of p53 (upregulated in all eight of AD’s ODDCs). Pathways linked to ATP synthesis through the electron transport chain were downregulated in AD and upregulated in LGCA.

As in the case of AD, immune system-related pathways were upregulated in both PD and its SDDCs, whereas pathways that were downregulated in both diseases included oxidative phosphorylation and the neural system. Pathways upregulated in PD and downregulated in its ODDCs presented high variability in the different cancers and included ribosome and translation-related pathways in BRCA; immune system-related pathways, such as signaling via NF-kB, inflammatory response, and interferon-gamma response, in LGCA; and epithelial-to-mesenchymal transition and extracellular matrix organization in PRCA. Finally, pathways downregulated in PD and upregulated in its ODDCs were linked to mTORC1 signaling, cell cycle checkpoints, mitochondrial translations, proteasomal functions, and stabilization of p53, among others. Oxidative phosphorylation was also downregulated in PD and upregulated in LGCA.

Supplementary Tables S3–S22 show the pathways that were deregulated (FDR < 0.01) in both AD or PD and specific cancers with significant transcriptomic associations (SDDCs and ODDCs). We developed a Shiny application designed to easily visualize the GSEA analysis results identifying biological processes and pathways altered in each specific disorder and those jointly deregulated in NDG and cancer for the three molecular signature databases employed in our analyses (Hallmarks, Canonical Pathways, and Gene Ontology). It allows filtering by different parameters (e.g., NES, FDR, and direction of the observed deregulation), as well as an interactive network-based visualization of the significant transcriptomic associations observed between AD, PD, and cancer. The application can be found on the following website: http://disease-perception.bsc.es/ndg_cancer_comorbidities/. The application source code is available at the following GitHub repository: https://github.com/bsc-life/neurodegenerative_diseases-cancer_comorbidities (accessed on 7 June 2021).

#### 3.4.2. Consensus Weighted Gene Co-Expression Network Analysis (WGCNA) Analysis Results

Consensus co-expression module analyses identified a total of 691 modules, of which 448 were significantly associated with disease status: 249 presented significant positive correlations with disease status, and 199 presented significant negative correlations. Supplementary Table S23 shows the number of modules identified in the consensus module co-expression analysis of each disorder. By default, WGCNA assigns a color as a name for each identified co-expression module. To discriminate between modules named with the same color in different disorders, we added the disease abbreviation to all modules identified for a particular disease.

Among neurodegenerative disorders, several co-expression modules enriched in immune system-related processes and specific cell-type markers were positively correlated with disease status, including AD_brown (r = 0.31, p-adj: 5.15 × 10^−5^), which was enriched in the immune response (GO:0006955; p-adj = 6.31 × 10^−9^) and cell type markers for macrophages (p-adj: 1.80 × 10^−6^) and microglia (p-adj: 7.68 × 10^−5^), and PD_black (r = 0.3, p-adj: 2.93 × 10^−3^), which was enriched in the cytokine-mediated signaling pathway (GO:0019221; p-adj = 1.58 × 10^−5^) and response to cytokines (GO:0034097; p-adj = 1.65 × 10^−5^). A particular PD module, PD_yellow (r = 0.35, p-adj: 1.64 × 10^−4^), was positively correlated with disease status and enriched in myelination (GO:0042552; p-adj = 1.98 × 10^−6^) and oligodendrocyte (p-adj: 2.56 × 10^−27^) markers.

Neurodegenerative disorders presented gene co-expression modules that were negatively correlated with disease status and heavily enriched in mitochondrial activity, ATP synthesis functions, and neural cell-type-specific markers. AD_turquoise (r = −0.39, p-adj: 2.31 × 10^−8^) was enriched in the mitochondrial inner membrane (GO:0005743; p-adj = 8.89 × 10^−34^), ATP synthesis coupled electron transport (GO:0042775; p-adj = 1.57 × 10^−21^), synapse (GO:0045202; p-adj = 1.40 × 10^−23^) functions, and cell type markers for interneurons (p-adj: 1.08 × 10^−7^) and neurons (p-adj: 3.07 × 10^−9^), whereas PD_turquoise (r = −0.41, p-adj: 1.49 × 10^−6^) was enriched in mitochondrial ATP synthesis coupled electron transport (GO: 0042775; p-adj = 3.28 × 10^−25^), dopaminergic neurons (p-adj: 6.11 × 10^−3^), and interneurons (p-adj: 1.57 × 10^−4^).

Most cancers presented gene co-expression modules that were positively correlated with disease status and enriched in cell-cycle-related functions. A non-comprehensive list of these instances includes BLCA_turquoise (r = 0.53, p-adj: 1.10 × 10^−17^), enriched in the cell cycle (GO:0007049; p-adj = 9.88 × 10^−69^); BRCA_green (r = 0.43, p-adj: 2.03 × 10^−64^), enriched in the cell cycle (GO:0007049; p-adj = 3.98 × 10^−115^); BRNCA_brown (r = 0.35, p-adj: 8.62 × 10^−37^), enriched in the cell cycle (GO:0007049; p-adj = 3.41 × 10^−97^); and CHLCA_brown (r = 0.52,p-adj: 1.13 × 10^−11^), enriched in the mitotic cell cycle process (GO:1903047; p-adj = 1.81 × 10^−64^).

Many of the studied cancer modules that had significant negative correlations with disease status were enriched in biological processes and cell type markers characteristic of healthy tissues, suggesting that dedifferentiation or tissue substitution has occurred. For instance, for genes in the CERV_yellow module (r = −0.58, p-adj: 3.14 × 10^−13^), the top negatively correlated co-expression module in the cervical cancer analysis was enriched in biological processes linked to cornification (GO:0070268; p-adj = 4.56 × 10^−28^), keratinocyte differentiation (GO:0030216; p-adj = 3.12 × 10^−26^), and epidermis development (GO:0008544; p-adj = 1.17 × 10^−25^), as well as in keratinocyte (p-adj: 2.39 × 10^−13^), epithelial (p-adj: 1.03 × 10^−3^), and basal (p-adj: 3.11 × 10^−3^) cell-type-specific markers. The BRNCA_blue module (r = −0.55, p-adj: 1.79 × 10^−100^) was enriched in biological processes linked to synapse (GO:0045202; p-adj = 2.59 × 10^−36^), axon (GO:0030424; p-adj = 6.09 × 10^−33^), and neuron projection (GO:0043005; p-adj = 1.71 × 10^−27^) and to interneural (p-adj: 4.59 × 10^−10^) and neural (p-adj: 6.24 × 10^−7^) markers. LGCA_turquoise (r = −0.74, p-adj: 1.98 × 10^−169^) and LGCA_pink (r = −0.63, p-adj: 1.73 × 10^−100^) were enriched in pulmonary alveolar type I cells (p-adj: 2.54 × 10^−10^) and pulmonary alveolar type II cells (p-adj: 8.14 × 10^−5^), respectively. Supplementary File S7 shows all disease-associated modules and the enrichment analysis results for biological functions and cell-type-specific markers.

We searched for the presence of significant overlaps between consensus co-expression modules that were significantly associated with neurodegenerative disorders and cancers. Two hundred and seventy-seven significant overlaps were found between AD and cancer-associated modules, whereas one hundred and seventy-seven module overlaps were observed in the case of PD. The AD-associated modules that overlapped with the most cancer modules were AD_turquoise, AD_brown, AD_purple, AD_darkgreen, and AD_skyblue. AD_turquoise was negatively correlated with AD disease status (r = −0.39, p-adj: 2.31 × 10^−8^) and enriched in ATP synthesis coupled electron transport (GO:0042773; p-adj = 1.57 × 10^−21^) and interneuron (p-adj: 1.08 × 10^−7^) and neuron (p-adj: 3.07 × 10^−9^) cell-type-specific markers. This module presented significant overlaps with 86 cancer modules, of which 25 and 61 presented negative and positive correlations with cancer, respectively. For instance, significant overlaps were found between AD_turquoise and (i) BRNCA_blue (r = −0.55, p-adj: 1.79 × 10^−100^), which was enriched in neuronal-related processes and markers, synapse (GO:0045202; p-adj = 2.59 × 10^−36^), interneuron (p-adj: 4.59 × 10^−10^), and neuron (p-adj: 6.24 × 10^−7^) markers; (ii) CRCA_magenta (r= −0.44, p-adj: 1.61 × 10^−60^), which was enriched in oxidative phosphorylation (GO:0006119; p-adj = 4.91 × 10^−60^) and mitochondrial ATP synthesis coupled electron transport (GO:0042775; p-adj = 2.99 × 10^−53^); (iii) KDNCA_turquoise (r = −0.78, p-adj: 4.95 × 10^−155^), which contained genes linked to mitochondrial ATP synthesis coupled electron transport (GO:0042775; p-adj = 3.530167 × 10^−15^); and (iv) THCA_salmon (r = −0.32, p-adj: 7.71 × 10^−6^), which was also enriched in mitochondrial ATP synthesis coupled electron transport (GO:0042775; p-adj = 2.47 × 10^−50^). By contrast, significant overlaps were also found between AD_turquoise and 61 consensus modules that were positively correlated with cancer status, which included many instances of ODDC-related modules, such as BLCA blue, turquoise, and black; BRCA_red; CERV_darkolive; CRCA cyan and royalblue; LGCA_royalblue; LIVCA_darkmagenta; and PACA_white, among others. These modules were enriched in mitochondrial genes, oxidative phosphorylation, and cell-cycle-related processes. AD_brown (r = 0.31, p-adj: 5.15 × 10^−5^), which was enriched in biological processes linked to cell adhesion (GO:0007155; p-adj = 6.62 × 10^−12^) and immune response (GO:0006955; p-adj = 6.31 × 10^−9^) and in macrophage (p-adj: 1.80 × 10^−6^) and microglia (p-adj: 7.68 × 10^−5^) cell markers, was found to present significant overlaps with several consensus co-expression modules that were both positively and negatively correlated with disease status. Cancer modules that were positively correlated with disease status included BRNCA_yellow (r = 0.12, p-adj: 9.43 × 10^−5^), which was enriched in the immune response (GO:0006955; p-adj = 3.21 × 10^−147^), macrophages (p-adj: 6.19 × 10^−24^), and microglia (p-adj: 1.56 × 10^−13^), and THCA_blue (r = 0.42, p-adj: 1.26 × 10^−10^), which was enriched in the immune system process (GO:0002376; p-adj = 1.96 × 10^−104^) and macrophages (p-adj: 1.79 × 10^−36^). Cancer modules that displayed a significant overlap with AD_brown and were negatively correlated with cancer status included modules related to PRCA, PACA, LIVCA, LGCA, CRCA, BRCA, and BLCA, among others.

In addition, AD_skyblue (r = −0.24, p-adj: 5.26 × 10^−3^), which was enriched in biological processes such as protein folding (GO:0006457; p-adj = 1.58 × 10^−11^), unfolded protein binding (GO:0051082; p-adj = 7.77 × 10^−9^), response to topologically incorrect protein (GO:0035966; p-adj = 9.13 × 10^−8^), and chaperone complex (GO:0101031; p-adj = 1.13 × 10^−7^), presented significant overlaps with 17 upregulated cancer-related co-expression modules. The PD-associated co-expression modules with the largest number of overlaps with cancer modules were PD_turquoise, PD_green, PD_black, and PD_yellow. In general, PD and AD followed similar patterns of module overlap with cancers. The complete information regarding the analyses of consensus co-expression module overlap for both AD and PD is provided in Supplementary Figures S5–S7 and Supplementary File S6.

### 3.5. Interactome-Based Overlaps and Genetic Correlation Analysis Results

#### 3.5.1. Interactome-Based Overlap Analyses Results

To ascertain whether genes associated with each studied disorder tended to cluster in nearby regions of the human interactome, we computed measures of network localization using lists of disease-associated genes and variants derived from DisGeNet, PheGenI, and eDGAR under different settings (see Supplementary Method Section 1.13). Supplementary Tables S24 and S25 show the disease-associated genes and variant genes previously linked to each studied disorder under relaxed and stringent settings. In general, AD and PD genes tended to cluster together in the same region of the human interactome, whereas diverse results were observed for cancer-associated genes, which depended on the selected interactome and the stringency level applied for gene selection. The number of cancer types with average intra-disease distances that were lower than expected by chance ranged between 12 and 21, depending on the tested settings. Supplementary Tables S26 and S27 include the results of both tested network localization measures ($d_{aa}$ and S) for disease-associated genes and variant genes obtained under the different analysis settings. Then, we computed interactome-based measures of network separation for each possible disease pair. We did not find evidence of interactome-based overlaps in the set of disease-associated genes between AD or PD and any cancer type under any of the tested settings, suggesting that neurodegenerative disorders and cancers do not present overlapping disease modules at the level of the human interactome. Supplementary Table S28 includes the significant interactome-based overlaps identified under the different tested settings.

#### 3.5.2. Cross-Trait LD Score Analyses Results

To determine whether the same genetic variability could be involved in the modulation of both neurodegenerative disorders and cancers, we computed the genetic correlations between them using cross-trait LD score regression and GWAS summary statistics data from previously published studies to uncover the role of genetic variability in each disorder. We were able to identify and retrieve GWAS summary statistics from 15 studies. The implication of genetic variability was explored for AD risk in three studies and PD risk in two studies. We could only obtain datasets for six site-specific cancers, namely, BRCA (three studies), PRCA (two studies), CRCA (one study), OVCA (one study), SKCM (two studies), and LGCA (one study). Table 2 summarizes the information on the available datasets included in the cross-trait LD score regression analysis. Significant positive genetic correlations ($r_{g}$) were observed between pairs of studies targeting the same disorder, including AD_2 and AD_3 ($r_{g}$ = 0.92, p-val = 1.60 × 10^−10^) and PD 1 and PD 2 ($r_{g}$ = 0.86, p-val = 3.51 × 10^−41^), among others. Significant genetic correlations were also observed between AD 3 and PD 1 ($r_{g}$ = 0.21, p-val = 1.21 × 10^−2^), as well as between different cancer types, such as BRCA 2 and OVCA 1 ($r_{g}$ = 0.23, p-val = 2.00 × 10^−4^), among others. Finally, significant genetic correlations were found between PD 1 and PRCA 2 ($r_{g}$ = 0.09, p-val = 3.16 × 10^−2^) and SKCM 2 ($r_{g}$ = 0.14, p-val = 4.41 × 10^−2^) and between PD 2 and PRCA 3 ($r_{g}$ = 0.16, p-val = 4.44 × 10^−2^). Supplementary Table S29 shows the full set of significant genetic correlations identified.

### 3.6. Transcriptomic Effects of Drugs Indicated for the Treatment of Neurodegenerative Disorders and Cancers

Next, we investigated the potential role of drugs indicated for the treatment of cancer and neurodegenerative disorders in their comorbidities. With this aim, we first obtained the differential gene expression consensus signatures of the indicated drugs using LINCS L1000 data. These signatures provide information regarding the gene expression changes driven by treatment with each indicated drug. Then, correlations were computed between the consensus signatures and the differential gene expression profiles of the studied neurodegenerative disorders and cancer types.

High-precision set queries in MEDI-AN yielded 272 indications for the studied disorders, encompassing 158 unique drugs. Level 5 gene expression signatures for 91 out of the 158 drugs were identified in LINCS L1000. These signatures are generated by treating different cell lines with each perturbagen at different concentrations and for different exposure times. The results are then compared to appropriate controls, resulting in multiple differential gene expression signatures. For each LINCS perturbation, we combined all available signatures into one consensus gene expression signature, as detailed in Supplementary Methods Section 1.17.2.

Supplementary Table S30 shows the drugs indicated for the treatment of each disorder for which LINCS L1000 consensus signatures were available. Correlations were computed between each drug-induced consensus signature and the differential gene expression profiles of each studied disorder obtained through DEG meta-analysis. Several drugs indicated for the treatment of neurodegenerative disorders presented negative correlations (Spearman’s < −0.2) with the differential gene expression profiles of different cancer types. For instance, the profile of carbidopa (DB00190), an inhibitor of dopamine decarboxylase used for the treatment of PD, was negatively correlated with the differential gene expression profiles of PACA and CHLCA. Donepezil (DB00843), a reversible cholinesterase inhibitor used in AD treatment, was negatively correlated with CERV, CHLCA, LIVCA, PACA, and STCA. Galantamine (DB00674), an allosteric potentiating ligand of human nicotinic acetylcholine receptors, presented negative correlations with the differential gene expression profiles of nine cancers (BRNCA, CERV, CHLCA, CRCA, DLBCL, HANC, LIVCA, PACA, and STCA), whereas selegiline, a monoamine oxidase inhibitor used as an antiparkinsonian and anti-depressive, was negatively correlated with eight cancer types (BRCA, CERV, CHLCA, DLBCL, FLYMPH, LIVCA, PACA, and STCA).

GSEA showed that carbidopa had the potential to downregulate genes linked to oxidative phosphorylation (NES = −2.81, p-adj = 9.76 × 10^−9^) and eukaryotic translation (NES = −3.17, p-adj = 9.76 × 10^−9^), among other processes, and to upregulate genes linked to immune function, such as the complement cascade (NES = 2.08, p-adj = 5.75 × 10^−5^) and the cytokine–cytokine receptor interaction (NES = 1.89, p-adj = 1.07 × 10^−6^). Donepezil treatment produced the downregulation of cell-cycle-related processes, such as cell cycle checkpoints (NES = −2.18, p-adj = 7.60 × 10^−9^), as well as genes linked to other processes, such as splicing and oxidative phosphorylation (NES = −2.53, p-adj = 4.28 × 10^−8^). Galantamine treatment also appeared to lower the expression levels of genes related to oxidative phosphorylation (NES = −3.77, p-adj = 2.49 × 10^−9^) and the cell cycle (NES = −2.09, p-adj = 9.04 × 10^−6^) and also affected other processes, such as those linked to the proteasome (NES = −3.42, p-adj = 2.49 × 10^−9^) and the spliceosome (NES = −3.80, p-adj = 1.80 × 10^−9^), among others. Similar sets of genes were affected by selegiline treatment. 

By contrast, the profiles of several drugs indicated for the treatment of neurodegenerative disorders presented positive correlations (Spearman’s > 0.2) with the differential gene expression profiles of different cancers. Entacapone, a selective and reversible inhibitor of catechol-O-methyltransferase, was positively correlated with eight cancer types (BRNCA, CERV, CHLCA, DLBCL, FLYMPH, LIVCA, PACA, and STCA). Pergolide was positively correlated with 11 (BRNCA, CERV, CHLCA, CRCA, DLBCL, FLYMPH, HANC, KDNCA, LIVCA, PACA, and STCA), and valproic acid, an anticonvulsant also used to control agitations in patients with dementia, was positively correlated with the differential gene expression profiles of eight cancers (BRNCA, CERV, CHLCA, CRCA, DLBCL, LIVCA, PACA, and STCA). Entacapone treatment was found to increase the expression levels of ribosomal genes, as well as DNA replication pre-initiation and p53 stabilization genes, among others, and valproic acid treatment was found to upregulate proteasomal and p53 stabilization genes.

Several drugs indicated for cancer treatment presented negative correlations with AD profiles, including the aromatase inhibitor exemestane (r = −0.28) used for the treatment of BRCA, the progestin medication megestrol (r = −0.27), the alkylating agent thiotepa (r = −0.25), tretinoin (r = −0.25), and estradiol (r = −0.24). Eight drugs indicated for the treatment of diverse cancers presented positive correlations with the differential gene expression profile of AD, including medroxyprogesterone (r = 0.27), temozolomide (r = 0.26), chlorambucil (r = 0.26), and testosterone (r = 0.25), among others. No drugs indicated for cancer presented correlations with PD above the selected threshold. Exemestane treatment was linked to the upregulation of genes related to oxidative phosphorylation (NES = 3.42, p-adj = 4.52 × 10^−9^), ribosomes (NES = 4.33, p-adj = 4.52 × 10^−9^), and DNA replication (NES = 3.20, p-adj = 4.52 × 10^−9^) and the downregulation of genes linked to immune function, such as cytokine–cytokine receptor interactions (NES = −1.88, p-adj = 4.52 × 10^−9^), JAK- STAT signaling (NES = −2.03, p-adj = 5.56 × 10^−8^), and Toll-like receptor signaling (NES = −1.98, p-adj = 1.54 × 10^−6^), among others. Similar results were observed for megestrol and estradiol. 

**Figure 6:**
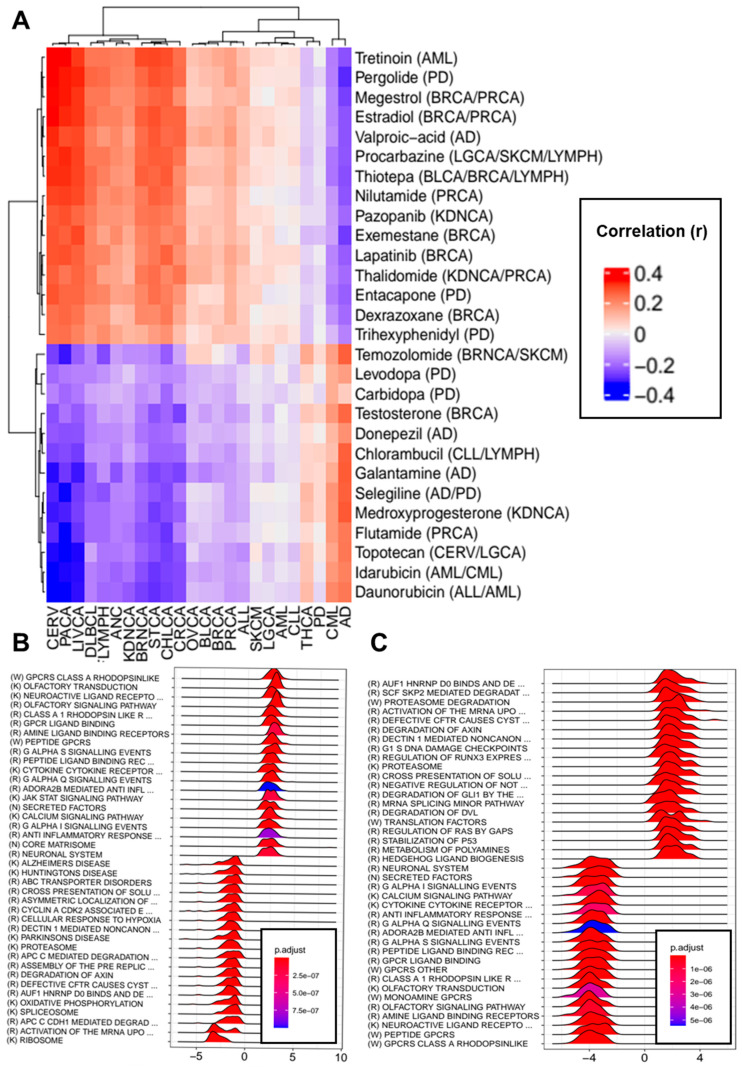
(**A**) Top associated drugs indicated for cancer treatment based on their correlations with AD, PD, and cancer signatures. Red and blue represent positive and negative correlations, respectively. The disorder/s for which a particular drug is indicated can be found between brackets after the drug name. (**B**) Riddle plot depicting the top 10 up- and downregulated pathways induced by treatment with galantamine. (**C**) Riddle plot depicting the top 10 up- and downregulated pathways induced by treatment with exemestane.

Figure 6A shows the indicated drugs with the highest correlations, and Supplementary Figure S6B,C show the top 10 up- and downregulated pathways induced by galantamine and exemestane treatment, respectively. Supplementary File S8 presents the complete results of the correlation analyses between the indicated drugs and disease signatures, and Supplementary File S9 contains the complete gene set enrichment analysis results for NDG disorders and drugs indicated for cancer that had absolute correlation values higher than 0.2 for at least one member of the other disease set.

## 4. Discussion

We observed diverse patterns of transcriptomic deregulation shared between AD, PD, and the 22 studied tumor types. BRNCA and THCA presented significant direct (same direction) associations of transcriptomic deregulation with both AD and PD. Direct associations of transcriptomic deregulation were also observed between AD and PD and KDNCA and STCA. In contrast, AD and PD presented inverse (opposite direction) associations of transcriptomic deregulation with BRCA, LGCA, and PRCA. In addition, AD also presented inverse associations of transcriptomic deregulation with BLCA, CRCA, HANC, LIVCA, PACA, and SKCM. All associations were validated using alternative cohorts of cancer samples derived from TCGA, with the exception of the direct associations between AD and PD and THCA and the inverse associations between AD and CRCA, HANC, PACA, and SKCM. Our results are in agreement with previously reported transcriptomic association studies [51,52] despite differences in the study designs and included cohorts. Interestingly, a number of pairs of NDG disorders and site-specific cancers that showed significant inverse transcriptomic associations in our analyses have presented inverse comorbidity patterns at a population level in epidemiological research. For example, a reduced risk of lung cancer has been observed in patients with AD or PD [2,10,18,19,20,21,22,23,24,33,34], and reduced risks of prostate and liver cancers have been detected in PD and AD patients, respectively [2,18,20,21,23,24,33,35]. In addition, the significant direct transcriptomic association found between PD and brain cancers is consistent with several observational studies that reported an increased risk of brain cancer in PD patients [22,41,42]. In contrast, some results of our transcriptomic analyses are not in agreement with well-documented epidemiological associations. For example, although PD patients are known to have an increased risk of melanoma compared to controls [2], no direct transcriptomic associations were found between them. In general, because of the lack of complete epidemiological and transcriptomic data covering all CNS and site-specific cancer associations, we were unable to test the predictive power that the presence of significant transcriptomic deregulation patterns has on epidemiological associations, which should be explored in future research.

The overrepresentation analyses of genes at significant intersections, together with the results of GSEA and consensus co-expression module analysis, pointed towards several pathways and biological processes that may be involved in the modulation of NDG disorders and cancer comorbidities. Examples include the presence of shared alterations in genes linked to the cell cycle, mTORC1 signaling, mitochondrial dysfunction, p53 signaling, DNA damage, apoptosis, proteasome, autophagocytosis, and immune system-related processes, among others.

Cell cycle-related processes were found to be downregulated in the GSEA analyses results obtained using the canonical pathways and gene ontology molecular signature datasets in both NDG disorders. In contrast, genes upregulated in most of the studied cancers were heavily enriched in cell cycle-related processes. Whereas cycle alterations constitute one of the hallmarks of cancer that allow tumors to acquire sustained malignant growth [71], the role of cell cycle alterations in neurodegeneration seems to be more complex than previously anticipated. An increasing body of evidence indicates that dysfunctional neuronal cell cycle re-entry could trigger apoptosis and precede neurodegeneration in AD and PD [72,73]. In this context, the presence of cell cycle markers in neurons in absence of mitotic structures has been documented [74]. Although further research is needed to elucidate the nature of the cell cycle alterations observed in NDG disorders and its evolution with disease progression, we consider that the joint cell cycle alterations observed in NDG disorders and cancers could represent a candidate to explain the comorbid associations observed between both sets of disorders.

MYC target pathways were found to be downregulated in AD and PD and upregulated in most of the studied cancers. MYC constitutes a family of transcription factors classified as proto-oncogenes, which are involved in the regulation of cell proliferation, cell growth promotion, and the modulation of apoptotic processes. Increased activity or expression of MYC has been reported in more than half of human cancers [75]. Therefore, the opposite patterns of pathway deregulation observed between both NDG disorders and cancer could constitute a molecular substrate for the inverse patterns of comorbidity observed between NDG disorders and cancer at an epidemiological level.

The Hallmark mTORC1 signaling pathway was found to be downregulated in both AD and PD and upregulated in 16 cancer types. The PI3K/AKT/mTOR axis has a well-documented role in the healthy nervous system, where it is involved in neurogenesis, axonal sprouting, dendritic spine growth, and myelination, among other processes [76]. Decreased phosphorylation and total levels of components of the PI3K/AKT/mTOR signaling pathway have been previously reported in AD brains [77], as well as reduced levels of PI3K subunits [78,79]. GSK3β, a major tau-phosphorylation kinase, is inhibited by PI3K-Akt signaling [80]. It has been shown that Aβ oligomers could inhibit the PI3K/AKT pathway leading to GSK3β activation, tau phosphorylation and neuronal death [81]. AKT and phosphorylated AKT levels have also been found to be reduced in the substantia nigra of PD patients [82]. Alterations in the PI3K/AKT/mTOR pathway are thought to be involved in PD pathogenesis and dopaminergic neuronal loss mainly through the regulation of apoptotic pathways [83]. Our differential gene expression meta-analyses showed that the expression levels of AKT3 and the PI3K regulatory subunits PIK3R3 and PIK3R4 were downregulated in both AD and PD, as well as the catalytic subunit PIK3CB. Furthermore, mTOR itself was found to be downregulated in PD brains compared to controls. PI3K/AKT/mTOR activation plays a pivotal role in human tumors, where it is involved in several processes, including cell proliferation, cell survival, metabolic reprogramming, metastasis, and the suppression of autophagy and apoptosis.

Our analyses suggest that downregulated genes in AD and PD are heavily enriched in mitochondrial-related processes, such as oxidative phosphorylation and ATP synthesis through the electron transport chain. Impaired bioenergetic processes have been observed to be a common feature of neurodegenerative disorders [84,85,86]. In cancers, higher rates of glycolysis and the suppression of mitochondrial function, even in the presence of oxygen, are frequently observed, a phenomenon known as the Warburg effect. Recent research has suggested that the Warburg effect is driven by increased demand for NAD+ relative to ATP [87]. Our data indicate that different cancer types present diverse patterns of transcriptomic alterations in genes linked to oxidative phosphorylation and ATP synthesis pathways, with upregulation trends observed for seven of them (BLCA, DLBCL, FLYMPH, LGCA, LIVCA, and OVCA) and downregulation trends identified for five (BRNCA, CRCA, KDNCA, STCA, and THCA). These observations highlight the heterogeneity of energy metabolism alterations in cancer and suggest that they may contribute to the modulation of NDG and cancer comorbidities. The joint downregulation of oxidative phosphorylation-related pathways seems particularly relevant in the direct patterns of transcriptomic deregulation observed between AD, PD and THCA, and between PD and KDNCA. Reductions in cellular respiration and increases in glycolytic pathways are known to take place in thyroid tumors, particularly in poorly differentiated and fast-growing types [88]. In addition, protein levels of complex I elements were reported to be reduced in papillary thyroid carcinomas [89]. Furthermore, oncolytic cell tumors of the thyroid are characterized by the accumulation of defective mitochondrial carrying mutations in elements of the electron transport system complex I [90]. Previous research has also highlighted the importance of metabolic reprogramming in kidney cancers in which the loss of VHL-dependent oxygen sensing results in HIFα stabilization that triggers increases in glycolysis and reductions in the expression of genes linked to the tricarboxylic acid cycle (TCA) [91].

In addition to the joint alterations observed in oxidative phosphorylation-related genes, the Hallmark fatty acid (FA) metabolism pathway was found to be downregulated in AD, PD, and nine of the studied cancers. A closer examination of the top downregulated genes belonging to this pathway revealed that genes linked to pyruvate dehydrogenase activity (PDHB, DLD, and PDHA1), mitochondrial β-oxidation (ACADS, ETFA, ETFB, ECHS1, PCCA, PCCB, ACAT2, and ACOT8), and mitochondrial fatty acid synthesis (AASDHPPT, ACACA, MCAT, MECR, NDUFAB1, OXSM) were downregulated in AD brains compared to controls. The same genes linked to the pyruvate dehydrogenase activity were also found to be downregulated in PD brains compared to controls, as well as other genes linked to lipid metabolism, such as DLD, ALDH1A, IDI1, AUH, HMGCL, RDH11, HMGCS1, SERINC1, ACADM, ACAT2, YWHAH, GSTZ1, NSDHL, HSD17B10, MIF, ELOVL5, CPT1A, ACSL4, HPGD, and IDH1. Two genes linked to the mitochondrial β-oxidation were found to be downregulated in PD (ACADM and PCCB). Among the nine cancer types, seven (BRCA, STCA, KDNCA, LIVCA, HANC, CRCA, and SKCM) presented downregulation in β-oxidation-related genes, whereas three of them (BRCA, LIVCA, and STCA) were also enriched in downregulated genes linked fatty acid biosynthesis. Fatty acid oxidation has been previously found to be downregulated and associated with disease outcome in multiple cancer types [92] and alterations in the metabolism of fatty acids have been linked to neurodegeneration. In particular, the excessive levels of FA produce mitochondrial uncoupling and dysfunction [93]. In this context, β-oxidation insufficiency has been documented in early PD in peripheral tissues [94,95]. In addition, a recent report has shown that the allelic variant ε4 of apolipoprotein E, which constitutes the most important genetic risk factor of late onset AD, is linked to a reduction in the β-oxidation capacity of astrocytes which leads to lipid accumulation in astrocytes and the hippocampus [96].

Our data suggest that p53 signaling could be upregulated in both AD and PD. This is in agreement with previous studies that have reported that increases in p53 activity are a common feature of NDG disorders [97,98]. Increased p53 levels have been found in both human AD patients and animal models [99,100,101]. The same pattern has been observed in the case of PD [102]. In contrast, the results derived from the Hallmark gene set enrichment analysis did not indicate a general downregulation of this pathway in the studied cancers. However, p53 inactivation is a widespread phenomenon in human cancers produced mainly by the acquisition of inactivating mutations of p53 itself which take place in approximately 50% of human tumors [103]. This implies that even if p53 pathway impairment is not detected at a transcriptomic level, it would likely be inactivated in the vast majority of studied cancers. Therefore, the increased activity of p53 observed in both NDG disorders could be an interesting candidate to explain the reduction in all cancer risk reported in AD and PD patients.

Shared alterations in proteasomal and autophagocytic processes may also be involved in the modulation of NDG disorders and cancer comorbidities. Both AD and PD have been previously linked to the downregulation of elements of the ubiquitin–proteasome system, as well as impairments in autophagy; in contrast, tumor cells have often been found to upregulate the ubiquitin–proteasome system [104].

Finally, our data suggest that NDG disorders and cancer share an extensive overlap of deregulated immune system-related processes. Activated microglia have been detected in almost all neurodegenerative disorders, whereas peripheral lymphocyte and monocyte activation has been found in some instances [105]. Additionally, the capacity to evade the host’s immune system is one of the hallmarks of cancer and the cornerstone of immunotherapy [106].

Although our transcriptomic approach provides an interesting descriptive tool to identify genes and pathways jointly downregulated in AD, PD, and cancer, it also presents some important limitations. First, the gene expression data included in our analyses provide a picture of a specific time-point of the disease physiopathology. In the case of NDG disorders, working with post-mortem brain tissues gives us a view that is likely biased towards the disease’s latest stages, preventing us from characterizing the complex changes that take place in the disorders along the temporal axis. Second, the transcriptomic datasets employed were derived from tissues that are composed of a heterogeneous set of cell types. This limits the possibility of determining if the alterations observed are due to changes in the patterns of gene expression of specific cell types or to differences in tissue composition. Further research dedicated to the analysis of transcriptomic deregulation patterns along the temporal axis could allow us to characterize better the alterations of specific disorders, which will result in a more accurate description of jointly altered in NDG and cancer. In addition, the emergence of single-cell RNA-seq studies will allow the identification of specific transcriptomic changes occurring in specific cell-types.

The computation of measures of network localization showed that disease-associated genes and variants tended to be clustered in nearby regions of the human interactomes in AD and PD, as well as in a variable number of cancer types, which depended on the employed analysis setting. The computed measures of network separation did not yield significant overlaps between the disease modules of neurodegenerative disorders and cancers under any of the tested settings, suggesting the lack of interactome-based overlaps between the sets of disease-associated genes and proteins in neurodegenerative disorders and cancers. As Menche and co-workers noted, the human interactome and the sets of disease-associated proteins and genes are still incomplete [57]. Therefore, further research employing more complete versions of the human interactome and highly accurate lists of disease-associated genes will be needed to confirm or refute our observations. In contrast, we were able to identify significant genetic correlations between PD and both PRCA and SKCM, although the observed effect sizes were small. This observation implies that shared genetic variability modulates the risk of developing both disorders. This is particularly interesting in the case of PD and SCKM, as a growing body of evidence suggests that PD patients are at an increased risk of SKCM [17,21,22,23,41,42,43]. The interpretation of the positive genetic correlation found between PD and PRCA is more challenging because epidemiological data suggest that PD patients are at a lower risk of PRCA compared to controls. Previous studies have reported significant genetic correlations between AD and BRCA and LGCA [53]. We could not reproduce any of these associations, presumably due to the use of different datasets. GWAS summary statistics data were obtained for a reduced number of cancer types. We are aware of the existence of additional cancer datasets derived from GWAS studies that could not be obtained due to data access and time constraints. Further research will be needed to complete the picture of genetic correlations between NDG disorders and cancer.

Several drugs indicated for the treatment of neurodegenerative disorders were found to produce transcriptomic alterations that mimicked or reversed those observed in several cancer types. For instance, galantamine and selegiline consensus signatures presented negative correlations with the differential gene expression profiles of nine and eight cancer types, respectively, indicating its potential role in the reduced cancer risk observed in patients with NDG disorders.

Galantamine (DB00674), an allosteric potentiating ligand of human nicotinic acetylcholine receptors, presented negative correlations with the differential gene expression profiles of nine cancers, and selegiline, a monoamine oxidase inhibitor used as an antiparkinsonian and anti-depressive, was negatively correlated with eight cancer types (BRCA, CERV, CHLCA, DLBCL, FLYMPH, LIVCA, PACA, and STCA). Some authors have suggested that acetylcholinesterase inhibitors such as galantamine could be used as potential anti-cancer medications [55]. Several lines of evidence support this possibility; for instance, nicotinic agonists (e.g., tobacco nicotine) are thought to modify the synthesis of antigenic, growth, and neurotrophic factors through perturbations in signaling cascades triggered by nicotinic acetylcholine receptors [31], and Schuller and co-workers proposed that smoking and other factors could affect the signaling of nicotinic acetylcholine receptors by increasing the function of homomeric receptors that stimulate cancer cells [107]. Additionally, selegiline has been shown to induce apoptosis in melanoma cell lines and acute myeloid leukemia cell lines through the inhibition of mitochondrial respiration [108,109].

Finally, we identified a number of drugs indicated for cancer treatment with the potential to reverse the transcriptomic changes observed in AD brain tissues. Among them were two breast cancer drugs (exemestane and estradiol). The use of aromatase inhibitors in BRCA treatment has recently been found to reduce the risk of AD as well as other dementias [110], and new evidence suggests that estradiol replacement therapy could prevent the tau protein from adopting its pathological conformation, helping to prevent AD [111]. Additionally, epidemiological data suggest that estrogen replacement therapy significantly decreases the risk of the onset and development of AD and PD.

## 5. Conclusions

In conclusion, our work provides evidence that shared alterations in biological processes may play a role in AD, PD and cancer associations. Further research will be needed to determine if these observations extend to other NDG disorders. The results suggest the presence of specific instances of shared genetic variability and highlight the potential role of different indicated medications in comorbid associations between the two sets of disorders. The shared transcriptomic alterations identified between NDG disorders and cancer could suggest that the presence of biological substrates underlie the inverse comorbid associations observed between neurodegeneration and both overall and site-specific cancers (i.e., LGCA, BRCA, PRCA), as well as the direct comorbidities observed with particular tumor types (i.e., PD and BRNCA). Further research using animal models and organoids could be key to providing additional insights into the role that some of the processes identified in our work actually play in the comorbid associations between neurodegeneration and cancer. Although the effect sizes were low, the significant genetic correlations observed suggest that shared genetic variability may be involved in the modulation of the risk of both NDG disorders and cancers in specific instances (i.e., PD and SKCM). Future investigation carried out as new GWAS data are released are needed to confirm these findings and to complete the landscape of genetic correlations between NDG disorders and cancer. Finally, our work supports the potential anti-cancer role of some medications indicated for the treatment of NDG disorders, including cholinesterase inhibitors, such as galantamine, and monoamine oxidase inhibitors, such as selegiline, among others. Future experimental research is needed to better characterize the anti-cancer properties of these compounds and to evaluate their inclusion as potential drug repurposing candidates.

## Figures and Tables

**Table 1:** Number of differentially expressed genes found in each meta-analysis.

Disease	Included Studies	No. Samples (Case/Control)	DEGs 0.05/Tested Genes, %	Up 0.05/Down 0.05	DEGs 0.01/Tested Genes, %	Up 0.01/Down 0.01	Mean Q	Mean Tau
AD	7	226 (124/102)	3341/11,536 (28.96%)	1364/1977	1641/11,536 (14.23%)	563/1078	10.59	0.33
PD	6	149 (83/66)	3473/11,714 (29.65%)	1626/1847	2267/11,714 (19.35%)	949/1318	7.24	0.31
ALL	5	1500 (1406/94)	2315/12,436 (18.62%)	1373/942	1387/12,436 (11.15%)	920/467	16.67	0.63
AML	8	1162 (949/213)	581/16,271 (3.57%)	415/166	217/16,271 (1.33%)	163/54	39.52	0.47
BCLA	5	228 (199/29)	5277/16,054 (32.87%)	3209/2068	3069/16,054 (19.12%)	1983/1086	10.41	0.85
BRCA	8	1396 (1164/232)	8362/20,536 (40.72%)	4700/3662	6662/20,536 (32.44%)	3781/2881	33.97	0.46
BRNCA	7	1218 (1124/94)	9757/15,401 (63.35%)	5161/4596	8228/15,401 (53.43%)	4437/3791	19.89	0.35
CERV	4	139 (82/57)	3612/15,683 (23.03%)	2215/1397	2344/15,683 (14.95%)	1517/827	11.73	0.64
CHLCA	4	152 (130/22)	1234/16,111 (7.66%)	766/468	535/16,111 (3.32%)	346/189	15.6	1.55
CLL	6	1021 (884/137)	2547/16,634 (15.31%)	1670/877	1488/16,634 (8.95%)	996/492	62.94	1.24
CML	3	226 (128/98)	1593/15,125 (10.53%)	1115/478	740/15,125 (4.89%)	461/279	6.88	0.33
CRCA	7	1261 (968/293)	9041/13,595 (66.5%)	4793/4248	7872/13,595 (57.9%)	4235/3637	27.98	0.3
DLBCL	3	246 (173/73)	4673/20,503 (22.79%)	3284/1389	3184/20,503 (15.53%)	2339/845	32.7	1.67
FLYMPH	6	214 (147/67)	1945/15,456 (12.58%)	1195/750	854/15,456 (5.53%)	542/312	26.71	1.22
HANC	9	560 (436/124)	6964/14,760 (47.18%)	3617/3347	5280/14,760 (35.77%)	2874/2406	24.94	0.5
KDNCA	8	650 (396/254)	9575/13,828 (69.24%)	4974/4601	8193/13,828 (59.25%)	4426/3767	31.65	0.38
LGCA	5	850 (522/328)	9468/14,718 (64.33%)	5637/3831	7954/14,718 (54.04%)	4695/3259	26.39	0.23
LIVCA	8	839 (383/456)	6624/10,648 (62.21%)	3567/3057	5401/10,648 (50.72%)	2996/2405	26.91	0.23
OVCA	6	222 (154/68)	5241/18,544 (28.26%)	3704/1537	3415/18,544 (18.42%)	2496/919	21.1	0.9
PACA	9	463 (318/145)	8306/14,257 (58.26%)	3938/4368	6270/14,257 (43.98%)	3061/3209	39.27	0.79
PRCA	6	1019 (648/371)	4663/15,169 (30.74%)	2001/2662	3253/15,169 (21.45%)	1378/1875	24.06	0.17
SKCM	5	486 (409/77)	4158/11,651 (35.69%)	2271/1887	2752/11,651 (23.62%)	1453/1299	13.43	0.38
STCA	9	664 (393/271)	7047/15,066 (46.77%)	3766/3281	5243/15,066 (34.8%)	2920/2323	44.96	0..42
THCA	5	245 (139/106)	8662/20,536 (42.18%)	4271/4391	6356/20,536 (30.95%)	2975/3381	10.02	0.39

The results for two different FDR thresholds (FDR < 0.05 and FDR < 0.01) are provided. The number of DEGs and their percentages (with respect to the total number of tested genes) are listed in columns 3 and 5, whereas the numbers of up- and downregulated genes are in columns 4 and 6 for FDR thresholds of 0.05 and 0.01. Columns 7 and 8 show the mean Q and tau values, respectively.

**Table 2:** Characteristics of the studies included in the genetic correlation analyses.

Study	Source	N (Cases/Controls)	Tested SNPs after Merging with SNP List	Mean Chi^2^	Lambda GC	Max Chi^2^	Genome-Wide Significant SNPs	SNP Heritability
AD 1	IGAP [61]	54,162 (17,008/37,154)	1,150,200	1.11	1.09	565.21	165	0.07
AD 2	GR@ACE project [62]	21,235 (11,999/9236)	1,204,123	1.09	1.068	1123.06	59	0.13
AD 3	GWAS catalog [63]	455,258 (71,880AD */383,378)	1,203,908	1.12	1.08	1009.11	320	0.01
PD 1	Nalls et al. [64]	482,730 (33,674/449,056)	1,137,530	1.14	1.09	180.42	276	0.02
PD 2	23andMe [65]	308,557 (6477/302,080)	1,211,658	1.10	1.08	164.95	142	0.02
PRCA 1	GWAS catalog [66]	140,254 (79,148/61,106)	1,206,082	1.51	1.23	846.34	2733	0.16
PRCA 2	UK Biobank	206,770 (6879/199,891)	1,211,361	1.13	1.09	181.15	397	0.03
BRCA 1	BCAC [67]	247,173 (133,384/113,789)	519,352	1.72	1.38	481.43	1389	0.12
BRCA 2	GWAS catalog [68]	139,274 (76,192/63,082)	1,128,758	1.68	1.36	1424.99	2832	0.22
BRCA 3	UK Biobank	245,494 (10,478/235,016)	1,211,361	1.11	1.08	314.15	276	0.02
CRCA	UK Biobank	387,318 (4562/382,756)	1,215,182	1.058	1.052	51.049	22	0.01
OVCA	GWAS catalog [69]	85,426 (16,924/68,502)	1,149,515	1.09	1.06	169.17	209	0.04
SKCM 1	UK Biobank	452,264 (2465/449,799)	1,211,361	1.04	1.03	121.09	144	0.00
SKCM 2	Genomel [70]	32,383 (11,523/20,860)	1,100,284	1.12	1.08	372.76	561	0.17
LGCA	UK Biobank	452,264 (1655/450,609)	1,211,361	1.02	1.01	26.03	0	0.00

* This dataset includes AD patients and individuals with a family history of AD as cases. PGC = Psychiatric Genomic Consortium. BCAC = Breast Cancer Association Consortium.

## Data Availability

All raw data employed in this work were derived from the following public repositories: Gene Expression Omnibus (GEO, https://www.ncbi.nlm.nih.gov/geo/), Array Express (AE, https://www.ebi.ac.uk/arrayexpress/), The Cancer Genome Atlas (TCGA, https://www.cancer.gov/about-nci/organization/ccg/research/structural-genomics/tcga), DrugBank (DB, https://go.drugbank.com/), and the Ensemble MEDIcation Indication Resource (MEDI-an, https://www.vumc.org/cpm/cpm-blog/medi-ensemble-medication-indication-resource-0). The last access to all databases occurred in 1 September 2020.

## Supplementary Materials

The following are available online at https://www.mdpi.com/article/10.3390/cancers13122990/s1. Supplementary File S1: Complete description of materials and methods, Supplementary Tables S1–S30, and Supplementary Figures S1–S7. Supplementary File S2: Complete differential gene expression meta-analysis results for all included disorders. Supplementary File S3: Gene Ontology overrepresentation enrichment analyses results of genes placed at the significant intersections. Supplementary File S4: GSEA results for all studied disorders using the Hallmark molecular signatures. Supplementary File S5: GSEA results for all studied disorders using the C2 canonical molecular signatures. Supplementary File S6: GSEA results for all studied disorders using Gene Ontology molecular signatures. Supplementary File S7: List of consensus co-expression modules significantly correlated with disease status and enrichment analysis results in biological functions and cell-type-specific markers of their gene content for all studied disorders. Supplementary File S8: Correlations between indicated drugs and disease signatures. Supplementary File S9: GSEA results of the differential gene expression profiles of indicated drugs.
