# Supplementary material for: Transcriptomic and Genetic Associations between Alzheimer’s Disease, Parkinson’s Disease, and Cancer
Source: Cancers (Basel). 2021 Jun 15;13(12):2990. doi: 10.3390/cancers13122990 (PMC8232649; doi:10.3390/cancers13122990)
Supplement: Supplementary file 1 [file cancers-13-02990-s001.zip › Supplementary_File_1.pdf]

Article

Transcriptomic and Genetic Associations between Alzheimer's Disease, Parkinson's Disease, and Cancer

Jaume Forés-Martos [1,2,3], Cesar Boullosa [4,†], David Rodrigo-Domínguez [5,†], Jon Sánchez-Valle [6,†], Beatriz Suay-García [2,3], Joan Climent [2,7], Antonio Falcó [2,3], Alfonso Valencia [6,8], Joan Anton Puig-Butillé [9,10], Susana Puig [10,11] and Rafael Tabarés-Seisdedos [1,12,13,*]

1 Biomedical Research Networking Center of Mental Health (CIBERSAM), 28029 Madrid, Spain; jaume.foresmartos@uchceu.es
2 ESI International Chair@CEU-UCH, Universidad Cardenal Herrera-CEU, CEU Universities, San Bartolomé 55, 46115 Alfara del Patriarca, Spain; beatriz.suay@uchceu.es (B.S.-G.); joan.climentbataller@uchceu.es (J.C.); afalco@uchceu.es (A.F.)
3 Departamento de Matemáticas, Física y Ciencias Tecnológicas, Universidad Cardenal Herrera-CEU, CEU Universities, San Bartolomé 55, 46115 Alfara del Patriarca, Spain
4 IES Miguel Catalán, 28823 Coslada, Spain; cesarboullosa@gmail.com
5 Consorcio Hospital General de Valencia, Servicio de Medicina Interna, 46014 Valencia, Spain; darodo@alumni.uv.es
6 Barcelona Supercomputing Center (BSC), 08034 Barcelona, Spain; jon.sanchez@bsc.es (J.S.-V.); alfonso.valencia@bsc.es (A.V.)
7 Departamento de Producción y Sanidad Animal, Salud Pública Veterinaria y Ciencia y Tecnología de los Alimentos, Universidad Cardenal Herrera-CEU, CEU Universities, C/ Tirant lo Blanc 7, 46115 Alfara del Patriarca, Spain
8 Catalan Institution for Research and Advanced Studies (ICREA), 08010 Barcelona, Spain
9 Biochemical and Molecular Genetics Service, Hospital Clínic and August Pi i Sunyer Biomedical Research Institute (IDIBAPS), 08036 Barcelona, Spain; japuig@clinic.ub.es
10 Melanoma Unit, Hospital Clínic, Center for Networked Biomedical Research on Rare Diseases (CIBERER), Carlos III Health Institute (ISCIII); 08036 Barcelona, Spain; spuig@clinic.cat
11 Dermatology Department, Hospital Clínic and August Pi i Sunyer Biomedical Research Institute (IDIBAPS), 08036 Barcelona, Spain
12 Teaching Unit of Psychiatry and Psychological Medicine, Department of Medicine, University of Valencia, Blasco-Ibañez 15, 46010 Valencia, Spain
13 INCLIVA Health Research Institute, 46010 Valencia, Spain
* Correspondence: rafael.tabares@uv.es; Tel.: +44-(0)1865-617-855
† These authors contributed equally to the work.

Citation: Forés-Martos, J.; Boullosa, C.; Rodrigo-Domínguez, D.; Sánchez-Valle, J.; Suay-García, B.; Climent, J.; Falcó, A.; Valencia, A.; Puig-Butillé, J.A.; Puig, S.; et al. Transcriptomic and Genetic Associations between Alzheimer's Disease, Parkinson's Disease, and Cancer. *Cancers* **2021**, *13*, 2990. https://doi.org/10.3390/cancers13122990

Academic Editors: Alexandra Kretz and Annamaria Brioli

Received: 6 May 2021
Accepted: 11 June 2021
Published: 15 June 2021

Publisher's Note: MDPI stays neutral with regard to jurisdictional claims in published maps and institutional affiliations.

Copyright: © 2021 by the authors. Licensee MDPI, Basel, Switzerland. This article is an open access article distributed under the terms and conditions of the Creative Commons Attribution (CC BY) license (http://creativecommons.org/licenses/by/4.0/).

Supplementary

1. Supplementary methods

1.1. Search strategy and inclusion criteria

Searches for available datasets were performed in the following genomic data repositories: Gene Expression Omnibus (GEO, https://www.ncbi.nlm.nih.gov/geo/), Array Express (AE, https://www.ebi.ac.uk/arrayexpress/), and the cancer genome atlas (TCGA, https://www.cancer.gov/about-nci/organization/ccg/research/structural-genomics/tcga).

Transcriptomic datasets should include at least 3 cases and three control samples derived from matching tissues. To ensure that compatible preprocessing strategies could be applied to all studies, we only selected one channel microarray data derived from the most popular Affymetrix, Illumina, and Agilent platforms.

In the case of neurodegenerative (NDG) disorders, we focused on studies carried out using samples obtained from post-mortem brains and excluded datasets derived from other tissues, such as blood or

immortalized cell lines. To reduce the heterogeneity that would be introduced by the inclusion of datasets from different brain regions, we selected a specific brain region for each disorder. The choice was based on two criteria, data availability and strong evidence linking the specific brain region with the physiopathology of the disease under consideration. In the case of Alzheimer's disease, we only selected studies and samples derived from hippocampal tissues. Volume reduction, neural loss, and the presence of neurofibrillary tangles and beta-amyloid deposition are core features of AD found in hippocampal tissues [1]. For PD only datasets with *substantia nigra* samples were included since PD is characterized by progressive degeneration and loss of neurons in this brain region [2].

Cancer studies including primary tumor and matched tissue control samples were selected for inclusion, whereas datasets and samples based on blood, metastatic, or cell lines, were excluded.

Since the presence of studies derived from the same cohort could artificially inflate the differential gene expression meta-analysis results, efforts were made to exclude datasets containing potentially redundant sets of individuals.

Supplementary Table 1 shows all the studies that met inclusion criteria, as well as information regarding the microarray platform and the number of samples (cases and controls) included before and after outlier sample filtering. The summary values of the study-level quality control and each study's inclusion status based on these values are also provided in the same table. **Supplementary Table 2** shows the TCGA datasets employed for the validation step using an alternative cohort of cancer samples.

1.2. Data preprocessing

Datasets generated using Affymetrix platforms were preprocessed as follows: CEL files were retrieved from GEO, AE or directly from the study authors, and the R packages oligo [3] and affy [4] were used to read them and to perform RMA normalization and summarization, which was followed by quantile between-sample normalization and log2 transformation. For Illumina platforms, non-normalized data was loaded to the R's environment using the limma package [5] and a set of custom functions. The Lumi package [6] was used to perform background correction using a normal exponential model fitting with the normexp RMA option selected, followed by quantile normalization and log2 transformation. Agilent data was preprocessed using the limma package [5] following the same preprocessing steps. We transformed dataset-specific IDs into ENTREZ IDs using annotation packages to harmonize probe annotations between different dataset platforms. Probes targeting the same gene were collapsed using the collapseRows function from the WGCNA package [7] and the MaxMean method. RNAseq data derived from TCGA was preprocessed as follows: The TCGAbiolinks package [8] was used to download gene-level counts derived from the HTSeq workflow. The TCGAanalyze_Preprocessing function was then applied with a correlation threshold of 0.6. Next, the TCGAAanalyze_Normalization function was employed to carry out normalization using the gcContent method. TCGAanalyze_Filtering was finally employed to remove mRNA transcripts with low expression values through the quantile method with a cutoff value of 0.30.

1.3. *Quality control and outlier samples removal*

We performed quality control and outlier sample detection using two different approaches for CNS and cancer-related datasets, respectively. Given the low amount of datasets and samples available for the analysis of CNS disorders, we selected a conservative and more computationally intensive approach to detect and remove outlier samples. We used three array quality measures described in detail below. Each one is aimed to evaluate a specific trait related to array quality in each sample. Measure 1 computes distances between arrays. The distance between two arrays a and b d_{ab}, is the mean absolute difference between the expression values of all probes $d_{ab} = mean\,|M_{ai} - M_{bi}|$, where M_{ai} is the value of the i-th probe of array a and M_{bi} is the is the value of the i-th probe of array b. Measure 1 tags a particular sample as an outlier when the sum of the distances to all other arrays $S_a = \sum_b d_{ab}$ is large. Measure 2 examines the array intensity distributions of the arrays. It is expected that the intensity distribution of the arrays has similar positions and widths. Intensity distributions of specific arrays that are very different from the rest of arrays distributions may indicate experimental problems. The Kolmogorov-Smirnov statistic K_a is used to measure the level of agreement between each array's distribution and the distribution of the pooled data. Arrays presenting a large deviation from the pooled intensities distribution are tagged as potential outliers by this measure. Finally, Measure 3 appraises the individual array quality by examining MA plots. where M is defined as $M = log_2(I_1) - log_2(I_2)$ and A is defined as $A = \frac{1}{2}\,(log_2(I_1) + log_2(I_2)\,)$ where I_1 is the intensity of the studied array and I_2 is the intensity of a "pseudo"-array that consists of the median across arrays. It is expected that the mass of the distribution concentrates around the M = 0 axis. Outlier detection was performed by computing Hoedffding's statistic D_a on the joint distribution of A and M for each array. Further information about the sample-level quality assessment can be found in the documentation of the ArrayQualityMetrics package [9]. Samples were removed from datasets only when the three measures tagged them as a potential outlier. To prevent that outlier samples were removed preferentially from cases or controls due to unbalanced study designs, the quality control and outlier detection procedures were carried out independently in each subgroup of samples. For cancer data, given the high amount of available studies and samples, we applied a less conservative outlier detection method, which had the advantage of being less computationally intensive. For each dataset, we computed array-array correlations. Then, the mean inter-array correlation was calculated. If the mean inter-array correlation was higher than 0.9, samples were not removed, and the complete dataset was included for downstream analyses. On the contrary, when the mean inter-array correlation was lower than the selected threshold, samples presenting more than two standard deviations in mean correlation measures with all other arrays were tagged as outliers and removed from the dataset. This procedure was repeated iteratively until a global mean inter-array correlation higher than 0.9 was reached. As in the instance of CNS-derived diseases, to prevent preferential removal of the case or control samples due to unbalanced study designs, the method was applied independently to cases and controls.

1.4. *Study-level quality appraisal*

Study-level quality control was carried out using the MetaQC package [10]. For each disorder, study-level quality control was carried out using all available studies after preprocessing using the set of genes jointly queried in all datasets. MetaQC computes six different quality control measures. IQC evaluates the homogeneity of the co-expression structure across studies, and it is based on the comparison of the co-expression structure of study k to the co-expression structure of all other studies. EQC appraises the consistency of the co-expression information with a pathway database. AQCg assesses the accuracy of biomarker detection by comparing the list of differentially expressed genes derived from study k to the list of differentially expressed genes obtained by performing meta-analysis using all studies except study k. AQCp represents an extension of AQCq where enriched pathways substitute genes. CQCg evaluates the consistency of the gene differential expression ranking from single study to the rank of differentially expressed genes obtained by performing a meta-analysis with all studies except study k, and CQCp assesses the consistency of the enriched pathway ranking. In general low values of each quality measure suggest poor agreement of study k to the rest of the studies indicating that it is a potential outlier, whereas high values indicate good agreement of a specific study with all other studies. Finally, MetaQC employs principal component analysis (PCA) biplots and a standardized mean rank (SMR) summary score to assist in identifying problematic studies. High SMR values indicate potential outlier studies. In the present study, we choose an SMR threshold of 7 as an exclusion criterion for potentially problematic studies.

1.5. Differential gene expression meta-analyses

Differential gene expression meta-analyses are known to increase the statistical power and reduce the noise of gene expression measurements [11]. For each disease, meta-analyses were carried out using Choi's et al. method [12] implemented in the MetaDE package [10]. All meta-analyses were performed using random effect models since high heterogeneity was expected, given our data's biological and technical variability. Genes showing a false discovery rate (FDR) adjusted p-value lower than 0.05 were considered to be differentially expressed.

1.6. Transcriptomic associations between neurodegenerative disorders and cancers

The expression profiles of each neurodegenerative disorder and all the studied cancer types were compared to evaluate the significance of the overlaps between the differentially expressed genes, as previously described [13-15]. For each neurodegenerative disorder and cancer pair, the significance of the four possible intersections formed by the upregulated and downregulated genes was evaluated by means of one-tailed Fisher's exact tests. The intersections were:

•1) Genes upregulated in both a specific NDG disorder and the selected cancer type (Intersection A),

•2) Genes downregulated in both a specific NDG disorder and the selected cancer type (Intersection B),

•3) Genes upregulated in a specific NDG disorder and downregulated in the selected cancer type (Intersection C), and

•4) Genes downregulated in a specific NDG disorder and upregulated in the selected cancer type (Intersection D).

Fisher's test p-values were corrected by multiple testing using the false discovery rate method (FDR). Overlaps showing adjusted p-values lower than 0.05 were considered significant. The background number of genes was set as the number of genes jointly included in the two meta-analyses under consideration, which depended on the platforms included in each meta-analysis. A cancer type was considered to be deregulated in the same direction as a NDG disorder when Intersections A and B were significant, and Intersections C and D were not. These cancer types were referred to as same direction deregulated cancers (SDDCs) and could be candidates for direct comorbidity with the specific NDG disorder. Conversely, a cancer type was considered to be deregulated in the opposite direction from a particular NDG disorder when intersections C and D were significant, but intersections A and B were not. These cancer types were referred to as opposite direction deregulated cancers (ODDCs) and could be candidates for inverse comorbidity with a specific NDG disorder. Additionally, to determine the strength of the overall associations between differential expression profiles, Pearson's correlations were computed using the Z-values obtained from each differential gene expression meta-analysis. Positive correlations suggest similar patterns of differential expression, while negative correlations would indicate opposite patterns.

1.7. Validation of the NDG disorders and cancer associations using an independent cohort of cancers.

The cancer genome atlas was queried for RNAseq-based experiments interrogating tumor samples for the tumor types included in our array-based analysis. Data was downloaded and preprocessed using the R package TCGAbiolinks [8]. DEseq2 [16] was used in order to compute differential gene expression between cases and controls. Results were used to perform intersection analysis following the same methodology explained for arrays in the previous section.

1.8. Weighted co-expression network analyses

We carried out consensus module detection for each disorder using all the datasets employed in the differential gene expression meta-analysis step and the WGCNA package [7]. Consensus modules are clusters of densely interconnected genes present across datasets. First, Bi -correlation networks were constructed for each study resulting in a set of gene-gene correlation matrices. The parameter choices for consensus module detection analyses were based on the parameters selected by the authors of the package in their analysis of a set of eight lung cancer datasets reported in [17]. Bi -correlation matrices were transformed into signed-hybrid adjacency matrices as described in the following equations:

$$a_{ij} = \left[cor\left(x_i, x_j\right)\right]^{\beta} \; for \; cor\left(x_i, x_j\right) > 0$$

$$a_{ij} = 0 \; for \; cor\left(x_i, x_j\right) \leq 0$$

Adjacency matrices are called "hybrid-signed" because they use a combination of hard and soft thresholding. A hard threshold is used for correlation values ≤0 and a soft threshold for values above 0. The parameter β is selected such that the resulting adjacency matrix fits a power-law distribution by using the pickSoftThreshold function implemented in the package. Adjacency matrices. $A = \{a_{ij}\}$ have the following properties:

$$a_{ij} = a_{ji}$$

$$0 \le a_{ij} \le 1$$
$$a_{ii} = 1$$

The next step involves the transformation of adjacency matrices into topological overlap matrices using the following equation:

$$TOM_{ij}(A) = \frac{\sum_{k \neq i,j} a_{ik}a_{kj} + a_{ij}}{min\left(\sum_{k \neq i} a_{ik}, \sum_{k \neq j} a_{jk}\right) + 1 - a_{ij}}$$

The $TOM_{ij}(A)$ matrix is also an adjacency matrix. The topological overlap of two genes reflects their similarity in terms of the commonality of the genes to which they are connected. The TOM leads to a more robust network and larger modules which satisfies the same properties than A. Using the resulting topological overlap matrices derived from each individual dataset, a consensus TOM matrix was generated following the quantile method. For a set of K matrices $TOM^{(1)}, TOM^{(2)}, \dots TOM^{(k)}$. A consensus adjacency matrix is constructed using the quantile method:

$$Quantile_{q,ij}\left(TOM^{(1)}, TOM^{(2)}, \dots TOM^{(k)}\right) = Quantile_q\left(tom_{ij}^{(1)}, tom_{ij}^{(2)} \dots tom_{ij}^{(k)}\right)$$

The consensus TOM was defined as the consensus of the individual TOM matrices with percentile q = 0.25. In the consensus TOM. Two variables are connected with the strength that is common to all input networks. Finally, a dissimilarity matrix based on the consensus TOM matrix is fed as an input for the dynamic tree-cutting algorithm, which identifies co-expressed gene clusters (consensus modules). The steps described above were carried out using the blockwiseConsensusModules with the following parameters set to maxBlockSize = 30000, corType = "bicor", networkType = "signed hybrid", deepSplit = 3, mergeCutHeight = 0.25, and consensusQuantile = 0.25). By default, WGCNA assigns a color as a name for each identified co-expression module. To be able to discriminate between modules named with the same color in different disorders we added the disease abbreviation to all modules identified for a particular disease. Once the consensus modules were identified, each module's eigengenes were computed, and correlations between them and disease status were calculated. A module eigengene is defined as the first principal component of the expression matrix of the genes included in a specific module. It typically explains more than 50% of the module expressions' variance [18]. Significant positive correlations between disease status and a particular module eigengene suggest that genes placed at that particular module tend to be upregulated in cases compared to controls. In contrast, significant negative correlations have the opposite implication (the genes places in the module tend to be downregulated in disease samples compared to controls).

1.9. Module-module overlap analyses

Genes contained in all modules significantly correlated to disease status were identified for all the included disorders. Then, associations between modules of co-expressed genes significantly correlated with disease status were computed for all possible pairwise comparisons through Fisher's exact tests. The p-values of all pairwise module-gene overlap analyses were adjusted for multiple comparisons by FDR.

1.10. Functional and cell type-specific enrichment analyses

Diverse functional analysis methods and sources for sets of genes were used in the different sections. Classic overrepresentation analysis

was carried out to compute enrichment in gene ontology (GO) terms for the genes placed at the intersections observed in the intersection analyses and for genes placed in the detected consensus co-expression modules using the clusterProfiler [19] and anRichment [20] R packages. In the case of the overrepresentation analyses carried out using the genes placed at the overlaps in the intersection analyses, the joint set of genes included in the meta-analyses of both disorders of each given pair was selected as background. For the overrepresentation analyses of genes placed at the identified gene consensus co-expression modules the background was defined as the set of genes jointly included in all the studies including data for a specific disorder. Gene Set Enrichment Analysis (GSEA) was used to identified upregulated and downregulated pathways in each disorder using as an input the list of genes ordered by its Z-score obtained by Choi's differential gene expression meta-analysis. GSEA analyses were carried out using the fGSEA package implementation [21]. The sets of genes were retrieved from the molecular signatures database (MSigDB) [22] and included the hallmarks (H) set of genes, the canonical pathways subset (C2:CP) of the C2 curated set of genes, and the Gene Ontology (C5) set of genes. Finally, enrichment in cell type-specific genetic markers was carried out for each detected consensus module using a collection of cell-specific gene markers derived from PanglaoDB database and hypergeometric tests. This database contains a list of gene expression markers used to define 154 cell types. The obtained p-values were corrected for multiple comparisons using the false discovery rate (FRD) method.

1.11. Sources of human protein-protein/gene-gene interaction data.

1.11.1. Interactome 1

We constructed a human interactome by integrating different sources of protein-protein and gene-gene interactions, including protein-protein interactions (PPI), gene co-expression data, information regarding protein complexes, data from transcription factors and their targets, and associations between genes participating in successive steps of metabolic pathways.

Binary PPIs: PPIs data was derived from the Human Reference Interactome (HuRI) [23]. HuRI is an initiative of the Center for Cancer System Biology at Dana-Farber Cancer Institute, which interrogated all pairwise combinations of human protein-coding genes to identify which are involved in binary protein-protein interactions [24] using yeast two-hybrid (Y2H) screenings.

Co-expressed genes identification: Co-expression partners were identified using data derived from the Genotype-Tissue Expression (GTEx) [25], which contains gene expression datasets derived from multiple healthy human tissues. Twenty-six of them (adipose tissue, adrenal gland, blood, blood vessel, brain, breast, colon, esophagus, heart, liver, lung, muscle, nerve, ovary, pancreas, pituitary, prostate, salivary gland, skin, small intestine, spleen, stomach, testis, thyroid, uterus, and vagina) were represented by more than one hundred samples. Expression data (Transcripts Per Million Reads, TPM) and annotation files were downloaded for those tissues. Then we randomly selected 100 samples from each tissue and computed Pearson's correlations between all pairwise gene combinations'. We considered that two genes were co-expression partners if they presented values of correlation higher than 0.7 in at least 16 tissue types.

Human protein complexes: Data from human protein complexes was downloaded from The comprehensive resource of mammalian protein complexes (CORUM) [26]. Human complexes were selected, and self-interacting genes were removed from the data.

Transcription factor targets data: The transcription factor targets dataset (TRANSFAC) was selected [27, 28] and retrieved from the Harmonizome database [29].

Kinase-substrate interactions: Kinase substrate interactions were retrieved from PhosphoSitePlus [30], which gathers curated data from low and high-throughput phosphoproteomic studies.

Metabolic links: Data regarding metabolic links was obtained from The Kyoto Encyclopedia of Genes and Genomes (KEGG) [31]. In particular, the KEGG PATHWAYS database that includes information regarding genes and their associated pathways and cellular processes was employed. In this database, pathways are represented using graphs where nodes are molecules (protein and compounds), and edges represent nodes' relations. First, the list of KEGG's human metabolic pathways was retrieved, and KGML files, including them, were obtained using the retrieveKGML function from KEGGgraph package [32]. Then, the files were parsed using parseKGML2Graph function included in the same package. Graphs were transformed to adjacency matrices, from which and all pairwise gene-gene associations were extracted.

1.11.2. Interactome 2

The second interactome was obtained from STRING [33]. STRING gathers information about known and predicted protein-protein interactions, including direct or physical interactions and indirect or functional associations. In short, we downloaded the subset of human interactions subset of STRING. The database provides a score ranging from 0 to 1 that informs about the strength of each association's evidence. Only the high-quality associations (those presenting a quality score higher than 0.7) were selected.

1.11.3. Interactome 3

The third interactome was derived from BioGriD [34]. BioGRID includes interaction data compiled through comprehensive curation efforts. We selected all human interactions included in the BioGRID database.

1.12. Sources of disease-associated genes and variants

Sets of genes and variant-genes linked to each studied disorder were extracted from the following repositories. Disease-associated genes were retrieved from DisGeNet [35]. This database gathers information from different sources, including curated data from UniProt, the Comparative Toxicogenomics Database (CTD), Orphanet, the Clinical Genome Resource (CLINGEN), Genomics England, The Cancer Genome Interpreter (CGI), and the Psychiatric disorders Gene association Network (PsyGeNET), as well as, sources of non-curated data. For each disease-gene association, DisGeNet provides a quality score that informs about the evidence supporting it. We excluded not- curated, literature-derived, and animal model-obtained association. Variants associated with each disease were retrieved from The Phenotype Genotype Integrator (PheGenI) [36], which contains information derived from the GWAS catalog, dbGaP, and

dbSNP, among others. Genes mapped to each variant (henceforth variant genes) were selected and used for subsequent analysis. Finally, the eD-GAR database [37], which collects information from OMIM, Humsvar, and ClinVar was also queried. Each included database employs its own dictionaries to identify phenotypes. For each disorder, two lists of disease-associated genes (stringent and relaxed) were created based on the use of two different thresholds for both the DisGeNET association quality score and the p-value associated with the variants linked to disease. The stringent sets were selected using a threshold of 0.6 for DisGeNET quality score and p-values lower than 1e-10 **Supplementary Table 23** shows the identifiers employed to select disease-associated genes and variant genes and the list of disease-associated variants and variant-genes for each disorder under the stringent setting. **Supplementary Table 24** provides the list of disease-associated genes and variant genes obtained under the relaxed selection setting. for the disease variant associations reported in PheGenI. In the case of the relaxed sets, the thresholds were 0.3 and 1e-08. The threshold selection was found to be a large but uneven impact on the number of included disease-associated genes.

1.13. Measures of network localization and statistical significance

For each set of disease-related genes and gene-variants, we computed two network localization measures to determine the degree to which disease proteins aggregate in specific areas of the interactome.

The first measure, termed as the intra-disease average distance $\langle d_{AA} \rangle$, was computed as follows. Let N be a set containing the disease-associated genes and variant genes for a particular disorder. For each element n in N the interactome distance to the next closest element of the set is determined. Then, the average of all shortest distances computed for all the elements of N is computed. Note that the shortest distance is defined as the shortest path between a given pair of nodes (i.e., the lowest number of edges that separate a pair of specific nodes in the network).

To assess the computed intra-disease average distances' significance, we randomly selected 10000 sets of genes of the same size as the original set. The degree distribution of the genes placed at a given disease-associated gene list impacts the computed distances' values. Therefore, we should account for the degrees observed in the original gene set's genes for the randomization process to be accurate.

Given the scale-free topology of human interactomes, low degree nodes are much more abundant than high degree nodes. We grouped nodes within a certain degree interval together to avoid choosing the same high degree nodes repeatedly in the degree-preserving random sampling analysis. Let V be the set of nodes in the interactome and u a particular element of V such that $u \in V$ and let k_u be the set of all nodes with degree u and $B_{i,j}$ the bin containing the nodes with degree equal or higher than i and lower than j. Nodes, with an increasing degree value are included in each bin such that it has at least 100 elements. Therefore, each bin will contain nodes such that, $B_{i,j} = \{u \in V \mid i \leq k_u < j\}$ such that $\left|\left|B_{i,j}\right|\right| \geq 100$. Once the bins have been created, the random sampling strategy proceeds as follows. The degree in the reference interactome of each gene in the original gene set is determined. Then, for each gene, a random gene is selected from the degree-matched bin. This is repeated for all genes in the studied set. The result is a randomly

selected set of genes presenting the same size than the original set and an approximately equivalent degree distribution.

The original intra-disease average distance is then compared to the resulting random distribution of intra-disease average distances. d^{rand} as follows:

$$z_{score}(d) \equiv \frac{d_{AA} - \langle d^{rand} \rangle}{\sigma\left(d^{tand}\right)}$$

Finally, one-tailed p-values are computed by comparing the obtained z-scores to the standard normal distribution.

The second measure, termed module size S represents the largest connected component produced by a particular set of disease-associated proteins in the human interactome (i.e., the highest number of disease proteins directly connected to one another). A random distribution of module sizes S^{rand} was then generated using the same random degree-preserving strategy described for the intra-disease average measures. Z-scores and p-values were also computed using the same methodology.

1.14. Network-based separation between pairs of disorders

The following equation shows the network-based overlap measure proposed by Menche and co-workers [38].

$$S_{AB} = \langle d_{AB} \rangle - \frac{\langle d_{AA} \rangle + \langle d_{BB} \rangle}{2}$$

S_{AB} compares the shortest distances between proteins within each disease $\langle d_{AA} \rangle$ and $\langle d_{BB} \rangle$, to the shortest distances $\langle d_{AB} \rangle$ between A-B protein pairs. Proteins associated to both A and B have d_{AB} = 0. Positive values of S_{AB} indicated separation between disease modules, whereas negative values suggest disease-disease overlaps. A null distribution of S_{AB} values ${S_{AB}}^{rand}$was used to compute the significance of the observed overlaps following the same degree preserving strategy described in the previous section. Z-scores and p-values were computed likewise.

1.15. Cross-trait LD score regression

GWAS summary statistics for the studied disorders were searched in public repositories (GWAS catalog) or directly requested to the authors. We were able to retrieve GWAS summary statistics for all the CNS disorders except for HD. Since HD is a monogenic disease, no genome-wide association studies have been carried out for it. GWAS summary statistics for a subset of the studied cancers were also obtained, including prostate cancer, breast cancer, cervical cancer, colorectal cancer, ovarian cancer, endometrial cancer, thyroid cancer, skin cancer melanoma, and lung cancer.

In order to generate the appropriate files to compute genetic correlations, GWAS summary statistic files were preprocessed using custom scripts in R and the munge_sumstats.py function from the ldsc python package. Note that ldsc interprets the A1 field as the effect allele. In other words, it assumes that the sign of the reported summary statistics (betta values, odds ratios, or log-odds ratios) are oriented in reference to the A1 allele.

Heritability estimates were computed for all disorders. Then disease-disease correlations were computed for those disease pairs that showed individually significant heritabilities.

Cross-trait LD score is a method for estimating genetic correlations from GWAS summary statistics data [39]. It is based on the idea that

GWAS effect-sizes estimates for a given SNP partially incorporate the effects of all SNPs in linkage disequilibrium (LD) with that SNP. For a polygenic trait, SNPs with high LD will have higher X^2 statistics on average than SNPs with low LD. A similar relationship holds if we replace the X^2 statistics of a single study with the product of z-scores from two studies of traits with non-zero genetic correlation.

Under a polygenic model, the expected value of $z_{1j}z_{2j}$ is defined by the following equation:

$$\mathrm{E}[z_{1j}z_{2j}] = \frac{\sqrt{N_1 N_2}\rho_g}{M} l_j + \frac{\rho N_s}{\sqrt{N_1 N_2}}$$

Where N_1 is the sample size of study one, $N_2$is the sample size of study two, ρ_g is the genetic covariance. l_j is the LD score of a particular SNP, N_s is the number of included in both studies, and ρ is the phenotypic correlation among the N_s overlapping samples. This setting allows estimating genetic covariance between two traits by identifying the slope from the regression of $z_{1j}z_{2j}$ on LD score. Sample overlap only affects the intercept of the regression because sample overlap creates spurious correlations between z_{1j} and z_{2j} but does not depend on LD score.

Then normalizing the genetic covariance by the SNP-heritability yields genetic correlation.

$$r_g := \frac{\rho_g}{\sqrt{h_1^2 h_2^2}}$$

Where $h_1^2$the SNP-heritability of is study one and h_2^2 is the study heritability from study two.

1.16. Sources of GWAS summary statistics data

GWAS summary statistics were obtained from different sources, including the GWAS summary statistics section of the GWAS catalog [40] and the and UK biobank [41]. GWAS summary statistics for PD and SKCM were directly requested to the 23andME and Genomel consortia. Only studies that tested more than 450000 variants and included at least 5000 individuals between cases and controls derived from European ancestry populations were selected. Precomputed LD scores of individuals with European ancestry derived from the 1000 Genomes project were used in the analyses.

1.17. Drug indications and LINCS L1000 analyses

1.17.1. Drug indications

Drug indications for each of the included disorders were obtained from MEDI-an [42]. MEDI-an is a medication indication repository that gathers information from multiple resources including, RxNorm, MedlinePlus, and SIDER2) constructed using combining ontology-based and natural language processing (NLP) techniques. We selected the MEDI high precision subset (MEDI-HPS), which contains 13400 unique indications regarding 2136 medications. The file (MEDI_HPS.csv) was downloaded from the site, and indications for all the studied CNS disorders and cancer types were selected by inspecting disease names and ICD codes. MEDI-HPS uses concept ids CUI IDs derived from the UMLS thesaurus to identify each available drug. Rxcui IDs were translated to Drugbank IDs using the restful web API from RxNav [43] through the use of the R packages httr [44]and rjson [45].

1.17.2. Differential expression profiles of cell lines treated with the indicated drugs

The differential gene expression profiles of cell lines treated with drugs indicated for the treatment of AD, PD, and the studied cancers were retrieved from LINCS L1000. LINCS L1000 contains an extensive collection of gene expression profiles generated using thousands of perturbagens (i.e., small molecules, ligands, micro-environments, CRISPR gene over-expression, and knockdown perturbations) and different cell lines, doses, and exposure times. Datasets from phases I (GSE92742) and II (GSE70138) of the project were downloaded from GEO. LINCS's perturbation IDs were mapped to Drugbank IDs through Inchi keys using the UnChem RESTful service [46]. All analyses were carried out using LINCS L1000 Level 5 data, which includes differential gene expression signatures computed by comparing three technical replicates of the same perturbation to appropriate controls. In Level 5 datasets, each perturbation (drug treatment) is represented by several differential gene expression signatures generated by treating different cell lines at different times and concentrations. We constructed an individual signature for each drug by combining all the available signatures generated using it, applying Stouffer's method.

For each signature, weights were computed as the average correlation to all other signatures. Then a consensus signature was created using the following formula applied to each gene.

$$Z \sim \frac{\sum_{i=1}^{k} w_i Z_i}{\sqrt{\sum_{i=1}^{k} w_i^2}}$$

Where w_i is the weight of the i-th signature and Z_i is the differential expression value of a particular gene in the i-th signature. Finally, Pearson's correlations between each disease signature and all drug consensus signatures were computed.

Table S1. Study characteristics for all included disorders. Platform.

Disease	Study ID	Year	Platform	Initial # Samples	Total # Samples	# Cases	# Controls	QC SMR	Included
**AD**	GSE1297	2004	1	31	31	22	9	5.12	Yes
**AD**	GSE5281	2006	2	161	23	10	13	4.75	Yes
**AD**	E_MEXP_2 280 [	2010	2	31	12	7	5	2.88	Yes
**AD**	GSE36980	2013	3	79	17	7	10	3.75	Yes
**AD**	GSE29378	2013	4	63	27	15	12	5.38	Yes
**AD**	GSE48350	2014	2	253	61	19	42	1.38	Yes
**AD**	GSE84422	2016	2	2004	55	44	11	4.75	Yes
**PD**	GSE7621	2007	2	25	25	16	9	3	Yes
**PD**	GSE8397	2008	1	94	38	24	14	1.88	Yes
**PD**	E_MEXP_1 416	2008	5	16	16	8	8	7.5	No
**PD**	GSE20141	2010	2	18	17	9	8	6.75	Yes
**PD**	GSE20163	2010	1	17	17	8	9	5.88	Yes
**PD**	GSE20164	2010	1	11	11	6	5	8	No
**PD**	GSE20295	2010	1	93	29	11	18	4.5	Yes
**PD**	GSE20159	2011	4	33	33	16	17	7.62	No
**PD**	GSE49036	2013	2	28	23	15	8	5.25	Yes
**PD**	GSE54282	2014	3	33	6	3	3	8.38	No
**PD**	GSE24378	NA	5	17	17	8	9	7.25	No
**ALL**	GSE13204	2009	2	3248	824	750	74	1.88	Yes
**ALL**	GSE26713	2011	2	124	124	117	7	2.38	Yes
**ALL**	GSE28497	2011	1	288	288	284	4	3.38	Yes
**ALL**	GSE46170	2013	2	38	38	32	6	3.75	Yes
**ALL**	GSE79533	2017	2	226	226	223	3	3.62	Yes
**AML**	GSE12662	2008	2	106	81	76	5	5.75	Yes
**AML**	GSE13204	2009	2	3248	574	501	73	2.88	Yes
**AML**	GSE30029	2011	4	121	121	90	31	3.38	Yes
**AML**	GSE34577	2011	4	89	75	57	18	3.88	Yes
**AML**	GSE48558	2013	3	170	21	18	3	5.88	Yes

**AML**	GSE67936	2015	6	168	168	150	18	4.88	Yes
**AML**	GSE68172	2015	2	77	15	10	5	8.25	No
**AML**	GSE63270	2016	2	104	104	42	62	3.62	Yes
**AML**	GSE76340	2016	4	166	18	15	3	6.5	Yes
**BLCA**	GSE7476	2007	2	12	12	9	3	1.88	Yes
**BLCA**	GSE13507	2008	7	256	84	75	9	3.5	Yes
**BLCA**	GSE52519	2013	8	12	8	5	3	3.38	Yes
**BLCA**	GSE38264	2014	3	51	38	28	10	2.25	Yes
**BLCA**	E_MTAB_1940	2015	2	86	86	82	4	4	Yes
**BRCA**	GSE7904	2007	2	62	47	40	7	6.12	Yes
**BRCA**	GSE10780	2009	2	185	185	42	143	3.62	Yes
**BRCA**	GSE10810	2009	2	58	58	31	27	2.38	Yes
**BRCA**	GSE29431	2011	2	66	54	44	10	4.38	Yes
**BRCA**	GSE31448	2011	2	357	356	352	4	3.88	Yes
**BRCA**	GSE42568	2013	2	121	114	98	16	3.12	Yes
**BRCA**	GSE54002	2014	2	433	433	417	16	6.12	Yes
**BRCA**	GSE45827	2016	2	155	149	140	9	6.38	Yes
**BRNCA**	GSE4290	2006	2	180	176	153	23	3.25	Yes
**BRNCA**	GSE9385	2008	9	55	55	49	6	4.5	Yes
**BRNCA**	GSE16011	2010	2	284	278	270	8	2.25	Yes
**BRNCA**	GSE21354	2010	2	18	18	14	4	9	No
**BRNCA**	GSE15824	2011	2	45	32	27	5	7.12	No
**BRNCA**	GSE42656	2013	4	73	55	44	11	6.5	Yes
**BRNCA**	GSE44971	2013	2	58	58	49	9	7.5	No
**BRNCA**	GSE50161	2013	2	130	128	115	13	4.75	Yes
**BRNCA**	GSE68848	2015	2	580	482	454	28	3.5	Yes
**BRNCA**	GSE74195	2015	2	51	44	39	5	6.62	Yes
**CERV**	GSE6791	2007	2	84	28	20	8	3.75	Yes
**CERV**	GSE39001	2013	3	79	24	19	5	3.08	Yes
**CERV**	GSE63514	2015	2	128	52	28	24	1.67	Yes
**CERV**	GSE67522	2015	6	42	35	15	20	2.5	Yes

**CHLCA**	GSE26566	2012	5	169	17	11	6	1.75	Yes
**CHLCA**	GSE32879	2012	3	37	23	16	7	1.75	Yes
**CHLCA**	GSE32225	2013	1	155	88	83	5	3.38	Yes
**CHLCA**	GSE22633	2014	7	63	24	20	4	3.12	Yes
**CLL**	GSE13204	2009	2	3248	522	448	74	2	Yes
**CLL**	GSE13987	2009	2	24	8	4	4	3.62	Yes
**CLL**	GSE26725	2011	2	17	17	12	5	5	Yes
**CLL**	GSE31048	2013	2	221	221	188	33	4.25	Yes
**CLL**	GSE51528	2015	3	2299	229	217	12	3.12	Yes
**CLL**	GSE67640	2017	6	24	24	15	9	3	Yes
**CML**	GSE13204	2009	2	3248	139	66	73	1.88	Yes
**CML**	GSE43754	2013	9	20	20	10	10	2	Yes
**CML**	GSE47927	2013	3	67	67	52	15	2.12	Yes
**CRCA**	GSE9348	2007	2	82	82	70	12	4.88	Yes
**CRCA**	GSE18105	2010	2	111	94	77	17	11.38	No
**CRCA**	GSE20916	2010	2	145	105	81	24	5.12	Yes
**CRCA**	GSE24550	2011	9	167	90	77	13	13.25	No
**CRCA**	GSE31279	2011	5	110	53	30	23	8.38	No
**CRCA**	GSE33113	2011	2	96	96	90	6	9.38	No
**CRCA**	GSE31737	2012	9	80	80	40	40	6.62	Yes
**CRCA**	GSE37182	2013	4	172	168	84	84	8.62	No
**CRCA**	GSE37364	2013	2	94	52	14	38	4.62	Yes
**CRCA**	GSE39582	2013	2	585	585	566	19	5.62	Yes
**CRCA**	GSE35834	2014	9	158	53	30	23	9	No
**CRCA**	GSE44076	2014	10	246	246	98	148	5.38	Yes
**CRCA**	GSE41657	2015	11	88	37	25	12	10.88	No
**CRCA**	GSE62932	2015	2	68	68	64	4	10.25	No
**CRCA**	GSE71187	2017	11	189	111	99	12	6.62	Yes
**DLBCL**	GSE12453	2008	2	67	36	11	25	1.88	Yes
**DLBCL**	GSE12195	2009	2	136	88	73	15	2	Yes
**DLBCL**	GSE56315	2015	2	122	122	89	33	2.12	Yes

**FLYMPH**	GSE12453	2008	2	67	30	5	25	4.62	Yes
**FLYMPH**	GSE12195	2009	2	136	53	38	15	2.25	Yes
**FLYMPH**	GSE14214	2011	12	13	12	9	3	4.62	Yes
**FLYMPH**	GSE48047	2014	13	55	26	18	8	4.12	Yes
**FLYMPH**	GSE55267	2014	2	69	69	63	6	2.62	Yes
**FLYMPH**	GSE65135	2015	2	28	24	14	10	2.75	Yes
**HANC**	GSE9844	2008	2	38	38	26	12	3.12	Yes
**HANC**	GSE23558	2011	12	32	28	23	5	4.62	Yes
**HANC**	GSE25099	2011	9	79	79	57	22	5	Yes
**HANC**	GSE30784	2011	2	229	212	167	45	2.12	Yes
**HANC**	GSE34105	2012	14	78	57	49	8	9	Yes
**HANC**	GSE29330	2014	2	18	18	13	5	4.25	Yes
**HANC**	GSE55550	2014	15	155	86	73	13	6.12	Yes
**HANC**	GSE59102	2014	11	42	32	23	9	4	Yes
**HANC**	GSE75538	2016	14	28	10	5	5	6.75	Yes
**KDNCA**	GSE11024	2008	2	79	22	10	12	6.38	Yes
**KDNCA**	GSE17895	2010	2	160	157	135	22	6.88	Yes
**KDNCA**	GSE36895	2012	2	76	52	29	23	2.62	Yes
**KDNCA**	GSE40435	2013	6	202	202	101	101	4.88	Yes
**KDNCA**	GSE47032	2013	9	40	20	10	10	5.12	Yes
**KDNCA**	GSE46699	2014	2	130	94	49	45	4.62	Yes
**KDNCA**	GSE76351	2015	16	24	24	12	12	7.62	No
**KDNCA**	GSE66272	2016	2	54	54	27	27	3.38	Yes
**KDNCA**	GSE68417	2016	3	49	49	35	14	6.38	Yes
**KDNCA**	GSE71963	2016	11	48	48	32	16	7.12	No
**LGCA**	GSE12236	2008	9	40	40	20	20	8.25	No
**LGCA**	GSE18842	2010	2	91	91	46	45	7	No
**LGCA**	GSE19188	2010	2	156	156	91	65	3.38	Yes
**LGCA**	GSE19804	2011	2	120	120	60	60	5.12	Yes
**LGCA**	GSE31210	2011	2	246	246	226	20	6.88	Yes
**LGCA**	GSE31552	2011	3	131	75	43	32	9.88	No

**LGCA**	GSE32863	2012	16	116	87	33	54	7.12	No
**LGCA**	GSE40275	2012	9	10	72	29	43	11.38	No
**LGCA**	GSE30219	2013	2	307	307	293	14	8.12	No
**LGCA**	GSE40791	2013	2	194	194	94	100	3.38	Yes
**LGCA**	GSE32665	2013	7	179	170	81	89	10.88	No
**LGCA**	GSE43458	2013	3	110	110	80	30	9.62	No
**LGCA**	GSE33532	2014	2	100	34	14	20	8.38	No
**LGCA**	GSE75037	2016	8	134	134	51	83	5.62	Yes
**LIVCA**	GSE6764	2007	2	75	75	65	10	7.12	No
**LIVCA**	GSE17967	2009	1	63	63	16	47	10.25	No
**LIVCA**	GSE12941	2010	9	20	20	10	10	8	No
**LIVCA**	GSE36376	2012	6	433	273	80	193	5.88	Yes
**LIVCA**	GSE50579	2013	13	80	34	26	8	9.12	No
**LIVCA**	GSE41804]	2013	2	40	40	20	20	5.12	Yes
**LIVCA**	GSE17548	2013	2	37	37	17	20	6.88	Yes
**LIVCA**	GSE54236	2014	11	161	133	55	78	6.38	Yes
**LIVCA**	GSE55092	2014	2	140	44	20	24	4.38	Yes
**LIVCA**	GSE62232	2014	2	91	86	81	5	6.12	Yes
**LIVCA**	GSE76427	2015	6	167	101	50	51	5.12	Yes
**LIVCA**	GSE64041	2016	3	44	125	60	65	3.62	Yes
**OVCA**	GSE10971	2008	2	37	37	13	24	3.62	Yes
**OVCA**	GSE14407	2009	2	24	19	7	12	4.12	Yes
**OVCA**	GSE36668	2012	2	12	12	8	4	3.38	Yes
**OVCA**	GSE40595	2014	2	77	75	61	14	2.38	Yes
**OVCA**	GSE69428	2015	2	29	17	8	9	3.5	Yes
**OVCA**	GSE66957	2015	17	69	62	57	5	4	Yes
**PACA**	GSE15471	2009	2	78	72	36	36	4.38	Yes
**PACA**	GSE19650	2010	2	22	19	12	7	6.62	Yes
**PACA**	GSE32676	2011	2	32	32	25	7	5.62	Yes
**PACA**	GSE41368	2013	3	12	12	6	6	3.75	Yes
**PACA**	GSE43795	2013	6	31	11	6	5	6.12	Yes

**PACA**	GSE55643	2014	11	53	21	18	3	6.75	Yes
**PACA**	GSE62165	2016	10	131	131	118	13	2.38	Yes
**PACA**	GSE62452	2016	3	130	130	69	61	2.88	Yes
**PACA**	GSE63111	2017	9	35	35	28	7	6.5	Yes
**PRCA**	GSE21034	2010	9	370	160	131	29	3.5	Yes
**PRCA**	GSE29079	2011	10	95	95	47	48	5.12	Yes
**PRCA**	GSE62872	2014	3	424	424	264	160	2.38	Yes
**PRCA**	GSE46602	2015	2	50	50	36	14	4.12	Yes
**PRCA**	GSE70768	2015	6	199	199	125	74	3.12	Yes
**PRCA**	GSE71016	2016	15	95	91	45	46	2.75	Yes
**SKCM**	GSE3189	2005	1	70	69	44	25	3.75	Yes
**SKCM**	GSE7553	2008	2	87	18	14	4	3.12	Yes
**SKCM**	GSE15605	2012	2	74	62	46	16	2.38	Yes
**SKCM**	GSE46517	2013	1	121	45	30	15	2	Yes
**SKCM**	GSE57715	2014	18	297	292	275	17	3.75	Yes
**STCA**	GSE13911	2008	2	69	69	38	31	3.88	Yes
**STCA**	GSE13195	2009	9	100	50	25	25	4.5	Yes
**STCA**	GSE27342	2011	9	160	146	73	73	5	Yes
**STCA**	GSE29998	2012	4	99	99	50	49	4.12	Yes
**STCA**	GSE30727	2014	9	60	60	30	30	4.5	Yes
**STCA**	GSE51575	2014	13	52	36	15	21	6.25	Yes
**STCA**	GSE26899	2016	4	108	52	41	11	5.88	Yes
**STCA**	GSE79973	2016	2	20	20	10	10	4.88	Yes
**STCA**	GSE54129	2017	2	132	132	111	21	6	Yes
**THCA**	GSE3467	2005	2	18	18	9	9	2.75	Yes
**THCA**	GSE53157	2013	2	27	27	24	3	4.5	Yes
**THCA**	GSE35570	2015	2	116	116	65	51	1.75	Yes
**THCA**	GSE60542	2015	2	92	59	29	30	2.62	Yes
**THCA**	GSE65144	2015	2	25	25	12	13	3.38	Yes

Codes: 1- hgu133a, 2- hgu133plus2, 3- Human Gene 1.0 ST, 4- Illumina HumanHT-12 V3.0, 5- Illumina humanRef-8 v2.0, 5- Affymetrix Human X3P Array, 6- Illumina HumanHT-12 V4.0, 7-Illumina human-6 v2.0 expression beadchip, 8- Illumina HumanWG-6 v3.0, 9- Affymetrix Human Exon 1.0 ST Array, 10- Affymetrix Human Genome U219 Array, 11- Agilent-014850 Whole Human Genome Microarray 4x44K G4112F, 12- Sentrix HumanRef-8 Expression Bead-Chip, 13-Agilent-028004 SurePrint G3 Human GE 8x60K Microarray, 14-Illumina HumanHT-12 WG-DASL V4.0, 15-

Agilent-039494 SurePrint G3 Human GE v2 8x60K, 16-Affymetrix Human Gene 1.1 ST Array, 17-Rosetta/Merck Human RSTA Custom Affymetrix 2.0 microarray, 18-Illumina HumanRef-8 WG-DASL v3.0.

Table S2. Differential gene expression analysis results of TCGA datasets.

TCGA Dataset	TCGA Dataset Description	Correspondence to Array Analysis Abbreviations	N	Nº Cases	Nº Controls	Nº Tested Genes	Nº DEGs	Nº Up	Nº Down
**BLCA**	Bladder Urothelial Carcinoma	BLCA	433	414	19	16251	9043	6045	2998
**BRCA**	Breast Invasive Carcinoma	BRCA	1215	1102	113	16251	13665	9026	4639
**GBM**	Glioblastoma Multiforme	BRNCA	161	156	5	16251	9187	5639	3548
**CESC**	Cervical Squamous Cell Carcinoma and Endocervical Adenocarcinoma	CERV	307	304	3	16251	4418	2407	2011
**CHOL**	Cholangiocarcinoma	CHOL	45	36	9	16251	8989	6055	2934
**COAD**	Colon Adenocarcinoma	CRCA	519	478	41	16250	12705	7938	4767
**READ**	Rectum Adenocarcinoma	CRCA	176	166	10	16251	9481	5934	3547
**HNSC**	Head and Neck Squamous Cell Carcinoma	HANC	544	500	44	16251	11334	6668	4666
**KICH**	Kidney Chromophobe	KDNCA	89	65	24	16251	11665	7257	4408
**KIRC**	Kidney Renal Clear Cell Carcinoma	KDNCA	610	538	72	16251	13291	7999	5292
**LUAD**	Lung Adenocarcinoma	LGCA	592	533	59	16251	12834	8590	4244

**LUSC**	Lung Squamous Cell Carcinoma	LGCA	551	502	49	16251	13367	8417	4950
**LIHC**	Liver Hepatocellular Carcinoma	LIVCA	421	371	50	16251	11597	8490	3107
**PAAD**	Pancreatic Adenocarcinoma	PACA	181	177	4	16251	842	94	748
**PRAD**	Prostate Adenocarcinoma	PRCA	550	498	52	16229	11644	7023	4621
**STAD**	Stomach Adenocarcinoma	STCA	407	375	32	16251	10306	5714	4592
**THCA**	Thyroid Carcinoma	THCA	560	502	58	16251	11638	6040	5598

Table S3. Jointly deregulated pathways in AD and BRNCA.

	Up_BRNCA	Down_BRNCA
Up_AD	(1) (H)K INFLAMMATORY RESPONSE, (2) (H)K EPITHELIAL MESENCHYMAL TRANSITION, (3) (H)K ALLOGRAFT REJECTION, (4) (H)K IL6 JAK STAT3 SIGNALING, (5) (H)K COAGULATION, (6) (H)K TNFA SIGNALING VIA NFKB, (7) (H)K IL2 STAT5 SIGNALING, (8) (K) CYTOKINE CYTOKINE RECEPTOR INTERACTION, (9) NABA CORE MATRISOME, (10) NABA MATRISOME, (11) (R) EXTRACELLULAR MATRIX ORGANIZATION, (12) (R) INTERLEUKIN 4 AND INTERLEUKIN 13 SIGNALING, (13) NABA ECM GLYCOPROTEINS, (14) (R) ELASTIC FIBRE FORMATION, (15) (R) COLLAGEN FORMATION, (16) (R) MOLECULES ASSOCIATED WITH ELASTIC FIBRES, (17) (K) ECM RECEPTOR INTERACTION, (18) (R) ECM PROTEOGLYCANS, (19) (R) ASSEMBLY OF COLLAGEN FIBRILS AND OTHER MULTIMERIC STRUCTURES, (20) (K) PATHWAYS IN CANCER, (21) (R) NON INTEGRIN MEMBRANE ECM INTERACTIONS, (22) (PID) INTEGRIN1 PATHWAY, (23) (R) INTEGRIN CELL SURFACE INTERACTIONS, (24) (R) DEGRADATION OF THE EXTRACELLULAR MATRIX, (25) (R) COLLAGEN DEGRADATION, (26) (R) SIGNALING BY INTERLEUKINS, (27) (K) GRAFT VERSUS HOST DISEASE, (28) (K) AUTOIMMUNE THYROID DISEASE, (29) (R) INTERLEUKIN 10 SIGNALING, (30) (GO) ADAPTIVE IMMUNE RESPONSE, (31) (GO) ANATOMICAL STRUCTURE FORMATION INVOLVED IN MORPHOGENESIS, (32) (GO) ANIMAL ORGAN MORPHOGENESIS, (33) (GO) BIOLOGICAL ADHESION, (34) (GO) BLOOD VESSEL MORPHOGENESIS, (35) (GO) CARDIOVASCULAR SYSTEM DEVELOPMENT, (36) (GO) CELL ACTIVATION, (37) (GO) CELL SURFACE, (38) (GO) CHROMATIN, (39) (GO) CHROMOSOME, (40) (GO) CIRCULATORY SYSTEM DEVELOPMENT, (41) (GO) CIS REGULATORY REGION BINDING, (42) (GO) COLLAGEN CONTAINING EXTRACELLULAR MATRIX, (43) (GO) CONNECTIVE TISSUE DEVELOPMENT, (44) (GO) CYTOKINE MEDIATED SIGNALING PATHWAY, (45) (GO) CYTOKINE PRODUCTION, (46) (GO) DEFENSE RESPONSE, (47) (GO) DOUBLE STRANDED DNA BINDING, (48) (GO) EMBRYO DEVELOPMENT, (49) (GO) EPITHELIUM DEVELOPMENT, (50) (GO) EXTRACELLULAR MATRIX, (51) (GO) EXTRACELLULAR MATRIX STRUCTURAL CONSTITUENT, (52) (GO) EXTRACELLULAR STRUCTURE ORGANIZATION, (53) (GO) IMMUNE SYSTEM DEVELOPMENT, (54) (GO) INFLAMMATORY RESPONSE, (55) (GO) LEUKOCYTE CELL CELL ADHESION, (56) (GO) LEUKOCYTE MIGRATION, (57) (GO) LEUKOCYTE PROLIFERATION, (58) (GO) LYMPHOCYTE ACTIVATION, (59) (GO) NEGATIVE REGULATION OF BIOSYNTHETIC PROCESS, (60) (GO) NEGATIVE REGULATION OF IMMUNE SYSTEM PROCESS, (61) (GO) NEGATIVE REGULATION OF NUCLEOBASE CONTAINING COMPOUND METABOLIC PROCESS, (62) (GO) NEGATIVE REGULATION OF RNA BIOSYNTHETIC PROCESS, (63) (GO) NEGATIVE REGULATION OF TRANSCRIPTION BY RNA POLYMERASE II, (64) (GO) NUCLEAR CHROMOSOME, (65) (GO) POSITIVE REGULATION OF CELLULAR BIOSYNTHETIC PROCESS, (66) (GO)	(1) (H)K KRAS SIGNALING DN, (2) (GO) INTRINSIC COMPONENT OF PLASMA MEMBRANE

	POSITIVE REGULATION OF CELL POPULATION PROLIFERATION, (67) (GO) POSITIVE REGULATION OF IMMUNE SYSTEM PROCESS, (68) (GO) POSITIVE REGULATION OF NUCLEOBASE CONTAINING COMPOUND METABOLIC PROCESS, (69) (GO) POSITIVE REGULATION OF RNA METABOLIC PROCESS, (70) (GO) POSITIVE REGULATION OF TRANSCRIPTION BY RNA POLYMERASE II, (71) (GO) POSITIVE REGULATION OF VASCULATURE DEVELOPMENT, (72) (GO) REGULATION OF CELL ACTIVATION, (73) (GO) REGULATION OF CELL ADHESION, (74) (GO) REGULATION OF CELL POPULATION PROLIFERATION, (75) (GO) REGULATION OF IMMUNE SYSTEM PROCESS, (76) (GO) REGULATION OF LEUKOCYTE PROLIFERATION, (77) (GO) REGULATION OF LYMPHOCYTE ACTIVATION, (78) (GO) REGULATION OF VASCULATURE DEVELOPMENT, (79) (GO) RESPONSE TO BACTERIUM, (80) (GO) RESPONSE TO BIOTIC STIMULUS, (81) (GO) RESPONSE TO MOLECULE OF BACTERIAL ORIGIN, (82) (GO) RESPONSE TO WOUNDING, (83) (GO) SEQUENCE SPECIFIC DNA BINDING, (84) (GO) SEQUENCE SPECIFIC DOUBLE STRANDED DNA BINDING, (85) (GO) SKELETAL SYSTEM DEVELOPMENT, (86) (GO) SKELETAL SYSTEM MORPHOGENESIS, (87) (GO) TRANSCRIPTION REGULATOR ACTIVITY, (88) (GO) TUBE DEVELOPMENT, (89) (GO) TUBE MORPHOGENESIS, (90) (GO) T CELL ACTIVATION, (91) (GO) UROGENITAL SYSTEM DEVELOPMENT, (92) (GO) WOUND HEALING, (93) (GO) RENAL SYSTEM DEVELOPMENT, (94) (GO) CARTILAGE DEVELOPMENT, (95) (GO) T CELL DIFFERENTIATION, (96) (GO) POSITIVE REGULATION OF CELL ACTIVATION, (97) (GO) RESPONSE TO CYTOKINE, (98) (GO) REGULATION OF T CELL ACTIVATION, (99) (GO) REGULATION OF CELL CELL ADHESION, (100) (GO) POSITIVE REGULATION OF CELL ADHESION, (101) (GO) T CELL PROLIFERATION, (102) (GO) EMBRYO DEVELOPMENT ENDING IN BIRTH OR EGG HATCHING, (103) (GO) DEFENSE RESPONSE TO OTHER ORGANISM, (104) (GO) REGULATION OF IMMUNE RESPONSE, (105) (GO) REGULATION OF IMMUNE EFFECTOR PROCESS, (106) (GO) GLAND DEVELOPMENT, (107) (GO) TISSUE MORPHOGENESIS, (108) (GO) BRANCHING MORPHOGENESIS OF AN EPITHELIAL TUBE, (109) (GO) TUMOR NECROSIS FACTOR SUPERFAMILY CYTOKINE PRODUCTION, (110) (GO) CELLULAR RESPONSE TO BIOTIC STIMULUS, (111) (GO) POSITIVE REGULATION OF LEUKOCYTE CELL CELL ADHESION, (112) (GO) ADAPTIVE IMMUNE RESPONSE BASED ON SOMATIC RECOMBINATION OF IMMUNE RECEPTORS BUILT FROM IMMUNOGLOBULIN SUPERFAMILY DOMAINS, (113) (GO) POSITIVE REGULATION OF CELL CELL ADHESION, (114) (GO) INTERLEUKIN 6 PRODUCTION, (115) (GO) FORMATION OF PRIMARY GERM LAYER, (116) (GO) GROWTH FACTOR BINDING, (117) (GO) EPITHELIAL CELL PROLIFERATION, (118) (GO) POSITIVE REGULATION OF LOCOMOTION, (119) (GO) TRANSCRIPTION FACTOR COMPLEX, (120) (GO) PATTERN RECOGNITION RECEPTOR SIGNALING PATHWAY, (121) (GO) POSITIVE REGULATION OF LEUKOCYTE PROLIFERATION, (122) (GO) OSSIFICATION, (123) (GO) INNATE IMMUNE RESPONSE, (124) (GO) COLLAGEN TRIMER, (125) (GO) COLLAGEN	

	FIBRIL ORGANIZATION, (126) (GO) NEPHRON DEVELOPMENT, (127) (GO) POSITIVE REGULATION OF IMMUNE RESPONSE, (128) (GO) COLLAGEN METABOLIC PROCESS, (129) (GO) POSITIVE REGULATION OF CYTOKINE PRODUCTION, (130) (GO) CELL ADHESION MEDIATED BY INTEGRIN, (131) (GO) IMMUNE EFFECTOR PROCESS, (132) (GO) TOLL LIKE RECEPTOR SIGNALING PATHWAY, (133) (GO) POSITIVE REGULATION OF T CELL PROLIFERATION, (134) (GO) MORPHOGENESIS OF AN EPITHELIUM, (135) (GO) REGULATION OF RESPONSE TO EXTERNAL STIMULUS, (136) (GO) BASEMENT MEMBRANE, (137) (GO) POSITIVE REGULATION OF HEMOPOIESIS, (138) (GO) REGULATION OF HEMOPOIESIS, (139) (GO) POSITIVE REGULATION OF EPITHELIAL CELL PROLIFERATION, (140) (GO) I KAPPAB KINASE NF KAPPAB SIGNALING, (141) (GO) POSITIVE REGULATION OF TUMOR NECROSIS FACTOR SUPERFAMILY CYTOKINE PRODUCTION, (142) (GO) CELLULAR RESPONSE TO VASCULAR ENDOTHELIAL GROWTH FACTOR STIMULUS, (143) (GO) CHROMATIN BINDING, (144) (GO) ENDOPLASMIC RETICULUM LUMEN, (145) (GO) B CELL MEDIATED IMMUNITY, (146) (GO) REGULATION OF ADAPTIVE IMMUNE RESPONSE, (147) (GO) ACTIVATION OF IMMUNE RESPONSE, (148) (GO) MESENCHYME MORPHOGENESIS, (149) (GO) REGULATION OF DEFENSE RESPONSE	
Down_AD	(1) (H)K MYC TARGETS V1, (2) (H)K MTORC1 SIGNALING, (3) (H)K DNA REPAIR, (4) (H)K UNFOLDED PROTEIN RESPONSE, (5) (R) APC C CDH1 MEDIATED DEGRADATION OF CDC20 AND OTHER APC C CDH1 TARGETED PROTEINS IN LATE MITOSIS EARLY G1, (6) (R) ASSEMBLY OF THE PRE REPLICATIVE COMPLEX, (7) (R) AUF1 HNRNP D0 BINDS AND DESTABILIZES MRNA, (8) (R) CELL CYCLE CHECKPOINTS, (9) (R) DNA REPLICATION, (10) (R) HIV INFECTION, (11) (R) METABOLISM OF RNA, (12) (R) MITOTIC METAPHASE AND ANAPHASE, (13) (R) ORC1 REMOVAL FROM CHROMATIN, (14) (R) SEPARATION OF SISTER CHROMATIDS, (15) (R) SWITCHING OF ORIGINS TO A POST REPLICATIVE STATE, (16) (R) TRANSLATION, (17) (R) POST TRANSLATIONAL PROTEIN MODIFICATION, (18) (R) APC C MEDIATED DEGRADATION OF CELL CYCLE PROTEINS, (19) (R) DNA REPLICATION PRE INITIATION, (20) (R) STABILIZATION OF P53, (21) (R) REGULATION OF RUNX3 EXPRESSION AND ACTIVITY, (22) (R) S PHASE, (23) (R) REGULATION OF EXPRESSION OF SLITS AND ROBOS, (24) (R) G2 M CHECKPOINTS, (25) (R) NUCLEOTIDE EXCISION REPAIR, (26) (R) CYCLIN A CDK2 ASSOCIATED EVENTS AT S PHASE ENTRY, (27) (R) PROCESSING OF CAPPED INTRON CONTAINING PRE MRNA, (28) (R) G1 S DNA DAMAGE CHECKPOINTS, (29) (R) CELL CYCLE, (30) (R) M PHASE, (31) (R) TRANSCRIPTION COUPLED NUCLEOTIDE EXCISION REPAIR TC NER, (32) (R) HIV LIFE CYCLE, (33) (R) SIGNALING BY NOTCH4, (34) (R) CELL CYCLE MITOTIC, (35) (R) CELLULAR RESPONSES TO EXTERNAL STIMULI, (36) (R) MRNA SPLICING, (37) (R) GLOBAL GENOME NUCLEOTIDE EXCISION REPAIR GG NER, (38) (R) MITOTIC G1 PHASE AND G1 S TRANSITION, (39) (R) TRNA PROCESSING, (40) (R) MRNA SPLICING MINOR PATHWAY, (41) (R) RRNA PROCESSING, (42) (R)	(1) (R) MEMBRANE TRAFFICKING, (2) (R) NEURONAL SYSTEM, (3) (R) TRANSMISSION ACROSS CHEMICAL SYNAPSES, (4) (R) VESICLE MEDIATED TRANSPORT, (5) (K) CARDIAC MUSCLE CONTRACTION, (6) (R) CLATHRIN MEDIATED ENDOCYTOSIS, (7) (R) NEUROTRANSMITTER RECEPTORS AND POSTSYNAPTIC SIGNAL TRANSMISSION, (8) (R) ACTIVATION OF NMDA RECEPTORS AND POSTSYNAPTIC EVENTS, (9) (R) CAR(GO) RECOGNITION FOR CLATHRIN MEDIATED ENDOCYTOSIS, (10) (R) PROTEIN PROTEIN INTERACTIONS AT SYNAPSES, (11) (GO) AXON, (12) (GO) EXOCYTIC VESICLE, (13) (GO) GLUTAMATERGIC SYNAPSE, (14) (GO) MEMBRANE PROTEIN COMPLEX, (15) (GO) NEURON PROJECTION, (16) (GO) NEURON TO NEURON SYNAPSE, (17) (GO) POSTSYNAPSE, (18) (GO) PRESYNAPSE, (19) (GO) SOMATODENDRITIC COMPARTMENT, (20) (GO) SYNAPSE, (21) (GO) SYNAPTIC MEMBRANE, (22) (GO) SYNAPTIC SIGNALING, (23) (GO) TRANSPORT VESICLE, (24) (GO) VESICLE MEDIATED TRANSPORT IN SYNAPSE, (25) (GO) TRANSPORT VESICLE MEMBRANE, (26) (GO) DENDRITIC TREE, (27) (GO) SYNAPTIC VESICLE MEMBRANE, (28) (GO) MEMBRANE ORGANIZATION, (29) (GO) REGULATION OF TRANS SYNAPTIC SIGNALING, (30) (GO)

	ANTIGEN PROCESSING CROSS PRESENTATION, (43) (R) VIRAL MESSENGER RNA SYNTHESIS, (44) (R) RRNA MODIFICATION IN THE NUCLEUS AND CYTOSOL, (45) (R) DUAL INCISION IN TC NER, (46) (GO) AMIDE BIOSYNTHETIC PROCESS, (47) (GO) ANAPHASE PROMOTING COMPLEX DEPENDENT CATABOLIC PROCESS, (48) (GO) CATALYTIC ACTIVITY ACTING ON RNA, (49) (GO) CATALYTIC COMPLEX, (50) (GO) CELLULAR AMIDE METABOLIC PROCESS, (51) (GO) CELLULAR MACROMOLECULE CATABOLIC PROCESS, (52) (GO) MITOCHONDRIAL GENE EXPRESSION, (53) (GO) MITOCHONDRIAL MATRIX, (54) (GO) NCRNA METABOLIC PROCESS, (55) (GO) NCRNA PROCESSING, (56) (GO) ORGANONITROGEN COMPOUND BIOSYNTHETIC PROCESS, (57) (GO) PEPTIDE BIOSYNTHETIC PROCESS, (58) (GO) PEPTIDE METABOLIC PROCESS, (59) (GO) RIBONUCLEOPROTEIN COMPLEX, (60) (GO) RIBONUCLEOPROTEIN COMPLEX BIOGENESIS, (61) (GO) RIBOSOME, (62) (GO) RIBOSOME BIOGENESIS, (63) (GO) RNA BINDING, (64) (GO) RNA PROCESSING, (65) (GO) TRNA METABOLIC PROCESS, (66) (GO) RIBOSOMAL SUBUNIT, (67) (GO) REGULATION OF CELL CYCLE G2 M PHASE TRANSITION, (68) (GO) CATALYTIC ACTIVITY ACTING ON A TRNA, (69) (GO) NUCLEOLUS, (70) (GO) NEGATIVE REGULATION OF CELL CYCLE G2 M PHASE TRANSITION, (71) (GO) PROTEIN LOCALIZATION TO ORGANELLE, (72) (GO) POSTTRANSCRIPTIONAL REGULATION OF GENE EXPRESSION, (73) (GO) RRNA METABOLIC PROCESS, (74) (GO) STRUCTURAL CONSTITUENT OF RIBOSOME, (75) (GO) MRNA METABOLIC PROCESS, (76) (GO) RIBONUCLEOPROTEIN COMPLEX SUBUNIT ORGANIZATION, (77) (GO) TRANSCRIPTION COUPLED NUCLEOTIDE EXCISION REPAIR, (78) (GO) MACROMOLECULE CATABOLIC PROCESS, (79) (GO) TRNA PROCESSING, (80) (GO) RNA SPLICING VIA TRANSESTERIFICATION REACTIONS, (81) (GO) REGULATION OF MRNA CATABOLIC PROCESS, (82) (GO) ANTIGEN PROCESSING AND PRESENTATION OF EXOGENOUS PEPTIDE ANTIGEN VIA MHC CLASS I, (83) (GO) RIBONUCLEOPROTEIN COMPLEX BINDING, (84) (GO) NUCLEOTIDE EXCISION REPAIR, (85) (GO) RNA CATABOLIC PROCESS, (86) (GO) ATPASE ACTIVITY, (87) (GO) CYTOPLASMIC TRANSLATION, (88) (GO) MRNA PROCESSING, (89) (GO) SPLICEOSOMAL TRI SNRNP COMPLEX, (90) (GO) RNA LOCALIZATION, (91) (GO) RNA SPLICING, (92) (GO) PRERIBOSOME, (93) (GO) SMALL RIBOSOMAL SUBUNIT	REGULATION OF SYNAPTIC PLASTICITY, (31) (GO) CELL BODY, (32) (GO) REGULATION OF SYNAPTIC VESICLE CYCLE, (33) (GO) DENDRITIC SPINE DEVELOPMENT, (34) (GO) POSTSYNAPTIC MEMBRANE, (35) (GO) COGNITION, (36) (GO) MONOVALENT INORGANIC CATION TRANSPORT, (37) (GO) LEARNING, (38) (GO) MICROTUBULE, (39) (GO) SYNAPTIC VESICLE EXOCYTOSIS, (40) (GO) DENDRITIC SPINE MORPHOGENESIS, (41) (GO) REGULATION OF DENDRITIC SPINE DEVELOPMENT, (42) (GO) NEURON PROJECTION CYTOPLASM, (43) (GO) BEHAVIOR, (44) (GO) MONOVALENT INORGANIC CATION TRANSMEMBRANE TRANSPORTER ACTIVITY, (45) (GO) DISTAL AXON, (46) (GO) NEUROTRANSMITTER SECRETION, (47) (GO) PRESYNAPTIC MEMBRANE, (48) (GO) VESICLE LOCALIZATION, (49) (GO) CATION TRANSMEMBRANE TRANSPORT, (50) (GO) GABA ERGIC SYNAPSE, (51) (GO) SYNAPTIC VESICLE RECYCLING, (52) (GO) AXON CYTOPLASM, (53) (GO) POSTSYNAPTIC SPECIALIZATION MEMBRANE, (54) (GO) POSTSYNAPTIC DENSITY MEMBRANE, (55) (GO) ORGANELLE TRANSPORT ALONG MICROTUBULE, (56) (GO) PROTEIN LOCALIZATION TO MEMBRANE, (57) (GO) NEUROTRANSMITTER TRANSPORT, (58) (GO) MICROTUBULE BASED TRANSPORT, (59) (GO) REGULATION OF NEURONAL SYNAPTIC PLASTICITY, (60) (GO) PRESYNAPTIC ACTIVE ZONE, (61) (GO) REGULATION OF SYNAPTIC VESICLE EXOCYTOSIS, (62) (GO) TRANSMEMBRANE TRANSPORT, (63) (GO) INORGANIC ION TRANSMEMBRANE TRANSPORT, (64) (GO) SNARE BINDING, (65) (GO) CYTOSKELETON DEPENDENT INTRACELLULAR TRANSPORT, (66) (GO) REGULATION OF DENDRITIC SPINE MORPHOGENESIS, (67) (GO) DENDRITE DEVELOPMENT, (68) (GO) PROTEIN LOCALIZATION TO SYNAPSE, (69) (GO) SITE OF POLARIZED GROWTH, (70) (GO) SYNAPSE ORGANIZATION, (71) (GO) ION TRANSMEMBRANE TRANSPORT, (72) (GO) SYNAPTIC VESICLE ENDOCYTOSIS, (73) (GO) REGULATION OF DENDRITE DEVELOPMENT, (74) (GO) SCHAFFER COLLATERAL CA1 SYNAPSE, (75) (GO) MEMORY, (76) (GO) GLUTAMATE RECEPTOR SIGNALING PATHWAY, (77) (GO) REGULATION OF NEURON PROJECTION DEVELOPMENT, (78) (GO) AXO DENDRITIC TRANSPORT, (79) (GO) POSTSYNAPSE

		ORGANIZATION, (80) (GO) REGULATION OF SYNAPTIC VESICLE RECYCLING, (81) (GO) NEURON PROJECTION ORGANIZATION, (82) (GO) REGULATION OF NEUROTRANSMITTER TRANSPORT, (83) (GO) DENDRITE MORPHOGENESIS, (84) (GO) TUBULIN BINDING, (85) (GO) INTRINSIC COMPONENT OF SYNAPTIC VESICLE MEMBRANE, (86) (GO) REGULATION OF LONG TERM NEURONAL SYNAPTIC PLASTICITY, (87) (GO) REGULATION OF POSTSYNAPTIC MEMBRANE NEUROTRANSMITTER RECEPTOR LEVELS, (88) (GO) POSITIVE REGULATION OF DENDRITIC SPINE DEVELOPMENT, (89) (GO) REGULATION OF NEUROTRANSMITTER SECRETION

Table S4. Jointly deregulated pathways in AD and THCA.

	Up_THCA	Down_THCA
Up_AD	(1) (H)K INFLAMMATORY RESPONSE, (2) (H)K EPITHELIAL MESENCHYMAL TRANSITION, (3) (H)K ALLOGRAFT REJECTION, (4) (H)K IL6 JAK STAT3 SIGNALING, (5) (H)K COAGULATION, (6) (H)K TNFA SIGNALING VIA NFKB, (7) (H)K IL2 STAT5 SIGNALING, (8) (K) CYTOKINE CYTOKINE RECEPTOR INTERACTION, (9) NABA MATRISOME, (10) NABA MATRISOME ASSOCIATED, (11) NABA SECRETED FACTORS, (12) (R) EXTRACELLULAR MATRIX ORGANIZATION, (13) (R) IMMUNOREGULATORY INTERACTIONS BETWEEN A LYMPHOID AND A NON LYMPHOID CELL, (14) (R) INTERLEUKIN 4 AND INTERLEUKIN 13 SIGNALING, (15) NABA ECM REGULATORS, (16) (R) CLASS A 1 RHODOPSIN LIKE RECEPTORS, (17) (R) CELL SURFACE INTERACTIONS AT THE VASCULAR WALL, (18) (R) COLLAGEN FORMATION, (19) (R) KERATINIZATION, (20) (K) ECM RECEPTOR INTERACTION, (21) (R) ECM PROTEOGLYCANS, (22) (R) ASSEMBLY OF COLLAGEN FIBRILS AND OTHER MULTIMERIC STRUCTURES, (23) (K) CELL ADHESION MOLECULES CAMS, (24) (R) GPCR LIGAND BINDING, (25) (R) NON INTEGRIN MEMBRANE ECM INTERACTIONS, (26) (PID) INTEGRIN1 PATHWAY, (27) (R) INTEGRIN CELL SURFACE INTERACTIONS, (28) (R) DEGRADATION OF THE EXTRACELLULAR MATRIX, (29) (R) COLLAGEN DEGRADATION, (30) (R) SIGNALING BY INTERLEUKINS, (31) (R) INTERLEUKIN 10 SIGNALING, (32) (R) SYNDECAN INTERACTIONS, (33) (GO) ADAPTIVE IMMUNE RESPONSE, (34) (GO) ANATOMICAL STRUCTURE FORMATION INVOLVED IN MORPHOGENESIS, (35) (GO) ANIMAL ORGAN MORPHOGENESIS, (36) (GO) BIOLOGICAL ADHESION, (37) (GO) BLOOD VESSEL MORPHOGENESIS, (38) (GO) CARDIOVASCULAR SYSTEM DEVELOPMENT, (39) (GO) CELL ACTIVATION, (40) (GO) CELL CELL ADHESION, (41) (GO) CELL MOTILITY, (42) (GO) CELL SURFACE, (43) (GO) CIRCULATORY SYSTEM DEVELOPMENT, (44) (GO) COLLAGEN CONTAINING EXTRACELLULAR MATRIX, (45) (GO) CYTOKINE MEDIATED SIGNALING PATHWAY, (46) (GO) CYTOKINE PRODUCTION, (47) (GO) DEFENSE RESPONSE, (48) (GO) EMBRYO DEVELOPMENT, (49) (GO) E(PID)ERMIS DEVELOPMENT, (50) (GO) EPITHELIUM DEVELOPMENT, (51) (GO) EXTRACELLULAR MATRIX, (52) (GO) EXTRACELLULAR STRUCTURE ORGANIZATION, (53) (GO) INFLAMMATORY RESPONSE, (54) (GO) INTRINSIC COMPONENT OF PLASMA MEMBRANE, (55) (GO) LEUKOCYTE MIGRATION, (56) (GO) LOCOMOTION, (57) (GO) MOLECULAR TRANSDUCER ACTIVITY, (58) (GO) NEGATIVE REGULATION OF DEVELOPMENTAL PROCESS, (59) (GO) NEGATIVE REGULATION OF MULTICELLULAR ORGANISMAL PROCESS, (60) (GO) POSITIVE REGULATION OF CELL POPULATION PROLIFERATION, (61) (GO) POSITIVE REGULATION OF DEVELOPMENTAL PROCESS, (62) (GO) POSITIVE REGULATION OF IMMUNE SYSTEM PROCESS, (63) (GO) REGULATION OF CELL ACTIVATION, (64) (GO) REGULATION OF CELL ADHESION, (65) (GO) REGULATION OF CELL POPULATION PROLIFERATION, (66) (GO) REGULATION OF IMMUNE SYSTEM PROCESS, (67) (GO) REGULATION OF VASCULATURE DEVELOPMENT, (68) (GO) RESPONSE TO BACTERIUM, (69) (GO) RESPONSE TO BIOTIC STIMULUS, (70) (GO) RESPONSE TO WOUNDING, (71) (GO) SKIN DEVELOPMENT, (72) (GO) TUBE DEVELOPMENT, (73) (GO)	(1) (GO) DNA BINDING TRANSCRIPTION FACTOR ACTIVITY, (2) (GO) TRANSCRIPTION REGULATOR ACTIVITY

	TUBE MORPHOGENESIS, (74) (GO) WOUND HEALING, (75) (GO) POSITIVE REGULATION OF MULTICELLULAR ORGANISMAL PROCESS, (76) (GO) E(PID)ERMAL CELL DIFFERENTIATION, (77) (GO) RESPONSE TO CYTOKINE, (78) (GO) EMBRYO DEVELOPMENT ENDING IN BIRTH OR EGG HATCHING, (79) (GO) DEFENSE RESPONSE TO OTHER ORGANISM, (80) (GO) REGULATION OF IMMUNE RESPONSE, (81) (GO) REGULATION OF CELL DIFFERENTIATION, (82) (GO) POSITIVE REGULATION OF INTRACELLULAR SIGNAL TRANSDUCTION, (83) (GO) KERATINOCYTE DIFFERENTIATION, (84) (GO) TISSUE MORPHOGENESIS, (85) (GO) CYTOKINE ACTIVITY, (86) (GO) TISSUE REMODELING, (87) (GO) DEFENSE RESPONSE TO BACTERIUM, (88) (GO) REGULATION OF CELLULAR COMPONENT MOVEMENT, (89) (GO) POSITIVE REGULATION OF LOCOMOTION, (90) (GO) CELL SUBSTRATE ADHESION, (91) (GO) SIGNALING RECEPTOR BINDING, (92) (GO) RECEPTOR REGULATOR ACTIVITY, (93) (GO) INNATE IMMUNE RESPONSE, (94) (GO) COLLAGEN FIBRIL ORGANIZATION, (95) (GO) CYTOKINE SECRETION, (96) (GO) CELL CHEMOTAXIS, (97) (GO) POSITIVE REGULATION OF MAPK CASCADE, (98) (GO) POSITIVE REGULATION OF CELL DIFFERENTIATION, (99) (GO) REGULATION OF BODY FLUID LEVELS, (100) (GO) POSITIVE REGULATION OF IMMUNE RESPONSE, (101) (GO) COLLAGEN METABOLIC PROCESS, (102) (GO) POSITIVE REGULATION OF CYTOKINE PRODUCTION, (103) (GO) POSITIVE REGULATION OF PHOSPHORUS METABOLIC PROCESS, (104) (GO) POSITIVE REGULATION OF SIGNALING, (105) (GO) REGULATION OF INFLAMMATORY RESPONSE, (106) (GO) IMMUNE EFFECTOR PROCESS, (107) (GO) MORPHOGENESIS OF AN EPITHELIUM, (108) (GO) REGULATION OF RESPONSE TO EXTERNAL STIMULUS, (109) (GO) GRANULOCYTE MIGRATION, (110) (GO) KERATINIZATION, (111) (GO) ANCHORING JUNCTION, (112) (GO) TAXIS, (113) (GO) POSITIVE REGULATION OF PROTEIN MODIFICATION PROCESS, (114) (GO) I KAPPAB KINASE NF KAPPAB SIGNALING, (115) (GO) PHA(GO)CYTOSIS, (116) (GO) REGULATION OF ANATOMICAL STRUCTURE MORPHOGENESIS, (117) (GO) POSITIVE REGULATION OF ERK1 AND ERK2 CASCADE, (118) (GO) INTEGRIN MEDIATED SIGNALING PATHWAY, (119) (GO) ENDOPLASMIC RETICULUM LUMEN, (120) (GO) REPRODUCTION, (121) (GO) ACTIVATION OF IMMUNE RESPONSE, (122) (GO) REGULATION OF DEFENSE RESPONSE	
Down_AD	(1) (H)K MTORC1 SIGNALING, (2) (R) CELL CYCLE CHECKPOINTS, (3) (R) G2 M CHECKPOINTS, (4) (R) CELL CYCLE, (5) (R) M PHASE, (6) (R) CELL CYCLE MITOTIC, (7) (R) MITOTIC G1 PHASE AND G1 S TRANSITION, (8) (R) MHC CLASS II ANTIGEN PRESENTATION, (9) (R) NERVOUS SYSTEM DEVELOPMENT, (10) (GO) GLUTAMATERGIC SYNAPSE, (11) (GO) NEURON PROJECTION, (12) (GO) POSTSYNAPSE, (13) (GO) SOMATODENDRITIC COMPARTMENT, (14) (GO) SYNAPSE, (15) (GO) SYNAPTIC MEMBRANE, (16) (GO) SYNAPTIC SIGNALING, (17) (GO) DENDRITIC TREE, (18) (GO) REGULATION OF TRANS SYNAPTIC SIGNALING, (19) (GO) ANTIGEN PROCESSING AND PRESENTATION OF PEPTIDE ANTIGEN, (20) (GO) SYNAPSE ORGANIZATION, (21) (GO) ORGANELLE SUBCOMPARTMENT, (22) (GO) REGULATION OF NEURON PROJECTION DEVELOPMENT, (23) (GO) POSTSYNAPSE ORGANIZATION, (24) (GO) ANTIGEN PROCESSING AND PRESENTATION	(1) (H)K OXIDATIVE PHOSPHORYLATION, (2) (K) HUNTINGTONS DISEASE, (3) (K) OXIDATIVE PHOSPHORYLATION, (4) (K) PARKINSONS DISEASE, (5) (R) COMPLEX I BIOGENESIS, (6) (R) METABOLISM OF RNA, (7) (R) RESPIRATORY ELECTRON TRANSPORT, (8) (R) RESPIRATORY ELECTRON TRANSPORT ATP SYNTHESIS BY CHEMIOSMOTIC COUPLING AND HEAT PRODUCTION BY UNCOUPLING PROTEINS, (9) (R) THE CITRIC ACID TCA CYCLE AND RESPIRATORY ELECTRON TRANSPORT, (10) (R) TRANSLATION, (11) (R) PROTEIN LOCALIZATION, (12) (R) CRISTAE FORMATION, (13) (R) PROCESSING OF CAPPED INTRON CONTAINING PRE MRNA, (14) (R) FORMATION OF ATP BY

		CHEMIOSMOTIC COUPLING, (15) (R) MITOCHONDRIAL BIOGENESIS, (16) (R) MRNA SPLICING, (17) (R) METABOLISM OF AMINO ACIDS AND DERIVATIVES, (18) (GO) ATP METABOLIC PROCESS, (19) (GO) ATP SYNTHESIS COUPLED ELECTRON TRANSPORT, (20) (GO) CATALYTIC COMPLEX, (21) (GO) CELLULAR RESPIRATION, (22) (GO) ELECTRON TRANSPORT CHAIN, (23) (GO) ENERGY DERIVATION BY OXIDATION OF ORGANIC COMPOUNDS, (24) (GO) ENVELOPE, (25) (GO) GENERATION OF PRECURSOR METABOLITES AND ENERGY, (26) (GO) INNER MITOCHONDRIAL MEMBRANE PROTEIN COMPLEX, (27) (GO) INTRACELLULAR PROTEIN TRANSPORT, (28) (GO) INTRACELLULAR TRANSPORT, (29) (GO) MITOCHONDRIAL ELECTRON TRANSPORT NADH TO UBIQUINONE, (30) (GO) MITOCHONDRIAL ENVELOPE, (31) (GO) MITOCHONDRIAL MATRIX, (32) (GO) MITOCHONDRIAL PROTEIN COMPLEX, (33) (GO) MITOCHONDRIAL RESPIRATORY CHAIN COMPLEX ASSEMBLY, (34) (GO) MITOCHONDRION, (35) (GO) MITOCHONDRION ORGANIZATION, (36) (GO) NADH DEHYDROGENASE ACTIVITY, (37) (GO) NADH DEHYDROGENASE COMPLEX, (38) (GO) NADH DEHYDROGENASE COMPLEX ASSEMBLY, (39) (GO) ORGANELLE INNER MEMBRANE, (40) (GO) OXIDATIVE PHOSPHORYLATION, (41) (GO) PEPTIDE BIOSYNTHETIC PROCESS, (42) (GO) RESPIRASOME, (43) (GO) RESPIRATORY CHAIN COMPLEX, (44) (GO) RESPIRATORY ELECTRON TRANSPORT CHAIN, (45) (GO) RIBONUCLEOPROTEIN COMPLEX, (46) (GO) RIBOSOME, (47) (GO) RNA BINDING, (48) (GO) RNA PROCESSING, (49) (GO) RIBOSOMAL SUBUNIT, (50) (GO) OXIDOREDUCTASE ACTIVITY ACTING ON NAD P H QUINONE OR SIMILAR COMPOUND AS ACCEPTOR, (51) (GO) PROTEIN TARGETING, (52) (GO) STRUCTURAL CONSTITUENT OF RIBOSOME, (53) (GO) MRNA METABOLIC PROCESS, (54) (GO) OXIDATION REDUCTION PROCESS, (55) (GO) RIBONUCLEOPROTEIN COMPLEX SUBUNIT ORGANIZATION, (56) (GO) CRISTAE FORMATION, (57) (GO) ATP SYNTHESIS COUPLED PROTON TRANSPORT, (58) (GO) PROTON TRANSPORTING ATP SYNTHASE COMPLEX, (59) (GO) RNA SPLICING VIA TRANSESTERIFICATION REACTIONS, (60) (GO) OXIDOREDUCTASE ACTIVITY, (61) (GO) MRNA PROCESSING, (62) (GO)

		**RNA SPLICING, (63) (GO) SMALL RIBOSOMAL SUBUNIT**

Table S5. Jointly deregulated pathways in AD and BLCA.

	Up_BLCA	Down_BLCA
Up_AD	(1) (R) KERATINIZATION, (2) (R) TRANSCRIPTIONAL REGULATION OF GRANULOPOIESIS, (3) (R) FORMATION OF THE CORNIFIED ENVELOPE, (4) (R) RUNX1 REGULATES GENES INVOLVED IN MEGAKARYOCYTE DIFFERENTIATION AND PLATELET FUNCTION, (5) (GO) CHROMATIN, (6) (GO) CHROMOSOME, (7) (GO) NUCLEAR CHROMOSOME, (8) (GO) KERATINIZATION, (9) (GO) CHROMATIN BINDING, (10) (GO) DNA PACKAGING COMPLEX	(1) (H)K INFLAMMATORY RESPONSE, (2) (H)K EPITHELIAL MESENCHYMAL TRANSITION, (3) (H)K ALLOGRAFT REJECTION, (4) (H)K MYOGENESIS, (5) (H)K COAGULATION, (6) (H)K TNFA SIGNALING VIA NFKB, (7) (H)K IL2 STAT5 SIGNALING, (8) (K) CYTOKINE CYTOKINE RECEPTOR INTERACTION, (9) NABA CORE MATRISOME, (10) NABA MATRISOME, (11) (R) EXTRACELLULAR MATRIX ORGANIZATION, (12) (R) IMMUNOREGULATORY INTERACTIONS BETWEEN A LYMPHOID AND A NON LYMPHOID CELL, (13) (K) HEMATOPOIETIC CELL LINEAGE, (14) NABA ECM GLYCOPROTEINS, (15) (R) ELASTIC FIBRE FORMATION, (16) (K) COMPLEMENT AND COAGULATION CASCADES, (17) (R) COMPLEMENT CASCADE, (18) (R) MOLECULES ASSOCIATED WITH ELASTIC FIBRES, (19) (K) CELL ADHESION MOLECULES CAMS, (20) (PID) INTEGRIN1 PATHWAY, (21) (R) INTEGRIN CELL SURFACE INTERACTIONS, (22) (K) JAK STAT SIGNALING PATHWAY, (23) (K) GRAFT VERSUS HOST DISEASE, (24) (GO) ADAPTIVE IMMUNE RESPONSE, (25) (GO) ALPHA BETA T CELL ACTIVATION, (26) (GO) ANATOMICAL STRUCTURE FORMATION INVOLVED IN MORPHOGENESIS, (27) (GO) ANIMAL ORGAN MORPHOGENESIS, (28) (GO) BIOLOGICAL ADHESION, (29) (GO) BLOOD VESSEL MORPHOGENESIS, (30) (GO) BONE DEVELOPMENT, (31) (GO) CARDIOVASCULAR SYSTEM DEVELOPMENT, (32) (GO) CELL ACTIVATION, (33) (GO) CELL CELL ADHESION, (34) (GO) CELL SURFACE, (35) (GO) CIRCULATORY SYSTEM DEVELOPMENT, (36) (GO) COLLAGEN CONTAINING EXTRACELLULAR MATRIX, (37) (GO) CONNECTIVE TISSUE DEVELOPMENT, (38) (GO) CYTOKINE MEDIATED SIGNALING PATHWAY, (39) (GO) CYTOKINE PRODUCTION, (40) (GO) EMBRYONIC MORPHOGENESIS, (41) (GO) EXTERNAL SIDE OF PLASMA MEMBRANE, (42) (GO) EXTRACELLULAR MATRIX, (43) (GO) EXTRACELLULAR MATRIX STRUCTURAL CONSTITUENT, (44) (GO) EXTRACELLULAR STRUCTURE ORGANIZATION, (45) (GO) IMMUNE RECEPTOR ACTIVITY, (46) (GO) IMMUNE SYSTEM DEVELOPMENT, (47) (GO) INFLAMMATORY RESPONSE, (48) (GO) LEUKOCYTE CELL CELL ADHESION, (49) (GO) LEUKOCYTE DIFFERENTIATION,

		(50) (GO) LEUKOCYTE MIGRATION, (51) (GO) LEUKOCYTE PROLIFERATION, (52) (GO) LYMPHOCYTE ACTIVATION, (53) (GO) LYMPHOCYTE DIFFERENTIATION, (54) (GO) MOLECULAR TRANSDUCER ACTIVITY, (55) (GO) NEGATIVE REGULATION OF DEVELOPMENTAL PROCESS, (56) (GO) NEGATIVE REGULATION OF IMMUNE SYSTEM PROCESS, (57) (GO) NEGATIVE REGULATION OF MULTICELLULAR ORGANISMAL PROCESS, (58) (GO) POSITIVE REGULATION OF CELL POPULATION PROLIFERATION, (59) (GO) POSITIVE REGULATION OF DEVELOPMENTAL PROCESS, (60) (GO) POSITIVE REGULATION OF IMMUNE SYSTEM PROCESS, (61) (GO) POSITIVE REGULATION OF VASCULATURE DEVELOPMENT, (62) (GO) REGULATION OF CELL ACTIVATION, (63) (GO) REGULATION OF CELL ADHESION, (64) (GO) REGULATION OF IMMUNE SYSTEM PROCESS, (65) (GO) REGULATION OF LEUKOCYTE PROLIFERATION, (66) (GO) REGULATION OF LYMPHOCYTE ACTIVATION, (67) (GO) REGULATION OF VASCULATURE DEVELOPMENT, (68) (GO) RESPONSE TO BACTERIUM, (69) (GO) RESPONSE TO BIOTIC STIMULUS, (70) (GO) RESPONSE TO LI(PID), (71) (GO) RESPONSE TO MOLECULE OF BACTERIAL ORIGIN, (72) (GO) RESPONSE TO WOUNDING, (73) (GO) SKELETAL SYSTEM DEVELOPMENT, (74) (GO) TUBE DEVELOPMENT, (75) (GO) TUBE MORPHOGENESIS, (76) (GO) T CELL ACTIVATION, (77) (GO) UROGENITAL SYSTEM DEVELOPMENT, (78) (GO) WOUND HEALING, (79) (GO) RENAL SYSTEM DEVELOPMENT, (80) (GO) CARTILAGE DEVELOPMENT, (81) (GO) REGULATION OF LEUKOCYTE DIFFERENTIATION, (82) (GO) NEGATIVE REGULATION OF CYTOKINE PRODUCTION, (83) (GO) T CELL DIFFERENTIATION, (84) (GO) POSITIVE REGULATION OF CELL ACTIVATION, (85) (GO) CYTOKINE RECEPTOR ACTIVITY, (86) (GO) RESPONSE TO CYTOKINE, (87) (GO) REGULATION OF T CELL ACTIVATION, (88) (GO) REGULATION OF CELL CELL ADHESION, (89) (GO) CYTOKINE BINDING, (90) (GO) MUSCLE STRUCTURE DEVELOPMENT, (91) (GO) POSITIVE REGULATION OF CELL ADHESION, (92) (GO) T CELL PROLIFERATION, (93) (GO) DEFENSE RESPONSE TO OTHER ORGANISM, (94) (GO) REGULATION OF IMMUNE RESPONSE, (95) (GO) HEART DEVELOPMENT, (96) (GO) REGULATION OF IMMUNE EFFECTOR PROCESS, (97)

		(GO) MORPHOGENESIS OF A BRANCHING STRUCTURE, (98) (GO) B CELL ACTIVATION, (99) (GO) NEGATIVE REGULATION OF CELL DIFFERENTIATION, (100) (GO) POSITIVE REGULATION OF INTRACELLULAR SIGNAL TRANSDUCTION, (101) (GO) RECEPTOR COMPLEX, (102) (GO) TUMOR NECROSIS FACTOR SUPERFAMILY CYTOKINE PRODUCTION, (103) (GO) NEGATIVE REGULATION OF CELL ACTIVATION, (104) (GO) GLAND MORPHOGENESIS, (105) (GO) SIDE OF MEMBRANE, (106) (GO) MYELOID CELL DIFFERENTIATION, (107) (GO) TISSUE REMODELING, (108) (GO) POSITIVE REGULATION OF LEUKOCYTE CELL CELL ADHESION, (109) (GO) B CELL DIFFERENTIATION, (110) (GO) T CELL ACTIVATION INVOLVED IN IMMUNE RESPONSE, (111) (GO) INTERLEUKIN 8 PRODUCTION, (112) (GO) ADAPTIVE IMMUNE RESPONSE BASED ON SOMATIC RECOMBINATION OF IMMUNE RECEPTORS BUILT FROM IMMUNOGLOBULIN SUPERFAMILY DOMAINS, (113) (GO) REGULATION OF CELLULAR COMPONENT MOVEMENT, (114) (GO) ALPHA BETA T CELL DIFFERENTIATION, (115) (GO) NEGATIVE REGULATION OF LYMPHOCYTE ACTIVATION, (116) (GO) POSITIVE REGULATION OF CELL CELL ADHESION, (117) (GO) INTERLEUKIN 6 PRODUCTION, (118) (GO) POSITIVE REGULATION OF RECEPTOR SIGNALING PATHWAY VIA STAT, (119) (GO) GASTRULATION, (120) (GO) MUSCLE ORGAN DEVELOPMENT, (121) (GO) FORMATION OF PRIMARY GERM LAYER, (122) (GO) NEGATIVE REGULATION OF HEMOPOIESIS, (123) (GO) EPITHELIAL CELL PROLIFERATION, (124) (GO) POSITIVE REGULATION OF LOCOMOTION, (125) (GO) CELL SUBSTRATE ADHESION, (126) (GO) SIGNALING RECEPTOR BINDING, (127) (GO) POSITIVE REGULATION OF PHOSPHATIDYLINOSITOL 3 KINASE SIGNALING, (128) (GO) HEART MORPHOGENESIS, (129) (GO) POSITIVE REGULATION OF LEUKOCYTE PROLIFERATION, (130) (GO) OSSIFICATION, (131) (GO) INNATE IMMUNE RESPONSE, (132) (GO) COLLAGEN TRIMER, (133) (GO) RESPONSE TO BMP, (134) (GO) REGULATION OF CARTILAGE DEVELOPMENT, (135) (GO) RESPONSE TO GROWTH FACTOR, (136) (GO) POSITIVE REGULATION OF TYROSINE PHOSPHORYLATION OF STAT PROTEIN, (137) (GO) CYTOKINE SECRETION, (138)

		(GO) CELL CHEMOTAXIS, (139) (GO) POSITIVE REGULATION OF MAPK CASCADE, (140) (GO) GLOMERULUS DEVELOPMENT, (141) (GO) POSITIVE REGULATION OF CELL DIFFERENTIATION, (142) (GO) POSITIVE REGULATION OF IMMUNE RESPONSE, (143) (GO) POSITIVE REGULATION OF CYTOKINE PRODUCTION, (144) (GO) REGULATION OF MYELOID CELL DIFFERENTIATION, (145) (GO) POSITIVE REGULATION OF PHOSPHORUS METABOLIC PROCESS, (146) (GO) CELL ADHESION MEDIATED BY INTEGRIN, (147) (GO) REGULATION OF INFLAMMATORY RESPONSE, (148) (GO) IMMUNE EFFECTOR PROCESS, (149) (GO) CHONDROCYTE DIFFERENTIATION, (150) (GO) OUTFLOW TRACT MORPHOGENESIS, (151) (GO) LYMPHOCYTE MEDIATED IMMUNITY, (152) (GO) ERK1 AND ERK2 CASCADE, (153) (GO) POSITIVE REGULATION OF T CELL PROLIFERATION, (154) (GO) CELLULAR RESPONSE TO LI(PID), (155) (GO) MESENCHYME DEVELOPMENT, (156) (GO) COAGULATION, (157) (GO) REGULATION OF RESPONSE TO EXTERNAL STIMULUS, (158) (GO) GRANULOCYTE MIGRATION, (159) (GO) BASEMENT MEMBRANE, (160) (GO) POSITIVE REGULATION OF HEMOPOIESIS, (161) (GO) POSITIVE REGULATION OF LEUKOCYTE DIFFERENTIATION, (162) (GO) REGULATION OF HEMOPOIESIS, (163) (GO) POSITIVE REGULATION OF EPITHELIAL CELL PROLIFERATION, (164) (GO) ENDOTHELIUM DEVELOPMENT, (165) (GO) ANCHORING JUNCTION, (166) (GO) NEGATIVE REGULATION OF CELL POPULATION PROLIFERATION, (167) (GO) TAXIS, (168) (GO) COMPLEMENT ACTIVATION, (169) (GO) NEGATIVE REGULATION OF CELL ADHESION, (170) (GO) POSITIVE REGULATION OF PROTEIN MODIFICATION PROCESS, (171) (GO) PEPTIDYL TYROSINE MODIFICATION, (172) (GO) REGULATION OF HUMORAL IMMUNE RESPONSE, (173) (GO) POSITIVE REGULATION OF LYMPHOCYTE DIFFERENTIATION, (174) (GO) REGULATION OF HEART MORPHOGENESIS, (175) (GO) I KAPPAB KINASE NF KAPPAB SIGNALING, (176) (GO) POSITIVE REGULATION OF PEPTIDYL TYROSINE PHOSPHORYLATION, (177) (GO) PHA(GO)CYTOSIS, (178) (GO) POSITIVE REGULATION OF TUMOR NECROSIS FACTOR SUPERFAMILY CYTOKINE PRODUCTION, (179) (GO) POSITIVE

		REGULATION OF LEUKOCYTE MIGRATION, (180) (GO) REGULATION OF CELLULAR RESPONSE TO GROWTH FACTOR STIMULUS, (181) (GO) REGULATION OF ANATOMICAL STRUCTURE MORPHOGENESIS, (182) (GO) B CELL RECEPTOR SIGNALING PATHWAY, (183) (GO) MYELOID LEUKOCYTE MIGRATION, (184) (GO) POSITIVE REGULATION OF ERK1 AND ERK2 CASCADE, (185) (GO) INTEGRIN MEDIATED SIGNALING PATHWAY, (186) (GO) PROTEIN COMPLEX INVOLVED IN CELL ADHESION, (187) (GO) TRANSMEMBRANE RECEPTOR PROTEIN SERINE THREONINE KINASE SIGNALING PATHWAY, (188) (GO) ACTIVATION OF IMMUNE RESPONSE, (189) (GO) REGULATION OF ANTIGEN RECEPTOR MEDIATED SIGNALING PATHWAY, (190) (GO) MUSCLE SYSTEM PROCESS, (191) (GO) MESENCHYME MORPHOGENESIS, (192) (GO) INTERFERON GAMMA PRODUCTION, (193) (GO) REGULATION OF DEFENSE RESPONSE
Down_AD	(1) (H)K MYC TARGETS V1, (2) (H)K MTORC1 SIGNALING, (3) (H)K DNA REPAIR, (4) (H)K UNFOLDED PROTEIN RESPONSE, (5) (R) APC C CDH1 MEDIATED DEGRADATION OF CDC20 AND OTHER APC C CDH1 TARGETED PROTEINS IN LATE MITOSIS EARLY G1, (6) (R) ASSEMBLY OF THE PRE REPLICATIVE COMPLEX, (7) (R) CELL CYCLE CHECKPOINTS, (8) (R) DNA REPLICATION, (9) (R) METABOLISM OF RNA, (10) (R) MITOCHONDRIAL TRANSLATION, (11) (R) MITOTIC G2 G2 M PHASES, (12) (R) MITOTIC METAPHASE AND ANAPHASE, (13) (R) ORC1 REMOVAL FROM CHROMATIN, (14) (R) SEPARATION OF SISTER CHROMATIDS, (15) (R) SWITCHING OF ORIGINS TO A POST REPLICATIVE STATE, (16) (R) TRANSLATION, (17) (R) METABOLISM OF POLYAMINES, (18) (R) THE ROLE OF GTSE1 IN G2 M PROGRESSION AFTER G2 CHECKPOINT, (19) (R) APC C MEDIATED DEGRADATION OF CELL CYCLE PROTEINS, (20) (R) DNA REPLICATION PRE INITIATION, (21) (R) SCF SKP2 MEDIATED DEGRADATION OF P27 P21, (22) (R) S PHASE, (23) (R) G2 M CHECKPOINTS, (24) (R) NUCLEOTIDE EXCISION REPAIR, (25) (R) CYCLIN A CDK2 ASSOCIATED EVENTS AT S PHASE ENTRY, (26) (R) PROCESSING OF CAPPED INTRON CONTAINING PRE MRNA, (27) (R) G1 S DNA DAMAGE CHECKPOINTS, (28) (R) CELL CYCLE, (29) (R) M PHASE, (30) (R) CELL CYCLE MITOTIC, (31) (R) MRNA SPLICING, (32) (R) GLOBAL GENOME NUCLEOTIDE EXCISION REPAIR GG NER, (33) (R) MITOTIC G1 PHASE AND G1 S TRANSITION, (34) (R) TRNA PROCESSING, (35) (R) MRNA SPLICING MINOR PATHWAY, (36) (R) RRNA PROCESSING, (37) (R) VIRAL MESSENGER RNA SYNTHESIS, (38) (R) RRNA MODIFICATION IN THE NUCLEUS AND CYTOSOL, (39) (R) DUAL INCISION IN TC NER, (40) (GO) ANAPHASE PROMOTING COMPLEX DEPENDENT CATABOLIC PROCESS, (41) (GO) CATALYTIC ACTIVITY ACTING ON RNA, (42) (GO) CATALYTIC COMPLEX, (43) (GO) CELLULAR PROTEIN CONTAINING COMPLEX ASSEMBLY, (44) (GO) ENVELOPE, (45) (GO)	

	HYDROLASE ACTIVITY ACTING ON ACID ANHYDRIDES, (46) (GO) MITOCHONDRIAL ENVELOPE, (47) (GO) MITOCHONDRIAL GENE EXPRESSION, (48) (GO) MITOCHONDRIAL MATRIX, (49) (GO) MITOCHONDRIAL PROTEIN COMPLEX, (50) (GO) MITOCHONDRIAL TRANSLATION, (51) (GO) MITOCHONDRIAL TRANSLATIONAL TERMINATION, (52) (GO) MITOCHONDRION, (53) (GO) NCRNA METABOLIC PROCESS, (54) (GO) NCRNA PROCESSING, (55) (GO) ORGANELLAR RIBOSOME, (56) (GO) ORGANELLE INNER MEMBRANE, (57) (GO) RIBONUCLEOPROTEIN COMPLEX, (58) (GO) RIBONUCLEOPROTEIN COMPLEX BIOGENESIS, (59) (GO) RIBOSOME BIOGENESIS, (60) (GO) RNA BINDING, (61) (GO) RNA PROCESSING, (62) (GO) TRANSLATIONAL ELONGATION, (63) (GO) TRANSLATIONAL TERMINATION, (64) (GO) TRNA METABOLIC PROCESS, (65) (GO) CELLULAR PROTEIN COMPLEX DISASSEMBLY, (66) (GO) RIBONUCLEOTIDE BINDING, (67) (GO) REGULATION OF CELL CYCLE G2 M PHASE TRANSITION, (68) (GO) CATALYTIC ACTIVITY ACTING ON A TRNA, (69) (GO) CELL CYCLE G2 M PHASE TRANSITION, (70) (GO) NUCLEOLUS, (71) (GO) NEGATIVE REGULATION OF CELL CYCLE G2 M PHASE TRANSITION, (72) (GO) POSTTRANSCRIPTIONAL REGULATION OF GENE EXPRESSION, (73) (GO) RRNA METABOLIC PROCESS, (74) (GO) MITOCHONDRIAL LARGE RIBOSOMAL SUBUNIT, (75) (GO) TRANSFERASE COMPLEX, (76) (GO) MRNA METABOLIC PROCESS, (77) (GO) RIBONUCLEOPROTEIN COMPLEX SUBUNIT ORGANIZATION, (78) (GO) MICROTUBULE, (79) (GO) MICROTUBULE CYTOSKELETON, (80) (GO) ADENYL NUCLEOTIDE BINDING, (81) (GO) MICROTUBULE BASED PROCESS, (82) (GO) TRNA PROCESSING, (83) (GO) RNA SPLICING VIA TRANSESTERIFICATION REACTIONS, (84) (GO) NUCLEOTIDE EXCISION REPAIR, (85) (GO) DRUG BINDING, (86) (GO) ATPASE ACTIVITY, (87) (GO) MRNA PROCESSING, (88) (GO) SPLICEOSOMAL TRI SNRNP COMPLEX, (89) (GO) RNA LOCALIZATION, (90) (GO) RETROGRADE VESICLE MEDIATED TRANSPORT (GO)LGI TO ENDOPLASMIC RETICULUM, (91) (GO) RNA SPLICING, (92) (GO) PRERIBOSOME	

Table S6. Jointly deregulated pathways in AD and BRCA.

	Up_BRCA	Down_BRCA
Up_AD	(1) (H)K ALLOGRAFT REJECTION, (2) (R) TRANSCRIPTIONAL REGULATION OF GRANULOPOIESIS, (3) (R) RUNX1 REGULATES GENES INVOLVED IN MEGAKARYOCYTE DIFFERENTIATION AND PLATELET FUNCTION, (4) (GO) CHROMOSOME, (5) (GO) NUCLEAR CHROMOSOME, (6) (GO) DEFENSE RESPONSE TO OTHER ORGANISM, (7) (GO) REGULATION OF IMMUNE RESPONSE, (8) (GO) INNATE IMMUNE RESPONSE, (9) (GO) IMMUNE EFFECTOR PROCESS, (10) (GO) CHROMATIN BINDING, (11) (GO) REPRODUCTION, (12) (GO) DNA PACKAGING COMPLEX	(1) (H)K MYOGENESIS, (2) (H)K TNFA SIGNALING VIA NFKB, (3) (H)K HYPOXIA, (4) NABA CORE MATRISOME, (5) NABA MATRISOME, (6) NABA ECM GLYCOPROTEINS, (7) (GO) ANATOMICAL STRUCTURE FORMATION INVOLVED IN MORPHOGENESIS, (8) (GO) ANIMAL ORGAN MORPHOGENESIS, (9) (GO) BIOLOGICAL ADHESION, (10) (GO) BLOOD VESSEL MORPHOGENESIS, (11) (GO) CARDIOVASCULAR SYSTEM DEVELOPMENT, (12) (GO) CELL MOTILITY, (13) (GO) CIRCULATORY SYSTEM DEVELOPMENT, (14) (GO) COLLAGEN CONTAINING EXTRACELLULAR MATRIX, (15) (GO)

		DNA BINDING TRANSCRIPTION ACTIVATOR ACTIVITY, (16) (GO) DNA BINDING TRANSCRIPTION FACTOR ACTIVITY RNA POLYMERASE II SPECIFIC, (17) (GO) EXTRACELLULAR MATRIX, (18) (GO) LOCOMOTION, (19) (GO) NEGATIVE REGULATION OF DEVELOPMENTAL PROCESS, (20) (GO) NEGATIVE REGULATION OF MULTICELLULAR ORGANISMAL PROCESS, (21) (GO) POSITIVE REGULATION OF DEVELOPMENTAL PROCESS, (22) (GO) POSITIVE REGULATION OF TRANSCRIPTION BY RNA POLYMERASE II, (23) (GO) POSITIVE REGULATION OF VASCULATURE DEVELOPMENT, (24) (GO) REGULATION OF CELL POPULATION PROLIFERATION, (25) (GO) REGULATION OF VASCULATURE DEVELOPMENT, (26) (GO) RESPONSE TO LI(PID), (27) (GO) TUBE DEVELOPMENT, (28) (GO) TUBE MORPHOGENESIS, (29) (GO) UROGENITAL SYSTEM DEVELOPMENT, (30) (GO) POSITIVE REGULATION OF MULTICELLULAR ORGANISMAL PROCESS, (31) (GO) RENAL SYSTEM DEVELOPMENT, (32) (GO) MUSCLE STRUCTURE DEVELOPMENT, (33) (GO) HEART DEVELOPMENT, (34) (GO) REGULATION OF CELL DIFFERENTIATION, (35) (GO) KIDNEY EPITHELIUM DEVELOPMENT, (36) (GO) REGULATION OF CELLULAR COMPONENT MOVEMENT, (37) (GO) MUSCLE ORGAN DEVELOPMENT, (38) (GO) EPITHELIAL CELL PROLIFERATION, (39) (GO) POSITIVE REGULATION OF LOCOMOTION, (40) (GO) CELL SUBSTRATE ADHESION, (41) (GO) SIGNALING RECEPTOR BINDING, (42) (GO) RESPONSE TO GROWTH FACTOR, (43) (GO) POSITIVE REGULATION OF MAPK CASCADE, (44) (GO) POSITIVE REGULATION OF CELL DIFFERENTIATION, (45) (GO) POSITIVE REGULATION OF PHOSPHORUS METABOLIC PROCESS, (46) (GO) MULTICELLULAR ORGANISMAL HOMEOSTASIS, (47) (GO) POSITIVE REGULATION OF EPITHELIAL CELL PROLIFERATION, (48) (GO) ENDOTHELIUM DEVELOPMENT, (49) (GO) ANCHORING JUNCTION, (50) (GO) NEGATIVE REGULATION OF CELL POPULATION PROLIFERATION, (51) (GO) TAXIS, (52) (GO) REGULATION OF FAT CELL DIFFERENTIATION, (53) (GO) REGULATION OF ANATOMICAL STRUCTURE MORPHOGENESIS, (54) (GO) MUSCLE SYSTEM PROCESS, (55) (GO) MESENCHYME MORPHOGENESIS

Down_AD	(1) (H)K MYC TARGETS V1, (2) (H)K MTORC1 SIGNALING, (3) (H)K DNA REPAIR, (4) (H)K UNFOLDED PROTEIN RESPONSE, (5) (K) PROTEASOME, (6) (R) APC C CDH1 MEDIATED DEGRADATION OF CDC20 AND OTHER APC C CDH1 TARGETED PROTEINS IN LATE MITOSIS EARLY G1, (7) (R) ASPARAGINE N LINKED GLYCOSYLATION, (8) (R) ASSEMBLY OF THE PRE REPLICATIVE COMPLEX, (9) (R) CELL CYCLE CHECKPOINTS, (10) (R) DNA REPLICATION, (11) (R) HIV INFECTION, (12) (R) HOST INTERACTIONS OF HIV FACTORS, (13) (R) METABOLISM OF RNA, (14) (R) MITOCHONDRIAL TRANSLATION, (15) (R) MITOTIC G2 G2 M PHASES, (16) (R) MITOTIC METAPHASE AND ANAPHASE, (17) (R) ORC1 REMOVAL FROM CHROMATIN, (18) (R) SEPARATION OF SISTER CHROMATIDS, (19) (R) SWITCHING OF ORIGINS TO A POST REPLICATIVE STATE, (20) (R) METABOLISM OF POLYAMINES, (21) (R) POST TRANSLATIONAL PROTEIN MODIFICATION, (22) (R) THE ROLE OF GTSE1 IN G2 M PROGRESSION AFTER G2 CHECKPOINT, (23) (R) APC C MEDIATED DEGRADATION OF CELL CYCLE PROTEINS, (24) (R) DNA REPLICATION PRE INITIATION, (25) (R) STABILIZATION OF P53, (26) (R) SCF SKP2 MEDIATED DEGRADATION OF P27 P21, (27) (R) S PHASE, (28) (R) G2 M CHECKPOINTS, (29) (R) NUCLEOTIDE EXCISION REPAIR, (30) (R) CYCLIN A CDK2 ASSOCIATED EVENTS AT S PHASE ENTRY, (31) (R) PROCESSING OF CAPPED INTRON CONTAINING PRE MRNA, (32) (R) G1 S DNA DAMAGE CHECKPOINTS, (33) (R) CELL CYCLE, (34) (R) M PHASE, (35) (R) UCH PROTEINASES, (36) (R) HIV LIFE CYCLE, (37) (R) CELL CYCLE MITOTIC, (38) (R) (GO)LGI TO ER RETROGRADE TRANSPORT, (39) (R) MRNA SPLICING, (40) (R) GLOBAL GENOME NUCLEOTIDE EXCISION REPAIR GG NER, (41) (R) MITOTIC G1 PHASE AND G1 S TRANSITION, (42) (R) UB SPECIFIC PROCESSING PROTEASES, (43) (R) MHC CLASS II ANTIGEN PRESENTATION, (44) (R) DEUBIQUITINATION, (45) (R) TRNA PROCESSING, (46) (R) ANTIGEN PROCESSING CROSS PRESENTATION, (47) (R) VIRAL MESSENGER RNA SYNTHESIS, (48) (GO) ANAPHASE PROMOTING COMPLEX DEPENDENT CATABOLIC PROCESS, (49) (GO) CATALYTIC ACTIVITY ACTING ON RNA, (50) (GO) CATALYTIC COMPLEX, (51) (GO) CELLULAR PROTEIN CONTAINING COMPLEX ASSEMBLY, (52) (GO) ENDOPEPTIDASE COMPLEX, (53) (GO) HYDROLASE ACTIVITY ACTING ON ACID ANHYDRIDES, (54) (GO) MITOCHONDRIAL GENE EXPRESSION, (55) (GO) MITOCHONDRIAL TRANSLATION, (56) (GO) NCRNA METABOLIC PROCESS, (57) (GO) NCRNA PROCESSING, (58) (GO) PROTEIN CONTAINING COMPLEX ASSEMBLY, (59) (GO) PROTEIN MODIFICATION BY SMALL PROTEIN CONJUGATION, (60) (GO) PROTEIN MODIFICATION BY SMALL PROTEIN CONJUGATION OR REMOVAL, (61) (GO) RIBONUCLEOPROTEIN COMPLEX BIOGENESIS, (62) (GO) RNA BINDING, (63) (GO) RNA PROCESSING, (64) (GO) TRNA METABOLIC PROCESS, (65) (GO) RIBONUCLEOTIDE BINDING, (66) (GO) REGULATION OF CELL CYCLE G2 M PHASE TRANSITION, (67) (GO) CATALYTIC ACTIVITY ACTING ON A TRNA, (68) (GO) CELL CYCLE G2 M PHASE TRANSITION, (69) (GO) NUCLEOLUS, (70) (GO) TRANSFERASE COMPLEX, (71) (GO) ORGANELLE LOCALIZATION, (72) (GO) MICROTUBULE, (73) (GO) MICROTUBULE CYTOSKELETON, (74) (GO) ADENYL NUCLEOTIDE BINDING, (75) (GO) ANTIGEN PROCESSING AND PRESENTATION OF PEPTIDE ANTIGEN, (76) (GO) MICROTUBULE BASED PROCESS, (77) (GO) PROTEIN	(1) (GO) SYNAPSE

	CONTAINING COMPLEX LOCALIZATION, (78) (GO) DRUG BINDING, (79) (GO) ATPASE ACTIVITY, (80) (GO) ESTABLISHMENT OF ORGANELLE LOCALIZATION, (81) (GO) RNA LOCALIZATION, (82) (GO) RETROGRADE VESICLE MEDIATED TRANSPORT (GO)LGI TO ENDOPLASMIC RETICULUM, (83) (GO) ANTIGEN PROCESSING AND PRESENTATION	

Table S7. Jointly deregulated pathways in AD and CERV.

	Up_CERV	Down_CERV
Up_AD	(1) (H)K INFLAMMATORY RESPONSE, (2) (H)K ALLOGRAFT REJECTION, (3) (H)K IL6 JAK STAT3 SIGNALING, (4) (R) TRANSCRIPTIONAL REGULATION OF GRANULOPOIESIS, (5) (GO) ADAPTIVE IMMUNE RESPONSE, (6) (GO) CHROMATIN, (7) (GO) CHROMOSOME, (8) (GO) CYTOKINE MEDIATED SIGNALING PATHWAY, (9) (GO) DOUBLE STRANDED DNA BINDING, (10) (GO) IMMUNE SYSTEM DEVELOPMENT, (11) (GO) NEGATIVE REGULATION OF BIOSYNTHETIC PROCESS, (12) (GO) NEGATIVE REGULATION OF NUCLEOBASE CONTAINING COMPOUND METABOLIC PROCESS, (13) (GO) NEGATIVE REGULATION OF RNA BIOSYNTHETIC PROCESS, (14) (GO) NUCLEAR CHROMOSOME, (15) (GO) POSITIVE REGULATION OF CELLULAR BIOSYNTHETIC PROCESS, (16) (GO) POSITIVE REGULATION OF NUCLEOBASE CONTAINING COMPOUND METABOLIC PROCESS, (17) (GO) POSITIVE REGULATION OF RNA METABOLIC PROCESS, (18) (GO) SEQUENCE SPECIFIC DNA BINDING, (19) (GO) TRANSCRIPTION REGULATOR ACTIVITY, (20) (GO) RESPONSE TO CYTOKINE, (21) (GO) DEFENSE RESPONSE TO OTHER ORGANISM, (22) (GO) INNATE IMMUNE RESPONSE, (23) (GO) POSITIVE REGULATION OF IMMUNE RESPONSE, (24) (GO) IMMUNE EFFECTOR PROCESS, (25) (GO) CHROMATIN BINDING, (26) (GO) DNA PACKAGING COMPLEX	(1) (H)K MYOGENESIS, (2) (H)K KRAS SIGNALING DN, (3) NABA CORE MATRISOME, (4) NABA MATRISOME, (5) NABA MATRISOME ASSOCIATED, (6) NABA SECRETED FACTORS, (7) NABA ECM GLYCOPROTEINS, (8) (R) KERATINIZATION, (9) (R) GPCR LIGAND BINDING, (10) (R) FORMATION OF THE CORNIFIED ENVELOPE, (11) (GO) BIOLOGICAL ADHESION, (12) (GO) CELL CELL ADHESION, (13) (GO) COLLAGEN CONTAINING EXTRACELLULAR MATRIX, (14) (GO) E(PID)ERMIS DEVELOPMENT, (15) (GO) EPITHELIAL CELL DIFFERENTIATION, (16) (GO) EPITHELIUM DEVELOPMENT, (17) (GO) EXTRACELLULAR MATRIX, (18) (GO) INTRINSIC COMPONENT OF PLASMA MEMBRANE, (19) (GO) MOLECULAR TRANSDUCER ACTIVITY, (20) (GO) SKIN DEVELOPMENT, (21) (GO) TUBE DEVELOPMENT, (22) (GO) E(PID)ERMAL CELL DIFFERENTIATION, (23) (GO) KIDNEY EPITHELIUM DEVELOPMENT, (24) (GO) KERATINOCYTE DIFFERENTIATION, (25) (GO) G PROTEIN COUPLED RECEPTOR ACTIVITY, (26) (GO) MESONEPHROS DEVELOPMENT, (27) (GO) KERATINIZATION, (28) (GO) PEPTIDE RECEPTOR ACTIVITY, (29) (GO) MAMMARY GLAND MORPHOGENESIS, (30) (GO) CORNIFICATION, (31) (GO) SENSORY PERCEPTION OF CHEMICAL STIMULUS
Down_AD	(1) (H)K MYC TARGETS V1, (2) (H)K MTORC1 SIGNALING, (3) (H)K DNA REPAIR, (4) (H)K UNFOLDED PROTEIN RESPONSE, (5) (K) PROTEASOME, (6) (R) ANTIGEN PROCESSING UBIQUITINATION PROTEASOME DEGRADATION, (7) (R) APC C CDH1 MEDIATED DEGRADATION OF CDC20 AND OTHER APC C CDH1 TARGETED PROTEINS IN LATE MITOSIS EARLY G1, (8) (R) ASSEMBLY OF THE PRE REPLICATIVE COMPLEX, (9) (R) AUF1 HNRNP D0 BINDS AND DESTABILIZES MRNA, (10) (R) CELL CYCLE CHECKPOINTS, (11) (R) CLASS I MHC MEDIATED ANTIGEN PROCESSING PRESENTATION, (12) (R) DEGRADATION OF DVL, (13) (R) DNA REPLICATION, (14) (R) HIV INFECTION, (15) (R) HOST INTERACTIONS OF HIV FACTORS, (16) (R) METABOLISM OF RNA, (17) (R) MITOCHONDRIAL TRANSLATION, (18) (R) MITOTIC G2 G2 M PHASES, (19) (R) MITOTIC METAPHASE AND ANAPHASE, (20)	(1) (R) NEURONAL SYSTEM, (2) (R) TRANSMISSION ACROSS CHEMICAL SYNAPSES, (3) (GO) NEURON PROJECTION, (4) (GO) NEURON TO NEURON SYNAPSE, (5) (GO) POSTSYNAPSE, (6) (GO) SYNAPSE, (7) (GO) SYNAPTIC MEMBRANE, (8) (GO) SYNAPTIC SIGNALING, (9) (GO) REGULATION OF TRANS SYNAPTIC SIGNALING, (10) (GO) POSTSYNAPTIC MEMBRANE, (11) (GO) BEHAVIOR, (12) (GO) PRESYNAPTIC MEMBRANE, (13) (GO) CATION TRANSMEMBRANE TRANSPORT, (14) (GO) POSTSYNAPTIC SPECIALIZATION MEMBRANE, (15) (GO)

	(R) NEGATIVE REGULATION OF NOTCH4 SIGNALING, (21) (R) ORC1 REMOVAL FROM CHROMATIN, (22) (R) REGULATION OF PTEN STABILITY AND ACTIVITY, (23) (R) SEPARATION OF SISTER CHROMATIDS, (24) (R) SWITCHING OF ORIGINS TO A POST REPLICATIVE STATE, (25) (R) DEGRADATION OF AXIN, (26) (R) METABOLISM OF POLYAMINES, (27) (R) POST TRANSLATIONAL PROTEIN MODIFICATION, (28) (R) THE ROLE OF GTSE1 IN G2 M PROGRESSION AFTER G2 CHECKPOINT, (29) (R) APC C MEDIATED DEGRADATION OF CELL CYCLE PROTEINS, (30) (R) CELLULAR RESPONSE TO HYPOXIA, (31) (R) DNA REPLICATION PRE INITIATION, (32) (R) STABILIZATION OF P53, (33) (R) ORGANELLE BIOGENESIS AND MAINTENANCE, (34) (R) SCF SKP2 MEDIATED DEGRADATION OF P27 P21, (35) (R) REGULATION OF RUNX3 EXPRESSION AND ACTIVITY, (36) (R) S PHASE, (37) (R) G2 M CHECKPOINTS, (38) (R) REGULATION OF MRNA STABILITY BY PROTEINS THAT BIND AU RICH ELEMENTS, (39) (R) NUCLEOTIDE EXCISION REPAIR, (40) (R) CYCLIN A CDK2 ASSOCIATED EVENTS AT S PHASE ENTRY, (41) (R) PROCESSING OF CAPPED INTRON CONTAINING PRE MRNA, (42) (R) G1 S DNA DAMAGE CHECKPOINTS, (43) (R) CELL CYCLE, (44) (R) M PHASE, (45) (R) TRANSCRIPTION COUPLED NUCLEOTIDE EXCISION REPAIR TC NER, (46) (R) UCH PROTEINASES, (47) (R) HIV LIFE CYCLE, (48) (R) CELL CYCLE MITOTIC, (49) (R) PTEN REGULATION, (50) (R) CELLULAR RESPONSES TO EXTERNAL STIMULI, (51) (R) MRNA SPLICING, (52) (R) GLOBAL GENOME NUCLEOTIDE EXCISION REPAIR GG NER, (53) (R) MITOTIC G1 PHASE AND G1 S TRANSITION, (54) (R) UB SPECIFIC PROCESSING PROTEASES, (55) (R) INFECTIOUS DISEASE, (56) (K) RNA DEGRADATION, (57) (K) UBIQUITIN MEDIATED PROTEOLYSIS, (58) (R) DEUBIQUITINATION, (59) (R) TRNA PROCESSING, (60) (R) MRNA SPLICING MINOR PATHWAY, (61) (R) CILIUM ASSEMBLY, (62) (R) FORMATION OF INCISION COMPLEX IN GG NER, (63) (R) ANTIGEN PROCESSING CROSS PRESENTATION, (64) (R) VIRAL MESSENGER RNA SYNTHESIS, (65) (R) DUAL INCISION IN TC NER, (66) (GO) ANAPHASE PROMOTING COMPLEX DEPENDENT CATABOLIC PROCESS, (67) (GO) ATP METABOLIC PROCESS, (68) (GO) CATALYTIC ACTIVITY ACTING ON RNA, (69) (GO) CATALYTIC COMPLEX, (70) (GO) CELLULAR MACROMOLECULE CATABOLIC PROCESS, (71) (GO) CELLULAR MACROMOLECULE LOCALIZATION, (72) (GO) CELLULAR PROTEIN CATABOLIC PROCESS, (73) (GO) CELLULAR PROTEIN CONTAINING COMPLEX ASSEMBLY, (74) (GO) ENDOPEPTIDASE COMPLEX, (75) (GO) ENVELOPE, (76) (GO) HYDROLASE ACTIVITY ACTING ON ACID ANHYDRIDES, (77) (GO) INTRACELLULAR TRANSPORT, (78) (GO) MITOCHONDRIAL GENE EXPRESSION, (79) (GO) MITOCHONDRIAL PROTEIN COMPLEX, (80) (GO) MITOCHONDRIAL TRANSLATION, (81) (GO) MITOCHONDRIAL TRANSLATIONAL TERMINATION, (82) (GO) MITOCHONDRION, (83) (GO) MITOCHONDRION ORGANIZATION, (84) (GO) MODIFICATION DEPENDENT MACROMOLECULE CATABOLIC PROCESS, (85) (GO) NCRNA METABOLIC PROCESS, (86) (GO) NCRNA PROCESSING, (87) (GO) ORGANELLAR RIBOSOME, (88) (GO) ORGANELLE INNER MEMBRANE, (89) (GO) PEPTIDASE COMPLEX, (90) (GO) PEPTIDE BIOSYNTHETIC PROCESS, (91) (GO) PROTEASOMAL PROTEIN CATABOLIC PROCESS, (92) (GO) PROTEIN CONTAINING COMPLEX ASSEMBLY, (93) (GO) PROTEIN MODIFICATION BY SMALL PROTEIN CONJUGATION, (94) (GO) PROTEIN MODIFICATION BY SMALL PROTEIN	POSTSYNAPTIC DENSITY MEMBRANE, (16) (GO) INORGANIC ION TRANSMEMBRANE TRANSPORT, (17) (GO) ION TRANSMEMBRANE TRANSPORT

	CONJUGATION OR REMOVAL, (95) (GO) RIBONUCLEOPROTEIN COMPLEX, (96) (GO) RIBONUCLEOPROTEIN COMPLEX BIOGENESIS, (97) (GO) RIBOSOME BIOGENESIS, (98) (GO) RNA BINDING, (99) (GO) RNA PROCESSING, (100) (GO) TRANSLATIONAL TERMINATION, (101) (GO) TRNA METABOLIC PROCESS, (102) (GO) RIBONUCLEOTIDE BINDING, (103) (GO) REGULATION OF CELL CYCLE G2 M PHASE TRANSITION, (104) (GO) CATALYTIC ACTIVITY ACTING ON A TRNA, (105) (GO) CELL CYCLE G2 M PHASE TRANSITION, (106) (GO) PROTEIN POLYUBIQUITINATION, (107) (GO) PROTEIN CATABOLIC PROCESS, (108) (GO) NUCLEOLUS, (109) (GO) NEGATIVE REGULATION OF CELL CYCLE G2 M PHASE TRANSITION, (110) (GO) PROTEIN LOCALIZATION TO ORGANELLE, (111) (GO) PROTEIN MODIFICATION BY SMALL PROTEIN REMOVAL, (112) (GO) POSTTRANSCRIPTIONAL REGULATION OF GENE EXPRESSION, (113) (GO) RRNA METABOLIC PROCESS, (114) (GO) UBIQUITIN LIKE PROTEIN TRANSFERASE ACTIVITY, (115) (GO) MITOCHONDRIAL LARGE RIBOSOMAL SUBUNIT, (116) (GO) TRANSFERASE COMPLEX, (117) (GO) MRNA METABOLIC PROCESS, (118) (GO) RIBONUCLEOPROTEIN COMPLEX SUBUNIT ORGANIZATION, (119) (GO) ORGANELLE LOCALIZATION, (120) (GO) TRANSCRIPTION COUPLED NUCLEOTIDE EXCISION REPAIR, (121) (GO) UNFOLDED PROTEIN BINDING, (122) (GO) MICROTUBULE, (123) (GO) MICROTUBULE CYTOSKELETON, (124) (GO) ADENYL NUCLEOTIDE BINDING, (125) (GO) MACROMOLECULE CATABOLIC PROCESS, (126) (GO) MICROTUBULE BASED PROCESS, (127) (GO) TRNA PROCESSING, (128) (GO) RNA SPLICING VIA TRANSESTERIFICATION REACTIONS, (129) (GO) UBIQUITIN LIGASE COMPLEX, (130) (GO) REGULATION OF MRNA CATABOLIC PROCESS, (131) (GO) ANTIGEN PROCESSING AND PRESENTATION OF EXOGENOUS PEPTIDE ANTIGEN VIA MHC CLASS I, (132) (GO) RIBONUCLEOPROTEIN COMPLEX BINDING, (133) (GO) NUCLEOTIDE EXCISION REPAIR, (134) (GO) PROTEIN CONTAINING COMPLEX LOCALIZATION, (135) (GO) RNA CATABOLIC PROCESS, (136) (GO) NUCLEAR ENVELOPE, (137) (GO) DRUG BINDING, (138) (GO) REGULATION OF CATABOLIC PROCESS, (139) (GO) ATPASE ACTIVITY, (140) (GO) ESTABLISHMENT OF ORGANELLE LOCALIZATION, (141) (GO) UBIQUITIN LIKE PROTEIN LIGASE BINDING, (142) (GO) MRNA PROCESSING, (143) (GO) SPLICEOSOMAL TRI SNRNP COMPLEX, (144) (GO) RNA LOCALIZATION, (145) (GO) REGULATION OF CELLULAR CATABOLIC PROCESS, (146) (GO) RNA SPLICING, (147) (GO) PRERIBOSOME	

Table S8. Jointly deregulated pathways in AD and CRCA.

	Up_CRCA	Down_CRCA
Up_AD	(1) (H)K EPITHELIAL MESENCHYMAL TRANSITION, (2) (R) COLLAGEN FORMATION, (3) (R) ASSEMBLY OF COLLAGEN FIBRILS AND OTHER MULTIMERIC STRUCTURES, (4) (GO) CHROMOSOME, (5) (GO) NUCLEAR CHROMOSOME, (6) (GO) CHROMATIN BINDING, (7) (GO) ENDOPLASMIC RETICULUM LUMEN, (8) (GO) REPRODUCTION	(1) (R) IMMUNOREGULATORY INTERACTIONS BETWEEN A LYMPHOID AND A NON LYMPHOID CELL, (2) (K) HEMATOPOIETIC CELL LINEAGE, (3) (K) CELL ADHESION MOLECULES CAMS, (4) (GO) ADAPTIVE IMMUNE RESPONSE, (5) (GO) BIOLOGICAL ADHESION, (6) (GO) CELL ACTIVATION, (7) (GO) CELL CELL ADHESION, (8) (GO) CELL SURFACE, (9)

		(GO) EXTERNAL SIDE OF PLASMA MEMBRANE, (10) (GO) INFLAMMATORY RESPONSE, (11) (GO) INTRINSIC COMPONENT OF PLASMA MEMBRANE, (12) (GO) LEUKOCYTE CELL CELL ADHESION, (13) (GO) LEUKOCYTE MIGRATION, (14) (GO) LEUKOCYTE PROLIFERATION, (15) (GO) LYMPHOCYTE ACTIVATION, (16) (GO) MOLECULAR TRANSDUCER ACTIVITY, (17) (GO) NEGATIVE REGULATION OF IMMUNE SYSTEM PROCESS, (18) (GO) POSITIVE REGULATION OF IMMUNE SYSTEM PROCESS, (19) (GO) REGULATION OF CELL ADHESION, (20) (GO) REGULATION OF IMMUNE SYSTEM PROCESS, (21) (GO) T CELL ACTIVATION, (22) (GO) REGULATION OF T CELL ACTIVATION, (23) (GO) REGULATION OF IMMUNE RESPONSE, (24) (GO) RECEPTOR COMPLEX, (25) (GO) SIDE OF MEMBRANE, (26) (GO) POSITIVE REGULATION OF CELL DIFFERENTIATION, (27) (GO) POSITIVE REGULATION OF IMMUNE RESPONSE, (28) (GO) REGULATION OF RESPONSE TO EXTERNAL STIMULUS, (29) (GO) B CELL RECEPTOR SIGNALING PATHWAY, (30) (GO) ACTIVATION OF IMMUNE RESPONSE, (31) (GO) MUSCLE SYSTEM PROCESS
**Down_AD**	(1) (H)K MYC TARGETS V1, (2) (H)K MTORC1 SIGNALING, (3) (H)K DNA REPAIR, (4) (H)K UNFOLDED PROTEIN RESPONSE, (5) (K) PROTEASOME, (6) (R) APC C CDH1 MEDIATED DEGRADATION OF CDC20 AND OTHER APC C CDH1 TARGETED PROTEINS IN LATE MITOSIS EARLY G1, (7) (R) ASSEMBLY OF THE PRE REPLICATIVE COMPLEX, (8) (R) AUF1 HNRNP D0 BINDS AND DESTABILIZES MRNA, (9) (R) CELL CYCLE CHECKPOINTS, (10) (R) CROSS PRESENTATION OF SOLUBLE EXOGENOUS ANTIGENS ENDOSOMES, (11) (R) DEGRADATION OF DVL, (12) (R) DNA REPLICATION, (13) (R) HIV INFECTION, (14) (R) HOST INTERACTIONS OF HIV FACTORS, (15) (R) METABOLISM OF RNA, (16) (R) MITOCHONDRIAL TRANSLATION, (17) (R) MITOTIC G2 G2 M PHASES, (18) (R) MITOTIC METAPHASE AND ANAPHASE, (19) (R) NEGATIVE REGULATION OF NOTCH4 SIGNALING, (20) (R) ORC1 REMOVAL FROM CHROMATIN, (21) (R) REGULATION OF PTEN STABILITY AND ACTIVITY, (22) (R) SEPARATION OF SISTER CHROMATIDS, (23) (R) SWITCHING OF ORIGINS TO A POST REPLICATIVE STATE, (24) (R) TRANSLATION, (25) (R) DEGRADATION OF AXIN, (26) (R) METABOLISM OF POLYAMINES, (27) (R) THE ROLE OF GTSE1 IN G2 M PROGRESSION AFTER G2 CHECKPOINT, (28) (R) APC C MEDIATED DEGRADATION OF CELL CYCLE PROTEINS, (29) (R) DNA REPLICATION PRE INITIATION, (30) (R) STABILIZATION OF P53, (31) (R) SCF SKP2 MEDIATED DEGRADATION OF P27 P21, (32) (R) REGULATION OF RUNX3 EXPRESSION AND ACTIVITY, (33) (R) S PHASE, (34) (R) REGULATION OF EXPRESSION OF SLITS AND ROBOS, (35) (R) G2 M CHECKPOINTS, (36) (R) DECTIN 1 MEDIATED NONCANONICAL NF KB SIGNALING, (37) (R) SIGNALING	(1) (H)K OXIDATIVE PHOSPHORYLATION, (2) (H)K FATTY ACID METABOLISM, (3) (K) ALZHEIMERS DISEASE, (4) (K) HUNTINGTONS DISEASE, (5) (K) OXIDATIVE PHOSPHORYLATION, (6) (K) PARKINSONS DISEASE, (7) (R) RESPIRATORY ELECTRON TRANSPORT, (8) (R) RESPIRATORY ELECTRON TRANSPORT ATP SYNTHESIS BY CHEMIOSMOTIC COUPLING AND HEAT PRODUCTION BY UNCOUPLING PROTEINS, (9) (R) THE CITRIC ACID TCA CYCLE AND RESPIRATORY ELECTRON TRANSPORT, (10) (R) NEURONAL SYSTEM, (11) (R) TRANSMISSION ACROSS CHEMICAL SYNAPSES, (12) (R) PYRUVATE METABOLISM AND CITRIC ACID TCA CYCLE, (13) (K) CARDIAC MUSCLE CONTRACTION, (14) (R) NEUROTRANSMITTER RECEPTORS AND POSTSYNAPTIC SIGNAL TRANSMISSION, (15) (GO) ATP SYNTHESIS COUPLED ELECTRON TRANSPORT, (16) (GO) MEMBRANE PROTEIN COMPLEX, (17) (GO) NEURON PROJECTION, (18) (GO) POSTSYNAPSE, (19) (GO) RESPIRASOME, (20) (GO) SOMATODENDRITIC COMPARTMENT, (21) (GO) SYNAPSE, (22) (GO) SYNAPTIC SIGNALING, (23) (GO) DENDRITIC TREE,

	BY ROBO RECEPTORS, (38) (R) REGULATION OF MRNA STABILITY BY PROTEINS THAT BIND AU RICH ELEMENTS, (39) (R) NUCLEOTIDE EXCISION REPAIR, (40) (R) CYCLIN A CDK2 ASSOCIATED EVENTS AT S PHASE ENTRY, (41) (R) PROCESSING OF CAPPED INTRON CONTAINING PRE MRNA, (42) (R) G1 S DNA DAMAGE CHECKPOINTS, (43) (R) FORMATION OF TC NER PRE INCISION COMPLEX, (44) (R) CELL CYCLE, (45) (R) M PHASE, (46) (R) TRANSCRIPTION COUPLED NUCLEOTIDE EXCISION REPAIR TC NER, (47) (R) TRNA AMINOACYLATION, (48) (R) UCH PROTEINASES, (49) (R) HIV LIFE CYCLE, (50) (R) CELL CYCLE MITOTIC, (51) (R) PTEN REGULATION, (52) (R) CELLULAR RESPONSES TO EXTERNAL STIMULI, (53) (K) AMINOACYL TRNA BIOSYNTHESIS, (54) (R) MRNA SPLICING, (55) (R) GLOBAL GENOME NUCLEOTIDE EXCISION REPAIR GG NER, (56) (R) MITOTIC G1 PHASE AND G1 S TRANSITION, (57) (R) COOPERATION OF PREFOLDIN AND TRIC CCT IN ACTIN AND TUBULIN FOLDING, (58) (K) RNA DEGRADATION, (59) (K) UBIQUITIN MEDIATED PROTEOLYSIS, (60) (R) TRNA PROCESSING, (61) (R) MRNA SPLICING MINOR PATHWAY, (62) (R) RRNA PROCESSING, (63) (R) CILIUM ASSEMBLY, (64) (R) FORMATION OF INCISION COMPLEX IN GG NER, (65) (R) VIRAL MESSENGER RNA SYNTHESIS, (66) (R) RRNA MODIFICATION IN THE NUCLEUS AND CYTOSOL, (67) (R) DUAL INCISION IN TC NER, (68) (GO) AMIDE BIOSYNTHETIC PROCESS, (69) (GO) ANAPHASE PROMOTING COMPLEX DEPENDENT CATABOLIC PROCESS, (70) (GO) CATALYTIC ACTIVITY ACTING ON RNA, (71) (GO) CATALYTIC COMPLEX, (72) (GO) CELLULAR AMIDE METABOLIC PROCESS, (73) (GO) CELLULAR MACROMOLECULE CATABOLIC PROCESS, (74) (GO) CELLULAR PROTEIN CONTAINING COMPLEX ASSEMBLY, (75) (GO) ENDOPEPTIDASE COMPLEX, (76) (GO) HYDROLASE ACTIVITY ACTING ON ACID ANHYDRIDES, (77) (GO) MITOCHONDRIAL GENE EXPRESSION, (78) (GO) MITOCHONDRIAL MATRIX, (79) (GO) MITOCHONDRIAL TRANSLATION, (80) (GO) MITOCHONDRIAL TRANSLATIONAL TERMINATION, (81) (GO) NCRNA METABOLIC PROCESS, (82) (GO) NCRNA PROCESSING, (83) (GO) ORGANELLAR RIBOSOME, (84) (GO) ORGANONITROGEN COMPOUND BIOSYNTHETIC PROCESS, (85) (GO) PEPTIDASE COMPLEX, (86) (GO) PEPTIDE BIOSYNTHETIC PROCESS, (87) (GO) PEPTIDE METABOLIC PROCESS, (88) (GO) PROTEIN CONTAINING COMPLEX ASSEMBLY, (89) (GO) PROTEIN MODIFICATION BY SMALL PROTEIN CONJUGATION, (90) (GO) PROTEIN MODIFICATION BY SMALL PROTEIN CONJUGATION OR REMOVAL, (91) (GO) RIBONUCLEOPROTEIN COMPLEX, (92) (GO) RIBONUCLEOPROTEIN COMPLEX BIOGENESIS, (93) (GO) RIBOSOME, (94) (GO) RIBOSOME BIOGENESIS, (95) (GO) RNA BINDING, (96) (GO) RNA PROCESSING, (97) (GO) TRANSLATIONAL ELONGATION, (98) (GO) TRANSLATIONAL TERMINATION, (99) (GO) TRNA METABOLIC PROCESS, (100) (GO) CELLULAR AMINO ACID METABOLIC PROCESS, (101) (GO) REGULATION OF CELLULAR AMINO ACID METABOLIC PROCESS, (102) (GO) RIBOSOMAL SUBUNIT, (103) (GO) RIBONUCLEOTIDE BINDING, (104) (GO) REGULATION OF CELL CYCLE G2 M PHASE TRANSITION, (105) (GO) CATALYTIC ACTIVITY ACTING ON A TRNA, (106) (GO) CELL CYCLE G2 M PHASE TRANSITION, (107) (GO) NUCLEOLUS, (108) (GO) NEGATIVE REGULATION OF CELL CYCLE G2 M PHASE TRANSITION, (109) (GO) PROTEIN LOCALIZATION TO ORGANELLE, (110)	(24) (GO) REGULATION OF TRANS SYNAPTIC SIGNALING, (25) (GO) REGULATION OF SYNAPTIC PLASTICITY, (26) (GO) CELL BODY, (27) (GO) POSTSYNAPTIC MEMBRANE, (28) (GO) MONOVALENT INORGANIC CATION TRANSPORT, (29) (GO) MONOVALENT INORGANIC CATION TRANSMEMBRANE TRANSPORTER ACTIVITY, (30) (GO) CATION TRANSMEMBRANE TRANSPORT, (31) (GO) TRANSMEMBRANE TRANSPORT, (32) (GO) INORGANIC ION TRANSMEMBRANE TRANSPORT, (33) (GO) ION TRANSMEMBRANE TRANSPORT, (34) (GO) GLUTAMATE RECEPTOR SIGNALING PATHWAY

	(GO) PROTEIN MODIFICATION BY SMALL PROTEIN REMOVAL, (111) (GO) POSTTRANSCRIPTIONAL REGULATION OF GENE EXPRESSION, (112) (GO) RRNA METABOLIC PROCESS, (113) (GO) STRUCTURAL CONSTITUENT OF RIBOSOME, (114) (GO) TRANSFERASE COMPLEX, (115) (GO) AMINO ACID ACTIVATION, (116) (GO) MRNA METABOLIC PROCESS, (117) (GO) RIBONUCLEOPROTEIN COMPLEX SUBUNIT ORGANIZATION, (118) (GO) LIGASE ACTIVITY, (119) (GO) PROTEIN FOLDING, (120) (GO) ORGANELLE LOCALIZATION, (121) (GO) TRANSCRIPTION COUPLED NUCLEOTIDE EXCISION REPAIR, (122) (GO) UNFOLDED PROTEIN BINDING, (123) (GO) LARGE RIBOSOMAL SUBUNIT, (124) (GO) MICROTUBULE, (125) (GO) MICROTUBULE CYTOSKELETON, (126) (GO) ADENYL NUCLEOTIDE BINDING, (127) (GO) LIGASE ACTIVITY FORMING CARBON OXYGEN BONDS, (128) (GO) MACROMOLECULE CATABOLIC PROCESS, (129) (GO) MICROTUBULE BASED PROCESS, (130) (GO) TRNA PROCESSING, (131) (GO) RNA SPLICING VIA TRANSESTERIFICATION REACTIONS, (132) (GO) REGULATION OF MRNA CATABOLIC PROCESS, (133) (GO) RIBONUCLEOPROTEIN COMPLEX BINDING, (134) (GO) NUCLEOTIDE EXCISION REPAIR, (135) (GO) PROTEIN CONTAINING COMPLEX LOCALIZATION, (136) (GO) TRANSLATION FACTOR ACTIVITY RNA BINDING, (137) (GO) RNA CATABOLIC PROCESS, (138) (GO) NUCLEAR ENVELOPE, (139) (GO) DRUG BINDING, (140) (GO) ATPASE ACTIVITY, (141) (GO) CYTOPLASMIC TRANSLATION, (142) (GO) TRNA BINDING, (143) (GO) MRNA PROCESSING, (144) (GO) SPLICEOSOMAL TRI SNRNP COMPLEX, (145) (GO) RNA LOCALIZATION, (146) (GO) RNA SPLICING, (147) (GO) PRERIBOSOME, (148) (GO) SMALL RIBOSOMAL SUBUNIT	

Table S9. Jointly deregulated pathways in AD and HANC.

	Up_HANC	Down_HANC
Up_AD	(1) (H)K INFLAMMATORY RESPONSE, (2) (H)K EPITHELIAL MESENCHYMAL TRANSITION, (3) (H)K IL6 JAK STAT3 SIGNALING, (4) (H)K TNFA SIGNALING VIA NFKB, (5) (R) EXTRACELLULAR MATRIX ORGANIZATION, (6) (R) CELL SURFACE INTERACTIONS AT THE VASCULAR WALL, (7) (R) COLLAGEN FORMATION, (8) (K) ECM RECEPTOR INTERACTION, (9) (R) ECM PROTEOGLYCANS, (10) (R) ASSEMBLY OF COLLAGEN FIBRILS AND OTHER MULTIMERIC STRUCTURES, (11) (K) PATHWAYS IN CANCER, (12) (R) NON INTEGRIN MEMBRANE ECM INTERACTIONS, (13) (PID) INTEGRIN1 PATHWAY, (14) (R) INTEGRIN CELL SURFACE INTERACTIONS, (15) (R) TRANSCRIPTIONAL REGULATION OF GRANULOPOIESIS, (16) (R) DEGRADATION OF THE EXTRACELLULAR MATRIX, (17) (R) COLLAGEN DEGRADATION, (18) (R) SIGNALING BY INTERLEUKINS, (19) (R) SYNDECAN INTERACTIONS, (20) (GO) ANIMAL ORGAN MORPHOGENESIS, (21) (GO) CHROMOSOME, (22) (GO) CYTOKINE MEDIATED SIGNALING PATHWAY, (23) (GO) DEFENSE RESPONSE, (24) (GO) EMBRYO DEVELOPMENT, (25) (GO) EXTRACELLULAR STRUCTURE ORGANIZATION, (26) (GO) IMMUNE SYSTEM DEVELOPMENT, (27) (GO) NUCLEAR CHROMOSOME, (28) (GO) POSITIVE REGULATION OF CELLULAR BIOSYNTHETIC PROCESS, (29) (GO) POSITIVE	(1) (R) KERATINIZATION, (2) (R) FORMATION OF THE CORNIFIED ENVELOPE, (3) (GO) KERATINIZATION, (4) (GO) CORNIFICATION

	REGULATION OF NUCLEOBASE CONTAINING COMPOUND METABOLIC PROCESS, (30) (GO) RESPONSE TO BIOTIC STIMULUS, (31) (GO) RESPONSE TO CYTOKINE, (32) (GO) EMBRYO DEVELOPMENT ENDING IN BIRTH OR EGG HATCHING, (33) (GO) DEFENSE RESPONSE TO OTHER ORGANISM, (34) (GO) TISSUE MORPHOGENESIS, (35) (GO) INNATE IMMUNE RESPONSE, (36) (GO) CELL ADHESION MEDIATED BY INTEGRIN, (37) (GO) IMMUNE EFFECTOR PROCESS, (38) (GO) MORPHOGENESIS OF AN EPITHELIUM, (39) (GO) BASEMENT MEMBRANE, (40) (GO) ENDOPLASMIC RETICULUM LUMEN, (41) (GO) REPRODUCTION, (42) (GO) REGULATION OF DEFENSE RESPONSE	
Down_AD	(1) (H)K MYC TARGETS V1, (2) (H)K MTORC1 SIGNALING, (3) (H)K DNA REPAIR, (4) (H)K UNFOLDED PROTEIN RESPONSE, (5) (K) PROTEASOME, (6) (R) ANTIGEN PROCESSING UBIQUITINATION PROTEASOME DEGRADATION, (7) (R) APC C CDH1 MEDIATED DEGRADATION OF CDC20 AND OTHER APC C CDH1 TARGETED PROTEINS IN LATE MITOSIS EARLY G1, (8) (R) ASSEMBLY OF THE PRE REPLICATIVE COMPLEX, (9) (R) AUF1 HNRNP D0 BINDS AND DESTABILIZES MRNA, (10) (R) CELL CYCLE CHECKPOINTS, (11) (R) CLASS I MHC MEDIATED ANTIGEN PROCESSING PRESENTATION, (12) (R) CROSS PRESENTATION OF SOLUBLE EXOGENOUS ANTIGENS ENDOSOMES, (13) (R) DEFECTIVE CFTR CAUSES CYSTIC FIBROSIS, (14) (R) DEGRADATION OF DVL, (15) (R) DEGRADATION OF GLI1 BY THE PROTEASOME, (16) (R) DNA REPLICATION, (17) (R) HEDGEHOG LIGAND BIOGENESIS, (18) (R) HIV INFECTION, (19) (R) HOST INTERACTIONS OF HIV FACTORS, (20) (R) METABOLISM OF RNA, (21) (R) MITOCHONDRIAL TRANSLATION, (22) (R) MITOTIC G2 G2 M PHASES, (23) (R) MITOTIC METAPHASE AND ANAPHASE, (24) (R) NEGATIVE REGULATION OF NOTCH4 SIGNALING, (25) (R) ORC1 REMOVAL FROM CHROMATIN, (26) (R) REGULATION OF PTEN STABILITY AND ACTIVITY, (27) (R) REGULATION OF RAS BY GAPS, (28) (R) REGULATION OF RUNX2 EXPRESSION AND ACTIVITY, (29) (R) SEPARATION OF SISTER CHROMATIDS, (30) (R) SWITCHING OF ORIGINS TO A POST REPLICATIVE STATE, (31) (R) DEGRADATION OF AXIN, (32) (R) METABOLISM OF POLYAMINES, (33) (R) POST TRANSLATIONAL PROTEIN MODIFICATION, (34) (R) THE ROLE OF GTSE1 IN G2 M PROGRESSION AFTER G2 CHECKPOINT, (35) (R) APC C MEDIATED DEGRADATION OF CELL CYCLE PROTEINS, (36) (R) CELLULAR RESPONSE TO HYPOXIA, (37) (R) DNA REPLICATION PRE INITIATION, (38) (R) STABILIZATION OF P53, (39) (R) SCF SKP2 MEDIATED DEGRADATION OF P27 P21, (40) (R) REGULATION OF RUNX3 EXPRESSION AND ACTIVITY, (41) (R) S PHASE, (42) (R) G2 M CHECKPOINTS, (43) (R) ASYMMETRIC LOCALIZATION OF PCP PROTEINS, (44) (R) DECTIN 1 MEDIATED NONCANONICAL NF KB SIGNALING, (45) (R) REGULATION OF MRNA STABILITY BY PROTEINS THAT BIND AU RICH ELEMENTS, (46) (R) ABC TRANSPORTER DISORDERS, (47) (R) ABC FAMILY PROTEINS MEDIATED TRANSPORT, (48) (R) HEDGEHOG OFF STATE, (49) (R) NUCLEOTIDE EXCISION REPAIR, (50) (R) FCERI MEDIATED NF KB ACTIVATION, (51) (R) CYCLIN A CDK2 ASSOCIATED EVENTS AT S PHASE ENTRY, (52) (R) DOWNSTREAM SIGNALING EVENTS OF B CELL RECEPTOR BCR, (53) (R) PROCESSING OF CAPPED INTRON CONTAINING PRE MRNA, (54) (R) G1 S DNA DAMAGE CHECKPOINTS, (55) (R) CELL CYCLE, (56) (R) PCP CE	(1) (GO) OXIDATION REDUCTION PROCESS, (2) (GO) OXIDOREDUCTASE ACTIVITY

	PATHWAY, (57) (R) M PHASE, (58) (R) UCH PROTEINASES, (59) (R) HIV LIFE CYCLE, (60) (R) MAPK6 MAPK4 SIGNALING, (61) (R) HEDGEHOG ON STATE, (62) (R) SIGNALING BY NOTCH4, (63) (R) CELL CYCLE MITOTIC, (64) (R) PTEN REGULATION, (65) (R) CELLULAR RESPONSES TO EXTERNAL STIMULI, (66) (R) MRNA SPLICING, (67) (R) MITOTIC G1 PHASE AND G1 S TRANSITION, (68) (R) DEGRADATION OF BETA CATENIN BY THE DESTRUCTION COMPLEX, (69) (R) UB SPECIFIC PROCESSING PROTEASES, (70) (R) FC EPSILON RECEPTOR FCERI SIGNALING, (71) (R) INFECTIOUS DISEASE, (72) (R) DEUBIQUITINATION, (73) (R) TRNA PROCESSING, (74) (R) RRNA PROCESSING, (75) (R) CILIUM ASSEMBLY, (76) (R) ANTIGEN PROCESSING CROSS PRESENTATION, (77) (R) VIRAL MESSENGER RNA SYNTHESIS, (78) (R) RRNA MODIFICATION IN THE NUCLEUS AND CYTOSOL, (79) (GO) ANAPHASE PROMOTING COMPLEX DEPENDENT CATABOLIC PROCESS, (80) (GO) CATALYTIC ACTIVITY ACTING ON RNA, (81) (GO) CATALYTIC COMPLEX, (82) (GO) CELLULAR MACROMOLECULE CATABOLIC PROCESS, (83) (GO) CELLULAR PROTEIN CATABOLIC PROCESS, (84) (GO) CELLULAR PROTEIN CONTAINING COMPLEX ASSEMBLY, (85) (GO) ENDOPEPTIDASE COMPLEX, (86) (GO) HYDROLASE ACTIVITY ACTING ON ACID ANHYDRIDES, (87) (GO) MITOCHONDRIAL GENE EXPRESSION, (88) (GO) MITOCHONDRIAL TRANSLATION, (89) (GO) NCRNA METABOLIC PROCESS, (90) (GO) NCRNA PROCESSING, (91) (GO) ORGANELLAR RIBOSOME, (92) (GO) ORGANONITROGEN COMPOUND BIOSYNTHETIC PROCESS, (93) (GO) PEPTIDASE COMPLEX, (94) (GO) PEPTIDE BIOSYNTHETIC PROCESS, (95) (GO) PROTEASOMAL PROTEIN CATABOLIC PROCESS, (96) (GO) PROTEIN CONTAINING COMPLEX ASSEMBLY, (97) (GO) PROTEIN MODIFICATION BY SMALL PROTEIN CONJUGATION, (98) (GO) PROTEIN MODIFICATION BY SMALL PROTEIN CONJUGATION OR REMOVAL, (99) (GO) RIBONUCLEOPROTEIN COMPLEX, (100) (GO) RIBONUCLEOPROTEIN COMPLEX BIOGENESIS, (101) (GO) RIBOSOME BIOGENESIS, (102) (GO) RNA BINDING, (103) (GO) RNA PROCESSING, (104) (GO) SCF DEPENDENT PROTEASOMAL UBIQUITIN DEPENDENT PROTEIN CATABOLIC PROCESS, (105) (GO) TRANSLATIONAL ELONGATION, (106) (GO) TRANSLATIONAL TERMINATION, (107) (GO) TRNA METABOLIC PROCESS, (108) (GO) REGULATION OF CELLULAR AMINO ACID METABOLIC PROCESS, (109) (GO) RIBONUCLEOTIDE BINDING, (110) (GO) REGULATION OF CELL CYCLE G2 M PHASE TRANSITION, (111) (GO) CELL CYCLE G2 M PHASE TRANSITION, (112) (GO) NUCLEOLUS, (113) (GO) NEGATIVE REGULATION OF CELL CYCLE G2 M PHASE TRANSITION, (114) (GO) PROTEIN MODIFICATION BY SMALL PROTEIN REMOVAL, (115) (GO) POSTTRANSCRIPTIONAL REGULATION OF GENE EXPRESSION, (116) (GO) RRNA METABOLIC PROCESS, (117) (GO) REGULATION OF CELLULAR AMINE METABOLIC PROCESS, (118) (GO) MITOCHONDRIAL LARGE RIBOSOMAL SUBUNIT, (119) (GO) TRANSFERASE COMPLEX, (120) (GO) MRNA METABOLIC PROCESS, (121) (GO) RIBONUCLEOPROTEIN COMPLEX SUBUNIT ORGANIZATION, (122) (GO) ORGANELLE LOCALIZATION, (123) (GO) REGULATION OF TRANSCRIPTION FROM RNA POLYMERASE II PROMOTER IN RESPONSE TO HYPOXIA, (124) (GO) MICROTUBULE, (125) (GO) MICROTUBULE CYTOSKELETON, (126) (GO) ADENYL	

	NUCLEOTIDE BINDING, (127) (GO) ANTIGEN PROCESSING AND PRESENTATION OF PEPTIDE ANTIGEN, (128) (GO) MACROMOLECULE CATABOLIC PROCESS, (129) (GO) MICROTUBULE BASED PROCESS, (130) (GO) RNA SPLICING VIA TRANSESTERIFICATION REACTIONS, (131) (GO) REGULATION OF MRNA CATABOLIC PROCESS, (132) (GO) ANTIGEN PROCESSING AND PRESENTATION OF EXOGENOUS PEPTIDE ANTIGEN VIA MHC CLASS I, (133) (GO) RIBONUCLEOPROTEIN COMPLEX BINDING, (134) (GO) RNA CATABOLIC PROCESS, (135) (GO) CELLULAR COMPONENT DISASSEMBLY, (136) (GO) FC EPSILON RECEPTOR SIGNALING PATHWAY, (137) (GO) AMINE METABOLIC PROCESS, (138) (GO) NUCLEAR ENVELOPE, (139) (GO) DRUG BINDING, (140) (GO) REGULATION OF CATABOLIC PROCESS, (141) (GO) ATPASE ACTIVITY, (142) (GO) MRNA PROCESSING, (143) (GO) RNA LOCALIZATION, (144) (GO) INTERLEUKIN 1 MEDIATED SIGNALING PATHWAY, (145) (GO) REGULATION OF CELLULAR CATABOLIC PROCESS, (146) (GO) RNA SPLICING, (147) (GO) ANTIGEN PROCESSING AND PRESENTATION, (148) (GO) POST TRANSLATIONAL PROTEIN MODIFICATION, (149) (GO) PRERIBOSOME	

Table S10. Jointly deregulated pathways in AD and LGCA.

	Up_LGCA	Down_LGCA
Up_AD	(1) (GO) CHROMOSOME, (2) (GO) KERATINIZATION	(1) (H)K INFLAMMATORY RESPONSE, (2) (H)K MYOGENESIS, (3) (H)K TNFA SIGNALING VIA NFKB, (4) (H)K IL2 STAT5 SIGNALING, (5) (K) CYTOKINE CYTOKINE RECEPTOR INTERACTION, (6) (R) IMMUNOREGULATORY INTERACTIONS BETWEEN A LYMPHOID AND A NON LYMPHOID CELL, (7) (R) SIGNALING BY INTERLEUKINS, (8) (PID) ANGIOPOIETIN RECEPTOR PATHWAY, (9) (GO) ADAPTIVE IMMUNE RESPONSE, (10) (GO) ALPHA BETA T CELL ACTIVATION, (11) (GO) ANATOMICAL STRUCTURE FORMATION INVOLVED IN MORPHOGENESIS, (12) (GO) BIOLOGICAL ADHESION, (13) (GO) BLOOD VESSEL MORPHOGENESIS, (14) (GO) CARDIOVASCULAR SYSTEM DEVELOPMENT, (15) (GO) CELL ACTIVATION, (16) (GO) CELL CELL ADHESION, (17) (GO) CELL MOTILITY, (18) (GO) CELL SURFACE, (19) (GO) CIRCULATORY SYSTEM DEVELOPMENT, (20) (GO) CYTOKINE MEDIATED SIGNALING PATHWAY, (21) (GO) CYTOKINE PRODUCTION, (22) (GO) DEFENSE RESPONSE, (23) (GO) EXTERNAL SIDE OF PLASMA MEMBRANE, (24) (GO) IMMUNE RECEPTOR ACTIVITY, (25) (GO) IMMUNE SYSTEM DEVELOPMENT, (26) (GO) INFLAMMATORY RESPONSE, (27) (GO) INTRINSIC COMPONENT OF PLASMA MEMBRANE, (28) (GO) LEUKOCYTE CELL CELL ADHESION, (29) (GO) LEUKOCYTE

		DIFFERENTIATION, (30) (GO) LEUKOCYTE MIGRATION, (31) (GO) LOCOMOTION, (32) (GO) LYMPHOCYTE ACTIVATION, (33) (GO) MOLECULAR TRANSDUCER ACTIVITY, (34) (GO) NEGATIVE REGULATION OF DEVELOPMENTAL PROCESS, (35) (GO) NEGATIVE REGULATION OF IMMUNE SYSTEM PROCESS, (36) (GO) NEGATIVE REGULATION OF MULTICELLULAR ORGANISMAL PROCESS, (37) (GO) POSITIVE REGULATION OF CELL POPULATION PROLIFERATION, (38) (GO) POSITIVE REGULATION OF DEVELOPMENTAL PROCESS, (39) (GO) POSITIVE REGULATION OF IMMUNE SYSTEM PROCESS, (40) (GO) POSITIVE REGULATION OF VASCULATURE DEVELOPMENT, (41) (GO) REGULATION OF CELL ACTIVATION, (42) (GO) REGULATION OF CELL ADHESION, (43) (GO) REGULATION OF CELL POPULATION PROLIFERATION, (44) (GO) REGULATION OF IMMUNE SYSTEM PROCESS, (45) (GO) REGULATION OF VASCULATURE DEVELOPMENT, (46) (GO) RESPONSE TO BACTERIUM, (47) (GO) RESPONSE TO BIOTIC STIMULUS, (48) (GO) RESPONSE TO LI(PID), (49) (GO) RESPONSE TO MOLECULE OF BACTERIAL ORIGIN, (50) (GO) RESPONSE TO WOUNDING, (51) (GO) TUBE DEVELOPMENT, (52) (GO) TUBE MORPHOGENESIS, (53) (GO) T CELL ACTIVATION, (54) (GO) WOUND HEALING, (55) (GO) POSITIVE REGULATION OF MULTICELLULAR ORGANISMAL PROCESS, (56) (GO) REGULATION OF LEUKOCYTE DIFFERENTIATION, (57) (GO) NEGATIVE REGULATION OF CYTOKINE PRODUCTION, (58) (GO) POSITIVE REGULATION OF CELL ACTIVATION, (59) (GO) RESPONSE TO CYTOKINE, (60) (GO) REGULATION OF CELL CELL ADHESION, (61) (GO) CYTOKINE BINDING, (62) (GO) MUSCLE STRUCTURE DEVELOPMENT, (63) (GO) POSITIVE REGULATION OF CELL ADHESION, (64) (GO) DEFENSE RESPONSE TO OTHER ORGANISM, (65) (GO) REGULATION OF IMMUNE RESPONSE, (66) (GO) HEART DEVELOPMENT, (67) (GO) REGULATION OF IMMUNE EFFECTOR PROCESS, (68) (GO) REGULATION OF CELL DIFFERENTIATION, (69) (GO) NEGATIVE REGULATION OF CELL DIFFERENTIATION, (70) (GO) POSITIVE REGULATION OF INTRACELLULAR SIGNAL TRANSDUCTION, (71) (GO) RECEPTOR COMPLEX, (72) (GO) TUMOR NECROSIS FACTOR SUPERFAMILY

		CYTOKINE PRODUCTION, (73) (GO) NEGATIVE REGULATION OF CELL ACTIVATION, (74) (GO) SIDE OF MEMBRANE, (75) (GO) CELLULAR RESPONSE TO BIOTIC STIMULUS, (76) (GO) MYELOID CELL DIFFERENTIATION, (77) (GO) ADAPTIVE IMMUNE RESPONSE BASED ON SOMATIC RECOMBINATION OF IMMUNE RECEPTORS BUILT FROM IMMUNOGLOBULIN SUPERFAMILY DOMAINS, (78) (GO) REGULATION OF CELLULAR COMPONENT MOVEMENT, (79) (GO) ALPHA BETA T CELL DIFFERENTIATION, (80) (GO) EPITHELIAL CELL APOPTOTIC PROCESS, (81) (GO) EPITHELIAL CELL PROLIFERATION, (82) (GO) POSITIVE REGULATION OF LOCOMOTION, (83) (GO) CELL SUBSTRATE ADHESION, (84) (GO) SIGNALING RECEPTOR BINDING, (85) (GO) POSITIVE REGULATION OF PHOSPHATIDYLINOSITOL 3 KINASE SIGNALING, (86) (GO) HEART MORPHOGENESIS, (87) (GO) INNATE IMMUNE RESPONSE, (88) (GO) RESPONSE TO GROWTH FACTOR, (89) (GO) CYTOKINE SECRETION, (90) (GO) CELL CHEMOTAXIS, (91) (GO) POSITIVE REGULATION OF MAPK CASCADE, (92) (GO) REGULATION OF PHA(GO)CYTOSIS, (93) (GO) POSITIVE REGULATION OF CELL DIFFERENTIATION, (94) (GO) REGULATION OF BODY FLUID LEVELS, (95) (GO) POSITIVE REGULATION OF IMMUNE RESPONSE, (96) (GO) ENDOTHELIAL CELL APOPTOTIC PROCESS, (97) (GO) POSITIVE REGULATION OF PHOSPHORUS METABOLIC PROCESS, (98) (GO) POSITIVE REGULATION OF SIGNALING, (99) (GO) REGULATION OF INFLAMMATORY RESPONSE, (100) (GO) IMMUNE EFFECTOR PROCESS, (101) (GO) LYMPHOCYTE MEDIATED IMMUNITY, (102) (GO) ERK1 AND ERK2 CASCADE, (103) (GO) REGULATION OF LEUKOCYTE MEDIATED IMMUNITY, (104) (GO) CELLULAR RESPONSE TO LI(PID), (105) (GO) COAGULATION, (106) (GO) REGULATION OF RESPONSE TO EXTERNAL STIMULUS, (107) (GO) GRANULOCYTE MIGRATION, (108) (GO) POSITIVE REGULATION OF HEMOPOIESIS, (109) (GO) POSITIVE REGULATION OF LEUKOCYTE DIFFERENTIATION, (110) (GO) NEGATIVE REGULATION OF CELL CELL ADHESION, (111) (GO) POSITIVE REGULATION OF EPITHELIAL CELL PROLIFERATION, (112) (GO) REGULATION OF EPITHELIAL CELL APOPTOTIC PROCESS, (113) (GO)

		ENDOTHELIUM DEVELOPMENT, (114) (GO) ANCHORING JUNCTION, (115) (GO) NEGATIVE REGULATION OF CELL POPULATION PROLIFERATION, (116) (GO) TAXIS, (117) (GO) REGULATION OF ALPHA BETA T CELL ACTIVATION, (118) (GO) POSITIVE REGULATION OF PHA(GO)CYTOSIS, (119) (GO) NEGATIVE REGULATION OF CELL ADHESION, (120) (GO) POSITIVE REGULATION OF PROTEIN MODIFICATION PROCESS, (121) (GO) PEPTIDYL TYROSINE MODIFICATION, (122) (GO) PHA(GO)CYTOSIS, (123) (GO) REGULATION OF FAT CELL DIFFERENTIATION, (124) (GO) CARDIAC CHAMBER MORPHOGENESIS, (125) (GO) REGULATION OF CELLULAR RESPONSE TO GROWTH FACTOR STIMULUS, (126) (GO) CELLULAR RESPONSE TO VASCULAR ENDOTHELIAL GROWTH FACTOR STIMULUS, (127) (GO) REGULATION OF ANATOMICAL STRUCTURE MORPHOGENESIS, (128) (GO) MYELOID LEUKOCYTE MIGRATION, (129) (GO) POSITIVE REGULATION OF ERK1 AND ERK2 CASCADE, (130) (GO) CD4 POSITIVE ALPHA BETA T CELL ACTIVATION, (131) (GO) REGULATION OF ADAPTIVE IMMUNE RESPONSE, (132) (GO) ACTIVATION OF IMMUNE RESPONSE, (133) (GO) MUSCLE SYSTEM PROCESS, (134) (GO) REGULATION OF DEFENSE RESPONSE
**Down_AD**	(1) (H)K MYC TARGETS V1, (2) (H)K MTORC1 SIGNALING, (3) (H)K DNA REPAIR, (4) (R) APC C CDH1 MEDIATED DEGRADATION OF CDC20 AND OTHER APC C CDH1 TARGETED PROTEINS IN LATE MITOSIS EARLY G1, (5) (R) ASSEMBLY OF THE PRE REPLICATIVE COMPLEX, (6) (R) CELL CYCLE CHECKPOINTS, (7) (R) DNA REPLICATION, (8) (R) HIV INFECTION, (9) (R) METABOLISM OF RNA, (10) (R) MITOCHONDRIAL PROTEIN IMPORT, (11) (R) MITOCHONDRIAL TRANSLATION, (12) (R) MITOTIC G2 G2 M PHASES, (13) (R) MITOTIC METAPHASE AND ANAPHASE, (14) (R) ORC1 REMOVAL FROM CHROMATIN, (15) (R) SEPARATION OF SISTER CHROMATIDS, (16) (R) SWITCHING OF ORIGINS TO A POST REPLICATIVE STATE, (17) (R) THE CITRIC ACID TCA CYCLE AND RESPIRATORY ELECTRON TRANSPORT, (18) (R) TRANSLATION, (19) (R) METABOLISM OF POLYAMINES, (20) (R) THE ROLE OF GTSE1 IN G2 M PROGRESSION AFTER G2 CHECKPOINT, (21) (R) APC C MEDIATED DEGRADATION OF CELL CYCLE PROTEINS, (22) (R) DNA REPLICATION PRE INITIATION, (23) (R) STABILIZATION OF P53, (24) (R) SCF SKP2 MEDIATED DEGRADATION OF P27 P21, (25) (R) S PHASE, (26) (R) G2 M CHECKPOINTS, (27) (R) NUCLEOTIDE EXCISION REPAIR, (28) (R) PROCESSING OF CAPPED INTRON CONTAINING PRE MRNA, (29) (R) G1 S DNA DAMAGE CHECKPOINTS, (30) (R) CELL CYCLE, (31) (R) M PHASE, (32) (R) TRNA AMINOACYLATION, (33) (R) CELL CYCLE MITOTIC, (34) (R) (GO)LGI TO ER RETROGRADE	

	TRANSPORT, (35) (K) AMINOACYL TRNA BIOSYNTHESIS, (36) (R) MITOTIC G1 PHASE AND G1 S TRANSITION, (37) (R) METABOLISM OF AMINO ACIDS AND DERIVATIVES, (38) (R) TRNA PROCESSING, (39) (R) RRNA PROCESSING, (40) (GO) AMIDE BIOSYNTHETIC PROCESS, (41) (GO) ANAPHASE PROMOTING COMPLEX DEPENDENT CATABOLIC PROCESS, (42) (GO) CATALYTIC ACTIVITY ACTING ON RNA, (43) (GO) CELLULAR AMIDE METABOLIC PROCESS, (44) (GO) ENDOPEPTIDASE COMPLEX, (45) (GO) ENVELOPE, (46) (GO) MITOCHONDRIAL ENVELOPE, (47) (GO) MITOCHONDRIAL GENE EXPRESSION, (48) (GO) MITOCHONDRIAL MATRIX, (49) (GO) MITOCHONDRIAL PROTEIN COMPLEX, (50) (GO) MITOCHONDRIAL TRANSLATION, (51) (GO) MITOCHONDRIAL TRANSLATIONAL TERMINATION, (52) (GO) MITOCHONDRION, (53) (GO) NCRNA METABOLIC PROCESS, (54) (GO) NCRNA PROCESSING, (55) (GO) ORGANELLAR RIBOSOME, (56) (GO) ORGANELLE INNER MEMBRANE, (57) (GO) ORGANONITROGEN COMPOUND BIOSYNTHETIC PROCESS, (58) (GO) PEPTIDE BIOSYNTHETIC PROCESS, (59) (GO) PEPTIDE METABOLIC PROCESS, (60) (GO) RIBONUCLEOPROTEIN COMPLEX, (61) (GO) RIBONUCLEOPROTEIN COMPLEX BIOGENESIS, (62) (GO) RIBOSOME, (63) (GO) RIBOSOME BIOGENESIS, (64) (GO) RNA BINDING, (65) (GO) RNA PROCESSING, (66) (GO) TRANSLATIONAL ELONGATION, (67) (GO) TRANSLATIONAL TERMINATION, (68) (GO) TRNA METABOLIC PROCESS, (69) (GO) CELLULAR AMINO ACID METABOLIC PROCESS, (70) (GO) RIBOSOMAL SUBUNIT, (71) (GO) REGULATION OF CELL CYCLE G2 M PHASE TRANSITION, (72) (GO) CATALYTIC ACTIVITY ACTING ON A TRNA, (73) (GO) CELL CYCLE G2 M PHASE TRANSITION, (74) (GO) NUCLEOLUS, (75) (GO) NEGATIVE REGULATION OF CELL CYCLE G2 M PHASE TRANSITION, (76) (GO) RRNA METABOLIC PROCESS, (77) (GO) STRUCTURAL CONSTITUENT OF RIBOSOME, (78) (GO) MITOCHONDRIAL LARGE RIBOSOMAL SUBUNIT, (79) (GO) AMINO ACID ACTIVATION, (80) (GO) LIGASE ACTIVITY, (81) (GO) LARGE RIBOSOMAL SUBUNIT, (82) (GO) LIGASE ACTIVITY FORMING CARBON OXYGEN BONDS, (83) (GO) TRNA PROCESSING, (84) (GO) ORGANELLE ENVELOPE LUMEN, (85) (GO) RIBONUCLEOPROTEIN COMPLEX BINDING, (86) (GO) DRUG BINDING, (87) (GO) ATPASE ACTIVITY, (88) (GO) PRERIBOSOME	

Table S11. Jointly deregulated pathways in AD and LIVCA.

	Up_LIVCA	Down_LIVCA
Up_AD	(1) (GO) CHROMOSOME, (2) (GO) NUCLEAR CHROMOSOME, (3) (GO) CHROMATIN BINDING	(1) (H)K INFLAMMATORY RESPONSE, (2) (H)K ALLOGRAFT REJECTION, (3) (H)K COAGULATION, (4) (H)K KRAS SIGNALING DN, (5) (K) CYTOKINE CYTOKINE RECEPTOR INTERACTION, (6) NABA CORE MATRISOME, (7) NABA MATRISOME, (8) NABA MATRISOME ASSOCIATED, (9) NABA SECRETED FACTORS, (10) (R) IMMUNOREGULATORY INTERACTIONS BETWEEN A LYMPHOID AND A NON LYMPHOID CELL, (11) (K) HEMATOPOIETIC CELL LINEAGE, (12) (R)

		CLASS A 1 RHODOPSIN LIKE RECEPTORS, (13) NABA ECM GLYCOPROTEINS, (14) (K) COMPLEMENT AND COAGULATION CASCADES, (15) (R) COMPLEMENT CASCADE, (16) (K) CELL ADHESION MOLECULES CAMS, (17) (R) GPCR LIGAND BINDING, (18) (R) CHEMOKINE RECEPTORS BIND CHEMOKINES, (19) (GO) ADAPTIVE IMMUNE RESPONSE, (20) (GO) BIOLOGICAL ADHESION, (21) (GO) CELL CELL ADHESION, (22) (GO) CELL MOTILITY, (23) (GO) CELL SURFACE, (24) (GO) COLLAGEN CONTAINING EXTRACELLULAR MATRIX, (25) (GO) DEFENSE RESPONSE, (26) (GO) EXTERNAL SIDE OF PLASMA MEMBRANE, (27) (GO) EXTRACELLULAR MATRIX, (28) (GO) EXTRACELLULAR MATRIX STRUCTURAL CONSTITUENT, (29) (GO) IMMUNE RECEPTOR ACTIVITY, (30) (GO) INFLAMMATORY RESPONSE, (31) (GO) INTRINSIC COMPONENT OF PLASMA MEMBRANE, (32) (GO) LEUKOCYTE CELL CELL ADHESION, (33) (GO) LEUKOCYTE MIGRATION, (34) (GO) LEUKOCYTE PROLIFERATION, (35) (GO) LOCOMOTION, (36) (GO) MOLECULAR TRANSDUCER ACTIVITY, (37) (GO) NEGATIVE REGULATION OF MULTICELLULAR ORGANISMAL PROCESS, (38) (GO) POSITIVE REGULATION OF IMMUNE SYSTEM PROCESS, (39) (GO) REGULATION OF CELL ACTIVATION, (40) (GO) REGULATION OF IMMUNE SYSTEM PROCESS, (41) (GO) REGULATION OF VASCULATURE DEVELOPMENT, (42) (GO) RESPONSE TO BACTERIUM, (43) (GO) RESPONSE TO BIOTIC STIMULUS, (44) (GO) RESPONSE TO LI(PID), (45) (GO) RESPONSE TO MOLECULE OF BACTERIAL ORIGIN, (46) (GO) RESPONSE TO WOUNDING, (47) (GO) POSITIVE REGULATION OF MULTICELLULAR ORGANISMAL PROCESS, (48) (GO) CYTOKINE RECEPTOR ACTIVITY, (49) (GO) REGULATION OF CELL CELL ADHESION, (50) (GO) HUMORAL IMMUNE RESPONSE, (51) (GO) REGULATION OF IMMUNE RESPONSE, (52) (GO) REGULATION OF IMMUNE EFFECTOR PROCESS, (53) (GO) RECEPTOR COMPLEX, (54) (GO) SIDE OF MEMBRANE, (55) (GO) G PROTEIN COUPLED RECEPTOR ACTIVITY, (56) (GO) ADAPTIVE IMMUNE RESPONSE BASED ON SOMATIC RECOMBINATION OF IMMUNE RECEPTORS BUILT FROM IMMUNOGLOBULIN SUPERFAMILY DOMAINS, (57) (GO) POSITIVE REGULATION OF CELL CELL ADHESION,

		(58) (GO) REGULATION OF BODY FLUID LEVELS, (59) (GO) IMMUNE EFFECTOR PROCESS, (60) (GO) LYMPHOCYTE MEDIATED IMMUNITY, (61) (GO) POSITIVE REGULATION OF T CELL PROLIFERATION, (62) (GO) REGULATION OF LEUKOCYTE MEDIATED IMMUNITY, (63) (GO) TAXIS, (64) (GO) PEPTIDE RECEPTOR ACTIVITY, (65) (GO) COMPLEMENT ACTIVATION, (66) (GO) REGULATION OF HUMORAL IMMUNE RESPONSE, (67) (GO) BLOOD MICROPARTICLE, (68) (GO) CELL KILLING, (69) (GO) SENSORY PERCEPTION OF CHEMICAL STIMULUS, (70) (GO) REGULATION OF CELL KILLING, (71) (GO) B CELL RECEPTOR SIGNALING PATHWAY, (72) (GO) B CELL MEDIATED IMMUNITY, (73) (GO) STEROL TRANSPORT, (74) (GO) COMPLEMENT ACTIVATION ALTERNATIVE PATHWAY
Down_AD	(1) (H)K MYC TARGETS V1, (2) (H)K MTORC1 SIGNALING, (3) (H)K PROTEIN SECRETION, (4) (H)K DNA REPAIR, (5) (H)K UNFOLDED PROTEIN RESPONSE, (6) (K) PROTEASOME, (7) (R) ANTIGEN PROCESSING UBIQUITINATION PROTEASOME DEGRADATION, (8) (R) APC C CDH1 MEDIATED DEGRADATION OF CDC20 AND OTHER APC C CDH1 TARGETED PROTEINS IN LATE MITOSIS EARLY G1, (9) (R) ASSEMBLY OF THE PRE REPLICATIVE COMPLEX, (10) (R) AUF1 HNRNP D0 BINDS AND DESTABILIZES MRNA, (11) (R) CELL CYCLE CHECKPOINTS, (12) (R) CLASS I MHC MEDIATED ANTIGEN PROCESSING PRESENTATION, (13) (R) DEFECTIVE CFTR CAUSES CYSTIC FIBROSIS, (14) (R) DEGRADATION OF DVL, (15) (R) DEGRADATION OF GLI1 BY THE PROTEASOME, (16) (R) DNA REPLICATION, (17) (R) HEDGEHOG LIGAND BIOGENESIS, (18) (R) HIV INFECTION, (19) (R) HOST INTERACTIONS OF HIV FACTORS, (20) (R) MEMBRANE TRAFFICKING, (21) (R) METABOLISM OF RNA, (22) (R) MITOCHONDRIAL TRANSLATION, (23) (R) MITOTIC G2 G2 M PHASES, (24) (R) MITOTIC METAPHASE AND ANAPHASE, (25) (R) NEDDYLATION, (26) (R) NEGATIVE REGULATION OF NOTCH4 SIGNALING, (27) (R) ORC1 REMOVAL FROM CHROMATIN, (28) (R) REGULATION OF PTEN STABILITY AND ACTIVITY, (29) (R) REGULATION OF RAS BY GAPS, (30) (R) REGULATION OF RUNX2 EXPRESSION AND ACTIVITY, (31) (R) SEPARATION OF SISTER CHROMATIDS, (32) (R) SWITCHING OF ORIGINS TO A POST REPLICATIVE STATE, (33) (R) TRANSLATION, (34) (R) DEGRADATION OF AXIN, (35) (R) METABOLISM OF POLYAMINES, (36) (R) POST TRANSLATIONAL PROTEIN MODIFICATION, (37) (R) THE ROLE OF GTSE1 IN G2 M PROGRESSION AFTER G2 CHECKPOINT, (38) (R) APC C MEDIATED DEGRADATION OF CELL CYCLE PROTEINS, (39) (R) CELLULAR RESPONSE TO HYPOXIA, (40) (R) DNA REPLICATION PRE INITIATION, (41) (R) STABILIZATION OF P53, (42) (R) ORGANELLE BIOGENESIS AND MAINTENANCE, (43) (R) SCF SKP2 MEDIATED DEGRADATION OF P27 P21, (44) (R) REGULATION OF RUNX3 EXPRESSION AND ACTIVITY, (45) (R) S PHASE, (46) (R) REGULATION OF EXPRESSION OF SLITS AND ROBOS, (47) (R) G2 M CHECKPOINTS, (48) (R) ASYMMETRIC	(1) (H)K FATTY ACID METABOLISM, (2) (R) NEURONAL SYSTEM, (3) (R) METABOLISM OF AMINO ACIDS AND DERIVATIVES, (4) (GO) MITOCHONDRIAL MATRIX, (5) (GO) SYNAPTIC SIGNALING, (6) (GO) CELLULAR AMINO ACID METABOLIC PROCESS, (7) (GO) POSTSYNAPTIC MEMBRANE, (8) (GO) OXIDATION REDUCTION PROCESS, (9) (GO) SMALL MOLECULE METABOLIC PROCESS, (10) (GO) COFACTOR BIOSYNTHETIC PROCESS, (11) (GO) MONOVALENT INORGANIC CATION TRANSMEMBRANE TRANSPORTER ACTIVITY, (12) (GO) CATION TRANSMEMBRANE TRANSPORT, (13) (GO) COENZYME BIOSYNTHETIC PROCESS, (14) (GO) OXIDOREDUCTASE ACTIVITY, (15) (GO) TRANSMEMBRANE TRANSPORT, (16) (GO) INORGANIC ION TRANSMEMBRANE TRANSPORT, (17) (GO) COENZYME METABOLIC PROCESS, (18) (GO) ION TRANSMEMBRANE TRANSPORT

	LOCALIZATION OF PCP PROTEINS, (49) (R) DECTIN 1 MEDIATED NONCANONICAL NF KB SIGNALING, (50) (R) SIGNALING BY ROBO RECEPTORS, (51) (R) REGULATION OF MRNA STABILITY BY PROTEINS THAT BIND AU RICH ELEMENTS, (52) (R) HEDGEHOG OFF STATE, (53) (R) NUCLEOTIDE EXCISION REPAIR, (54) (R) FCERI MEDIATED NF KB ACTIVATION, (55) (R) CYCLIN A CDK2 ASSOCIATED EVENTS AT S PHASE ENTRY, (56) (R) DOWNSTREAM SIGNALING EVENTS OF B CELL RECEPTOR BCR, (57) (R) PROCESSING OF CAPPED INTRON CONTAINING PRE MRNA, (58) (R) G1 S DNA DAMAGE CHECKPOINTS, (59) (R) FORMATION OF TC NER PRE INCISION COMPLEX, (60) (R) CELL CYCLE, (61) (R) PCP CE PATHWAY, (62) (R) M PHASE, (63) (R) TRANSCRIPTION COUPLED NUCLEOTIDE EXCISION REPAIR TC NER, (64) (R) TRNA AMINOACYLATION, (65) (R) UCH PROTEINASES, (66) (R) HIV LIFE CYCLE, (67) (R) MAPK6 MAPK4 SIGNALING, (68) (R) HEDGEHOG ON STATE, (69) (R) SIGNALING BY NOTCH4, (70) (R) CELL CYCLE MITOTIC, (71) (R) (GO)LGI TO ER RETROGRADE TRANSPORT, (72) (R) PTEN REGULATION, (73) (R) CELLULAR RESPONSES TO EXTERNAL STIMULI, (74) (K) AMINOACYL TRNA BIOSYNTHESIS, (75) (R) MRNA SPLICING, (76) (R) GLOBAL GENOME NUCLEOTIDE EXCISION REPAIR GG NER, (77) (R) MITOTIC G1 PHASE AND G1 S TRANSITION, (78) (R) DEGRADATION OF BETA CATENIN BY THE DESTRUCTION COMPLEX, (79) (R) UB SPECIFIC PROCESSING PROTEASES, (80) (R) FC EPSILON RECEPTOR FCERI SIGNALING, (81) (R) INFECTIOUS DISEASE, (82) (K) RNA DEGRADATION, (83) (K) UBIQUITIN MEDIATED PROTEOLYSIS, (84) (R) DEUBIQUITINATION, (85) (R) INTRA (GO)LGI AND RETROGRADE (GO)LGI TO ER TRAFFIC, (86) (R) TRNA PROCESSING, (87) (R) MRNA SPLICING MINOR PATHWAY, (88) (R) RRNA PROCESSING, (89) (R) CILIUM ASSEMBLY, (90) (R) VIRAL MESSENGER RNA SYNTHESIS, (91) (R) DUAL INCISION IN TC NER, (92) (GO) AMIDE BIOSYNTHETIC PROCESS, (93) (GO) ANAPHASE PROMOTING COMPLEX DEPENDENT CATABOLIC PROCESS, (94) (GO) CATALYTIC ACTIVITY ACTING ON RNA, (95) (GO) CATALYTIC COMPLEX, (96) (GO) CELLULAR AMIDE METABOLIC PROCESS, (97) (GO) CELLULAR MACROMOLECULE CATABOLIC PROCESS, (98) (GO) CELLULAR MACROMOLECULE LOCALIZATION, (99) (GO) CELLULAR PROTEIN CATABOLIC PROCESS, (100) (GO) CELLULAR PROTEIN CONTAINING COMPLEX ASSEMBLY, (101) (GO) ENDOPEPTIDASE COMPLEX, (102) (GO) ENVELOPE, (103) (GO) HYDROLASE ACTIVITY ACTING ON ACID ANHYDRIDES, (104) (GO) INTRACELLULAR PROTEIN TRANSPORT, (105) (GO) INTRACELLULAR TRANSPORT, (106) (GO) MACROAUTOPHAGY, (107) (GO) MITOCHONDRIAL GENE EXPRESSION, (108) (GO) MITOCHONDRIAL TRANSLATION, (109) (GO) MODIFICATION DEPENDENT MACROMOLECULE CATABOLIC PROCESS, (110) (GO) NCRNA METABOLIC PROCESS, (111) (GO) NCRNA PROCESSING, (112) (GO) PEPTIDASE COMPLEX, (113) (GO) PEPTIDE BIOSYNTHETIC PROCESS, (114) (GO) PEPTIDE METABOLIC PROCESS, (115) (GO) PROTEASOMAL PROTEIN CATABOLIC PROCESS, (116) (GO) PROTEIN CONTAINING COMPLEX ASSEMBLY, (117) (GO) PROTEIN MODIFICATION BY SMALL PROTEIN CONJUGATION, (118) (GO) PROTEIN MODIFICATION BY SMALL PROTEIN CONJUGATION OR REMOVAL, (119) (GO) RIBONUCLEOPROTEIN COMPLEX, (120) (GO) RIBONUCLEOPROTEIN COMPLEX BIOGENESIS, (121) (GO)	

	RIBOSOME, (122) (GO) RIBOSOME BIOGENESIS, (123) (GO) RNA BINDING, (124) (GO) RNA PROCESSING, (125) (GO) SCF DEPENDENT PROTEASOMAL UBIQUITIN DEPENDENT PROTEIN CATABOLIC PROCESS, (126) (GO) TRANSLATIONAL ELONGATION, (127) (GO) TRANSLATIONAL TERMINATION, (128) (GO) TRNA METABOLIC PROCESS, (129) (GO) CELLULAR PROTEIN COMPLEX DISASSEMBLY, (130) (GO) REGULATION OF CELLULAR AMINO ACID METABOLIC PROCESS, (131) (GO) RIBOSOMAL SUBUNIT, (132) (GO) RIBONUCLEOTIDE BINDING, (133) (GO) REGULATION OF CELL CYCLE G2 M PHASE TRANSITION, (134) (GO) CATALYTIC ACTIVITY ACTING ON A TRNA, (135) (GO) CELL CYCLE G2 M PHASE TRANSITION, (136) (GO) PROTEIN POLYUBIQUITINATION, (137) (GO) PROTEIN CATABOLIC PROCESS, (138) (GO) NUCLEOLUS, (139) (GO) NEGATIVE REGULATION OF CELL CYCLE G2 M PHASE TRANSITION, (140) (GO) PROTEIN LOCALIZATION TO ORGANELLE, (141) (GO) PROTEIN MODIFICATION BY SMALL PROTEIN REMOVAL, (142) (GO) POSTTRANSCRIPTIONAL REGULATION OF GENE EXPRESSION, (143) (GO) RRNA METABOLIC PROCESS, (144) (GO) UBIQUITIN LIKE PROTEIN TRANSFERASE ACTIVITY, (145) (GO) STRUCTURAL CONSTITUENT OF RIBOSOME, (146) (GO) PROTEIN CONTAINING COMPLEX DISASSEMBLY, (147) (GO) TRANSFERASE COMPLEX, (148) (GO) AMINO ACID ACTIVATION, (149) (GO) MRNA METABOLIC PROCESS, (150) (GO) ESTABLISHMENT OF PROTEIN LOCALIZATION TO ORGANELLE, (151) (GO) RIBONUCLEOPROTEIN COMPLEX SUBUNIT ORGANIZATION, (152) (GO) ORGANELLE LOCALIZATION, (153) (GO) TRANSCRIPTION COUPLED NUCLEOTIDE EXCISION REPAIR, (154) (GO) LARGE RIBOSOMAL SUBUNIT, (155) (GO) REGULATION OF TRANSCRIPTION FROM RNA POLYMERASE II PROMOTER IN RESPONSE TO HYPOXIA, (156) (GO) MICROTUBULE, (157) (GO) MICROTUBULE CYTOSKELETON, (158) (GO) ADENYL NUCLEOTIDE BINDING, (159) (GO) ANTIGEN PROCESSING AND PRESENTATION OF PEPTIDE ANTIGEN, (160) (GO) MACROMOLECULE CATABOLIC PROCESS, (161) (GO) MICROTUBULE BASED PROCESS, (162) (GO) TRNA PROCESSING, (163) (GO) PROTEASOME ACCESSORY COMPLEX, (164) (GO) RNA SPLICING VIA TRANSESTERIFICATION REACTIONS, (165) (GO) UBIQUITIN LIGASE COMPLEX, (166) (GO) REGULATION OF MRNA CATABOLIC PROCESS, (167) (GO) RIBONUCLEOPROTEIN COMPLEX BINDING, (168) (GO) NUCLEOTIDE EXCISION REPAIR, (169) (GO) ENDOSOMAL TRANSPORT, (170) (GO) PROTEIN CONTAINING COMPLEX LOCALIZATION, (171) (GO) TRANSLATION FACTOR ACTIVITY RNA BINDING, (172) (GO) RNA CATABOLIC PROCESS, (173) (GO) CELLULAR COMPONENT DISASSEMBLY, (174) (GO) ESTABLISHMENT OF PROTEIN LOCALIZATION TO MEMBRANE, (175) (GO) NUCLEAR ENVELOPE, (176) (GO) REGULATION OF CATABOLIC PROCESS, (177) (GO) ATPASE ACTIVITY, (178) (GO) CYTOPLASMIC TRANSLATION, (179) (GO) ESTABLISHMENT OF ORGANELLE LOCALIZATION, (180) (GO) UBIQUITIN LIKE PROTEIN LIGASE BINDING, (181) (GO) MRNA PROCESSING, (182) (GO) SPLICEOSOMAL TRI SNRNP COMPLEX, (183) (GO) RNA LOCALIZATION, (184) (GO) INTERLEUKIN 1 MEDIATED SIGNALING PATHWAY, (185) (GO) REGULATION OF CELLULAR CATABOLIC PROCESS, (186) (GO) RNA SPLICING, (187) (GO)	

	TRANSLATION REGULATOR ACTIVITY NUCLEIC ACID BINDING, (188) (GO) PRERIBOSOME, (189) (GO) SMALL RIBOSOMAL SUBUNIT	

Table S12. Jointly deregulated pathways in AD and PACA.

	Up_PACA	Down_PACA
Up_AD	(1) (H)K INFLAMMATORY RESPONSE, (2) (H)K EPITHELIAL MESENCHYMAL TRANSITION, (3) (H)K TNFA SIGNALING VIA NFKB, (4) (H)K IL2 STAT5 SIGNALING, (5) (H)K HYPOXIA, (6) (R) EXTRACELLULAR MATRIX ORGANIZATION, (7) (R) CELL SURFACE INTERACTIONS AT THE VASCULAR WALL, (8) (R) COLLAGEN FORMATION, (9) (R) ASSEMBLY OF COLLAGEN FIBRILS AND OTHER MULTIMERIC STRUCTURES, (10) (K) PATHWAYS IN CANCER, (11) (R) NON INTEGRIN MEMBRANE ECM INTERACTIONS, (12) (PID) INTEGRIN1 PATHWAY, (13) (R) SIGNALING BY INTERLEUKINS, (14) (R) SYNDECAN INTERACTIONS, (15) (GO) BIOLOGICAL ADHESION, (16) (GO) CARDIOVASCULAR SYSTEM DEVELOPMENT, (17) (GO) CELL ACTIVATION, (18) (GO) CELL CELL ADHESION, (19) (GO) CELL MOTILITY, (20) (GO) CHROMOSOME, (21) (GO) CYTOKINE MEDIATED SIGNALING PATHWAY, (22) (GO) CYTOKINE PRODUCTION, (23) (GO) DEFENSE RESPONSE, (24) (GO) EMBRYO DEVELOPMENT, (25) (GO) E(PID)ERMIS DEVELOPMENT, (26) (GO) EPITHELIAL CELL DIFFERENTIATION, (27) (GO) EPITHELIUM DEVELOPMENT, (28) (GO) EXTRACELLULAR STRUCTURE ORGANIZATION, (29) (GO) IMMUNE SYSTEM DEVELOPMENT, (30) (GO) LEUKOCYTE CELL CELL ADHESION, (31) (GO) LEUKOCYTE MIGRATION, (32) (GO) LOCOMOTION, (33) (GO) LYMPHOCYTE ACTIVATION, (34) (GO) POSITIVE REGULATION OF IMMUNE SYSTEM PROCESS, (35) (GO) REGULATION OF CELL ACTIVATION, (36) (GO) REGULATION OF CELL ADHESION, (37) (GO) REGULATION OF CELL POPULATION PROLIFERATION, (38) (GO) REGULATION OF IMMUNE SYSTEM PROCESS, (39) (GO) REGULATION OF LYMPHOCYTE ACTIVATION, (40) (GO) RESPONSE TO BIOTIC STIMULUS, (41) (GO) RESPONSE TO WOUNDING, (42) (GO) SKIN DEVELOPMENT, (43) (GO) TUBE DEVELOPMENT, (44) (GO) TUBE MORPHOGENESIS, (45) (GO) T CELL ACTIVATION, (46) (GO) WOUND HEALING, (47) (GO) RESPONSE TO CYTOKINE, (48) (GO) REGULATION OF T CELL ACTIVATION, (49) (GO) REGULATION OF CELL CELL ADHESION, (50) (GO) POSITIVE REGULATION OF CELL ADHESION, (51) (GO) EMBRYO DEVELOPMENT ENDING IN BIRTH OR EGG HATCHING, (52) (GO) DEFENSE RESPONSE TO OTHER ORGANISM, (53) (GO) REGULATION OF IMMUNE RESPONSE, (54) (GO) POSITIVE REGULATION OF INTRACELLULAR SIGNAL TRANSDUCTION, (55) (GO) KERATINOCYTE DIFFERENTIATION, (56) (GO) TISSUE MORPHOGENESIS, (57) (GO) MYELOID CELL DIFFERENTIATION, (58) (GO) POSITIVE REGULATION OF LEUKOCYTE CELL CELL ADHESION, (59) (GO) REGULATION OF CELLULAR COMPONENT MOVEMENT, (60) (GO) POSITIVE REGULATION OF CELL CELL ADHESION, (61) (GO) EPITHELIAL CELL PROLIFERATION, (62) (GO) POSITIVE REGULATION OF LOCOMOTION, (63) (GO) PATTERN RECOGNITION RECEPTOR SIGNALING PATHWAY, (64) (GO) CELL SUBSTRATE ADHESION, (65) (GO) INNATE IMMUNE RESPONSE, (66) (GO) POSITIVE	(1) (H)K KRAS SIGNALING DN, (2) (R) CLASS A 1 RHODOPSIN LIKE RECEPTORS, (3) (R) GPCR LIGAND BINDING, (4) (K) MATURITY ONSET DIABETES OF THE YOUNG, (5) (GO) INTRINSIC COMPONENT OF PLASMA MEMBRANE, (6) (GO) MOLECULAR TRANSDUCER ACTIVITY, (7) (GO) G PROTEIN COUPLED RECEPTOR ACTIVITY, (8) (GO) RECEPTOR REGULATOR ACTIVITY, (9) (GO) SENSORY PERCEPTION OF LIGHT STIMULUS, (10) (GO) PEPTIDE RECEPTOR ACTIVITY, (11) (GO) SENSORY PERCEPTION OF CHEMICAL STIMULUS

	REGULATION OF IMMUNE RESPONSE, (67) (GO) POSITIVE REGULATION OF CYTOKINE PRODUCTION, (68) (GO) POSITIVE REGULATION OF SIGNALING, (69) (GO) CELL ADHESION MEDIATED BY INTEGRIN, (70) (GO) IMMUNE EFFECTOR PROCESS, (71) (GO) MORPHOGENESIS OF AN EPITHELIUM, (72) (GO) REGULATION OF RESPONSE TO EXTERNAL STIMULUS, (73) (GO) REGULATION OF HEMOPOIESIS, (74) (GO) ANCHORING JUNCTION, (75) (GO) POSITIVE REGULATION OF PROTEIN MODIFICATION PROCESS, (76) (GO) I KAPPAB KINASE NF KAPPAB SIGNALING, (77) (GO) PHA(GO)CYTOSIS, (78) (GO) REGULATION OF ANATOMICAL STRUCTURE MORPHOGENESIS, (79) (GO) INTEGRIN MEDIATED SIGNALING PATHWAY, (80) (GO) ACTIVATION OF IMMUNE RESPONSE, (81) (GO) REGULATION OF DEFENSE RESPONSE	
Down_AD	(1) (H)K MYC TARGETS V1, (2) (H)K MTORC1 SIGNALING, (3) (H)K PROTEIN SECRETION, (4) (H)K DNA REPAIR, (5) (K) PROTEASOME, (6) (R) ANTIGEN PROCESSING UBIQUITINATION PROTEASOME DEGRADATION, (7) (R) APC C CDH1 MEDIATED DEGRADATION OF CDC20 AND OTHER APC C CDH1 TARGETED PROTEINS IN LATE MITOSIS EARLY G1, (8) (R) ASSEMBLY OF THE PRE REPLICATIVE COMPLEX, (9) (R) AUF1 HNRNP D0 BINDS AND DESTABILIZES MRNA, (10) (R) CELL CYCLE CHECKPOINTS, (11) (R) CLASS I MHC MEDIATED ANTIGEN PROCESSING PRESENTATION, (12) (R) CROSS PRESENTATION OF SOLUBLE EXOGENOUS ANTIGENS ENDOSOMES, (13) (R) DEFECTIVE CFTR CAUSES CYSTIC FIBROSIS, (14) (R) DEGRADATION OF DVL, (15) (R) DEGRADATION OF GLI1 BY THE PROTEASOME, (16) (R) DNA REPLICATION, (17) (R) HEDGEHOG LIGAND BIOGENESIS, (18) (R) HIV INFECTION, (19) (R) HOST INTERACTIONS OF HIV FACTORS, (20) (R) MEMBRANE TRAFFICKING, (21) (R) METABOLISM OF RNA, (22) (R) MITOTIC G2 G2 M PHASES, (23) (R) MITOTIC METAPHASE AND ANAPHASE, (24) (R) NEGATIVE REGULATION OF NOTCH4 SIGNALING, (25) (R) ORC1 REMOVAL FROM CHROMATIN, (26) (R) REGULATION OF PTEN STABILITY AND ACTIVITY, (27) (R) REGULATION OF RAS BY GAPS, (28) (R) REGULATION OF RUNX2 EXPRESSION AND ACTIVITY, (29) (R) SEPARATION OF SISTER CHROMATIDS, (30) (R) SWITCHING OF ORIGINS TO A POST REPLICATIVE STATE, (31) (R) DEGRADATION OF AXIN, (32) (R) METABOLISM OF POLYAMINES, (33) (R) POST TRANSLATIONAL PROTEIN MODIFICATION, (34) (R) THE ROLE OF GTSE1 IN G2 M PROGRESSION AFTER G2 CHECKPOINT, (35) (R) APC C MEDIATED DEGRADATION OF CELL CYCLE PROTEINS, (36) (R) CELLULAR RESPONSE TO HYPOXIA, (37) (R) DNA REPLICATION PRE INITIATION, (38) (R) STABILIZATION OF P53, (39) (R) ORGANELLE BIOGENESIS AND MAINTENANCE, (40) (R) SCF SKP2 MEDIATED DEGRADATION OF P27 P21, (41) (R) VESICLE MEDIATED TRANSPORT, (42) (R) REGULATION OF RUNX3 EXPRESSION AND ACTIVITY, (43) (R) S PHASE, (44) (R) G2 M CHECKPOINTS, (45) (R) ASYMMETRIC LOCALIZATION OF PCP PROTEINS, (46) (R) DECTIN 1 MEDIATED NONCANONICAL NF KB SIGNALING, (47) (R) REGULATION OF MRNA STABILITY BY PROTEINS THAT BIND AU RICH ELEMENTS, (48) (R) ABC TRANSPORTER DISORDERS, (49) (R) ABC FAMILY PROTEINS MEDIATED TRANSPORT, (50) (R) HEDGEHOG OFF STATE, (51) (R) NUCLEOTIDE EXCISION	(1) (R) NEURONAL SYSTEM, (2) (R) TRANSMISSION ACROSS CHEMICAL SYNAPSES, (3) (GO) NEURON PROJECTION, (4) (GO) POSTSYNAPSE, (5) (GO) PRESYNAPSE, (6) (GO) SYNAPSE, (7) (GO) SYNAPTIC MEMBRANE, (8) (GO) SYNAPTIC SIGNALING, (9) (GO) REGULATION OF TRANS SYNAPTIC SIGNALING, (10) (GO) REGULATION OF SYNAPTIC PLASTICITY, (11) (GO) POSTSYNAPTIC MEMBRANE, (12) (GO) MONOVALENT INORGANIC CATION TRANSPORT, (13) (GO) BEHAVIOR, (14) (GO) MONOVALENT INORGANIC CATION TRANSMEMBRANE TRANSPORTER ACTIVITY, (15) (GO) PRESYNAPTIC MEMBRANE, (16) (GO) CATION TRANSMEMBRANE TRANSPORT, (17) (GO) POSTSYNAPTIC SPECIALIZATION MEMBRANE, (18) (GO) POSTSYNAPTIC DENSITY MEMBRANE, (19) (GO) TRANSMEMBRANE TRANSPORT, (20) (GO) INORGANIC ION TRANSMEMBRANE TRANSPORT, (21) (GO) ION TRANSMEMBRANE TRANSPORT, (22) (GO) GLUTAMATE RECEPTOR SIGNALING PATHWAY

	REPAIR, (52) (R) FCERI MEDIATED NF KB ACTIVATION, (53) (R) CYCLIN A CDK2 ASSOCIATED EVENTS AT S PHASE ENTRY, (54) (R) DOWNSTREAM SIGNALING EVENTS OF B CELL RECEPTOR BCR, (55) (R) PROCESSING OF CAPPED INTRON CONTAINING PRE MRNA, (56) (R) G1 S DNA DAMAGE CHECKPOINTS, (57) (R) CELL CYCLE, (58) (R) PCP CE PATHWAY, (59) (R) M PHASE, (60) (R) SIGNALING BY HEDGEHOG, (61) (R) UCH PROTEINASES, (62) (R) HIV LIFE CYCLE, (63) (R) MAPK6 MAPK4 SIGNALING, (64) (R) HEDGEHOG ON STATE, (65) (R) SIGNALING BY NOTCH4, (66) (R) CELL CYCLE MITOTIC, (67) (R) (GO)LGI TO ER RETROGRADE TRANSPORT, (68) (R) PTEN REGULATION, (69) (R) CELLULAR RESPONSES TO EXTERNAL STIMULI, (70) (R) MRNA SPLICING, (71) (R) GLOBAL GENOME NUCLEOTIDE EXCISION REPAIR GG NER, (72) (R) MITOTIC G1 PHASE AND G1 S TRANSITION, (73) (R) DEGRADATION OF BETA CATENIN BY THE DESTRUCTION COMPLEX, (74) (R) UB SPECIFIC PROCESSING PROTEASES, (75) (R) FC EPSILON RECEPTOR FCERI SIGNALING, (76) (R) INFECTIOUS DISEASE, (77) (R) MHC CLASS II ANTIGEN PRESENTATION, (78) (R) NERVOUS SYSTEM DEVELOPMENT, (79) (K) RNA DEGRADATION, (80) (K) UBIQUITIN MEDIATED PROTEOLYSIS, (81) (R) DEUBIQUITINATION, (82) (R) INTRA (GO)LGI AND RETROGRADE (GO)LGI TO ER TRAFFIC, (83) (R) CILIUM ASSEMBLY, (84) (R) ANTIGEN PROCESSING CROSS PRESENTATION, (85) (R) VIRAL MESSENGER RNA SYNTHESIS, (86) (GO) ANAPHASE PROMOTING COMPLEX DEPENDENT CATABOLIC PROCESS, (87) (GO) CATALYTIC ACTIVITY ACTING ON RNA, (88) (GO) CATALYTIC COMPLEX, (89) (GO) CELLULAR MACROMOLECULE CATABOLIC PROCESS, (90) (GO) CELLULAR MACROMOLECULE LOCALIZATION, (91) (GO) CELLULAR PROTEIN CATABOLIC PROCESS, (92) (GO) CELLULAR PROTEIN CONTAINING COMPLEX ASSEMBLY, (93) (GO) ENDOPEPTIDASE COMPLEX, (94) (GO) ENVELOPE, (95) (GO) (GO)LGI VESICLE TRANSPORT, (96) (GO) HYDROLASE ACTIVITY ACTING ON ACID ANHYDRIDES, (97) (GO) INTRACELLULAR PROTEIN TRANSPORT, (98) (GO) INTRACELLULAR TRANSPORT, (99) (GO) MACROAUTOPHAGY, (100) (GO) MODIFICATION DEPENDENT MACROMOLECULE CATABOLIC PROCESS, (101) (GO) PEPTIDASE COMPLEX, (102) (GO) PROTEASOMAL PROTEIN CATABOLIC PROCESS, (103) (GO) PROTEIN CONTAINING COMPLEX ASSEMBLY, (104) (GO) PROTEIN MODIFICATION BY SMALL PROTEIN CONJUGATION, (105) (GO) PROTEIN MODIFICATION BY SMALL PROTEIN CONJUGATION OR REMOVAL, (106) (GO) RIBONUCLEOPROTEIN COMPLEX, (107) (GO) RNA BINDING, (108) (GO) RNA PROCESSING, (109) (GO) SCF DEPENDENT PROTEASOMAL UBIQUITIN DEPENDENT PROTEIN CATABOLIC PROCESS, (110) (GO) REGULATION OF CELLULAR AMINO ACID METABOLIC PROCESS, (111) (GO) MEMBRANE ORGANIZATION, (112) (GO) RIBONUCLEOTIDE BINDING, (113) (GO) REGULATION OF CELL CYCLE G2 M PHASE TRANSITION, (114) (GO) CELL CYCLE G2 M PHASE TRANSITION, (115) (GO) PROTEIN POLYUBIQUITINATION, (116) (GO) PROTEIN CATABOLIC PROCESS, (117) (GO) NUCLEOLUS, (118) (GO) NEGATIVE REGULATION OF CELL CYCLE G2 M PHASE TRANSITION, (119) (GO) PROTEIN LOCALIZATION TO ORGANELLE, (120) (GO) PROTEIN MODIFICATION BY SMALL PROTEIN REMOVAL, (121) (GO) POSTTRANSCRIPTIONAL	

	REGULATION OF GENE EXPRESSION, (122) (GO) PROTEIN CONTAINING COMPLEX DISASSEMBLY, (123) (GO) TRANSFERASE COMPLEX, (124) (GO) MRNA METABOLIC PROCESS, (125) (GO) ORGANELLE LOCALIZATION, (126) (GO) REGULATION OF TRANSCRIPTION FROM RNA POLYMERASE II PROMOTER IN RESPONSE TO HYPOXIA, (127) (GO) MICROTUBULE CYTOSKELETON, (128) (GO) VESICLE ORGANIZATION, (129) (GO) ADENYL NUCLEOTIDE BINDING, (130) (GO) ANTIGEN PROCESSING AND PRESENTATION OF PEPTIDE ANTIGEN, (131) (GO) MACROMOLECULE CATABOLIC PROCESS, (132) (GO) MICROTUBULE BASED PROCESS, (133) (GO) PROCESS UTILIZING AUTOPHAGIC MECHANISM, (134) (GO) (GO)LGI MEMBRANE, (135) (GO) PROTEASOME ACCESSORY COMPLEX, (136) (GO) VACUOLAR TRANSPORT, (137) (GO) RNA SPLICING VIA TRANSESTERIFICATION REACTIONS, (138) (GO) REGULATION OF MRNA CATABOLIC PROCESS, (139) (GO) ANTIGEN PROCESSING AND PRESENTATION OF EXOGENOUS PEPTIDE ANTIGEN VIA MHC CLASS I, (140) (GO) ENDOSOMAL TRANSPORT, (141) (GO) PROTEIN CONTAINING COMPLEX LOCALIZATION, (142) (GO) RNA CATABOLIC PROCESS, (143) (GO) CELLULAR COMPONENT DISASSEMBLY, (144) (GO) FC EPSILON RECEPTOR SIGNALING PATHWAY, (145) (GO) REGULATION OF MACROAUTOPHAGY, (146) (GO) ORGANELLE SUBCOMPARTMENT, (147) (GO) NUCLEAR ENVELOPE, (148) (GO) DRUG BINDING, (149) (GO) REGULATION OF CATABOLIC PROCESS, (150) (GO) ATPASE ACTIVITY, (151) (GO) ESTABLISHMENT OF ORGANELLE LOCALIZATION, (152) (GO) UBIQUITIN LIKE PROTEIN LIGASE BINDING, (153) (GO) PROTEASOME REGULATORY PARTICLE BASE SUBCOMPLEX, (154) (GO) MRNA PROCESSING, (155) (GO) RNA LOCALIZATION, (156) (GO) INTERLEUKIN 1 MEDIATED SIGNALING PATHWAY, (157) (GO) REGULATION OF CELLULAR CATABOLIC PROCESS, (158) (GO) RNA SPLICING, (159) (GO) ANTIGEN PROCESSING AND PRESENTATION, (160) (GO) ORGANONITROGEN COMPOUND CATABOLIC PROCESS, (161) (GO) ENDOSOME ORGANIZATION	

Table S13. Jointly deregulated pathways in AD and PRCA.

	Up_PRCA	Down_PRCA
Up_AD		(1) (H)K EPITHELIAL MESENCHYMAL TRANSITION, (2) (H)K MYOGENESIS, (3) (H)K HYPOXIA, (4) NABA CORE MATRISOME, (5) NABA MATRISOME, (6) (R) EXTRACELLULAR MATRIX ORGANIZATION, (7) (GO) ANATOMICAL STRUCTURE FORMATION INVOLVED IN MORPHOGENESIS, (8) (GO) ANIMAL ORGAN MORPHOGENESIS, (9) (GO) BIOLOGICAL ADHESION, (10) (GO) BLOOD VESSEL MORPHOGENESIS, (11) (GO) BONE DEVELOPMENT, (12) (GO) CARDIOVASCULAR SYSTEM DEVELOPMENT, (13) (GO) CELL MOTILITY, (14) (GO) CELL SURFACE, (15) (GO) CIRCULATORY SYSTEM DEVELOPMENT, (16) (GO) COLLAGEN CONTAINING EXTRACELLULAR

		MATRIX, (17) (GO) CONNECTIVE TISSUE DEVELOPMENT, (18) (GO) EMBRYONIC MORPHOGENESIS, (19) (GO) EMBRYONIC ORGAN DEVELOPMENT, (20) (GO) EMBRYO DEVELOPMENT, (21) (GO) EPITHELIAL CELL DIFFERENTIATION, (22) (GO) EPITHELIUM DEVELOPMENT, (23) (GO) EXTRACELLULAR MATRIX, (24) (GO) EXTRACELLULAR MATRIX STRUCTURAL CONSTITUENT, (25) (GO) EXTRACELLULAR STRUCTURE ORGANIZATION, (26) (GO) INTRINSIC COMPONENT OF PLASMA MEMBRANE, (27) (GO) LOCOMOTION, (28) (GO) NEGATIVE REGULATION OF DEVELOPMENTAL PROCESS, (29) (GO) NEGATIVE REGULATION OF MULTICELLULAR ORGANISMAL PROCESS, (30) (GO) POSITIVE REGULATION OF DEVELOPMENTAL PROCESS, (31) (GO) REGULATION OF CELL ADHESION, (32) (GO) REGULATION OF CELL POPULATION PROLIFERATION, (33) (GO) REGULATION OF VASCULATURE DEVELOPMENT, (34) (GO) RESPONSE TO WOUNDING, (35) (GO) SENSORY ORGAN DEVELOPMENT, (36) (GO) SKELETAL SYSTEM DEVELOPMENT, (37) (GO) SKELETAL SYSTEM MORPHOGENESIS, (38) (GO) TUBE DEVELOPMENT, (39) (GO) TUBE MORPHOGENESIS, (40) (GO) UROGENITAL SYSTEM DEVELOPMENT, (41) (GO) WOUND HEALING, (42) (GO) POSITIVE REGULATION OF MULTICELLULAR ORGANISMAL PROCESS, (43) (GO) RENAL SYSTEM DEVELOPMENT, (44) (GO) CARTILAGE DEVELOPMENT, (45) (GO) SENSORY SYSTEM DEVELOPMENT, (46) (GO) MUSCLE STRUCTURE DEVELOPMENT, (47) (GO) POSITIVE REGULATION OF CELL ADHESION, (48) (GO) HEART DEVELOPMENT, (49) (GO) REGULATION OF CELL DIFFERENTIATION, (50) (GO) NEGATIVE REGULATION OF CELL DIFFERENTIATION, (51) (GO) DIGESTIVE SYSTEM DEVELOPMENT, (52) (GO) TISSUE MORPHOGENESIS, (53) (GO) REGULATION OF CELLULAR COMPONENT MOVEMENT, (54) (GO) POSITIVE REGULATION OF LOCOMOTION, (55) (GO) CELL SUBSTRATE ADHESION, (56) (GO) SIGNALING RECEPTOR BINDING, (57) (GO) HEART MORPHOGENESIS, (58) (GO) RESPONSE TO GROWTH FACTOR, (59) (GO) POSITIVE REGULATION OF CELL DIFFERENTIATION, (60) (GO) ENDOTHELIAL CELL APOPTOTIC PROCESS, (61) (GO) POSITIVE REGULATION OF PHOSPHORUS

		METABOLIC PROCESS, (62) (GO) POSITIVE REGULATION OF SIGNALING, (63) (GO) CHONDROCYTE DIFFERENTIATION, (64) (GO) MESENCHYME DEVELOPMENT, (65) (GO) MORPHOGENESIS OF AN EPITHELIUM, (66) (GO) BASEMENT MEMBRANE, (67) (GO) ANCHORING JUNCTION, (68) (GO) NEGATIVE REGULATION OF CELL POPULATION PROLIFERATION, (69) (GO) TAXIS, (70) (GO) REGULATION OF FAT CELL DIFFERENTIATION, (71) (GO) CARDIAC CHAMBER MORPHOGENESIS, (72) (GO) REGULATION OF CELLULAR RESPONSE TO GROWTH FACTOR STIMULUS, (73) (GO) REGULATION OF ANATOMICAL STRUCTURE MORPHOGENESIS, (74) (GO) MUSCLE SYSTEM PROCESS
Down_AD	(1) (H)K MYC TARGETS V1, (2) (H)K MTORC1 SIGNALING, (3) (H)K UNFOLDED PROTEIN RESPONSE, (4) (R) ASPARAGINE N LINKED GLYCOSYLATION, (5) (R) CELL CYCLE CHECKPOINTS, (6) (R) METABOLISM OF RNA, (7) (R) MITOCHONDRIAL TRANSLATION, (8) (R) MITOTIC METAPHASE AND ANAPHASE, (9) (R) TRANSLATION, (10) (R) G2 M CHECKPOINTS, (11) (R) CELL CYCLE, (12) (R) M PHASE, (13) (R) TRNA AMINOACYLATION, (14) (R) CELL CYCLE MITOTIC, (15) (K) AMINOACYL TRNA BIOSYNTHESIS, (16) (R) METABOLISM OF AMINO ACIDS AND DERIVATIVES, (17) (R) CYTOSOLIC TRNA AMINOACYLATION, (18) (R) TRNA PROCESSING, (19) (R) RRNA PROCESSING, (20) (R) RRNA MODIFICATION IN THE NUCLEUS AND CYTOSOL, (21) (GO) AMIDE BIOSYNTHETIC PROCESS, (22) (GO) CATALYTIC ACTIVITY ACTING ON RNA, (23) (GO) CELLULAR AMIDE METABOLIC PROCESS, (24) (GO) MITOCHONDRIAL GENE EXPRESSION, (25) (GO) MITOCHONDRIAL MATRIX, (26) (GO) MITOCHONDRIAL PROTEIN COMPLEX, (27) (GO) MITOCHONDRIAL TRANSLATION, (28) (GO) MITOCHONDRION, (29) (GO) NCRNA METABOLIC PROCESS, (30) (GO) NCRNA PROCESSING, (31) (GO) ORGANONITROGEN COMPOUND BIOSYNTHETIC PROCESS, (32) (GO) PEPTIDE BIOSYNTHETIC PROCESS, (33) (GO) PEPTIDE METABOLIC PROCESS, (34) (GO) RIBONUCLEOPROTEIN COMPLEX, (35) (GO) RIBONUCLEOPROTEIN COMPLEX BIOGENESIS, (36) (GO) RIBOSOME, (37) (GO) RIBOSOME BIOGENESIS, (38) (GO) RNA BINDING, (39) (GO) RNA PROCESSING, (40) (GO) TRNA METABOLIC PROCESS, (41) (GO) CELLULAR AMINO ACID METABOLIC PROCESS, (42) (GO) RIBOSOMAL SUBUNIT, (43) (GO) CATALYTIC ACTIVITY ACTING ON A TRNA, (44) (GO) NUCLEOLUS, (45) (GO) RRNA METABOLIC PROCESS, (46) (GO) STRUCTURAL CONSTITUENT OF RIBOSOME, (47) (GO) AMINO ACID ACTIVATION, (48) (GO) RIBONUCLEOPROTEIN COMPLEX SUBUNIT ORGANIZATION, (49) (GO) SMALL MOLECULE METABOLIC PROCESS, (50) (GO) LARGE RIBOSOMAL SUBUNIT, (51) (GO) ADENYL NUCLEOTIDE BINDING, (52) (GO) LIGASE ACTIVITY FORMING CARBON OXYGEN BONDS, (53) (GO) RIBONUCLEOPROTEIN COMPLEX BINDING, (54) (GO) DRUG BINDING, (55) (GO) ATPASE ACTIVITY, (56) (GO) CYTOPLASMIC TRANSLATION, (57) (GO) PRERIBOSOME	(1) (GO) SYNAPSE, (2) (GO) REGULATION OF NEURON PROJECTION DEVELOPMENT

Table S14. Jointly deregulated pathways in AD and SKCM.

	Up_SKCM	Down_SKCM
Up_AD	(1) (H)K INFLAMMATORY RESPONSE, (2) (H)K ALLOGRAFT REJECTION, (3) (R) IMMUNOREGULATORY INTERACTIONS BETWEEN A LYMPHOID AND A NON LYMPHOID CELL, (4) (GO) ADAPTIVE IMMUNE RESPONSE, (5) (GO) CELL ACTIVATION, (6) (GO) CYTOKINE MEDIATED SIGNALING PATHWAY, (7) (GO) CYTOKINE PRODUCTION, (8) (GO) DEFENSE RESPONSE, (9) (GO) POSITIVE REGULATION OF IMMUNE SYSTEM PROCESS, (10) (GO) REGULATION OF IMMUNE SYSTEM PROCESS, (11) (GO) RESPONSE TO BIOTIC STIMULUS, (12) (GO) DEFENSE RESPONSE TO OTHER ORGANISM, (13) (GO) REGULATION OF IMMUNE RESPONSE, (14) (GO) CELLULAR RESPONSE TO BIOTIC STIMULUS, (15) (GO) INNATE IMMUNE RESPONSE, (16) (GO) POSITIVE REGULATION OF IMMUNE RESPONSE, (17) (GO) IMMUNE EFFECTOR PROCESS, (18) (GO) ACTIVATION OF IMMUNE RESPONSE, (19) (GO) REGULATION OF DEFENSE RESPONSE	(1) (H)K MYOGENESIS, (2) NABA CORE MATRISOME, (3) NABA ECM GLYCOPROTEINS, (4) (R) KERATINIZATION, (5) (R) FORMATION OF THE CORNIFIED ENVELOPE, (6) (GO) ANIMAL ORGAN MORPHOGENESIS, (7) (GO) CIRCULATORY SYSTEM DEVELOPMENT, (8) (GO) E(PID)ERMIS DEVELOPMENT, (9) (GO) EPITHELIAL CELL DIFFERENTIATION, (10) (GO) EPITHELIUM DEVELOPMENT, (11) (GO) SKIN DEVELOPMENT, (12) (GO) UROGENITAL SYSTEM DEVELOPMENT, (13) (GO) E(PID)ERMAL CELL DIFFERENTIATION, (14) (GO) RENAL SYSTEM DEVELOPMENT, (15) (GO) MUSCLE STRUCTURE DEVELOPMENT, (16) (GO) HEART DEVELOPMENT, (17) (GO) GLAND DEVELOPMENT, (18) (GO) KIDNEY EPITHELIUM DEVELOPMENT, (19) (GO) KERATINOCYTE DIFFERENTIATION, (20) (GO) MUSCLE ORGAN DEVELOPMENT, (21) (GO) EPITHELIAL CELL PROLIFERATION, (22) (GO) REPRODUCTIVE SYSTEM DEVELOPMENT, (23) (GO) KERATINIZATION, (24) (GO) ANCHORING JUNCTION, (25) (GO) MOLTING CYCLE, (26) (GO) CORNIFICATION, (27) (GO) KERATIN FILAMENT, (28) (GO) MOLTING CYCLE PROCESS
Down_AD	(1) (H)K MTORC1 SIGNALING, (2) (H)K DNA REPAIR, (3) (H)K UNFOLDED PROTEIN RESPONSE, (4) (R) APC C CDH1 MEDIATED DEGRADATION OF CDC20 AND OTHER APC C CDH1 TARGETED PROTEINS IN LATE MITOSIS EARLY G1, (5) (R) ASSEMBLY OF THE PRE REPLICATIVE COMPLEX, (6) (R) CELL CYCLE CHECKPOINTS, (7) (R) DNA REPLICATION, (8) (R) HIV INFECTION, (9) (R) MITOTIC G2 G2 M PHASES, (10) (R) MITOTIC METAPHASE AND ANAPHASE, (11) (R) ORC1 REMOVAL FROM CHROMATIN, (12) (R) SEPARATION OF SISTER CHROMATIDS, (13) (R) SWITCHING OF ORIGINS TO A POST REPLICATIVE STATE, (14) (R) APC C MEDIATED DEGRADATION OF CELL CYCLE PROTEINS, (15) (R) DNA REPLICATION PRE INITIATION, (16) (R) S PHASE, (17) (R) G2 M CHECKPOINTS, (18) (R) CELL CYCLE, (19) (R) M PHASE, (20) (R) CELL CYCLE MITOTIC, (21) (R) MITOTIC G1 PHASE AND G1 S TRANSITION, (22) (R) ANTIGEN PROCESSING CROSS PRESENTATION, (23) (GO) ANAPHASE	(1) (H)K FATTY ACID METABOLISM, (2) (GO) RIBOSOMAL SUBUNIT, (3) (GO) PROTEIN TARGETING, (4) (GO) STRUCTURAL CONSTITUENT OF RIBOSOME, (5) (GO) OXIDATION REDUCTION PROCESS, (6) (GO) PROTEIN LOCALIZATION TO MEMBRANE, (7) (GO) OXIDOREDUCTASE ACTIVITY, (8) (GO) ESTABLISHMENT OF PROTEIN LOCALIZATION TO MEMBRANE

	PROMOTING COMPLEX DEPENDENT CATABOLIC PROCESS, (24) (GO) CATALYTIC ACTIVITY ACTING ON RNA, (25) (GO) HYDROLASE ACTIVITY ACTING ON ACID ANHYDRIDES, (26) (GO) REGULATION OF CELL CYCLE G2 M PHASE TRANSITION, (27) (GO) CELL CYCLE G2 M PHASE TRANSITION, (28) (GO) ORGANELLE LOCALIZATION, (29) (GO) MICROTUBULE CYTOSKELETON, (30) (GO) ANTIGEN PROCESSING AND PRESENTATION OF PEPTIDE ANTIGEN, (31) (GO) ATPASE ACTIVITY, (32) (GO) ESTABLISHMENT OF ORGANELLE LOCALIZATION, (33) (GO) ANTIGEN PROCESSING AND PRESENTATION	

Table S15. Jointly deregulated pathways in PD and BRNCA.

	Up_BRNCA	Down_BRNCA
Up_PD	(1) (H)K INFLAMMATORY RESPONSE, (2) (H)K IL6 JAK STAT3 SIGNALING, (3) (H)K TNFA SIGNALING VIA NFKB, (4) (H)K IL2 STAT5 SIGNALING, (5) (H)K INTERFERON GAMMA RESPONSE, (6) (H)K P53 PATHWAY, (7) (H)K INTERFERON ALPHA RESPONSE, (8) (K) CYTOKINE CYTOKINE RECEPTOR INTERACTION, (9) (R) EXTRACELLULAR MATRIX ORGANIZATION, (10) (R) COLLAGEN FORMATION, (11) (K) PATHWAYS IN CANCER, (12) (R) INTEGRIN CELL SURFACE INTERACTIONS, (13) (R) COLLAGEN BIOSYNTHESIS AND MODIFYING ENZYMES, (14) (BC) NFKB PATHWAY, (15) (PID) E2F PATHWAY, (16) (R) RNA POLYMERASE II TRANSCRIPTION, (17) (R) RESPONSE OF EIF2AK4 GCN2 TO AMINO ACID DEFICIENCY, (18) (R) EUKARYOTIC TRANSLATION ELONGATION, (19) (GO) ADAPTIVE IMMUNE RESPONSE, (20) (GO) ANATOMICAL STRUCTURE FORMATION INVOLVED IN MORPHOGENESIS, (21) (GO) BIOLOGICAL ADHESION, (22) (GO) BLOOD VESSEL MORPHOGENESIS, (23) (GO) CARDIOVASCULAR SYSTEM DEVELOPMENT, (24) (GO) CELL ACTIVATION, (25) (GO) CHROMATIN, (26) (GO) CHROMOSOME, (27) (GO) CIRCULATORY SYSTEM DEVELOPMENT, (28) (GO) CIS REGULATORY REGION BINDING, (29) (GO) COLLAGEN CONTAINING EXTRACELLULAR MATRIX, (30) (GO) CYTOKINE MEDIATED SIGNALING PATHWAY, (31) (GO) CYTOKINE PRODUCTION, (32) (GO) DEFENSE RESPONSE, (33) (GO) DOUBLE STRANDED DNA BINDING, (34) (GO)	

	EMBRYO DEVELOPMENT, (35) (GO) EXTRACELLULAR STRUCTURE ORGANIZATION, (36) (GO) IMMUNE SYSTEM DEVELOPMENT, (37) (GO) INFLAMMATORY RESPONSE, (38) (GO) LEUKOCYTE CELL CELL ADHESION, (39) (GO) LEUKOCYTE PROLIFERATION, (40) (GO) LYMPHOCYTE ACTIVATION, (41) (GO) NEGATIVE REGULATION OF BIOSYNTHETIC PROCESS, (42) (GO) NEGATIVE REGULATION OF IMMUNE SYSTEM PROCESS, (43) (GO) NEGATIVE REGULATION OF NUCLEOBASE CONTAINING COMPOUND METABOLIC PROCESS, (44) (GO) NEGATIVE REGULATION OF RNA BIOSYNTHETIC PROCESS, (45) (GO) NEGATIVE REGULATION OF TRANSCRIPTION BY RNA POLYMERASE II, (46) (GO) NUCLEAR CHROMOSOME, (47) (GO) POSITIVE REGULATION OF CELLULAR BIOSYNTHETIC PROCESS, (48) (GO) POSITIVE REGULATION OF IMMUNE SYSTEM PROCESS, (49) (GO) POSITIVE REGULATION OF NUCLEOBASE CONTAINING COMPOUND METABOLIC PROCESS, (50) (GO) POSITIVE REGULATION OF RNA METABOLIC PROCESS, (51) (GO) POSITIVE REGULATION OF TRANSCRIPTION BY RNA POLYMERASE II, (52) (GO) POSITIVE REGULATION OF VASCULATURE DEVELOPMENT, (53) (GO) REGULATION OF CELL ACTIVATION, (54) (GO) REGULATION OF CELL ADHESION, (55) (GO) REGULATION OF CELL POPULATION PROLIFERATION, (56) (GO) REGULATION OF IMMUNE SYSTEM PROCESS, (57) (GO) REGULATION OF LEUKOCYTE PROLIFERATION, (58) (GO) REGULATION OF LYMPHOCYTE ACTIVATION, (59) (GO) REGULATION OF VASCULATURE DEVELOPMENT, (60) (GO) RESPONSE TO BACTERIUM, (61) (GO) RESPONSE TO BIOTIC STIMULUS, (62) (GO) RESPONSE TO MOLECULE OF BACTERIAL ORIGIN, (63) (GO) SEQUENCE SPECIFIC DNA BINDING, (64) (GO) SEQUENCE SPECIFIC DOUBLE STRANDED DNA BINDING, (65) (GO) SKELETAL SYSTEM DEVELOPMENT, (66) (GO) TRANSCRIPTION REGULATOR ACTIVITY, (67) (GO) TUBE DEVELOPMENT, (68) (GO) TUBE MORPHOGENESIS, (69) (GO) T CELL ACTIVATION, (70) (GO) T CELL DIFFERENTIATION, (71) (GO) POSITIVE REGULATION OF CELL ACTIVATION, (72) (GO) RESPONSE TO CYTOKINE, (73) (GO) REGULATION OF T CELL ACTIVATION, (74) (GO) T CELL PROLIFERATION, (75) (GO) EMBRYO DEVELOPMENT ENDING IN BIRTH OR	

	EGG HATCHING, (76) (GO) DEFENSE RESPONSE TO OTHER ORGANISM, (77) (GO) REGULATION OF IMMUNE RESPONSE, (78) (GO) REGULATION OF IMMUNE EFFECTOR PROCESS, (79) (GO) TUMOR NECROSIS FACTOR SUPERFAMILY CYTOKINE PRODUCTION, (80) (GO) CELLULAR RESPONSE TO BIOTIC STIMULUS, (81) (GO) ADAPTIVE IMMUNE RESPONSE BASED ON SOMATIC RECOMBINATION OF IMMUNE RECEPTORS BUILT FROM IMMUNOGLOBULIN SUPERFAMILY DOMAINS, (82) (GO) INTERLEUKIN 6 PRODUCTION, (83) (GO) GROWTH FACTOR BINDING, (84) (GO) POSITIVE REGULATION OF LOCOMOTION, (85) (GO) TRANSCRIPTION FACTOR COMPLEX, (86) (GO) PATTERN RECOGNITION RECEPTOR SIGNALING PATHWAY, (87) (GO) INNATE IMMUNE RESPONSE, (88) (GO) POSITIVE REGULATION OF IMMUNE RESPONSE, (89) (GO) COLLAGEN METABOLIC PROCESS, (90) (GO) POSITIVE REGULATION OF CYTOKINE PRODUCTION, (91) (GO) IMMUNE EFFECTOR PROCESS, (92) (GO) TOLL LIKE RECEPTOR SIGNALING PATHWAY, (93) (GO) REGULATION OF RESPONSE TO EXTERNAL STIMULUS, (94) (GO) I KAPPAB KINASE NF KAPPAB SIGNALING, (95) (GO) POSITIVE REGULATION OF TUMOR NECROSIS FACTOR SUPERFAMILY CYTOKINE PRODUCTION, (96) (GO) CELLULAR RESPONSE TO VASCULAR ENDOTHELIAL GROWTH FACTOR STIMULUS, (97) (GO) CHROMATIN BINDING, (98) (GO) REGULATION OF ADAPTIVE IMMUNE RESPONSE, (99) (GO) ACTIVATION OF IMMUNE RESPONSE, (100) (GO) REGULATION OF DEFENSE RESPONSE, (101) (GO) REGULATION OF RESPONSE TO CYTOKINE STIMULUS, (102) (GO) CELL SUBSTRATE JUNCTION, (103) (GO) POSITIVE REGULATION OF I KAPPAB KINASE NF KAPPAB SIGNALING, (104) (GO) MYELOID LEUKOCYTE ACTIVATION, (105) (GO) CELL ACTIVATION INVOLVED IN IMMUNE RESPONSE, (106) (GO) LEUKOCYTE MEDIATED IMMUNITY, (107) (GO) NEGATIVE REGULATION OF CELL CYCLE G1 S PHASE TRANSITION, (108) (GO) NEGATIVE REGULATION OF MULTI ORGANISM PROCESS, (109) (GO) REGULATION OF RESPONSE TO STRESS, (110) (GO) NUCLEAR SPECK, (111) (GO) CHROMOSOME ORGANIZATION, (112) (GO) INTERSPECIES INTERACTION BETWEEN ORGANISMS, (113) (GO)	

	NUCLEAR BODY, (114) (GO) CHROMATIN ORGANIZATION, (115) (GO) REGULATION OF GENE EXPRESSION EPIGENETIC	
Down_PD	(1) (H)K MTORC1 SIGNALING, (2) (R) APC C CDH1 MEDIATED DEGRADATION OF CDC20 AND OTHER APC C CDH1 TARGETED PROTEINS IN LATE MITOSIS EARLY G1, (3) (R) ASSEMBLY OF THE PRE REPLICATIVE COMPLEX, (4) (R) AUF1 HNRNP D0 BINDS AND DESTABILIZES MRNA, (5) (R) CELL CYCLE CHECKPOINTS, (6) (R) DNA REPLICATION, (7) (R) HIV INFECTION, (8) (R) MITOTIC METAPHASE AND ANAPHASE, (9) (R) ORC1 REMOVAL FROM CHROMATIN, (10) (R) SEPARATION OF SISTER CHROMATIDS, (11) (R) SWITCHING OF ORIGINS TO A POST REPLICATIVE STATE, (12) (R) APC C MEDIATED DEGRADATION OF CELL CYCLE PROTEINS, (13) (R) DNA REPLICATION PRE INITIATION, (14) (R) STABILIZATION OF P53, (15) (R) REGULATION OF RUNX3 EXPRESSION AND ACTIVITY, (16) (R) G2 M CHECKPOINTS, (17) (R) CYCLIN A CDK2 ASSOCIATED EVENTS AT S PHASE ENTRY, (18) (R) G1 S DNA DAMAGE CHECKPOINTS, (19) (R) M PHASE, (20) (R) SIGNALING BY NOTCH4, (21) (GO) ANAPHASE PROMOTING COMPLEX DEPENDENT CATABOLIC PROCESS, (22) (GO) MITOCHONDRIAL GENE EXPRESSION, (23) (GO) MITOCHONDRIAL MATRIX, (24) (GO) CARBOHYDRATE DERIVATIVE METABOLIC PROCESS	(1) (R) MEMBRANE TRAFFICKING, (2) (R) NEURONAL SYSTEM, (3) (R) TRANSMISSION ACROSS CHEMICAL SYNAPSES, (4) (R) VESICLE MEDIATED TRANSPORT, (5) (K) CARDIAC MUSCLE CONTRACTION, (6) (R) NEUROTRANSMITTER RECEPTORS AND POSTSYNAPTIC SIGNAL TRANSMISSION, (7) (R) ACTIVATION OF NMDA RECEPTORS AND POSTSYNAPTIC EVENTS, (8) (R) PROTEIN PROTEIN INTERACTIONS AT SYNAPSES, (9) (R) L1CAM INTERACTIONS, (10) (R) NEUROTRANSMITTER RELEASE CYCLE, (11) (R) TRAFFICKING OF AMPA RECEPTORS, (12) (R) TRANSPORT OF SMALL MOLECULES, (13) (R) PHASE 0 RA(PID) DEPOLARISATION, (14) (GO) AXON, (15) (GO) EXOCYTIC VESICLE, (16) (GO) GLUTAMATERGIC SYNAPSE, (17) (GO) MEMBRANE PROTEIN COMPLEX, (18) (GO) NEURON PROJECTION, (19) (GO) NEURON TO NEURON SYNAPSE, (20) (GO) POSTSYNAPSE, (21) (GO) PRESYNAPSE, (22) (GO) SOMATODENDRITIC COMPARTMENT, (23) (GO) SYNAPSE, (24) (GO) SYNAPTIC MEMBRANE, (25) (GO) SYNAPTIC SIGNALING, (26) (GO) TRANSPORT VESICLE, (27) (GO) VESICLE MEDIATED TRANSPORT IN SYNAPSE, (28) (GO) TRANSPORT VESICLE MEMBRANE, (29) (GO) DENDRITIC TREE, (30) (GO) SYNAPTIC VESICLE MEMBRANE, (31) (GO) MEMBRANE ORGANIZATION, (32) (GO) REGULATION OF TRANS SYNAPTIC SIGNALING, (33) (GO) REGULATION OF SYNAPTIC PLASTICITY, (34) (GO) CELL BODY, (35) (GO) REGULATION OF SYNAPTIC VESICLE CYCLE, (36) (GO) POSTSYNAPTIC MEMBRANE, (37) (GO) MONOVALENT INORGANIC CATION TRANSPORT, (38) (GO) SYNAPTIC VESICLE EXOCYTOSIS, (39) (GO) BEHAVIOR, (40) (GO) MONOVALENT INORGANIC CATION TRANSMEMBRANE TRANSPORTER ACTIVITY, (41) (GO) DISTAL AXON, (42) (GO) NEUROTRANSMITTER SECRETION, (43) (GO) PRESYNAPTIC MEMBRANE, (44) (GO) VESICLE LOCALIZATION, (45) (GO) CATION TRANSMEMBRANE TRANSPORT, (46) (GO) GABA ERGIC SYNAPSE, (47) (GO) SYNAPTIC VESICLE RECYCLING, (48) (GO) AXON CYTOPLASM, (49) (GO) POSTSYNAPTIC SPECIALIZATION MEMBRANE, (50) (GO)

		POSTSYNAPTIC DENSITY MEMBRANE, (51) (GO) NEUROTRANSMITTER TRANSPORT, (52) (GO) MICROTUBULE BASED TRANSPORT, (53) (GO) REGULATION OF NEURONAL SYNAPTIC PLASTICITY, (54) (GO) PRESYNAPTIC ACTIVE ZONE, (55) (GO) REGULATION OF SYNAPTIC VESICLE EXOCYTOSIS, (56) (GO) TRANSMEMBRANE TRANSPORT, (57) (GO) INORGANIC ION TRANSMEMBRANE TRANSPORT, (58) (GO) CYTOSKELETON DEPENDENT INTRACELLULAR TRANSPORT, (59) (GO) SITE OF POLARIZED GROWTH, (60) (GO) SYNAPSE ORGANIZATION, (61) (GO) ION TRANSMEMBRANE TRANSPORT, (62) (GO) SYNAPTIC VESICLE ENDOCYTOSIS, (63) (GO) SCHAFFER COLLATERAL CA1 SYNAPSE, (64) (GO) REGULATION OF NEUROTRANSMITTER TRANSPORT, (65) (GO) TUBULIN BINDING, (66) (GO) REGULATION OF NEUROTRANSMITTER SECRETION, (67) (GO) REGULATION OF POSTSYNAPTIC MEMBRANE POTENTIAL, (68) (GO) REGULATION OF SYNAPSE STRUCTURE OR ACTIVITY, (69) (GO) INTRINSIC COMPONENT OF SYNAPTIC MEMBRANE, (70) (GO) SYNAPTIC TRANSMISSION GLUTAMATERGIC, (71) (GO) EXCITATORY SYNAPSE, (72) (GO) INTRINSIC COMPONENT OF POSTSYNAPTIC SPECIALIZATION MEMBRANE, (73) (GO) ACIDIC AMINO ACID TRANSPORT, (74) (GO) CATION CHANNEL COMPLEX, (75) (GO) CATION TRANSMEMBRANE TRANSPORTER ACTIVITY, (76) (GO) REGULATION OF MEMBRANE POTENTIAL, (77) (GO) CYTOPLASMIC REGION, (78) (GO) CLATHRIN BINDING, (79) (GO) ESTABLISHMENT OF LOCALIZATION IN CELL, (80) (GO) INTRINSIC COMPONENT OF POSTSYNAPTIC MEMBRANE, (81) (GO) REGULATION OF NEUROTRANSMITTER LEVELS, (82) (GO) NEURON DEVELOPMENT, (83) (GO) REGULATION OF EXOCYTOSIS, (84) (GO) ION TRANSMEMBRANE TRANSPORTER ACTIVITY, (85) (GO) SIGNAL RELEASE, (86) (GO) CELL PART MORPHOGENESIS, (87) (GO) NEURON PROJECTION TERMINUS, (88) (GO) CATION TRANSPORT, (89) (GO) CELL MORPHOGENESIS INVOLVED IN NEURON DIFFERENTIATION, (90) (GO) TRANSPORTER COMPLEX, (91) (GO) AMINE TRANSPORT, (92) (GO) SYNAPSE ASSEMBLY, (93) (GO) TRANSPORTER ACTIVITY, (94) (GO) ION TRANSPORT, (95) (GO) CATION CHANNEL ACTIVITY, (96) (GO) REGULATION OF SYNAPSE ASSEMBLY, (97) (GO) CELL CELL

		SIGNALING, (98) (GO) DOPAMINE TRANSPORT, (99) (GO) CATECHOLAMINE SECRETION, (100) (GO) NEUROTRANSMITTER RECEPTOR ACTIVITY

Table S16. Jointly deregulated pathways in PD and KDNCA.

	Up_KDNCA	Down_KDNCA
Up_PD	(1) (H)K INFLAMMATORY RESPONSE, (2) (H)K IL6 JAK STAT3 SIGNALING, (3) (H)K TNFA SIGNALING VIA NFKB, (4) (H)K IL2 STAT5 SIGNALING, (5) (H)K INTERFERON GAMMA RESPONSE, (6) (H)K P53 PATHWAY, (7) (H)K INTERFERON ALPHA RESPONSE, (8) (K) CYTOKINE CYTOKINE RECEPTOR INTERACTION, (9) (PID) E2F PATHWAY, (10) (R) RNA POLYMERASE II TRANSCRIPTION, (11) (GO) ADAPTIVE IMMUNE RESPONSE, (12) (GO) BIOLOGICAL ADHESION, (13) (GO) BLOOD VESSEL MORPHOGENESIS, (14) (GO) CELL ACTIVATION, (15) (GO) CELL CELL ADHESION, (16) (GO) CELL MOTILITY, (17) (GO) CHROMOSOME, (18) (GO) CYTOKINE MEDIATED SIGNALING PATHWAY, (19) (GO) CYTOKINE PRODUCTION, (20) (GO) DEFENSE RESPONSE, (21) (GO) IMMUNE SYSTEM DEVELOPMENT, (22) (GO) INFLAMMATORY RESPONSE, (23) (GO) LEUKOCYTE CELL CELL ADHESION, (24) (GO) LEUKOCYTE DIFFERENTIATION, (25) (GO) LEUKOCYTE PROLIFERATION, (26) (GO) LYMPHOCYTE ACTIVATION, (27) (GO) LYMPHOCYTE DIFFERENTIATION, (28) (GO) NEGATIVE REGULATION OF BIOSYNTHETIC PROCESS, (29) (GO) NEGATIVE REGULATION OF IMMUNE SYSTEM PROCESS, (30) (GO) NEGATIVE REGULATION OF NUCLEOBASE CONTAINING COMPOUND METABOLIC PROCESS, (31) (GO) POSITIVE REGULATION OF IMMUNE SYSTEM PROCESS, (32) (GO) REGULATION OF CELL ACTIVATION, (33) (GO) REGULATION OF CELL ADHESION, (34) (GO) REGULATION OF IMMUNE SYSTEM PROCESS, (35) (GO) REGULATION OF LEUKOCYTE PROLIFERATION, (36) (GO) REGULATION OF LYMPHOCYTE ACTIVATION, (37) (GO) RESPONSE TO BACTERIUM, (38) (GO) RESPONSE TO BIOTIC STIMULUS, (39) (GO) RESPONSE TO MOLECULE OF BACTERIAL ORIGIN, (40) (GO) T CELL ACTIVATION, (41) (GO) REGULATION OF LEUKOCYTE DIFFERENTIATION, (42) (GO) NEGATIVE REGULATION OF CYTOKINE	

	PRODUCTION, (43) (GO) T CELL DIFFERENTIATION, (44) (GO) POSITIVE REGULATION OF CELL ACTIVATION, (45) (GO) RESPONSE TO CYTOKINE, (46) (GO) REGULATION OF T CELL ACTIVATION, (47) (GO) T CELL PROLIFERATION, (48) (GO) DEFENSE RESPONSE TO OTHER ORGANISM, (49) (GO) REGULATION OF IMMUNE RESPONSE, (50) (GO) REGULATION OF IMMUNE EFFECTOR PROCESS, (51) (GO) B CELL ACTIVATION, (52) (GO) POSITIVE REGULATION OF INTRACELLULAR SIGNAL TRANSDUCTION, (53) (GO) TUMOR NECROSIS FACTOR SUPERFAMILY CYTOKINE PRODUCTION, (54) (GO) NEGATIVE REGULATION OF CELL ACTIVATION, (55) (GO) CELLULAR RESPONSE TO BIOTIC STIMULUS, (56) (GO) MYELOID CELL DIFFERENTIATION, (57) (GO) ADAPTIVE IMMUNE RESPONSE BASED ON SOMATIC RECOMBINATION OF IMMUNE RECEPTORS BUILT FROM IMMUNOGLOBULIN SUPERFAMILY DOMAINS, (58) (GO) NEGATIVE REGULATION OF LYMPHOCYTE ACTIVATION, (59) (GO) INTERLEUKIN 6 PRODUCTION, (60) (GO) POSITIVE REGULATION OF LOCOMOTION, (61) (GO) PATTERN RECOGNITION RECEPTOR SIGNALING PATHWAY, (62) (GO) INNATE IMMUNE RESPONSE, (63) (GO) POSITIVE REGULATION OF INTERLEUKIN 6 PRODUCTION, (64) (GO) POSITIVE REGULATION OF IMMUNE RESPONSE, (65) (GO) POSITIVE REGULATION OF CYTOKINE PRODUCTION, (66) (GO) IMMUNE EFFECTOR PROCESS, (67) (GO) TOLL LIKE RECEPTOR SIGNALING PATHWAY, (68) (GO) REGULATION OF LEUKOCYTE MEDIATED IMMUNITY, (69) (GO) REGULATION OF RESPONSE TO EXTERNAL STIMULUS, (70) (GO) POSITIVE REGULATION OF LEUKOCYTE DIFFERENTIATION, (71) (GO) LYMPHOCYTE ACTIVATION INVOLVED IN IMMUNE RESPONSE, (72) (GO) I KAPPAB KINASE NF KAPPAB SIGNALING, (73) (GO) PHA(GO)CYTOSIS, (74) (GO) POSITIVE REGULATION OF TUMOR NECROSIS FACTOR SUPERFAMILY CYTOKINE PRODUCTION, (75) (GO) REGULATION OF ADAPTIVE IMMUNE RESPONSE, (76) (GO) ACTIVATION OF IMMUNE RESPONSE, (77) (GO) REGULATION OF DEFENSE RESPONSE, (78) (GO) REGULATION OF LYMPHOCYTE MEDIATED IMMUNITY, (79) (GO) LEUKOCYTE APOPTOTIC PROCESS, (80) (GO) REGULATION OF RESPONSE TO	

	CYTOKINE STIMULUS, (81) (GO) CELL SUBSTRATE JUNCTION, (82) (GO) POSITIVE REGULATION OF I KAPPAB KINASE NF KAPPAB SIGNALING, (83) (GO) MYELOID LEUKOCYTE ACTIVATION, (84) (GO) CELL ACTIVATION INVOLVED IN IMMUNE RESPONSE, (85) (GO) LEUKOCYTE MEDIATED IMMUNITY, (86) (GO) REGULATION OF INTRACELLULAR SIGNAL TRANSDUCTION, (87) (GO) NEGATIVE REGULATION OF MULTI ORGANISM PROCESS, (88) (GO) REGULATION OF RESPONSE TO STRESS, (89) (GO) CHROMOSOME ORGANIZATION, (90) (GO) INTERSPECIES INTERACTION BETWEEN ORGANISMS, (91) (GO) NUCLEAR BODY, (92) (GO) REGULATION OF GENE EXPRESSION EPIGENETIC	
Down_PD	(1) (H)K MTORC1 SIGNALING, (2) (R) CELL CYCLE CHECKPOINTS, (3) (R) DNA REPLICATION, (4) (R) HIV INFECTION, (5) (R) HOST INTERACTIONS OF HIV FACTORS, (6) (R) MITOTIC G2 G2 M PHASES, (7) (R) MITOTIC METAPHASE AND ANAPHASE, (8) (R) ORC1 REMOVAL FROM CHROMATIN, (9) (R) SEPARATION OF SISTER CHROMATIDS, (10) (R) SWITCHING OF ORIGINS TO A POST REPLICATIVE STATE, (11) (R) DNA REPLICATION PRE INITIATION, (12) (R) G2 M CHECKPOINTS, (13) (R) CYCLIN A CDK2 ASSOCIATED EVENTS AT S PHASE ENTRY, (14) (R) DOWNSTREAM SIGNALING EVENTS OF B CELL RECEPTOR BCR, (15) (R) M PHASE, (16) (R) SIGNALING BY NOTCH4, (17) (R) TNFR2 NON CANONICAL NF KB PATHWAY, (18) (GO) CELL CYCLE G2 M PHASE TRANSITION, (19) (GO) ORGANELLE LOCALIZATION, (20) (GO) ESTABLISHMENT OF ORGANELLE LOCALIZATION	(1) (H)K OXIDATIVE PHOSPHORYLATION, (2) (H)K FATTY ACID METABOLISM, (3) (K) OXIDATIVE PHOSPHORYLATION, (4) (K) PARKINSONS DISEASE, (5) (R) RESPIRATORY ELECTRON TRANSPORT, (6) (R) RESPIRATORY ELECTRON TRANSPORT ATP SYNTHESIS BY CHEMIOSMOTIC COUPLING AND HEAT PRODUCTION BY UNCOUPLING PROTEINS, (7) (R) THE CITRIC ACID TCA CYCLE AND RESPIRATORY ELECTRON TRANSPORT, (8) (R) NEURONAL SYSTEM, (9) (R) TRANSMISSION ACROSS CHEMICAL SYNAPSES, (10) (R) PYRUVATE METABOLISM AND CITRIC ACID TCA CYCLE, (11) (K) CITRATE CYCLE TCA CYCLE, (12) (R) TRANSPORT OF SMALL MOLECULES, (13) (GO) AEROBIC RESPIRATION, (14) (GO) ATP SYNTHESIS COUPLED ELECTRON TRANSPORT, (15) (GO) CELLULAR RESPIRATION, (16) (GO) ELECTRON TRANSPORT CHAIN, (17) (GO) GENERATION OF PRECURSOR METABOLITES AND ENERGY, (18) (GO) INNER MITOCHONDRIAL MEMBRANE PROTEIN COMPLEX, (19) (GO) MITOCHONDRIAL ELECTRON TRANSPORT NADH TO UBIQUINONE, (20) (GO) MITOCHONDRIAL ENVELOPE, (21) (GO) MITOCHONDRIAL MATRIX, (22) (GO) MITOCHONDRIAL PROTEIN COMPLEX, (23) (GO) MITOCHONDRIAL RESPIRATORY CHAIN COMPLEX ASSEMBLY, (24) (GO) MITOCHONDRION, (25) (GO) NADH DEHYDROGENASE COMPLEX, (26) (GO) ORGANELLE INNER MEMBRANE, (27) (GO) OXIDATIVE PHOSPHORYLATION, (28) (GO) PROTON TRANSMEMBRANE TRANSPORT, (29) (GO) PROTON TRANSMEMBRANE

		TRANSPORTER ACTIVITY, (30) (GO) PROTON TRANSPORTING TWO SECTOR ATPASE COMPLEX, (31) (GO) RESPIRASOME, (32) (GO) RESPIRATORY CHAIN COMPLEX, (33) (GO) RESPIRATORY ELECTRON TRANSPORT CHAIN, (34) (GO) CELLULAR AMINO ACID METABOLIC PROCESS, (35) (GO) OXIDOREDUCTASE ACTIVITY ACTING ON NAD P H QUINONE OR SIMILAR COMPOUND AS ACCEPTOR, (36) (GO) TRICARBOXYLIC ACID CYCLE, (37) (GO) ORGANOPHOSPHATE METABOLIC PROCESS, (38) (GO) POSTSYNAPTIC MEMBRANE, (39) (GO) PURINE CONTAINING COMPOUND METABOLIC PROCESS, (40) (GO) NUCLEOBASE CONTAINING SMALL MOLECULE METABOLIC PROCESS, (41) (GO) OXIDATION REDUCTION PROCESS, (42) (GO) CRISTAE FORMATION, (43) (GO) MONOVALENT INORGANIC CATION TRANSPORT, (44) (GO) SMALL MOLECULE METABOLIC PROCESS, (45) (GO) MONOVALENT INORGANIC CATION TRANSMEMBRANE TRANSPORTER ACTIVITY, (46) (GO) CATION TRANSMEMBRANE TRANSPORT, (47) (GO) OXIDOREDUCTASE ACTIVITY, (48) (GO) TRANSMEMBRANE TRANSPORT, (49) (GO) INORGANIC ION TRANSMEMBRANE TRANSPORT, (50) (GO) ION TRANSMEMBRANE TRANSPORT, (51) (GO) COFACTOR METABOLIC PROCESS, (52) (GO) ORGANIC ACID METABOLIC PROCESS, (53) (GO) CATION TRANSMEMBRANE TRANSPORTER ACTIVITY, (54) (GO) DICARBOXYLIC ACID METABOLIC PROCESS, (55) (GO) REGULATION OF MEMBRANE POTENTIAL, (56) (GO) ION TRANSMEMBRANE TRANSPORTER ACTIVITY, (57) (GO) CATION TRANSPORT, (58) (GO) COFACTOR BINDING, (59) (GO) TRANSPORTER COMPLEX, (60) (GO) TRANSPORTER ACTIVITY, (61) (GO) ION TRANSPORT, (62) (GO) CATION CHANNEL ACTIVITY, (63) (GO) NEUROTRANSMITTER RECEPTOR ACTIVITY

Table S17. Jointly deregulated pathways in PD and STCA.

	**Up_STCA**	**Down_STCA**
**Up_PD**	(1) (H)K INFLAMMATORY RESPONSE, (2) (H)K IL6 JAK STAT3 SIGNALING, (3) (H)K TNFA SIGNALING VIA NFKB, (4) (H)K IL2 STAT5 SIGNALING, (5) (H)K INTERFERON GAMMA RESPONSE, (6) (H)K INTERFERON ALPHA RESPONSE, (7)	

	(R) EXTRACELLULAR MATRIX ORGANIZATION, (8) (R) COLLAGEN FORMATION, (9) (K) PATHWAYS IN CANCER, (10) (R) INTEGRIN CELL SURFACE INTERACTIONS, (11) (R) COLLAGEN BIOSYNTHESIS AND MODIFYING ENZYMES, (12) (PID) E2F PATHWAY, (13) (R) RNA POLYMERASE II TRANSCRIPTION, (14) (R) CHROMATIN MODIFYING ENZYMES, (15) (R) RESPONSE OF EIF2AK4 GCN2 TO AMINO ACID DEFICIENCY, (16) (R) EUKARYOTIC TRANSLATION ELONGATION, (17) (GO) ADAPTIVE IMMUNE RESPONSE, (18) (GO) ANATOMICAL STRUCTURE FORMATION INVOLVED IN MORPHOGENESIS, (19) (GO) BIOLOGICAL ADHESION, (20) (GO) BLOOD VESSEL MORPHOGENESIS, (21) (GO) CARDIOVASCULAR SYSTEM DEVELOPMENT, (22) (GO) CELL ACTIVATION, (23) (GO) CELL MOTILITY, (24) (GO) CHROMOSOME, (25) (GO) CIRCULATORY SYSTEM DEVELOPMENT, (26) (GO) COLLAGEN CONTAINING EXTRACELLULAR MATRIX, (27) (GO) CYTOKINE MEDIATED SIGNALING PATHWAY, (28) (GO) CYTOKINE PRODUCTION, (29) (GO) DEFENSE RESPONSE, (30) (GO) EMBRYO DEVELOPMENT, (31) (GO) EXTRACELLULAR STRUCTURE ORGANIZATION, (32) (GO) IMMUNE SYSTEM DEVELOPMENT, (33) (GO) INFLAMMATORY RESPONSE, (34) (GO) LEUKOCYTE CELL CELL ADHESION, (35) (GO) LOCOMOTION, (36) (GO) LYMPHOCYTE ACTIVATION, (37) (GO) NEGATIVE REGULATION OF BIOSYNTHETIC PROCESS, (38) (GO) NEGATIVE REGULATION OF IMMUNE SYSTEM PROCESS, (39) (GO) NEGATIVE REGULATION OF NUCLEOBASE CONTAINING COMPOUND METABOLIC PROCESS, (40) (GO) NUCLEAR CHROMOSOME, (41) (GO) POSITIVE REGULATION OF CELLULAR BIOSYNTHETIC PROCESS, (42) (GO) POSITIVE REGULATION OF DEVELOPMENTAL PROCESS, (43) (GO) POSITIVE REGULATION OF IMMUNE SYSTEM PROCESS, (44) (GO) POSITIVE REGULATION OF NUCLEOBASE CONTAINING COMPOUND METABOLIC PROCESS, (45) (GO) POSITIVE REGULATION OF RNA METABOLIC PROCESS, (46) (GO) REGULATION OF CELL ACTIVATION, (47) (GO) REGULATION OF CELL ADHESION, (48) (GO) REGULATION OF CELL POPULATION PROLIFERATION, (49) (GO) REGULATION OF IMMUNE SYSTEM PROCESS, (50) (GO) REGULATION OF	

	LYMPHOCYTE ACTIVATION, (51) (GO) REGULATION OF VASCULATURE DEVELOPMENT, (52) (GO) RESPONSE TO BIOTIC STIMULUS, (53) (GO) SKELETAL SYSTEM DEVELOPMENT, (54) (GO) TUBE DEVELOPMENT, (55) (GO) TUBE MORPHOGENESIS, (56) (GO) T CELL ACTIVATION, (57) (GO) POSITIVE REGULATION OF CELL ACTIVATION, (58) (GO) RESPONSE TO CYTOKINE, (59) (GO) REGULATION OF T CELL ACTIVATION, (60) (GO) T CELL PROLIFERATION, (61) (GO) EMBRYO DEVELOPMENT ENDING IN BIRTH OR EGG HATCHING, (62) (GO) DEFENSE RESPONSE TO OTHER ORGANISM, (63) (GO) REGULATION OF IMMUNE RESPONSE, (64) (GO) REGULATION OF IMMUNE EFFECTOR PROCESS, (65) (GO) POSITIVE REGULATION OF INTRACELLULAR SIGNAL TRANSDUCTION, (66) (GO) NEGATIVE REGULATION OF CELL ACTIVATION, (67) (GO) MYELOID CELL DIFFERENTIATION, (68) (GO) ADAPTIVE IMMUNE RESPONSE BASED ON SOMATIC RECOMBINATION OF IMMUNE RECEPTORS BUILT FROM IMMUNOGLOBULIN SUPERFAMILY DOMAINS, (69) (GO) REGULATION OF CELLULAR COMPONENT MOVEMENT, (70) (GO) NEGATIVE REGULATION OF LYMPHOCYTE ACTIVATION, (71) (GO) POSITIVE REGULATION OF LOCOMOTION, (72) (GO) CELL SUBSTRATE ADHESION, (73) (GO) INNATE IMMUNE RESPONSE, (74) (GO) POSITIVE REGULATION OF IMMUNE RESPONSE, (75) (GO) COLLAGEN METABOLIC PROCESS, (76) (GO) POSITIVE REGULATION OF CYTOKINE PRODUCTION, (77) (GO) IMMUNE EFFECTOR PROCESS, (78) (GO) REGULATION OF LEUKOCYTE MEDIATED IMMUNITY, (79) (GO) REGULATION OF RESPONSE TO EXTERNAL STIMULUS, (80) (GO) ANCHORING JUNCTION, (81) (GO) LYMPHOCYTE ACTIVATION INVOLVED IN IMMUNE RESPONSE, (82) (GO) NEGATIVE REGULATION OF CELL ADHESION, (83) (GO) I KAPPAB KINASE NF KAPPAB SIGNALING, (84) (GO) PHA(GO)CYTOSIS, (85) (GO) CHROMATIN BINDING, (86) (GO) INTEGRIN MEDIATED SIGNALING PATHWAY, (87) (GO) REGULATION OF ADAPTIVE IMMUNE RESPONSE, (88) (GO) ACTIVATION OF IMMUNE RESPONSE, (89) (GO) REGULATION OF DEFENSE RESPONSE, (90) (GO) TRANSCRIPTION FACTOR BINDING, (91) (GO) REGULATION OF RESPONSE TO	

	CYTOKINE STIMULUS, (92) (GO) CELL SUBSTRATE JUNCTION, (93) (GO) MYELOID LEUKOCYTE ACTIVATION, (94) (GO) CELL ACTIVATION INVOLVED IN IMMUNE RESPONSE, (95) (GO) LEUKOCYTE MEDIATED IMMUNITY, (96) (GO) IN UTERO EMBRYONIC DEVELOPMENT, (97) (GO) REGULATION OF INTRACELLULAR SIGNAL TRANSDUCTION, (98) (GO) NEGATIVE REGULATION OF MULTI ORGANISM PROCESS, (99) (GO) TRANSCRIPTION COREGULATOR ACTIVITY, (100) (GO) REGULATION OF RESPONSE TO STRESS, (101) (GO) NUCLEAR SPECK, (102) (GO) CHROMOSOME ORGANIZATION, (103) (GO) INTERSPECIES INTERACTION BETWEEN ORGANISMS, (104) (GO) NUCLEAR BODY, (105) (GO) CHROMATIN ORGANIZATION, (106) (GO) COVALENT CHROMATIN MODIFICATION, (107) (GO) REGULATION OF GENE EXPRESSION EPIGENETIC	
Down_PD	(1) (H)K MTORC1 SIGNALING, (2) (K) PROTEASOME, (3) (R) APC C CDH1 MEDIATED DEGRADATION OF CDC20 AND OTHER APC C CDH1 TARGETED PROTEINS IN LATE MITOSIS EARLY G1, (4) (R) ASSEMBLY OF THE PRE REPLICATIVE COMPLEX, (5) (R) AUF1 HNRNP D0 BINDS AND DESTABILIZES MRNA, (6) (R) CELL CYCLE CHECKPOINTS, (7) (R) CROSS PRESENTATION OF SOLUBLE EXOGENOUS ANTIGENS ENDOSOMES, (8) (R) DEFECTIVE CFTR CAUSES CYSTIC FIBROSIS, (9) (R) DEGRADATION OF DVL, (10) (R) DEGRADATION OF GLI1 BY THE PROTEASOME, (11) (R) DNA REPLICATION, (12) (R) HEDGEHOG LIGAND BIOGENESIS, (13) (R) HIV INFECTION, (14) (R) HOST INTERACTIONS OF HIV FACTORS, (15) (R) MITOCHONDRIAL TRANSLATION, (16) (R) MITOTIC G2 G2 M PHASES, (17) (R) MITOTIC METAPHASE AND ANAPHASE, (18) (R) NEGATIVE REGULATION OF NOTCH4 SIGNALING, (19) (R) ORC1 REMOVAL FROM CHROMATIN, (20) (R) REGULATION OF PTEN STABILITY AND ACTIVITY, (21) (R) REGULATION OF RAS BY GAPS, (22) (R) REGULATION OF RUNX2 EXPRESSION AND ACTIVITY, (23) (R) SEPARATION OF SISTER CHROMATIDS, (24) (R) SWITCHING OF ORIGINS TO A POST REPLICATIVE STATE, (25) (R) DEGRADATION OF AXIN, (26) (R) METABOLISM OF POLYAMINES, (27) (R) THE ROLE OF GTSE1 IN G2 M PROGRESSION AFTER G2 CHECKPOINT, (28) (R) APC C MEDIATED	(1) (H)K OXIDATIVE PHOSPHORYLATION, (2) (H)K FATTY ACID METABOLISM, (3) (H)K ADIPOGENESIS, (4) (K) OXIDATIVE PHOSPHORYLATION, (5) (K) PARKINSONS DISEASE, (6) (R) RESPIRATORY ELECTRON TRANSPORT ATP SYNTHESIS BY CHEMIOSMOTIC COUPLING AND HEAT PRODUCTION BY UNCOUPLING PROTEINS, (7) (R) THE CITRIC ACID TCA CYCLE AND RESPIRATORY ELECTRON TRANSPORT, (8) (R) NEURONAL SYSTEM, (9) (R) PYRUVATE METABOLISM AND CITRIC ACID TCA CYCLE, (10) (GO) ATP SYNTHESIS COUPLED ELECTRON TRANSPORT, (11) (GO) ENERGY DERIVATION BY OXIDATION OF ORGANIC COMPOUNDS, (12) (GO) NADH DEHYDROGENASE COMPLEX, (13) (GO) OXIDATIVE PHOSPHORYLATION, (14) (GO) RESPIRASOME, (15) (GO) RESPIRATORY CHAIN COMPLEX, (16) (GO) OXIDOREDUCTASE ACTIVITY ACTING ON NAD P H QUINONE OR SIMILAR COMPOUND AS ACCEPTOR, (17) (GO) OXIDATION REDUCTION PROCESS, (18) (GO) MONOVALENT INORGANIC CATION TRANSPORT, (19) (GO) SMALL MOLECULE METABOLIC PROCESS, (20) (GO) MONOVALENT INORGANIC CATION TRANSMEMBRANE TRANSPORTER ACTIVITY, (21) (GO) CATION TRANSMEMBRANE TRANSPORT, (22) (GO) OXIDOREDUCTASE ACTIVITY, (23) (GO) TRANSMEMBRANE TRANSPORT, (24) (GO) ION TRANSMEMBRANE

	DEGRADATION OF CELL CYCLE PROTEINS, (29) (R) CELLULAR RESPONSE TO HYPOXIA, (30) (R) DNA REPLICATION PRE INITIATION, (31) (R) STABILIZATION OF P53, (32) (R) SCF SKP2 MEDIATED DEGRADATION OF P27 P21, (33) (R) REGULATION OF RUNX3 EXPRESSION AND ACTIVITY, (34) (R) G2 M CHECKPOINTS, (35) (R) ASYMMETRIC LOCALIZATION OF PCP PROTEINS, (36) (R) DECTIN 1 MEDIATED NONCANONICAL NF KB SIGNALING, (37) (R) REGULATION OF MRNA STABILITY BY PROTEINS THAT BIND AU RICH ELEMENTS, (38) (R) ABC TRANSPORTER DISORDERS, (39) (R) HEDGEHOG OFF STATE, (40) (R) FCERI MEDIATED NF KB ACTIVATION, (41) (R) CYCLIN A CDK2 ASSOCIATED EVENTS AT S PHASE ENTRY, (42) (R) DOWNSTREAM SIGNALING EVENTS OF B CELL RECEPTOR BCR, (43) (R) G1 S DNA DAMAGE CHECKPOINTS, (44) (R) PCP CE PATHWAY, (45) (R) M PHASE, (46) (R) UCH PROTEINASES, (47) (R) MAPK6 MAPK4 SIGNALING, (48) (R) HEDGEHOG ON STATE, (49) (R) SIGNALING BY NOTCH4, (50) (R) DEGRADATION OF BETA CATENIN BY THE DESTRUCTION COMPLEX, (51) (R) NERVOUS SYSTEM DEVELOPMENT, (52) (R) TNFR2 NON CANONICAL NF KB PATHWAY, (53) (R) BETA CATENIN INDEPENDENT WNT SIGNALING, (54) (GO) ANAPHASE PROMOTING COMPLEX DEPENDENT CATABOLIC PROCESS, (55) (GO) CELLULAR PROTEIN CONTAINING COMPLEX ASSEMBLY, (56) (GO) ENDOPEPTIDASE COMPLEX, (57) (GO) HYDROLASE ACTIVITY ACTING ON ACID ANHYDRIDES, (58) (GO) INTRACELLULAR TRANSPORT, (59) (GO) MITOCHONDRIAL GENE EXPRESSION, (60) (GO) MITOCHONDRIAL TRANSLATION, (61) (GO) MITOCHONDRIAL TRANSLATIONAL TERMINATION, (62) (GO) ORGANELLAR RIBOSOME, (63) (GO) PEPTIDASE COMPLEX, (64) (GO) PROTEIN CONTAINING COMPLEX ASSEMBLY, (65) (GO) TRANSLATIONAL TERMINATION, (66) (GO) REGULATION OF CELLULAR AMINO ACID METABOLIC PROCESS, (67) (GO) CELL CYCLE G2 M PHASE TRANSITION, (68) (GO) REGULATION OF CELLULAR AMINE METABOLIC PROCESS, (69) (GO) ORGANELLE LOCALIZATION, (70) (GO) PROTEASOME ACCESSORY COMPLEX, (71) (GO) ESTABLISHMENT OF ORGANELLE LOCALIZATION	TRANSPORT, (25) (GO) COFACTOR METABOLIC PROCESS, (26) (GO) ORGANIC ACID METABOLIC PROCESS, (27) (GO) CATION CHANNEL COMPLEX, (28) (GO) CATION TRANSMEMBRANE TRANSPORTER ACTIVITY, (29) (GO) REGULATION OF MEMBRANE POTENTIAL, (30) (GO) ION TRANSMEMBRANE TRANSPORTER ACTIVITY, (31) (GO) CATION TRANSPORT, (32) (GO) COFACTOR BINDING, (33) (GO) TRANSPORTER COMPLEX, (34) (GO) TRANSPORTER ACTIVITY, (35) (GO) ION TRANSPORT, (36) (GO) CATION CHANNEL ACTIVITY

Table S18. Jointly deregulated pathways in PD and THCA.

	Up_THCA	Down_THCA
Up_PD	(1) (H)K INFLAMMATORY RESPONSE, (2) (H)K IL6 JAK STAT3 SIGNALING, (3) (H)K TNFA SIGNALING VIA NFKB, (4) (H)K IL2 STAT5 SIGNALING, (5) (H)K INTERFERON GAMMA RESPONSE, (6) (H)K P53 PATHWAY, (7) (H)K INTERFERON ALPHA RESPONSE, (8) (K) CYTOKINE CYTOKINE RECEPTOR INTERACTION, (9) (R) EXTRACELLULAR MATRIX ORGANIZATION, (10) (R) COLLAGEN FORMATION, (11) (R) INTEGRIN CELL SURFACE INTERACTIONS, (12) (R) COLLAGEN BIOSYNTHESIS AND MODIFYING ENZYMES, (13) (GO) ADAPTIVE IMMUNE RESPONSE, (14) (GO) ANATOMICAL STRUCTURE FORMATION INVOLVED IN MORPHOGENESIS, (15) (GO) BIOLOGICAL ADHESION, (16) (GO) BLOOD VESSEL MORPHOGENESIS, (17) (GO) CARDIOVASCULAR SYSTEM DEVELOPMENT, (18) (GO) CELL ACTIVATION, (19) (GO) CELL CELL ADHESION, (20) (GO) CELL MOTILITY, (21) (GO) CIRCULATORY SYSTEM DEVELOPMENT, (22) (GO) COLLAGEN CONTAINING EXTRACELLULAR MATRIX, (23) (GO) CYTOKINE MEDIATED SIGNALING PATHWAY, (24) (GO) CYTOKINE PRODUCTION, (25) (GO) DEFENSE RESPONSE, (26) (GO) EMBRYO DEVELOPMENT, (27) (GO) EXTRACELLULAR STRUCTURE ORGANIZATION, (28) (GO) INFLAMMATORY RESPONSE, (29) (GO) LOCOMOTION, (30) (GO) NEGATIVE REGULATION OF MULTICELLULAR ORGANISMAL PROCESS, (31) (GO) POSITIVE REGULATION OF DEVELOPMENTAL PROCESS, (32) (GO) POSITIVE REGULATION OF IMMUNE SYSTEM PROCESS, (33) (GO) REGULATION OF CELL ACTIVATION, (34) (GO) REGULATION OF CELL ADHESION, (35) (GO) REGULATION OF CELL POPULATION PROLIFERATION, (36) (GO) REGULATION OF IMMUNE SYSTEM PROCESS, (37) (GO) REGULATION OF VASCULATURE DEVELOPMENT, (38) (GO) RESPONSE TO BACTERIUM, (39) (GO) RESPONSE TO BIOTIC STIMULUS, (40) (GO) TUBE DEVELOPMENT, (41) (GO) TUBE MORPHOGENESIS, (42) (GO) RESPONSE TO CYTOKINE, (43) (GO) EMBRYO DEVELOPMENT ENDING IN BIRTH OR EGG HATCHING, (44) (GO) DEFENSE RESPONSE TO OTHER ORGANISM, (45) (GO) REGULATION OF IMMUNE	(1) (R) RNA POLYMERASE II TRANSCRIPTION, (2) (R) RESPONSE OF EIF2AK4 GCN2 TO AMINO ACID DEFICIENCY, (3) (R) EUKARYOTIC TRANSLATION ELONGATION, (4) (GO) DNA BINDING TRANSCRIPTION FACTOR ACTIVITY, (5) (GO) TRANSCRIPTION REGULATOR ACTIVITY

	RESPONSE, (46) (GO) POSITIVE REGULATION OF INTRACELLULAR SIGNAL TRANSDUCTION, (47) (GO) TISSUE REMODELING, (48) (GO) REGULATION OF CELLULAR COMPONENT MOVEMENT, (49) (GO) POSITIVE REGULATION OF LOCOMOTION, (50) (GO) CELL SUBSTRATE ADHESION, (51) (GO) INNATE IMMUNE RESPONSE, (52) (GO) POSITIVE REGULATION OF IMMUNE RESPONSE, (53) (GO) COLLAGEN METABOLIC PROCESS, (54) (GO) POSITIVE REGULATION OF CYTOKINE PRODUCTION, (55) (GO) IMMUNE EFFECTOR PROCESS, (56) (GO) REGULATION OF RESPONSE TO EXTERNAL STIMULUS, (57) (GO) ANCHORING JUNCTION, (58) (GO) I KAPPAB KINASE NF KAPPAB SIGNALING, (59) (GO) PHA(GO)CYTOSIS, (60) (GO) INTEGRIN MEDIATED SIGNALING PATHWAY, (61) (GO) ACTIVATION OF IMMUNE RESPONSE, (62) (GO) REGULATION OF DEFENSE RESPONSE, (63) (GO) CELL JUNCTION ORGANIZATION, (64) (GO) CELL SUBSTRATE JUNCTION, (65) (GO) POSITIVE REGULATION OF I KAPPAB KINASE NF KAPPAB SIGNALING, (66) (GO) MYELOID LEUKOCYTE ACTIVATION, (67) (GO) CELL ACTIVATION INVOLVED IN IMMUNE RESPONSE, (68) (GO) ENTRY INTO HOST, (69) (GO) LEUKOCYTE MEDIATED IMMUNITY, (70) (GO) CELL JUNCTION ASSEMBLY, (71) (GO) REGULATION OF INTRACELLULAR SIGNAL TRANSDUCTION, (72) (GO) REGULATION OF RESPONSE TO STRESS, (73) (GO) RUFFLE	
Down_PD	(1) (H)K MTORC1 SIGNALING, (2) (R) CELL CYCLE CHECKPOINTS, (3) (R) G2 M CHECKPOINTS, (4) (R) M PHASE, (5) (R) MHC CLASS II ANTIGEN PRESENTATION, (6) (R) NERVOUS SYSTEM DEVELOPMENT, (7) (GO) GLUTAMATERGIC SYNAPSE, (8) (GO) NEURON PROJECTION, (9) (GO) POSTSYNAPSE, (10) (GO) SOMATODENDRITIC COMPARTMENT, (11) (GO) SYNAPSE, (12) (GO) SYNAPTIC MEMBRANE, (13) (GO) SYNAPTIC SIGNALING, (14) (GO) DENDRITIC TREE, (15) (GO) REGULATION OF TRANS SYNAPTIC SIGNALING, (16) (GO) SYNAPSE ORGANIZATION, (17) (GO) REGULATION OF SYNAPSE STRUCTURE OR ACTIVITY, (18) (GO) NEURON DEVELOPMENT, (19) (GO) CELL PART MORPHOGENESIS, (20) (GO) CELL MORPHOGENESIS INVOLVED IN	(1) (H)K OXIDATIVE PHOSPHORYLATION, (2) (H)K ADIPOGENESIS, (3) (K) HUNTINGTONS DISEASE, (4) (K) OXIDATIVE PHOSPHORYLATION, (5) (K) PARKINSONS DISEASE, (6) (R) COMPLEX I BIOGENESIS, (7) (R) RESPIRATORY ELECTRON TRANSPORT, (8) (R) RESPIRATORY ELECTRON TRANSPORT ATP SYNTHESIS BY CHEMIOSMOTIC COUPLING AND HEAT PRODUCTION BY UNCOUPLING PROTEINS, (9) (R) THE CITRIC ACID TCA CYCLE AND RESPIRATORY ELECTRON TRANSPORT, (10) (R) PROTEIN LOCALIZATION, (11) (R) CRISTAE FORMATION, (12) (R) FORMATION OF ATP BY CHEMIOSMOTIC COUPLING, (13) (GO) ATP METABOLIC PROCESS, (14) (GO) ATP SYNTHESIS COUPLED ELECTRON TRANSPORT, (15) (GO) CELLULAR

	NEURON DIFFERENTIATION, (21) (GO) CELL CELL SIGNALING	RESPIRATION, (16) (GO) ELECTRON TRANSPORT CHAIN, (17) (GO) ENERGY DERIVATION BY OXIDATION OF ORGANIC COMPOUNDS, (18) (GO) ENVELOPE, (19) (GO) GENERATION OF PRECURSOR METABOLITES AND ENERGY, (20) (GO) INNER MITOCHONDRIAL MEMBRANE PROTEIN COMPLEX, (21) (GO) INTRACELLULAR TRANSPORT, (22) (GO) MITOCHONDRIAL ELECTRON TRANSPORT NADH TO UBIQUINONE, (23) (GO) MITOCHONDRIAL ENVELOPE, (24) (GO) MITOCHONDRIAL MATRIX, (25) (GO) MITOCHONDRIAL PROTEIN COMPLEX, (26) (GO) MITOCHONDRIAL RESPIRATORY CHAIN COMPLEX ASSEMBLY, (27) (GO) MITOCHONDRION, (28) (GO) MITOCHONDRION ORGANIZATION, (29) (GO) NADH DEHYDROGENASE ACTIVITY, (30) (GO) NADH DEHYDROGENASE COMPLEX, (31) (GO) NADH DEHYDROGENASE COMPLEX ASSEMBLY, (32) (GO) ORGANELLE INNER MEMBRANE, (33) (GO) OXIDATIVE PHOSPHORYLATION, (34) (GO) RESPIRASOME, (35) (GO) RESPIRATORY CHAIN COMPLEX, (36) (GO) RESPIRATORY ELECTRON TRANSPORT CHAIN, (37) (GO) OXIDOREDUCTASE ACTIVITY ACTING ON NAD P H QUINONE OR SIMILAR COMPOUND AS ACCEPTOR, (38) (GO) OXIDATION REDUCTION PROCESS, (39) (GO) CRISTAE FORMATION, (40) (GO) ATP SYNTHESIS COUPLED PROTON TRANSPORT, (41) (GO) PROTON TRANSPORTING ATP SYNTHASE COMPLEX, (42) (GO) OXIDOREDUCTASE ACTIVITY

Table S19. Jointly deregulated pathways in PD and BRCA.

	Up_BRCA	Down_BRCA
Up_PD	(1) (H)K INTERFERON GAMMA RESPONSE, (2) (H)K INTERFERON ALPHA RESPONSE, (3) (PID) E2F PATHWAY, (4) (R) RNA POLYMERASE II TRANSCRIPTION, (5) (R) CHROMATIN MODIFYING ENZYMES, (6) (GO) CHROMOSOME, (7) (GO) NUCLEAR CHROMOSOME, (8) (GO) DEFENSE RESPONSE TO OTHER ORGANISM, (9) (GO) REGULATION OF IMMUNE RESPONSE, (10) (GO) INNATE IMMUNE RESPONSE, (11) (GO) IMMUNE EFFECTOR PROCESS, (12) (GO) CHROMATIN BINDING, (13) (GO) CHROMOSOME ORGANIZATION, (14) (GO) CHROMATIN ORGANIZATION, (15) (GO)	(1) (H)K TNFA SIGNALING VIA NFKB, (2) (R) RESPONSE OF EIF2AK4 GCN2 TO AMINO ACID DEFICIENCY, (3) (R) EUKARYOTIC TRANSLATION ELONGATION, (4) (GO) ANATOMICAL STRUCTURE FORMATION INVOLVED IN MORPHOGENESIS, (5) (GO) BIOLOGICAL ADHESION, (6) (GO) BLOOD VESSEL MORPHOGENESIS, (7) (GO) CARDIOVASCULAR SYSTEM DEVELOPMENT, (8) (GO) CELL MOTILITY, (9) (GO) CIRCULATORY SYSTEM DEVELOPMENT, (10) (GO) COLLAGEN CONTAINING EXTRACELLULAR MATRIX, (11) (GO) DNA BINDING TRANSCRIPTION ACTIVATOR ACTIVITY, (12) (GO) DNA

	REGULATION OF GENE EXPRESSION EPIGENETIC	BINDING TRANSCRIPTION FACTOR ACTIVITY RNA POLYMERASE II SPECIFIC, (13) (GO) LOCOMOTION, (14) (GO) NEGATIVE REGULATION OF MULTICELLULAR ORGANISMAL PROCESS, (15) (GO) POSITIVE REGULATION OF DEVELOPMENTAL PROCESS, (16) (GO) POSITIVE REGULATION OF TRANSCRIPTION BY RNA POLYMERASE II, (17) (GO) POSITIVE REGULATION OF VASCULATURE DEVELOPMENT, (18) (GO) REGULATION OF CELL POPULATION PROLIFERATION, (19) (GO) REGULATION OF VASCULATURE DEVELOPMENT, (20) (GO) TUBE DEVELOPMENT, (21) (GO) TUBE MORPHOGENESIS, (22) (GO) MUSCLE STRUCTURE DEVELOPMENT, (23) (GO) REGULATION OF CELLULAR COMPONENT MOVEMENT, (24) (GO) POSITIVE REGULATION OF LOCOMOTION, (25) (GO) CELL SUBSTRATE ADHESION, (26) (GO) ENDOTHELIUM DEVELOPMENT, (27) (GO) ANCHORING JUNCTION, (28) (GO) NEGATIVE REGULATION OF CELL POPULATION PROLIFERATION, (29) (GO) CELL JUNCTION ORGANIZATION, (30) (GO) CELL SUBSTRATE JUNCTION, (31) (GO) CELL JUNCTION ASSEMBLY, (32) (GO) REGULATION OF INTRACELLULAR SIGNAL TRANSDUCTION
Down_PD	(1) (H)K MTORC1 SIGNALING, (2) (K) PROTEASOME, (3) (R) APC C CDH1 MEDIATED DEGRADATION OF CDC20 AND OTHER APC C CDH1 TARGETED PROTEINS IN LATE MITOSIS EARLY G1, (4) (R) ASPARAGINE N LINKED GLYCOSYLATION, (5) (R) ASSEMBLY OF THE PRE REPLICATIVE COMPLEX, (6) (R) CELL CYCLE CHECKPOINTS, (7) (R) DNA REPLICATION, (8) (R) HIV INFECTION, (9) (R) HOST INTERACTIONS OF HIV FACTORS, (10) (R) MITOCHONDRIAL TRANSLATION, (11) (R) MITOTIC G2 G2 M PHASES, (12) (R) MITOTIC METAPHASE AND ANAPHASE, (13) (R) ORC1 REMOVAL FROM CHROMATIN, (14) (R) SEPARATION OF SISTER CHROMATIDS, (15) (R) SWITCHING OF ORIGINS TO A POST REPLICATIVE STATE, (16) (R) METABOLISM OF POLYAMINES, (17) (R) THE ROLE OF GTSE1 IN G2 M PROGRESSION AFTER G2 CHECKPOINT, (18) (R) APC C MEDIATED DEGRADATION OF CELL CYCLE PROTEINS, (19) (R) DNA REPLICATION PRE INITIATION, (20) (R) STABILIZATION OF P53, (21) (R) SCF SKP2 MEDIATED DEGRADATION OF P27 P21, (22) (R) G2 M CHECKPOINTS, (23) (R) CYCLIN A CDK2 ASSOCIATED EVENTS	(1) (H)K ADIPOGENESIS, (2) (GO) SYNAPSE, (3) (GO) CELL CELL SIGNALING

	AT S PHASE ENTRY, (24) (R) G1 S DNA DAMAGE CHECKPOINTS, (25) (R) M PHASE, (26) (R) UCH PROTEINASES, (27) (R) (GO)LGI TO ER RETROGRADE TRANSPORT, (28) (R) MHC CLASS II ANTIGEN PRESENTATION, (29) (R) COPI DEPENDENT (GO)LGI TO ER RETROGRADE TRAFFIC, (30) (R) GLUCOSE METABOLISM, (31) (GO) ANAPHASE PROMOTING COMPLEX DEPENDENT CATABOLIC PROCESS, (32) (GO) CELLULAR PROTEIN CONTAINING COMPLEX ASSEMBLY, (33) (GO) ENDOPEPTIDASE COMPLEX, (34) (GO) HYDROLASE ACTIVITY ACTING ON ACID ANHYDRIDES, (35) (GO) MITOCHONDRIAL GENE EXPRESSION, (36) (GO) MITOCHONDRIAL TRANSLATION, (37) (GO) PROTEIN CONTAINING COMPLEX ASSEMBLY, (38) (GO) CELL CYCLE G2 M PHASE TRANSITION, (39) (GO) ORGANELLE LOCALIZATION, (40) (GO) ESTABLISHMENT OF ORGANELLE LOCALIZATION	

Table S20. Jointly deregulated pathways in PD and CLL.

	Up_CLL	Down_CLL
Up_PD	(1) (R) RNA POLYMERASE II TRANSCRIPTION	(1) (H)K INFLAMMATORY RESPONSE, (2) (H)K TNFA SIGNALING VIA NFKB, (3) (GO) BIOLOGICAL ADHESION, (4) (GO) CELL ACTIVATION, (5) (GO) CELL MOTILITY, (6) (GO) EPITHELIAL CELL DIFFERENTIATION, (7) (GO) LOCOMOTION, (8) (GO) LYMPHOCYTE ACTIVATION, (9) (GO) RESPONSE TO CYTOKINE
Down_PD	(1) , (0)	(1) (H)K MTORC1 SIGNALING

Table S21. Jointly deregulated pathways in PD and LGCA.

	Up_LGCA	Down_LGCA
Up_PD	(1) (GO) CHROMOSOME, (2) (GO) CHROMOSOME ORGANIZATION	(1) (H)K INFLAMMATORY RESPONSE, (2) (H)K TNFA SIGNALING VIA NFKB, (3) (H)K IL2 STAT5 SIGNALING, (4) (H)K INTERFERON GAMMA RESPONSE, (5) (H)K INTERFERON ALPHA RESPONSE, (6) (K) CYTOKINE CYTOKINE RECEPTOR INTERACTION, (7) (GO) ADAPTIVE IMMUNE RESPONSE, (8) (GO) ANATOMICAL STRUCTURE FORMATION INVOLVED IN MORPHOGENESIS, (9) (GO) BIOLOGICAL ADHESION, (10) (GO) BLOOD VESSEL MORPHOGENESIS, (11) (GO) CARDIOVASCULAR SYSTEM DEVELOPMENT, (12) (GO) CELL

		ACTIVATION, (13) (GO) CELL CELL ADHESION, (14) (GO) CELL MOTILITY, (15) (GO) CIRCULATORY SYSTEM DEVELOPMENT, (16) (GO) CYTOKINE MEDIATED SIGNALING PATHWAY, (17) (GO) CYTOKINE PRODUCTION, (18) (GO) DEFENSE RESPONSE, (19) (GO) IMMUNE RECEPTOR ACTIVITY, (20) (GO) IMMUNE SYSTEM DEVELOPMENT, (21) (GO) INFLAMMATORY RESPONSE, (22) (GO) LEUKOCYTE CELL CELL ADHESION, (23) (GO) LEUKOCYTE DIFFERENTIATION, (24) (GO) LOCOMOTION, (25) (GO) LYMPHOCYTE ACTIVATION, (26) (GO) NEGATIVE REGULATION OF IMMUNE SYSTEM PROCESS, (27) (GO) NEGATIVE REGULATION OF MULTICELLULAR ORGANISMAL PROCESS, (28) (GO) POSITIVE REGULATION OF DEVELOPMENTAL PROCESS, (29) (GO) POSITIVE REGULATION OF IMMUNE SYSTEM PROCESS, (30) (GO) POSITIVE REGULATION OF VASCULATURE DEVELOPMENT, (31) (GO) REGULATION OF CELL ACTIVATION, (32) (GO) REGULATION OF CELL ADHESION, (33) (GO) REGULATION OF CELL POPULATION PROLIFERATION, (34) (GO) REGULATION OF IMMUNE SYSTEM PROCESS, (35) (GO) REGULATION OF VASCULATURE DEVELOPMENT, (36) (GO) RESPONSE TO BACTERIUM, (37) (GO) RESPONSE TO BIOTIC STIMULUS, (38) (GO) RESPONSE TO MOLECULE OF BACTERIAL ORIGIN, (39) (GO) TUBE DEVELOPMENT, (40) (GO) TUBE MORPHOGENESIS, (41) (GO) T CELL ACTIVATION, (42) (GO) REGULATION OF LEUKOCYTE DIFFERENTIATION, (43) (GO) NEGATIVE REGULATION OF CYTOKINE PRODUCTION, (44) (GO) POSITIVE REGULATION OF CELL ACTIVATION, (45) (GO) RESPONSE TO CYTOKINE, (46) (GO) CYTOKINE BINDING, (47) (GO) MUSCLE STRUCTURE DEVELOPMENT, (48) (GO) DEFENSE RESPONSE TO OTHER ORGANISM, (49) (GO) REGULATION OF IMMUNE RESPONSE, (50) (GO) REGULATION OF IMMUNE EFFECTOR PROCESS, (51) (GO) POSITIVE REGULATION OF INTRACELLULAR SIGNAL TRANSDUCTION, (52) (GO) TUMOR NECROSIS FACTOR SUPERFAMILY CYTOKINE PRODUCTION, (53) (GO) NEGATIVE REGULATION OF CELL ACTIVATION, (54) (GO) CELLULAR RESPONSE TO BIOTIC STIMULUS, (55) (GO) MYELOID CELL DIFFERENTIATION, (56) (GO) ADAPTIVE IMMUNE RESPONSE BASED ON SOMATIC RECOMBINATION OF IMMUNE RECEPTORS BUILT FROM

		IMMUNOGLOBULIN SUPERFAMILY DOMAINS, (57) (GO) REGULATION OF CELLULAR COMPONENT MOVEMENT, (58) (GO) POSITIVE REGULATION OF LOCOMOTION, (59) (GO) CELL SUBSTRATE ADHESION, (60) (GO) INNATE IMMUNE RESPONSE, (61) (GO) POSITIVE REGULATION OF IMMUNE RESPONSE, (62) (GO) IMMUNE EFFECTOR PROCESS, (63) (GO) REGULATION OF LEUKOCYTE MEDIATED IMMUNITY, (64) (GO) REGULATION OF RESPONSE TO EXTERNAL STIMULUS, (65) (GO) POSITIVE REGULATION OF LEUKOCYTE DIFFERENTIATION, (66) (GO) ENDOTHELIUM DEVELOPMENT, (67) (GO) ANCHORING JUNCTION, (68) (GO) NEGATIVE REGULATION OF CELL POPULATION PROLIFERATION, (69) (GO) NEGATIVE REGULATION OF CELL ADHESION, (70) (GO) PHA(GO)CYTOSIS, (71) (GO) CELLULAR RESPONSE TO VASCULAR ENDOTHELIAL GROWTH FACTOR STIMULUS, (72) (GO) REGULATION OF ADAPTIVE IMMUNE RESPONSE, (73) (GO) ACTIVATION OF IMMUNE RESPONSE, (74) (GO) REGULATION OF DEFENSE RESPONSE, (75) (GO) REGULATION OF LYMPHOCYTE MEDIATED IMMUNITY, (76) (GO) CELL JUNCTION ORGANIZATION, (77) (GO) CELL SUBSTRATE JUNCTION, (78) (GO) MYELOID LEUKOCYTE ACTIVATION, (79) (GO) CELL ACTIVATION INVOLVED IN IMMUNE RESPONSE, (80) (GO) LEUKOCYTE MEDIATED IMMUNITY, (81) (GO) CELL JUNCTION ASSEMBLY, (82) (GO) REGULATION OF INTRACELLULAR SIGNAL TRANSDUCTION, (83) (GO) REGULATION OF RESPONSE TO STRESS
Down_PD	(1) (H)K MTORC1 SIGNALING, (2) (R) APC C CDH1 MEDIATED DEGRADATION OF CDC20 AND OTHER APC C CDH1 TARGETED PROTEINS IN LATE MITOSIS EARLY G1, (3) (R) ASSEMBLY OF THE PRE REPLICATIVE COMPLEX, (4) (R) CELL CYCLE CHECKPOINTS, (5) (R) DNA REPLICATION, (6) (R) HIV INFECTION, (7) (R) MITOCHONDRIAL PROTEIN IMPORT, (8) (R) MITOCHONDRIAL TRANSLATION, (9) (R) MITOTIC G2 G2 M PHASES, (10) (R) MITOTIC METAPHASE AND ANAPHASE, (11) (R) ORC1 REMOVAL FROM CHROMATIN, (12) (R) SEPARATION OF SISTER CHROMATIDS, (13) (R) SWITCHING OF ORIGINS TO A POST REPLICATIVE STATE, (14) (R) THE CITRIC ACID TCA CYCLE AND RESPIRATORY ELECTRON TRANSPORT, (15) (R) METABOLISM OF POLYAMINES, (16) (R) THE ROLE OF GTSE1 IN G2 M	(1) (GO) MICROTUBULE BASED MOVEMENT, (2) (GO) CYTOPLASMIC REGION, (3) (GO) CELL PART MORPHOGENESIS, (4) (GO) PLASMA MEMBRANE BOUNDED CELL PROJECTION CYTOPLASM

	PROGRESSION AFTER G2 CHECKPOINT, (17) (R) APC C MEDIATED DEGRADATION OF CELL CYCLE PROTEINS, (18) (R) DNA REPLICATION PRE INITIATION, (19) (R) STABILIZATION OF P53, (20) (R) SCF SKP2 MEDIATED DEGRADATION OF P27 P21, (21) (R) G2 M CHECKPOINTS, (22) (R) G1 S DNA DAMAGE CHECKPOINTS, (23) (R) M PHASE, (24) (R) (GO)LGI TO ER RETROGRADE TRANSPORT, (25) (R) COPI DEPENDENT (GO)LGI TO ER RETROGRADE TRAFFIC, (26) (GO) ANAPHASE PROMOTING COMPLEX DEPENDENT CATABOLIC PROCESS, (27) (GO) ENDOPEPTIDASE COMPLEX, (28) (GO) ENVELOPE, (29) (GO) MITOCHONDRIAL ENVELOPE, (30) (GO) MITOCHONDRIAL GENE EXPRESSION, (31) (GO) MITOCHONDRIAL MATRIX, (32) (GO) MITOCHONDRIAL PROTEIN COMPLEX, (33) (GO) MITOCHONDRIAL TRANSLATION, (34) (GO) MITOCHONDRIAL TRANSLATIONAL TERMINATION, (35) (GO) MITOCHONDRION, (36) (GO) ORGANELLAR RIBOSOME, (37) (GO) ORGANELLE INNER MEMBRANE, (38) (GO) TRANSLATIONAL ELONGATION, (39) (GO) TRANSLATIONAL TERMINATION, (40) (GO) CELLULAR AMINO ACID METABOLIC PROCESS, (41) (GO) CELL CYCLE G2 M PHASE TRANSITION, (42) (GO) COFACTOR BINDING	

Table S22. Jointly deregulated pathways in PD and PRCA.

	Up_PRCA	Down_PRCA
Up_PD	(1) (R) RESPONSE OF EIF2AK4 GCN2 TO AMINO ACID DEFICIENCY, (2) (R) EUKARYOTIC TRANSLATION ELONGATION, (3) (GO) CHROMOSOME ORGANIZATION	(1) (R) EXTRACELLULAR MATRIX ORGANIZATION, (2) (GO) ANATOMICAL STRUCTURE FORMATION INVOLVED IN MORPHOGENESIS, (3) (GO) BIOLOGICAL ADHESION, (4) (GO) BLOOD VESSEL MORPHOGENESIS, (5) (GO) CARDIOVASCULAR SYSTEM DEVELOPMENT, (6) (GO) CELL MOTILITY, (7) (GO) CIRCULATORY SYSTEM DEVELOPMENT, (8) (GO) COLLAGEN CONTAINING EXTRACELLULAR MATRIX, (9) (GO) EMBRYO DEVELOPMENT, (10) (GO) EPITHELIAL CELL DIFFERENTIATION, (11) (GO) EXTRACELLULAR STRUCTURE ORGANIZATION, (12) (GO) LOCOMOTION, (13) (GO) NEGATIVE REGULATION OF MULTICELLULAR ORGANISMAL PROCESS, (14) (GO) POSITIVE REGULATION OF DEVELOPMENTAL PROCESS, (15) (GO) REGULATION OF CELL ADHESION, (16)

		(GO) REGULATION OF CELL POPULATION PROLIFERATION, (17) (GO) REGULATION OF VASCULATURE DEVELOPMENT, (18) (GO) SKELETAL SYSTEM DEVELOPMENT, (19) (GO) TUBE DEVELOPMENT, (20) (GO) TUBE MORPHOGENESIS, (21) (GO) MUSCLE STRUCTURE DEVELOPMENT, (22) (GO) REGULATION OF CELLULAR COMPONENT MOVEMENT, (23) (GO) POSITIVE REGULATION OF LOCOMOTION, (24) (GO) CELL SUBSTRATE ADHESION, (25) (GO) ANCHORING JUNCTION, (26) (GO) NEGATIVE REGULATION OF CELL POPULATION PROLIFERATION, (27) (GO) REGULATION OF OSSIFICATION, (28) (GO) CELL JUNCTION ORGANIZATION, (29) (GO) CELL SUBSTRATE JUNCTION, (30) (GO) CELL JUNCTION ASSEMBLY, (31) (GO) REGULATION OF INTRACELLULAR SIGNAL TRANSDUCTION, (32) (GO) NEGATIVE REGULATION OF CELLULAR RESPONSE TO GROWTH FACTOR STIMULUS
**Down_PD**	(1) (H)K MTORC1 SIGNALING, (2) (R) ASPARAGINE N LINKED GLYCOSYLATION, (3) (R) CELL CYCLE CHECKPOINTS, (4) (R) MITOCHONDRIAL TRANSLATION, (5) (R) MITOTIC METAPHASE AND ANAPHASE, (6) (R) G2 M CHECKPOINTS, (7) (R) M PHASE, (8) (GO) MITOCHONDRIAL GENE EXPRESSION, (9) (GO) MITOCHONDRIAL MATRIX, (10) (GO) MITOCHONDRIAL PROTEIN COMPLEX, (11) (GO) MITOCHONDRIAL TRANSLATION, (12) (GO) MITOCHONDRION, (13) (GO) CELLULAR AMINO ACID METABOLIC PROCESS, (14) (GO) SMALL MOLECULE METABOLIC PROCESS, (15) (GO) ORGANIC ACID METABOLIC PROCESS	(1) (GO) SYNAPSE, (2) (GO) NEURON DEVELOPMENT, (3) (GO) CELL PART MORPHOGENESIS, (4) (GO) CELL MORPHOGENESIS INVOLVED IN NEURON DIFFERENTIATION, (5) (GO) CELL CELL SIGNALING

Table S23. Number of consensus co-expression modules and modules significantly correlated with disease status found in each disorder.

10	Nº Modules	Nº Modules significantly associated with disease status	Nº Modules positively correlated with disease status	Nº Modules negatively correlated with disease status
AD	33	13	6	7
PD	20	8	5	3
ALL	16	8	3	5
AML	11	8	4	4
BLCA	15	11	4	7
BRCA	32	27	16	11
BRNCA	12	12	6	6
CERV	45	21	13	8
CHLCA	13	10	6	4
CLL	13	9	4	5
CML	58	10	5	5
CRCA	26	24	15	9

DLBCL	29	23	13	10
FLYMPH	31	18	15	3
HANC	8	6	3	3
KDNCA	13	13	8	5
LGCA	25	24	14	10
LIVCA	44	39	23	16
OVCA	75	39	23	16
PACA	48	37	20	17
PRCA	51	31	13	18
SKCM	23	19	12	7
STCA	9	8	5	3
THCA	41	30	13	17

Table S24. Disease identifiers employed to query the gene-disease association databases. Genes and variant-genes identified for each disorder using the stringent selection criterion, which are included in the interactome 1. Genes included in the largest connected component.

Disorder	IDs used in the different DBs.	Disease associated genes and variant-genes (stringent)	Genes included in the largest connected component in the interactome 1 stringent analysis
**AD**	**-DisGeNet MESH:** D000544<br>**-EDGAR:** 104300<br>**-PhenGeID trait:** Alzheimer Disease	SORL1, NOS3, ACE, PLAU, MPO, APP, PSEN1, APBB2, PAXIP1, BLMH, PSEN2, HFE, A2M, ADAM10, GSK3B, APOE, MAPT, TREM2, BACE1, IDE, IL1B, INSR, LEP, NPY, BCL2, BDNF, CASP3, IL6R, CR1, MRPL50P4, SPON1, MS4A2, MS4A6A, MIR6503, PICALM, MMP3, BCAS3, CYB561, ABCA7, PVRL2, TOMM40, APOC1, BIN1, ACKR2, CCRL2, LOC102724297, SUCLG2, SNAR-I, RANP6, LOC100289673, HLA-DRB1, CD2AP, GAPDHP15, BZW2, EPHA1-AS1, PTK2B, CLU, MIR6843, HNF4G, SLC16A9, MS4A4A, FNTAP1, MMP12, CYP27C1, OSTN, FBXO8, HLA-DQA1, RBBP4P4, LINC01111	PSEN1, APP, TOMM40, GSK3B, PSEN2, MAPT
**ASD**	**-DisGeNet MESH:** D000067877<br>**-EDGAR:** PS209850, 605309<br>**-PhenGeID** trait: Autism Spectrum Disorder∣Autistic Disorder∣Child Development Disorders, Pervasive∣ Asperger Syndrome	NLGN3, CHD8, MECP2, NLGN4X, SLC9A9, MET, EN2, CNTNAP2, RPL10, PTCHD1, EIF4E, SHANK2, TMLHE, PTEN, NRXN2, SHANK3, NRXN1, ITIH3, TRIM26	EIF4E
**BD**	**-DisGeNet MESH:** D001714<br>**-EDGAR:** NA<br>**-PhenGeID trait:** Bipolar Disorder	S100B, COMT, ANK3, CACNA1C, NCAN, SP4, ADCY2, POLG, LMAN2L, FADS2, DRD1, GAD1, NR3C1, GSK3B, ITIH1, MTHFR, BDNF, SLC6A4, CLOCK, ITIH3, TRIM26, MAD1L1	ANK3, NR3C1, GSK3B, CLOCK, BDNF
**HD**	**-DisGeNet MESH:** D006816<br>**-EDGAR:** 143100<br>**-PhenGeID trait:** NA	HTT	HTT
**MD**	**-DisGeNet MESH:** D003866<br>**-EDGAR:** 608516<br>**-PhenGeID trait:** Depression∣Depressive Disorder∣Depressive Disorder, Major	FKBP5, TPH2, APOE, DISC1, SLC6A4, FGFR1, SOD1, CRH, NR3C1, IL6, KCNK2, MTHFR, NPY, PTGS2, BDNF, S100A10, TOMM40, SEPT3, WBP2NL, CYP2D6, ITIH3, TRIM26, CYP2C9	NR3C1, BDNF
**PD**	**-DisGeNet MESH:** D010300<br>**-EDGAR:** PS168600<br>**-PhenGeID trait:** Parkinson Disease	GIGYF2, GBA, PODXL, PINK1, HTRA2, CHCHD2, LRRK2, PARK7, ATP13A2, PARK2, PLA2G6, SYNJ1,	CHCHD2, PARK7, PARK2, SNCA, PINK1

		DNAJC13, VPS13C, NR4A2, UCHL1, VPS35, SNCAIP, TBP, EIF4G1, GLUD2, ADH1C, MAPT, SNCA, ATXN2, ATXN3, FGF20, DDC, DRD2, MAOB, SLC18A2, TH, RAB25, NUCKS1, RAB29, TIAL1, INPP5F, SLC2A13, CNTN1, GCH1, TMEM229B, TPM1, BCKDK, CRHR1, SPPL2C, NSF, WNT3, RIT2, DCUN1D1, MCCC1, GAK, TMEM175, DGKQ, BST1, FAM47E-STBD1, LHFPL2, HLA-DRB1, HLA-DQB1, FAM126A, GPNMB, KRTCAP2, SLC41A1, SYT10, ACMSD, CERS6, DDRGK1, MMRN1, HLA-DRA, HLA-DQA1	
**SCZ**	**-DisGeNet MESH:** D012559 **-EDGAR:** 181500∣604906∣603013∣600850∣613950∣615232 **-PhenGeID trait:** Schizophrenia	DRD3, DISC1, MTHFR, DAO, COMT, SETD1A, AKT1, RTN4R, CHI3L1, DTNBP1, APOL4, SYN2, APOL2, MC4R, SLC1A1, SHANK3, PRODH, NRG1, MAGI2, CHRNA7, GRIN2B, NOS1, RELN, SRR, TCF4, NRXN1, SP4, PPP3R1, SYNGAP1, MDK, GRM5, GSK3B, ZDHHC8, APOE, NR4A2, SLC6A3, PPP1R1B, KMO	AKT1, GSK3B
**ALL**	**-DisGeNet MESH:** D054198 **-EDGAR:** 613065 **-PhenGeID trait:** Precursor B-Cell Lymphoblastic Leukemia-Lymphoma∣Precursor Cell Lymphoblastic Leukemia-Lymphoma	TAL1, TCF3, TAL2, BAX, PAX5, NBN, NUP214, FLT3, BCR, IKZF1, CDKN2A, ABL1, GATA3, LHPP, CEBPE, C14orf119	TCF3, ABL1, GATA3, TAL1, FLT3, BCR, CEBPE
**AML**	**-DisGeNet MESH:** D015470 **-EDGAR:** 601626 **-PhenGeID trait:** Myeloproliferative Disorders	*TGM6, SETBP1, SH3GL1, FLT3, CHIC2, CEBPA, NPM1, WHSC1L1, CBFB, JAK2, NUP214, TERT, MLF1, MLLT10, LPP, GATA2, KRAS, ETV6, DDX41, KIT, RUNX1, NSD1, PICALM, DNMT3A, PTPN11, IDH1, IDH2, NRAS, TP53, WT1, SBDS, CREBBP, KMT2A, SPI1*	*CBFB, PICALM, MLLT10, CHIC2, DNMT3A, RUNX1, NSD1, KMT2A, CREBBP, NRAS, SPI1, TP53, CEBPA, ETV6, LPP, WHSC1L1*
**BLCA**	**-DisGeNet MESH:** D001749 **-EDGAR:** 109800 **-PhenGeID trait:** Urinary Bladder Neoplasms	*HRAS, FGFR3, RB1, KRAS, ATM, CDH1, NQO1, ERCC2, GSTP1, TP53, CDKN2A, TSC1, SLC14A1, CBX6, TACC3, CLPTM1L, CWC27, NAT2, PSCA, CCNE1, APOBEC3A, PSD3*	*ATM, TP53*
**BRCA**	**-DisGeNet MESH:** D001943 **-EDGAR:** 114480 **-PhenGeID trait:** Breast Neoplasms∣Triple Negative Breast Neoplasms	*TSG101, HMMR, ATM, NQO2, AKT1, BRIP1, XRCC3, RB1CC1, PPM1D, RAD54L, FAM175A, NBN, CDH1, CHEK2, BRCA1, BRCA2, BARD1, KRAS, TP53, SLC22A18, ESR1, PHB, PALB2, CASP8, RAD51, PIK3CA, ERBB2, PTEN, CAV1, EP300, FGFR2, NOTCH2, CDKN1B, PARP1, NQO1, AKT2, ESR2, FGF3, FGFR1, FLT1, FN1, GATA3, FOXA1, HRAS, IGF1, AR, MDM2, MMP1, NOS2, NOTCH1, ROR1, FBXW7, PTHLH, RB1, STAT1, TBX3, NCOA3, BAP1, FGF4, TRIM33, MDM4, MLLT10, DNAJC1, ZNF365, ZMIZ1, LSP1, KRT8, USP44, PAX9, PELI2, RAD51B, NTRK3, TOX3, FTO, CDYL2, HNF1B, STXBP4, BABAM1,*	*IGF1, SLC4A7, HMMR, MLLT10, AKT2, BABAM1, GATA3, CDKN1B, NQO2, BARD1, ESR2, AKT1, ATM, TNXB, PAX9, BAG6, CDSN, MDM4, EP300, PRRC2A, RAD54L, TRIM33, PIK3CA, RB1CC1, NBN, RAD51, NOTCH4, RB1, PHB, NCOA3, BAP1, CAV1, PPM1D, STXBP4, ELL, STAT1, TP53, MDM2, BRCA1, CCND1, ESR1, BRCA2, AR, CHEK2, ERBB2, FGFR2, NOTCH1, FAM175A, SEMA3A, FOXA1, ERBB4, ENPP2, HNF1B, BRIP1, HNF4G, PTEN, PTHLH, NTRK3, USP44, GLI2, TERT*

		ELL, ERBB4, TNP1, NRIP1, CYYR1, MKL1, ITPR1, NEK10, SLC4A7, TERT, MAP3K1, MIER3, EBF1, CDSN, PSORS1C1, DDX39B, MCCD1, AIF1, BAG6, EHMT2, C2, TNXB, NOTCH4, HLA-DRA, HLA-DRB1, HLA-DQA1, HLA-DQB1, TAP2, ADGRB3, UST, CCDC170, SEMA3A, ARHGEF5, NOV, SNX32, MYEOV, CCND1, CCDC91, GLI2, MRPS30, GPBP1, HLA-C, PRRC2A, HLA-DRB5, HLA-DOB, TAB2, HNF4G, ENPP2	
**BRNCA**	**-DisGeNet MESH:** D001932<br>**-EDGAR:** PS137800<br>**-PhenGeID trait:** Glioblastoma|Glioma	*ERBB2, BRCA2, TP53, PTEN, IDH2, IDH1, POT1, PPARG, ALK, RTEL1, TERT, SEC61G, ZBTB16, PHLDB1, POLR2A, EGFR, PHLDA1*	*TP53, EGFR, ERBB2, PTEN*
**CERV**	**-DisGeNet MESH:** D002583<br>**-EDGAR:** 603956<br>**-PhenGeID trait:** Uterine Cervical Neoplasms	*FGFR3, HLA-DRB1, HLA-DQA1*	*HLA-DRB1, HLA-DQA1*
**CHLCA**	**-DisGeNet MESH:** D018281<br>**-EDGAR:** NA<br>**-PhenGeID trait:** NA	*IDH1, TP53, KRAS*	*TP53*
**CLL**	**-DisGeNet MESH:** D015451<br>**-EDGAR:** NA<br>**-PhenGeID trait:** Leukemia, Lymphocytic	*TP53, ATM, PLCG2, POT1, ID3, ACTA2, FAS, TSPAN32, C11orf21, BMF, MNS1, RPLP1, PHLPP1, BCL2, ACOXL, BCL2L11, CFLAR, SP110, SP140, FARP2, EOMES, ULK4, IRF4, HLA-DRB1, BAK1, TMPRSS5, BUB1B, ZNF280D, IRF8, CMC1, EXOC2, HLA-DQA1*	*BMF, ATM, ZNF280D, TP53, BCL2*
**CML**	**-DisGeNet MESH:** D054438<br>**-EDGAR:** NA<br>**-PhenGeID trait:** Leukemia, Myeloid, Chronic-Phase	*SETBP1, BCR*	*SETBP1*
**CRCA**	**-DisGeNet MESH:** D015179<br>**-EDGAR:** 114500<br>**-PhenGeID trait:** Colorectal Neoplasms	*MT-CO1, BUB1B, MLH3, BAX, PDGFRL, CTNNB1, AKT1, ODC1, AXIN2, RAD54B, PIK3CA, NRAS, AURKA, DLC1, TRIM28, PLA2G2A, EP300, FGFR3, TP53, CCND1, PTPN12, MCC, KAT5, TLR2, BRAF, TLR4, APC, PTPRJ, MLH1, MSH2, CHEK2, SMAD3, SMAD4, MMP2, TCF7L2, BUB1, KLF5, MSH6, IGFBP3, KDR, KRAS, ABCB1, POLD1, POLE, RET, STK11, DPYD, TYMS, NKX2-3, CYP17A1, FGFR2, MYRF, POLD3, SPSB2, ETV6, KRT8, BRCA2, GREM1, HNF1B, SMAD7, UTP23, SLC25A28, MYEOV, RPS21, MAP3K1, EIF3H, RAD21*	*MLH1, SMAD4, AKT1, TCF7L2, SMAD3, BUB1, TRIM28, EP300, BRAF, BUB1B, PIK3CA, RAD21, MSH6, TYMS, AURKA, APC, STK11, TP53, MSH2, CTNNB1, RET, CHEK2, FGFR2, KDR, FGFR3, NKX2-3*
**DLBCL**	**-DisGeNet MESH:** D016403<br>**-EDGAR:** NA<br>**-PhenGeID trait:** Lymphoma, Large B-Cell, Diffuse	*EZH2, MYD88, PIK3CD, CARD11, CD79B, IRF4, EXOC2*	*CD79B*
**FLYMPH**	**-DisGeNet MESH:** D008224<br>**-EDGAR:** NA<br>**-PhenGeID trait:** Lymphoma, Follicular	*EZH2, BCL2, HLA-DQB1, CXCR5, FLI1, C6orf15, HLA-DRB5*	*EZH2*
**HANC**	**-DisGeNet MESH:** D006258<br>**-EDGAR:** 275355	*PTEN, TNFRSF10B, EGFR, ING1, ING3, ADH1B, ADH7*	*EGFR, PTEN*

	**-PhenGeID trait:** Carcinoma, squamous cell of head and neck∣Head and Neck Neoplasms		
**KDNCA**	**-DisGeNet MESH:** D002292<br>**-EDGAR:** 144700∣605074<br>**-PhenGeID trait:** Carcinoma, Renal Cell	*DIRC2, HNF1B, VHL, HNF1A, RNF139, OGG1, PRCC, MET, EPAS1, PTEN, SETD2, PBRM1, PTGS2, MTOR, TSC1, KDM5C, BAP1*	*SETD2, PBRM1, HNF1A, PTGS2, HNF1B*
**LGCA**	**-DisGeNet MESH:** D008175<br>**-EDGAR:** 211980<br>**-PhenGeID trait:** Adenocarcinoma of lung∣Carcinoma, Non-Small-Cell Lung∣Small Cell Lung Carcinoma	*ERBB2, IRF1, SLC22A18, ERCC6, RASSF1, PIK3CA, EGFR, MAP3K8, CASP8, PARK2, FASLG, BRAF, PPP2R1B, KRAS, CYP2A6, CDKN2A, TP53, BRCA2, PTEN, GSTP1, ERCC1, STK11, VTI1A, FGFR2, KRT8, HNF1B, BPTF, TP63, TERT, BAG6, APOM, MYEOV, MAP3K1*	*GSTP1, BRAF, STK11, TP53, EGFR, ERBB2, FGFR2, PARK2, IRF1, CYP2A6, CDKN2A, PTEN*
**LIVCA**	**-DisGeNet MESH:** D006528<br>**-EDGAR:** 114550<br>**-PhenGeID trait:** Carcinoma, Hepatocellular	*TP53, AXIN1, MTUS1, CDKN3, PIK3CA, CASP8, PDGFRL, CTNNB1, MET, APC, IGF2R, HNF1A, CDKN2A, IGF2, KRAS, HTATIP2, ARID2, FOXM1, GPC3, GNMT, MYC, ABCB1, PTEN, PTGS2, PTK2, HAMP, SKP2, TERT, TGFA, CCNE1, CDK1, KIF1B, HLA-DRB1, HLA-DQB1, HLA-DQA1*	*CASP8, MTUS1, PTK2, FOXM1, CDK1, AXIN1, APC, TP53, CTNNB1, MYC, MET, IGF2R, HNF1A, PTGS2, GPC3*
**OVCA**	**-DisGeNet MESH:** D010051<br>**-EDGAR:** 167000<br>**-PhenGeID trait:** Ovarian Neoplasms∣Ovarian epithelial cancer	*BRCA1, PIK3CA, RRAS2, PARK2, CTNNB1, AKT1, SEPT9, CDH1, OPCML, BRCA2, ERBB2, KRAS, MLH1, MSH2, PTEN, TP53, RAD51C, BRIP1, PMS2, ESR1, CCNE1, RAD51D, BRAF, RSPO1, FGFR2, KRT8, HNF1B, NSF, SKAP1, BABAM1, HOXD3, MYEOV, MAP3K1, IFNL3*	*MLH1, AKT1, BRAF, PMS2, TP53, BRCA1, MSH2, ESR1, CDH1, CTNNB1, BRCA2, ERBB2, FGFR2, BRIP1, PTEN*
**PACA**	**-DisGeNet MESH:** D010190<br>**-EDGAR:** 260350∣613348∣606856∣614320∣613347<br>**-PhenGeID trait:** Pancreatic Carcinoma∣Pancreatic Neoplasms	*RBBP8, TP53, ACVR1B, SMAD4, STK11, KRAS, PALLD, BRCA2, PALB2, BRCA1, CDKN2A, BACH1, TFF2, TERT, CLPTM1L, ABO, NR5A2, PRLHR, TFF1*	*RBBP8, BRCA1, BACH1, BRCA2*
**PRCA**	**-DisGeNet MESH:** D011471<br>**-EDGAR:** 176807∣614731∣601518∣611928∣611868<br>**-PhenGeID trait:** Prostatic Neoplasms	*EPHB2, BRCA2, PTEN, MAD1L1, HIP1, CD82, ZFHX3, KLF6, AR, MXI1, CDH1, FGFR4, MSR1, CHEK2, HOXB13, RNASEL, MSMB, ELAC2, EHBP1, APC, SPOP, CTNNB1, IGF1, NKX3-1, KRAS, CCND1, TGFBR2, KCND3, GOLPH3L, MDM4, FGFR2, MMP7, TUBA1C, KRT8, FERMT2, VPS53, HNF1B, DPF1, PCAT19, KLK15, KLK3, GGCX, TANC1, ITGA6, ADNP, ZGPAT, XAGE3, TEX11, SIDT1, PRKCI, SKIL, AFM, PDLIM5, TERT, RFX6, RGS17, SLC22A1, JAZF1, EBF2, FAM84B, RAD23B, ASCL2, MYEOV, PRPH, TBX5, PPP1R14A, KLK2, LILRA5, VAMP8, BIK, NUDT11, SLC7A3, CLDN11, MAP3K1, SLC22A2*	*TBX5, FERMT2, APC, CDH1, CTNNB1, FGFR2, PTEN*
**SKCM**	**-DisGeNet MESH:** D008545<br>**-EDGAR:** PS155600<br>**-PhenGeID trait:** Melanoma	*CDKN2A, STK11, POT1, PTEN, CDK4, BRAF, MITF, XRCC3, TERT, TYR, HRAS, ERBB4, GNA11, GNAQ, NRAS, MAP2K1, TP53, BAP1, MAP2K2, NF1, RAC1, FTO, CDK10, AFG3L1P, RALY, PIGU, MYH7B,*	*MTAP, RALY, MAP2K2, FTO, MAP2K1, NF1, BRAF, GNAQ, STK11, TP53, GNA11, TYR, PTEN, MITF*

		SLC45A2, IRF4, MTAP, CCND1, EXOC2	
**STCA**	**-DisGeNet MESH:** D013274 **-EDGAR:** 137215∣613659 **-PhenGeID trait:** Stomach Neoplasms	*ERBB2, MUTYH, IRF1, PIK3CA, CASP10, KLF6, APC, FGFR2, KRAS, CDH1, IL1RN, IL1B, MET, ATM, MUC1, ASH1L, PRKAA1, DNAH11, PSCA*	*ERBB2, IRF1, KLF6*
**THCA**	**-DisGeNet MESH:** D013964 **-EDGAR:** PS188550 **-PhenGeID trait:** Thyroid Neoplasms	*NKX2-1, FOXE1, BRAF, HABP2, RET, KRAS, TSHR, TP53, PCNXL2, OBFC1, NRG1, SLK, MBIP*	*NRG1, BRAF, TP53, TSHR*

Table S25. Genes and variant-associated genes included in the stringent and relaxed analyses.

Disorder	Disease-associated genes and variant-genes stringent	Disease-associated genes and variant genes relaxed
**AD**	SORL1, NOS3, ACE, PLAU, MPO, APP, PSEN1, APBB2, PAXIP1, BLMH, PSEN2, HFE, A2M, ADAM10, GSK3B, APOE, MAPT, TREM2, BACE1, IDE, IL1B, INSR, LEP, NPY, BCL2, BDNF, CASP3, IL6R, CR1, MRPL50P4, SPON1, MS4A2, MS4A6A, MIR6503, PICALM, MMP3, BCAS3, CYB561, ABCA7, PVRL2, TOMM40, APOC1, BIN1, ACKR2, CCRL2, LOC102724297, SUCLG2, SNAR-I, RANP6, LOC100289673, HLA-DRB1, CD2AP, GAPDHP15, BZW2, EPHA1-AS1, PTK2B, CLU, MIR6843, HNF4G, SLC16A9, MS4A4A, FNTAP1, MMP12, CYP27C1, OSTN, FBXO8, HLA-DQA1, RBBP4P4, LINC01111	SORL1, NOS3, ACE, PLAU, MPO, APP, PSEN1, APBB2, PAXIP1, BLMH, PSEN2, HFE, A2M, ADAM10, GSK3B, APOE, MAPT, TREM2, BACE1, IDE, IL1B, INSR, LEP, NPY, BCL2, BDNF, CASP3, IGF2, IGF1R, ATP5F1A, INS, BAX, TOMM40, ABCA7, CLU, CR1, EPHA1, CD2AP, BIN1, APOC1, PICALM, VSNL1, INPP5D, NECTIN2, MS4A4A, PCDH11X, CASS4, CYP46A1, CHRNA7, CST3, CYP2D6, DHCR24, DPYSL2, ESR1, NCSTN, HMOX1, IGF1, MIR146A, MAOB, ACHE, MTHFR, PPARG, PRNP, RELN, BCHE, TFAM, TNF, VEGFA, CD33, CRH, SOD2, PLCG2, UNC5C, ABI3, WWOX, TF, CHRNB2, SLC30A6, PGRMC1, EIF2S1, F2, ARC, CALM1, ENO1, HLA-DRB5, IGF2R, TPI1, SNAR-I, IQCK, AMFR, MIR100, MIR296, MIR375, SLC2A4, SLC30A4, MIR708, MIR3622B, MIR4467, TPP1, GAPDHS, PYY, MIR505, MIR766, ADAMTS1, RNA5SP43, IL6R, FRMD4A, MRPL50P4, SPON1, RPL36AP40, LOC101928704, MS4A2, MS4A6A, MIR6503, GAB2, MMP3, RPL10P13, MRPL2P1, PCDH8, FERMT2, SLC24A4, LINC00618, RBFOX1, TMC5, VWA3A, LRRC37A, ARL17B, CACNA1G, BCAS3, CYB561, NFATC1, PVRL2, PPP1R37, PAPOLG, TSN, LINC01116, CST1, SYNJ1, ACKR2, CCRL2, LOC102724297, SUCLG2, GABRA2, UCP1, RANP6, LOC100289673, HLA-DRB1, GAPDHP15, RPL17P25, MTHFD1L, BZW2, NECAP1P1, ZCWPW1, EPHA1-AS1, PTK2B, MIR6843, SMARCE1P4, NKAIN3, HNF4G, RFPL4AP5, GLIS3, RPS29P6, SLC16A9, ANO3, FNTAP1, MMP12, PHLDA1, LINC00615, OLFM4, LINC01550, TRL-TAG3-1, LOC284241, LINC01185, CNTNAP5, CYP27C1, FUCA1P1, CSTP2, OSTN, RAC1P2, TBC1D9, FBXO8, HLA-DQA1, RBBP4P4, FAM46A, NME8, ZNF703, LINC01111, FAM84B
**PD**	GIGYF2, GBA, PODXL, FBXO7, PINK1, HTRA2, CHCHD2, LRRK2, PARK7, ATP13A2, PARK2, PLA2G6, SYNJ1, DNAJC13, DNAJC6, VPS13C, NR4A2, ATXN8OS, UCHL1, VPS35, SNCAIP, TBP, EIF4G1, GLUD2, ADH1C, MAPT, SNCA, ATXN2, ATXN3, FGF20, DDC, DRD2, MAOB, PRKN, SLC18A2, TH, HMGN2P18, RAB25, NUCKS1, RAB29, TIAL1, INPP5F, SPATA19, ASS1P14, SLC2A13, RPL30P13, CNTN1,	GIGYF2, GBA, PODXL, FBXO7, PINK1, HTRA2, CHCHD2, LRRK2, PARK7, ATP13A2, PARK2, PLA2G6, SYNJ1, DNAJC13, DNAJC6, VPS13C, NR4A2, ATXN8OS, UCHL1, VPS35, SNCAIP, TBP, EIF4G1, GLUD2, ADH1C, MAPT, SNCA, ATXN2, ATXN3, FGF20, DDC, DRD2, MAOB, PRKN, SLC18A2, TH, DRD1, IGF1R, GAK, BST1, HLA-DRA, PARK16, DNM1L, PPARGC1A, CP, CYP2D6, GDNF, GFAP, TMEM230, GSTM1,

	CCDC62, GCH1, TMEM229B, RNA5SP397, TPM1, BCKDK, NCOR1P2, CRHR1, MGC57346, CRHR1-IT1, SPPL2C, MAPT-AS1, NSF, WNT3, RIT2, TMEM163, PHF5GP, LZTS3, DCUN1D1, MCCC1, GAK, TMEM175, DGKQ, BST1, FAM47E, FAM47E-STBD1, GPRIN3, LHFPL2, BTNL2, HLA-DRB1, HLA-DQB1, FAM126A, GPNMB, KRTCAP2, SLC41A1, RAD1P1, MIR4697HG, SYT10, UBBP4, ACMSD, CERS6, DDRGK1, LAMP3, MMRN1, HLA-DRA, HLA-DQA1, MTCO3P1, KLHL7-AS1	HFE, HMOX1, HSPA9, IL6, MAOA, MTHFR, NOS1, ABCB1, BDNF, SLC6A3, SOD1, SOD2, TNF, GSTP1, DDIT4, CYP2E1, MAP3K5, NGF, NQO1, AIF1, GSTA4, IGF2, TRPM2, HLA-DRB5, IGF2R, INS, ENO2, FBP1, FCER2, GPX1, HGF, HSPA1A, INSR, MIR181C, MAG, MTA1, BAG5, TCL1B, ADARB2, COL19A1, SLC2A14, EDN1, FGB, CNTNAP2, HBG1, DRAXIN, KCNJ4, MAP2, CEACAM6, NCAPG2, SLC30A10, NECTIN2, RPL6, RPL23A, RPS8, TALDO1, RPL14, PPIAP7, HMGN2P18, RAB25, NUCKS1, RAB29, SIPA1L2, TIAL1, INPP5F, OR5AZ1P, SPATA19, ASS1P14, SLC2A13, RPL30P13, CNTN1, PRICKLE1, CCDC62, GCH1, TMEM229B, RNA5SP397, TPM1, BCKDK, CDH8, NCOR1P2, CRHR1, MGC57346, CRHR1-IT1, SPPL2C, MAPT-AS1, KANSL1, NSF, WNT3, RIT2, CTIF, TMEM163, PHF5GP, LZTS3, DCUN1D1, MCCC1, TMEM175, DGKQ, FAM47E, FAM47E-STBD1, GPRIN3, LHFPL2, LINC01012, BTNL2, HLA-DRB1, HLA-DQB1, FAM126A, GPNMB, LOC101928208, SH3GL2, RPSAP19, KRTCAP2, SLC41A1, RAD1P1, OR5BD1P, MIR4697HG, SYT10, RPS27P21, UBBP4, ACMSD, CERS6, DDRGK1, LAMP3, MMRN1, HLA-DQA1, MTCO3P1, KLHL7-AS1
**ALL**	TAL1, TCF3, TAL2, BAX, PAX5, NBN, NUP214, FLT3, BCR, IKZF1, CDKN2A, ABL1, CRLF2, GATA3, ARID5B, LHPP, CEBPE, OR5AL2P, C14orf119, OR5AL1	TAL1, TCF3, TAL2, BAX, PAX5, NBN, NUP214, FLT3, BCR, IKZF1, CDKN2A, ABL1, CRLF2, SH2B3, ARID5B, LIG4, CEBPE, NQO1, ETV6, KMT2A, MTHFR, ABCB1, TP53, XRCC1, RUNX1, ERG, GNB1, HLF, JAK2, SLC19A1, MTRR, NAT2, NOTCH1, CYP2E1, NT5C2, FPGS, CREBBP, P2RY8, CASP8, DUX4, JAK1, EPHX1, EP300, IDH1, JAK3, PAG1, FBXW7, RB1, NSD2, IL7R, CDK6, IKZF3, HOXD4, CYP1B1, IKZF2, ARNT, NOTCH2, VPREB1, CYP1A2, CYP2C8, HCK, PRDM14, SETD2, BLM, GATA3, LHPP, ELK3, CDC20, ELOVL1, OR5AL2P, C14orf119, PYGL, ADAMTS18, TP63, LOC100996325, CSGALNACT1, OR5AL1, FIGNL1, INTS10
**AML**	TGM6, SETBP1, SH3GL1, FLT3, CHIC2, CEBPA, NPM1, WHSC1L1, CBFB, JAK2, NUP214, TERT, MLF1, MLLT10, LPP, GATA2, KRAS, ETV6, DDX41, KIT, RUNX1, NSD1, PICALM, DNMT3A, PTPN11, IDH1, IDH2, NRAS, TP53, WT1, SBDS, CREBBP, KMT2A, SPI1	TGM6, SETBP1, SH3GL1, FLT3, CHIC2, CEBPA, NPM1, WHSC1L1, CBFB, JAK2, NUP214, TERT, MLF1, MLLT10, LPP, GATA2, KRAS, ETV6, DDX41, KIT, RUNX1, NSD1, PICALM, DNMT3A, PTPN11, IDH1, IDH2, NRAS, TP53, WT1, SBDS, CREBBP, KMT2A, SPI1, FANCA, FANCC, FANCD2, FANCF, FANCG, CSF3R, MECOM, FANCE, SF3B1, GFI1, HRAS, MYH11, TET2, PALB2, BRIP1, CBL, INPP4B, U2AF1, CTNNA1, SEPTIN9, JAK1, NTRK3, CHMP5, PTPRT, CSF1R, CSF2, CSF3, ERG, FANCB, H1-2, HOXA9, MYC, NUP98, RAD51, BCL2, STAT3, SVIL, TSC2, BAALC, RUNX1T1, RUNX3, CD33, CD44, TNFSF10, FAS, IRF1, NF1, KMT2C, CDK6, PIM2, BCOR, PSIP1, EP300, DAPK1, FOXO1, SETD2, MN1, PVR, EIF4EBP1, EPHX1, ARHGAP26, MET, S100A8, RASGRP1, CUX1, HGF, HSPB1, MALAT1, NOTCH2, ASXL2, SPARC, BRCA1, CD9, ADCY7, CNR2, DHX15, ERCC4, ACSL6, ANXA2, LYL1, ZBTB7A, POU4F1, NECTIN2, DLEU2, CCND2, CEBPD, CST3, FHL2, ANXA5, EHD3, ID2, FANCL, RGS2, S100A10, SPRY4, CBLB, AQP9, SH2B3,

		IFI30, MAD2L2, GAS2L1, EHMT2, VSIG4, CTSH, CTSZ, GPR183, LPAR1, ENO2, CBLC, UBE2T, GTF2I, H1-0, ANXA4, ANXA6, GCOM2, AGRN, MX1, ATP1B1, PDE4B, FXYD6, FANCI, ENAH, FANCM, RAD21, RAD51C, BACH2, BLM, SGK1, TCEA2, TRH, TRIO, TUBB2A, XRCC2, PXDN, CTC1, VOPP1, CAPG, CAPN2, CASP7, SLX4, ASMTL, SYNGR1, RPL21P43
**BLCA**	HRAS, FGFR3, RB1, KRAS, ATM, CDH1, NQO1, ERCC2, GSTP1, TP53, CDKN2A, TSC1, RNA5SP357, SLC14A1, C19orf12, FAT1P1, CBX6, MIR944, TACC3, CLPTM1L, CWC27, NAT2, CASC8, PSCA, RNA5SP358, CCNE1, RPS11P1, APOBEC3A, P3H2, PSD3, CASC11	HRAS, FGFR3, RB1, KRAS, ATM, CDH1, NQO1, ERCC2, GSTP1, TP53, CDKN2A, TSC1, STAG2, KDM6A, PSCA, TACC3, NAT2, CDKN1A, RASSF1, EGFR, MTOR, GPX1, GSTM1, IGF1, CXCL8, MIR145, MMP2, MMP9, MTHFR, MYC, PTGS2, SOD2, TERT, TNF, CLPTM1L, NAT1, IL2, ESR1, FAS, ESR2, MIR100, NOTCH1, SOX2, KLF5, CCNE1, GLI1, MIR29C, MIR99A, ERBB3, TIMP2, TYMP, ERCC4, NOTCH2, HMGN5, KMT2D, GSTZ1, HMGB3, MDM4, POLB, AS3MT, UGT2B7, LOXL4, CSF3, IFNA2, IGFBP3, ARID1A, FERMT2, GSTO2, CREBBP, CYP4B1, IGFBP5, MPO, MT3, PRSS3, SLC19A1, MIR532, HDAC4, MIR4324, EP300, FANCA, BPTF, HOXA9, BIRC3, IFNB1, LIG1, LOXL1, MIR34B, KMT2A, ACHE, MT2A, POR, PPP3CC, BRCA2, TFRC, TRPV1, USP7, BAP1, EOMES, INPP4B, APOBEC3B, SLC12A7, NRSN1, VWA3A, NCAN, ENO2, FANCC, MAPK15, CALHM1, FBXW8, AMFR, PABPC1, SMC1B, ANPEP, OR8S1, AQP3, ISL1, YJEFN3, KRT16, RHOA, ANXA2R, LAMA2, MAGEA9, MT1A, ASAP1, ATP5F1D, ANKFY1, ASXL2, PSMB2, NECTIN2, KMT2C, SRC, MIR33B, PRDM2, TBL1XR1, SMC1A, CHD6, IGSF21, GGH, NCOR1, RALGPS1, ESPL1, RNA5SP357, CLK3, SLC14A1, C19orf12, FAT1P1, CBX6, MYNN, MIR944, CWC27, CASC8, RNA5SP358, RPS11P1, APOBEC3A, P3H2, PSD3, CASC11
**BRCA**	TSG101, HMMR, ATM, NQO2, AKT1, BRIP1, XRCC3, RB1CC1, PPM1D, RAD54L, FAM175A, NBN, CDH1, CHEK2, BRCA1, BRCA2, BARD1, BCPR, KRAS, TP53, SLC22A18, ESR1, PHB, PALB2, CASP8, RAD51, PIK3CA, ERBB2, PTEN, CAV1, EP300, FGFR2, NOTCH2, CDKN1B, PARP1, NQO1, AKT2, ESR2, FGF3, FGFR1, FLT1, FN1, GATA3, FOXA1, HRAS, IGF1, AR, MDM2, MMP1, NOS2, NOTCH1, ROR1, FBXW7, PTHLH, RB1, STAT1, TBX3, NCOA3, BAP1, FGF4, TRIM33, LOC101928890, EMBP1, MDM4, MLLT10, DNAJC1, ZNF365, ZMIZ1, MAPKAPK5P1, LSP1, PRR33, OVOL1-AS1, MIR3164, LINC01488, IFITM9P, KRT8, USP44, RPL32P28, PAX9, PELI2, RAD51B, THSD4, NTRK3, TOX3, CASC16, FTO, CDYL2, HNF1B, STXBP4, BABAM1, ANKLE1, ELL, LINC01122, LINC01101, ERBB4, TNP1, DIRC3, NRIP1, CYYR1, CYYR1-AS1, MKL1, ITPR1, NEK10, SLC4A7, ADAM29, TERT, FGF10-AS1, RPL26P19, MAP3K1, MIER3, EBF1, CDSN, PSORS1C1, PSORS1C3, HCG27, USP8P1, DHFRP2, FGFR3P1, HLA-S, HCG26, MICB, DDX39B, MCCD1, ATP6V1G2-DDX39B, AIF1, BAG6, EHMT2, C2, TNXB, NOTCH4, C6orf10, BTNL2, HLA-DRA, HLA-DRB9, HLA-DRB1, HLA-DQA1, HLA-DQB1, MTCO3P1, HLA-	TSG101, HMMR, ATM, NQO2, AKT1, BRIP1, XRCC3, RB1CC1, PPM1D, RAD54L, FAM175A, NBN, CDH1, CHEK2, BRCA1, BRCA2, BARD1, BCPR, KRAS, TP53, SLC22A18, ESR1, PHB, PALB2, CASP8, RAD51, PIK3CA, ERBB2, PTEN, CAV1, EP300, FGFR2, NOTCH2, CDKN1B, PARP1, NQO1, AKT2, ESR2, FGF3, FGFR1, FLT1, FN1, GATA3, FOXA1, HRAS, IGF1, AR, MDM2, MMP1, NOS2, NOTCH1, ROR1, FBXW7, PTHLH, RB1, STAT1, TBX3, NCOA3, BAP1, FGF4, GPNMB, IFNB1, ZNF366, PDPK1, PLA2G4A, NCOR1, ADAR, MYH9, SREBF2, ICAM5, MAP3K1, APC2, GRIK2, NRCAM, STARD8, CDKN2A, HOXB13, COL7A1, NOP9, ERBB4, ZNF365, TOX3, MSH6, HADHB, LSP1, MDM4, ABCC1, ARID1B, RAD51B, FTO, ZNF432, CTNNB1, PHGDH, KRT8, RAD51C, CCND1, NCOA1, ERBB3, MTOR, TERT, RNF115, H19, LGR6, PRC1, KCNH1, NRIP1, STXBP4, FGF10, RELA, EXO1, MED12, OLA1, ZMIZ1, DAP3, ATG10, NR2F6, FLACC1, PTPRD, ZC3H11A, NAT2, ABCB6, ADAM10, NDRG1, YAP1, AGR2, SORBS1, HPSE, IL24, UBE2C, CHEK1, NISCH, COMT, CLDN4, CSF1, CSF1R, CSF2, CSF3, CST6, CYP1A1, CYP1B1, CYP2D6, CYP3A4, CYP17A1, CYP19A1, CYP24A1, DNMT1, DNMT3A, DNMT3B, JAG1,

	DQA2, HLA-DQB2, TAP2, ADGRB3, UST, CCDC170, SEMA3A, ARHGEF5, RPL17P33, HIGD1AP6, NOV, MIR1208, CHCHD4P2, ADIPOR1P1, PHB2P1, SNX32, MYEOV, CCND1, CCDC91, PGAM1P5, UBA52P7, LINC00395, RNA5SP94, GLI2, CYCSP42, MIR4457, MRPS30, GPBP1, HLA-C, RPL3P2, ZDHHC20P2, MICA, PPIAP9, PRRC2A, HLA-DRB5, HLA-DOB, TAB2, LINC00589, HNF4G, ENPP2, LINC00824, RPL36P14	E2F1, EDNRA, EGF, EGFR, AHR, EPHB4, ESRRA, ETV4, EZH2, F3, FASN, DKK1, FOXM1, SIRT1, FOS, ABL1, SLC39A6, GJA1, PDCD4, GLI1, GPER1, GPX1, GRB7, SETD2, GSN, GSTP1, H2AX, NRG1, HIC1, HIF1A, HMOX1, HRG, HSP90AA1, IFNG, IGF1R, IGFBP5, IGFBP7, APRT, IL1B, IL6, CXCL8, IL10, IDO1, JUN, AREG, MALAT1, KDR, KRT5, STMN1, LEP, LEPR, BCAR4, LOXL2, MIR10B, MIR126, MIR141, MIR145, MIR146A, MIR200B, MIR200C, MIR205, MIR206, MIR214, MIR221, MIR222, MIR29A, MKI67, MMP2, MMP3, MMP9, MMP14, MRE11, MST1, MTHFR, MTR, NHS, NOS3, NOTCH3, NOTCH4, YBX1, PAEP, SERPINB2, PAK1, FOXP3, WWOX, PGR, ABCB1, SERPINB5, PIK3CB, PIN1, PRKAA1, PRKAA2, MAP2K7, KLK10, BAG1, PTGS2, BAX, RAF1, RARA, RARB, BCL2, RECQL, CXCL12, SFRP1, BMP2, SLC2A1, BMP4, SLC5A5, SNAI2, SNAI1, SNCG, SOD2, SPP1, SRC, BRAF, STAT3, STAT5A, AURKA, SULT1A1, ZEB1, TFAP2A, TFRC, TGM2, THBS1, TNF, TOP2A, TP53BP1, TP73, TYMS, VDR, VEGFC, VIM, WT1, XBP1, XRCC2, CXCR4, ADAM12, ARID1A, BCAR3, CAT, TNFSF10, INPP4B, CCNE1, CLDN1, MTDH, ABCG2, BCAR1, APOBEC3B, KEAP1, ZEB2, ETS2, SETBP1, GPI, KIT, KRT14, TXN, L3MBTL3, ALK, BIRC5, TUBB3, PPARGC1B, ADRA1A, CUX1, DHFR, KCNIP3, MIR342, NF1, WNK1, SLC2A5, TEK, PER2, EPOR, SLCO1B1, DDX3X, APOBEC3A, FLNA, CADM1, HES1, IKBKB, MIR152, MIF, LEF1, MIR429, EMSY, RPS6KA3, SFRP2, ANKRD30A, CDH5, FST, LAMTOR5, ATF2, FABP4, MIR127, PIM1, PLXNB1, PER3, RASAL2, CD40, CPT1A, ENO1, KRT18, MFGE8, SLC16A3, CD74, NET1, NCOA2, CFL1, SGK3, MIR132, MIR301A, MME, NF2, PER1, DLL4, KDM3A, PTGS1, MIR489, RBM3, MAP2K4, CXCR5, C1QBP, TOP1, TIMELESS, DLEC1, DDIT3, DPYD, HP, ADAMTS1, OCLN, FHL2, JMJD6, FOLR2, GPX4, HOXB9, MIR10A, NEDD4, ATP2A3, PFKFB4, SULF2, BCL2A1, RPS6KB2, CCL20, RAPH1, NR2F1, WNT10B, TFPI2, ABRAXAS1, STC2, LPAR1, DEK, CDKN2C, TACC2, CSNK1D, ECT2, EIF4A2, ELK3, FLNB, GAB1, PRDX5, GPX2, GZMB, LDHB, LHCGR, ACHE, MSI1, MYOD1, PLD2, PLS3, PBRM1, ARRDC3, PTPRC, BCHE, RPS6, FKBPL, CLSPN, SHMT1, NORAD, KMT2D, PDLIM7, FOXQ1, THEMIS2, H6PD, CLCA2, CXCL9, ANGPTL4, ARTN, CTCF, EPB41L3, TGFBR2, AKAP9, TENM1, TNIP1, ARFGEF2, CENPF, SLCO2B1, CNR2, SRARP, TMPRSS6, DSC3, EFNA1, ERCC6, FBL, MECOM, FABP7, TRIM29, ABCA4, GABRP, GPC1, HDLBP, HHEX, APCS, BIRC2, MAL, MAOA, NFKBIA, OCA2, HDAC7, SERPINE2, BCL11A, RARG, ROBO1, RRAD, CLIP1, SFRP5, BMPR2, SLC35A2, XDH, NAA25, MED28, ADAM33, CUL5, CBFB, ACVR1, AKAP12, HDAC4, CDA, CDC27, LRRC3B, EDNRB, DLL1, HSPA1B, TP53BP2, IBSP, CDH2, PDCD6, SEMA3A, FOXP4, FGD5, DDX10, DES, AFP, ENPEP, EREG, FAAH, EFEMP1, PPM1E, DIP2C,

	NCOA6, CIC, PLCB1, ABCB10, ZFYVE26, FUS, CHD5, FBXO8, AMFR, UTS2R, HSD11B1, IRF8, ZNF404, IFNA2, GEN1, ARF1, ARHGDIA, LLGL2, MIR101-2, MIR24-2, MAGEA4, MEIS1, MMP10, NUP98, PCDH8, KLK15, GSDMB, RGS2, RPS4X, BGN, SRSF1, ARAP3, MICAL1, SLC2A2, BTK, TCP1, NSD2, EHMT1, NUP214, MFAP5, LIMD2, H4C1, CASP7, ACCS, EIF2S2, MTMR3, CTU1, MAP3K13, REPS2, TCL1B, SYNE1, CRHR1, PDGFA, TRERF1, AKAP8, UBD, LBX1, CFL2, MAGED2, DEPP1, BTN3A2, HELQ, COL11A1, AHSA2P, SPATA18, ADHFE1, TICAM1, ZNF569, B3GALNT2, TEKT4, DIO3, DMD, DVL3, SUSD3, ERCC3, GPC2, DOP1A, MCF2L2, SIK3, COTL1, HEY2, MACF1, KAT6B, NIPBL, APPL1, HEYL, NSMCE2, GRIK3, CXCL3, HLCS, HNRNPK, HNRNPL, HOXA4, ACADM, AQP8, SLC10A6, JAG2, KCNA5, CEP85L, LIPE, MAGEA1, ASL, MT3, NDUFS3, NMBR, TAS2R13, MAGEC2, MRPS23, PDZK1, CFP, TREM1, POU2F1, PPA1, RMND1, ELP3, RPRD1A, P3H2, MIR31HG, DHX32, PRODH, GKN1, PRPS1, GALNT16, EPG5, PTPN14, RAC2, RIC8A, RPS9, RXRB, BCORL1, SCP2, MNX1-AS1, BMP1, SIM1, SLAMF1, SLC6A3, TBXAS1, UMPS, VEGFB, VWF, WARS1, SMC6, MANEA, SUV39H2, ZNF668, ZNF436, PRRT1, SLC2A10, CLPTM1L, H2BC4, RSPO3, SYNJ2, PRPF4B, HERC2, CCNH, RCCD1, SLC6A5, SLC28A1, TP53I3, SPAG6, ARHGAP11A, SRGAP3, G3BP2, PABPC1, NUDT2, ATP7B, EEF2, CXCL2, EVL, MRPL19, TANK, ZNF667-AS1, SH2D3A, CDH10, ARFRP1, FARP1, HNRNPR, PKDREJ, CDS1, EIF3M, SPTLC1, FAM189B, POP1, TMED1, B4GAT1, CLASRP, ZNF277, WBP4, ABCB8, GALNT5, GPR45, ADAT3, FAM83F, CHRND, SLC22A9, SORCS1, OSBPL11, FCRL3, AGAP2, SCGB3A2, CLCN1, CLIC1, DNAJC24, EFCAB13, FAM210A, CNGA2, NLRP8, WDR88, KLHDC7A, C1orf87, TMEM125, COL19A1, FAM131A, METTL6, RTP1, DNAJC21, UBLCP1, CD109, MPLKIP, RALYL, OR1N1, CRX, CRYAA, ABHD12B, UNC45B, CST4, ANKRD29, DTX3L, TAFA4, VPS13B, MAMDC4, USP54, DGKG, GPR180, DBN1, DAW1, XIRP1, GIMAP1, ADAMTS19, DNAH9, DNASE1L3, DPAGT1, EEF1B2, DTX3, EIF5, CELA1, NUDT17, ABCA3, ZNF25, ZNF438, SLC39A12, NSUN6, ADGRF4, KIF6, RNF182, PXDC1, FKTN, BEND7, ALDOA, NID2, FRMPD1, TRAPPC8, CCT5, KLHDC10, SPEN, TBC1D9B, COLGALT2, GGA3, ZCCHC14, RGL1, SYNE2, ASTN2, EHBP1, VPS39, UBR4, HEY1, ZNF281, STX12, ZNF318, HEPACAM2, G6PC, SNX32, GABRA4, MAP7D2, HEATR9, CATSPERE, ALS2CL, BOD1L1, SIPA1L1, GGA1, TOR1AIP1, RIBC2, IL1RAPL2, ARFGAP3, CNNM4, DAZAP1, GDF10, PGBD3, TRMT2A, CLUL1, CNTN6, GOLIM4, UTP20, GNAI2, GOLGB1, JAKMIP3, NPBWR1, CDH20, OTOGL, EXOC3L1, CAVIN1, ANKRD34A, RNF149, ANK1, ANK2, MRPS28, MRPL13, GRIN2D, GRM6, GTF2A1, GUCY1A2, GUCY2F, PURG, H1-2, PIK3R4, HK3, H2AC21,

	HOXA3, HOXD11, KRTAP20-1, KRTAP21-1, NLRP9, NLRP14, ZNF546, TRIML1, TREML1, SP110, KCNT2, APOC4, IGBP1, CTU2, WDR53, SHROOM2, ITGA9, ARAF, EIF6, SPATA21, MROH7, C5orf34, KCNJ1, KCNJ15, KPNA5, KRTAP10-8, LINC00671, C4orf50, GFRAL, KTN1, LAMA2, LGALS2, LLGL1, PLEKHD1, PRICKLE3, HAPLN4, YY2, MAN2C1, MIA2, AFF1, MIR345, MYH1, PPP1R12B, NDUFA2, NDUFA3, NDUFA8, LRRC37A2, NFYC, ACO2, SMARCAL1, ITSN2, ARHGEF4, CUTC, MRPS7, TRAPPC12, CYB5R4, HSPA14, NME8, KRT76, HOOK1, PDE2A, NIP7, ANAPC5, PHF7, PPHLN1, CPSF3, PADI3, INPP5K, SIX4, ATP8B1, PHKB, ATP6V0B, PLOD1, PNLIPRP1, POLH, POLR2F, SEMA5B, NLE1, SMOX, GNB1L, UGT1A9, ARHGEF38, TMEM161A, OXSM, PPP1R3A, MAGOHB, ZDHHC4, RIF1, ZNF532, SBNO1, UQCC1, PACC1, TMEM39A, STRBP, AVPR2, MTMR8, KIF16B, N4BP2, ZFP64, NUP133, PCDHGB6, PCDHB15, MOSPD1, PRRG1, PANX2, FEM1C, CMC2, MRPS22, SLC17A6, MCOLN1, KIAA1324, LRRC7, PDZD4, ZSWIM5, MAGEE1, WFDC1, NECTIN2, OSTC, ACTA2, RANBP1, RAP1GAP, RASGRF2, RFC4, RFX2, RHD, TRMT11, RPL23A, RPL31, RPLP2, RPS7, RPS8, SCNN1B, ANKEF1, FAM217B, KANSL1-AS1, C16orf58, FNDC3B, PCDH20, SKIV2L, MRPL9, SLC8A3, SLC9A2, SMARCD1, SORL1, SPAST, SPTAN1, STAT4, STX5, SURF1, VPS72, TECTA, TESK1, THBS3, TLE3, TLL1, TSPAN4, C1QB, TNS1, TPM4, TPTE, TRPC4, TTC3, UPK1B, ZNHIT2, ZNF22, ZNF75A, FASTKD3, PRRC2A, HSD17B8, DYNC2H1, TXNDC15, LONRF3, ZBTB3, RUFY1, CUBN, WDCP, PUS1, FXR1, CFHR5, TSC22D4, OR12D3, DYSF, EOMES, FCRL5, TCF7L1, DDX59, COG3, INHBE, TFAP2D, OBSCN, FSCB, ZNF644, NLRC5, ZNF541, SYT3, NOA1, HOOK3, LZTS2, KBTBD8, PLEKHA8, MFSD9, TMEM25, TMTC4, ELP1, H2BC12, DGKE, DDO, MICALL1, APOL1, THOC5, SSNA1, DDX18, TAX1BP1, AP1M1, KALRN, MACIR, STPG1, SLC7A7, MAP3K6, SPOCD1, SCARF2, INA, LMF2, RRP9, CD2, RASL10B, BOC, ALKBH8, SLC25A51, LRRFIP1, ATP6AP1L, LDHAL6B, MED14, TRMT10A, OTOF, PIGS, ARHGAP29, AKAP6, ACY1, PPM1F, ZNF646, ACAP1, CTIF, LRBA, LRRC37A, RCE1, MIR6808, PEX14, TRIM33, LOC101928890, EMBP1, ZBED6, MLLT10, DNAJC1, MAPKAPK5P1, TCF7L2, PRR33, OVOL1-AS1, MIR3164, LINC01488, IFITM9P, PKNOX2, RPS27P20, USP44, FRY, RPL32P28, RNY1P8, PAX9, PELI2, CCDC88C, THSD4, NTRK3, CASC16, CDYL2, HNF1B, PCAT18, BABAM1, ANKLE1, ELL, KCNN4, WDR43, LINC01122, RALB, LINC01101, TNP1, DIRC3, RPS2P1, CYYR1, CYYR1-AS1, EMID1, MKL1, ITPR1, NEK10, SLC4A7, ADAM29, FGF10-AS1, RPL26P19, MIER3, ARRDC3-AS1, EBF1, RANBP9, DHX16, CDSN, PSORS1C1, POU5F1, PSORS1C3, HCG27, USP8P1, WASF5P, DHFRP2, FGFR3P1, HLA-S, HCG26, MICB, DDX39B, MCCD1, ATP6V1G2-DDX39B, AIF1,

		BAG6, EHMT2, C2, CFB, NELFE, TNXB, C6orf10, HNRNPA1P2, BTNL2, LOC101929163, HLA-DRA, HLA-DRB9, HLA-DRB1, HLA-DQA1, HLA-DQB1, MTCO3P1, HLA-DQA2, HLA-DQB2, TAP2, HLA-DMB, ADGRB3, RPL17P25, UST, CCDC170, ARHGEF5, RPL17P33, HIGD1AP6, NOV, MIR1208, CHCHD4P2, MXRA8, ADIPOR1P1, PHB2P1, MYEOV, TMEM45B, CCDC91, PGAM1P5, UBA52P7, LINC00395, MARK2P12, AQP4, RNA5SP94, INHBB, GLI2, XPOTP1, CYCSP42, MIR4457, MRPS30, SETD9, GPBP1, RAB5CP2, FOXF2, MCUR1, HLA-C, RPL3P2, HLA-B, ZDHHC20P2, MICA, PPIAP9, HCG23, HLA-DRB5, HLA-DOB, FAM46A, TAB2, LINC00589, HNF4G, ENPP2, LINC00824, RPL36P14
**BRNCA**	ERBB2, BRCA2, TP53, PTEN, IDH2, IDH1, POT1, PPARG, ALK, RTEL1, RTEL1-TNFRSF6B, TERT, SEC61G, CDKN2B-AS1, ZBTB16, LOC101928875, PHLDB1, RPL10P13, POLR2A, LINC00977, MIR3686, EGFR, PHLDA1, GSDMC	ERBB2, BRCA2, TP53, PTEN, IDH2, IDH1, POT1, PPARG, ALK, EGFR, BRAF, CTNNB1, CDKN2A, MGMT, VEGFA, CDK4, CCND1, SOX2, IL2, SPP1, YAP1, HRAS, PON1, CYP2C9, SIRT1, PCNA, PTCH1, RELA, S100B, SOD2, TGM2, EML4, IQGAP2, DTX2, AMOT, DAPK1, FJX1, DLL1, ITGB2, HES5, NRARP, SMO, TGFBR2, IQGAP1, POLR3B, RTEL1, RTEL1-TNFRSF6B, TERT, SEC61G, CDKN2B-AS1, ZBTB16, LOC101928875, PHLDB1, RPL10P13, ETFA, POLR2A, LRRC31, LINC00977, MIR3686, CDKN2B, PHLDA1, GSDMC
**CERV**	FGFR3, MICA, HLA-DRB1, HLA-X, HLA-DQA1	FGFR3, ZPBP2, EXOC1, RNA5SP173, MICA, HLA-DRB1, ZNF70P1, GSDMB, LINC00290, HLA-X, HLA-DQA1, HCG25
**CHLCA**	IDH1, TP53, KRAS	IDH1, TP53, KRAS, IDH2, RNF43, EGFR, ERBB2, FGFR2, IL6, PTGS2, BAP1, BRAF, SMAD4, PBRM1, ARID1A, PTEN, GNAS, MSLN, PRKACB, NOS2, PTPN3, SLC5A5, PEG3, PRKACA, KMT2C, ROBO2
**CLL**	TP53, ATM, PLCG2, POT1, IGHV3-21, ID3, ACTA2, FAS, FAS-AS1, TSPAN32, C11orf21, MIR4301, GRAMD1B, BMF, MNS1, RPLP1, RPL10AP12, PHLPP1, BCL2, ACOXL, LOC400997, BCL2L11, CFLAR, CFLAR-AS1, SP110, SP140, FARP2, EOMES, ULK4, SDHDP3, IRF4, HLA-DRB1, BAK1, SRRM1P1, MDS2, TMPRSS5, BUB1B, ZNF280D, GEMIN8P1, IRF8, CMC1, TERC, EXOC2, HLA-DQA1, CCAT1	TP53, ATM, PLCG2, POT1, IGHV3-21, P2RX7, SF3B1, IRF4, LEF1, BCL2, BRAF, XPO1, SP140, BMF, PRKD2, ACOXL, FARP2, QPCT, C11orf21, BIRC3, IL6, ITGA4, MYC, PIK3CA, CCND1, BCL6, SYK, BTK, CD5, RPS15, PMAIP1, IKZF3, MTHFR, TFRC, TNFRSF11A, ARL11, PTGS2, TNFSF11, MIR143, U2AF1, CDKN2B-AS1, TOPBP1, LILRA4, IL19, IGHG1, MIR145, NFKBIE, POLB, PPP2R5C, RBL2, SRSF2, CPEB1, VDR, ZRSR2, KLRC4, ID3, RHOU, ACTA2, FAS, FAS-AS1, ASCL2, TSPAN32, MIR4301, GRAMD1B, MNS1, RPLP1, RPL10AP12, IRF8, PHLPP1, DTNB, LOC400997, BCL2L11, CFLAR, CFLAR-AS1, CASP8, SP110, ODF3B, EOMES, ULK4, NCK1, SDHDP3, MYNN, BANK1, CAMK2D, TERT, HLA-DRB1, HLA-DQA1, BAK1, OPRM1, IPCEF1, GPR37, SRRM1P1, UBA52P6, MDS2, TMPRSS5, BUB1B, ZNF280D, GEMIN8P1, CMC1, TERC, ARSJ, EXOC2, HLA-DQB1, CCAT1, DMRTA1
**CML**	SETBP1, BCR, TUFMP1, RPL34P31	SETBP1, BCR, TUFMP1, RPL34P31
**CRCA**	MT-CO1, BUB1B, MLH3, BAX, PDGFRL, CTNNB1, AKT1, ODC1, AXIN2, RAD54B, PIK3CA, NRAS, DCC, AURKA, DLC1, FLCN, TRIM28, PLA2G2A, EP300, FGFR3, TP53, CCND1, PTPN12, MCC, KAT5, TLR2, BRAF, TLR4, APC, PTPRJ, MLH1, MSH2, CHEK2, SMAD3, SMAD4, MMP2, TCF7L2, BUB1, KLF5,	MT-CO1, BUB1B, MLH3, BAX, PDGFRL, CTNNB1, AKT1, ODC1, AXIN2, RAD54B, PIK3CA, NRAS, DCC, AURKA, DLC1, FLCN, TRIM28, PLA2G2A, EP300, FGFR3, TP53, CCND1, PTPN12, MCC, KAT5, TLR2, BRAF, TLR4, APC, PTPRJ, MLH1, MSH2, CHEK2, SMAD3, SMAD4, MMP2, TCF7L2, BUB1, KLF5,

	MSH6, IGFBP3, KDR, KRAS, ABCB1, POLD1, POLE, RET, STK11, DPYD, TYMS, RNA5SP299, NKX2-3, CYP17A1, FGFR2, MYRF, MIR3164, POLD3, SPSB2, ETV6, KRT8, BRCA2, GREM1, LOC100131315, HNF1B, SMAD7, MIR4758, CABLES2, RPL26P19, C5orf66, LINC00536, UTP23, CCAT2, LINC00709, SLC25A28, MYEOV, RPS21, MAP3K1, EIF3H, RAD21	MSH6, IGFBP3, KDR, KRAS, ABCB1, POLD1, POLE, RET, STK11, DPYD, TYMS, NAMPT, SLCO1B3, EYA4, SYNE1, ABCA1, EVL, LGR6, TCERG1L, ABCB5, IL6ST, DACT1, PRKD1, CD109, HAPLN1, RNF182, GREM1, GUCY1A2, SMAD7, COX1, COX2, MUTYH, PMS2, SH3TC1, PPARG, PRKCE, PTPRD, BMP4, TGFB1, CDH1, EGFR, ARID1A, GNAS, CCND2, RHPN2, FEN1, POLD3, VTI1A, LPAR1, LRP1, TET2, HHIP, SLCO2A1, TMEM238L, LAMC1, CUBN, BOC, YAP1, CHD1, WIF1, PARP1, CYP1A2, CYP1B1, NQO1, TYMP, ERCC2, ESR2, ALDH2, SATB2, FN1, GSTM1, GUCY2C, IGF2, CXCL8, FADS1, SMAD2, SMAD9, MMP1, ABCC1, CYTB, MTHFR, ND4L, MYC, NME1, LEF1, KRT20, RNF43, FBXW7, ZMIZ1, PTEN, PTGS2, BCL2, SFRP1, SFRP2, NXN, SPARC, SRC, TGFBR1, TGFBR2, TNF, XRCC1, TFPI2, CALR, CASP8, LGR5, TNFSF10, PROM1, DCLK1, MTDH, FADS2, ABCG2, ADH1B, BIRC5, MTRR, NTHL1, WNK1, TGM2, TIAM1, ATG5, CSF2, EPHA1, BMP2, TNFSF13, ZEB2, RRM2, SOD2, CDO1, CSE1L, FPGS, IDH1, MIR106B, MIR26B, MPO, ING4, TLR9, PRKCB, AKR1B10, GGH, EXO1, KL, AKAP12, KLF2, STRAP, DHFR, MIR93, MMP11, ACKR3, KMT2C, SLC19A1, TK1, WRN, GALNT12, ABCC3, UMPS, MIR1271, AKAP9, ABCC2, GATA5, EPHA3, FAT1, FOLH1, AMACR, GAPDH, CHD5, MIR130B, MKI67, NR4A2, PPP2R1B, PRKCZ, RPS6KB1, SELENOP, BMPR1A, TFRC, ZNF217, SESN2, ATAD1, TP63, ABCB6, PTPRU, TENM1, APC2, POSTN, CFTR, CAPN10, CPE, PRIMA1, CYP2A6, BRINP1, DPEP1, EPHB6, ACSL4, EFEMP1, TPX2, LAMA1, PYCARD, IFNA1, LIFR, ARNT, PER1, XAF1, RTN4, SFRP4, SRSF6, TCF3, TCN2, PDGFD, IRS4, TNS4, EIF3H, SELENBP1, RASAL2, RASSF2, ABCC5, ABCC4, MCRS1, GPNMB, CACUL1, CTNND2, CUX1, ADAMTS15, SLC29A1, EPHA7, ERCC6, NTNG1, ABCA4, MTHFD1L, GATA4, GPX4, GSTM3, GTF2B, ACACA, VWA2, NME2, P2RX7, NUSAP1, ACSL5, SERPINB5, ASAP3, PRPS1, SLC12A5, PTPRS, SOX17, SCG5, CENPH, BMP6, TBX1, CAD, ZKSCAN3, FZD4, CD9, DHRS2, SF3A1, CD226, CHL1, PTPRT, CSMD3, UHRF2, LRRC3B, COL3A1, ADORA1, CTBP1, DDIT3, ADAMTS18, DPYSL2, EIF4G1, F5, FBLN2, FBN2, CD93, ACIN1, ZNF292, GSE1, VPS13A, ABCB10, LY96, SETBP1, TTLL3, GHRHR, GLI3, GRIN2A, BRF1, HDC, HK3, HRH1, APOB, IFNA2, TNFRSF9, LIG3, LRP2, CD46, NR3C2, NF1, NFATC1, SLC11A2, NRCAM, SDF4, PCSK2, ABCB4, PKHD1, ATP7B, TRPM7, ODAM, PRSS1, CD248, ADAMTSL3, RARB, RASGRF2, GNB4, RHEB, SFRP5, SKIV2L, FOXL2, TXNRD1, UNG, UQCRC2, KDM6A, ST8SIA4, ANP32A, OBSCN, CDC14A, RUNX1T1, ADAM19, LDB1, FOXH1, NEURL1, CABLES1, OTOP2, DMKN, PPP1R14A, NCR1, QKI, HOXD9, RASL11A, MAF, PAX8, CDH5, MIR1273C, LINC00673, CDH7, PQBP1, CALCOCO2, EFS, BCAS2, TFG, ABCA10, ABCA9, ABCA8, CORO2B, ZNHIT1, UNC13B, ADARB2, ARFGEF1, AKAP3, GNB5,

	ALDH1L1, DSTN, ADAM29, ERI2, TP53TG1, ADCY8, SLC22A9, OMA1, C8orf34, CSNK1A1L, SPACA7, B4GALNT2, CYB5D2, NLRP8, KDF1, CNTN1, COL4A6, COL7A1, CPO, EVC2, ZNF572, ADORA3, FRMPD2, SYT9, PHETA1, ZNF480, ZNF560, ZNF569, ABCC13, PUS10, CTSH, C2CD6, WDR49, CNTN4, JAKMIP1, ABCA13, IQUB, ERICH1, ZNF540, LONRF2, DEFA4, UGT3A2, HTR3C, DLG3, DMD, DNAH8, DNASE1L3, DPAGT1, EDA, VSTM4, SPAG17, AMZ2P1, SPNS3, PDILT, ABCA3, ETFDH, ETV1, FANCG, AMER2, GJD4, FCN1, FCN2, FERD3L, ITPRID1, ABCD2, FHL3, TFEC, PPM1E, CNKSR2, DIP2C, KDM2A, SNRNP200, MCF2L2, NCDN, LPIN1, FLNC, CAMSAP2, AGTPBP1, KCNH4, ABCA6, ABCA5, CBX5, PIK3R5, RAB38, NPAP1, EML2, TTLL10, GABPB1, GABRA6, FBXL2, GALNS, ZZZ3, RPAP1, EDRF1, ABCA12, CABYR, SACS, AMPD1, CNPPD1, SIGLEC7, SDCBP2, CPAMD8, SALL3, PCDH11X, STK32C, NPBWR2, ACTL9, ATP11C, ANK2, GRID1, GRID2, GRIK3, GRM1, SNX8, ANAPC4, PDZRN4, DONSON, H1-4, H1-5, NT5C, KRT73, HOXC9, HOXD1, HTR5A, CD300E, IPP, ISLR, ABCC6, KCNA10, KCND3, KRTAP10-8, CCDC9B, CFAP77, LAMB3, LIG1, C12orf76, LMO7, AMTN, MAP1B, MAP2, MYO1B, NDUFA1, NELL1, NID1, NODAL, OSBP, OVGP1, SHANK1, TBX22, AMDHD2, TNNI3K, ZDHHC2, ELOA2, PAIP2, WAC, WNT16, CINP, PEX5L, ERGIC3, PEG3, PGM1, ATP6V1E1, PKNOX1, PMM2, GPR87, TSPEAR, SETD4, ETAA1, CCDC93, ZNHIT6, PPOX, PHIP, ARHGEF10L, PANK4, VRTN, TMEM140, SLC22A15, PRG2, DNAH3, ZDHHC7, MFN1, INTS13, CCAR1, SCN3B, HR, PROS1, KCNQ5, PSMA2, FSTL5, PAK6, MFF, PSMC5, RNPEPL1, CORO1B, JPH3, DSCAML1, LRRC47, NLGN4X, ZNF624, ZNF471, RELCH, VPS18, PTPRN2, ABCD3, ABCD4, PZP, RAB5C, RALGDS, HIVEP3, RBP3, RCN1, TSKS, EXOC4, ABCE1, RPL6, SCNN1B, SCNN1G, DUSP21, DMRTA1, CLSTN2, LRRC4, NCAPG, MS4A5, CHST8, GMCL2, CDH22, ARAP3, PLPPR2, BCL11B, SLC26A10, SLC1A7, SMTN, SLCO1A2, BOLL, SNRPB2, SORL1, SPTBN2, ZFP36L2, CDKL5, SULT2B1, ABCC8, TCOF1, ICAM5, TUBB2A, ZBTB8B, WNT8B, ZNF43, MKRN3, ZNF155, ZNF232, DNALI1, CACNB1, CACNB2, CYREN, GDAP1L1, ZSCAN5A, RIC3, PARP8, ZNF385D, MMRN2, ZNF442, CSRNP3, CEBPA-DT, SLC66A2, PREX2, STAM, BPIFB2, TRAF3IP3, ZSCAN16, PBX4, SLC44A4, MLF2, LAS1L, RBM10, CAPN6, GFI1B, H2BC14, OR1E2, FBXO30, TOMM40L, TMEM164, HPS3, LZTS2, CCDC62, PIGO, LMNB2, IGSF21, HAT1, CCNB3, CBFA2T3, DCHS1, ABCB11, MCM3AP, KALRN, FAM110B, ARHGEF1, ZNF804A, MYLK3, SLC33A1, ZMYM4, DSEL, KCNB2, OLFM2, NDST3, SFXN1, AKAP6, MYOT, ABCG1, PPM1F, ZNF432, ACAP1, CLSTN3, STARD8, JAKMIP2, PAN2, P2RY14, MIR6808, DUSP10, GATA3, RNA5SP299, NKX2-3,

		CYP17A1, FGFR2, MYRF, MIR3164, COLCA2, COLCA1, RPL18P9, SPSB2, ETV6, RPL7P40, DIP2B, KRT8, SH2B3, FRY, BRCA2, RPS3AP46, RAD51B, LOC100131315, TMC3, HNF1B, BABAM1, FGFR3P3, RPS2P1, PREX1, CYP24A1, LAMA5, MIR4758, CABLES2, GPR143, NEK10, RPS27P4, MTHFD2P4, TERT, RPL26P19, C5orf66, MIR3925, ELOVL5, SLC22A3, LINC00536, UTP23, CCAT2, MXRA8, QRSL1P2, LINC00709, SLC25A28, MYEOV, PSMC1P8, ATF1, MIR5580, MEX3B, CASC20, XPOTP1, PFDN4, RPS21, SHROOM2, MRPS31P1, TOMM22P4, MAP3K1, PANDAR, RAD21
**DLBCL**	EZH2, MYD88, PIK3CD, CARD11, CD79B, IRF4, PVT1, MIR1208, EXOC2, LINC00824	EZH2, MYD88, PIK3CD, CARD11, CD79B, CD79A, CDKN2A, MALT1, ALK, CD274, MYC, ABCB1, PIK3CA, PIK3CB, PIK3CG, BCL2, PRDM1, CREBBP, EP300, JAK2, FAS, FOXO1, GLI1, B2M, KMT2D, ABCG2, CDK2, GNA13, RPS6KB1, STAT6, IKZF1, IRF8, NOTCH2, SKP2, FBXO11, CDK1, CLTC, PDPK1, PRDM11, NECTIN2, RANGAP1, SGK1, SOD2, BCAS2, PRDX4, TXNIP, PRDX3, SEC31A, SPEN, NOM1, SOD3, TRAF5, CCDC86, IMMP2L, CAT, IRF4, HLA-B, MIR6891, PVT1, MIR1208, EXOC2, LINC00824
**FLYMPH**	EZH2, BCL2, SETP16, MIR6090, HCG22, HLA-DRB9, HLA-DQB1, CXCR5, FLI1, C6orf15, HLA-DRB5, MTCO3P1	EZH2, BCL2, HLA-DRB1, BCL10, IGH, BCL6, CREBBP, KMT2D, TNFRSF14, MTHFR, KDSR, MTR, RRAGC, TYMS, MYD88, ATP6V1B2, TNFAIP3, EBF1, IFNA2, ATP6AP1, SETP16, GRAMD1B, MIR6090, PHLPP1, LPP, HCG22, HLA-DRB9, HLA-DQB1, CXCR5, FLI1, C6orf15, HLA-DRB5, MTCO3P1
**HANC**	PTEN, TNFRSF10B, EGFR, ING1, ING3, ADH1B, ADH7	PTEN, TNFRSF10B, EGFR, ING1, ING3, TP53, FGFR2, PIK3CA, PIK3CB, BCL2, VEGFA, RAD51, ERBB3, XRCC3, CDKN2A, ERBB2, GPX1, MAPK1, TGFA, DPYD, FANCD2, RARB, BCL2L1, CSF3, FAT1, AREG, MAL, TYMS, UROD, BAP1, CEBPA, CYLD, FGFR1, PRAME, GRP, MAPK3, STAT6, CDC73, APOBEC3B, FANCA, FANCC, FANCE, FANCB, FANCF, FANCG, FANCL, FANCI, FANCM, BRIP1, TP63, ADH1B, ADH7
**KDNCA**	FLCN, DIRC2, HNF1B, VHL, HNF1A, RNF139, OGG1, PRCC, MET, EPAS1, PTEN, SETD2, PBRM1, PTGS2, MTOR, TSC1, KDM5C, BAP1, LOC102724265, MIR1204, PVT1	FLCN, DIRC2, HNF1B, VHL, HNF1A, RNF139, OGG1, PRCC, MET, EPAS1, PTEN, SETD2, PBRM1, PTGS2, MTOR, TSC1, KDM5C, BAP1, NF2, MITF, PIK3CA, TFE3, TP53, ELOC, SLC49A4, KEAP1, ERBB2, ALK, GJB1, GSTM1, GSTT1, IL6, KRT7, TGM2, TNFSF10, ADIPOQ, SCARB1, KDR, TSC2, FLT1, GSTP1, BIRC7, MAPK8, SLC2A1, IL13, DAPK1, KDM6A, ARID1A, BIRC5, APAF1, ANXA4, APRT, L1CAM, POMC, PVALB, SFRP2, ATM, ALOX5, HSPB1, IFNA2, IL4R, PAK1, SOD2, ALDH1A1, FLT4, KRT8, TMEM127, RELA, TEK, NDRG1, BTG3, CRABP1, ACHE, PGK1, BCHE, CASP2, UNC5C, ACY1, PRAME, HSPD1, MUC4, PEBP1, DCLK3, CPQ, AKAP13, CSMD3, LRRK2, CNN2, CRYAB, ASB15, DNHD1, CTSB, CTSD, FAAH2, EEF2, ALAD, PDXDC1, SYNE2, ALOX12B, YIPF3, PNKD, OR4C13, VMO1, GRB7, HARS1, HSPA9, MSGN1, IL6R, FAM111B, KCNMA1, KRT32, LDHB, M6PR, SHANK1, PDHB, LRP1B, TET2, PIDD1, AP5M1, FMN2, SPTBN4, RYR1, SLC5A3, AHNAK, MLLT10, LMAN2L, CAPG,

		TAGLN2, CARD11, CRADD, FPGT, INPP4B, NAV3, RPL14, ZNF765, NLRP12, ZNF804A, ZNF536, CUL7, MERTK, ERN1, CILK1, ROR1, MYO3A, RIOK2, CDK15, WNK3, BMPR1A, BRD3, IFITM9P, LOC102724265, SSPN, MIR1204, CCND1, ITPR2, PVT1
**LGCA**	ERBB2, IRF1, SLC22A18, ERCC6, RASSF1, PIK3CA, EGFR, MAP3K8, CASP8, PARK2, DLEC1, FASLG, BRAF, PPP2R1B, MXRA5, KRAS, CYP2A6, CDKN2A, TP53, BRCA2, PTEN, GSTP1, ERCC1, STK11, VTI1A, FGFR2, MIR3164, KRT8, CHRNA3, HNF1B, BPTF, TP63, TERT, RPL26P19, BAG6, APOM, BTNL2, RPL32P1, FOXP4-AS1, MYEOV, MAP3K1, HLA-DPA2	ERBB2, IRF1, SLC22A18, ERCC6, RASSF1, PIK3CA, EGFR, MAP3K8, CASP8, PARK2, DLEC1, FASLG, BRAF, PPP2R1B, MXRA5, KRAS, CYP2A6, CDKN2A, TP53, BRCA2, PTEN, GSTP1, ERCC1, STK11, BAP1, VHL, CDH13, AKT1, CHEK2, CHRNA3, CHRNA5, CHRNB4, CLPTM1L, TP63, FEN1, RAD52, NOTCH3, IL24, CTNNB1, FHIT, CD274, GSTM1, IL6, IL10, MET, OGG1, MAPK1, TERT, WT1, CDKN1A, CDKN1B, CRP, MAPK14, CYP1A2, CYP1B1, CYP2E1, CYP24A1, ACE, EPHX1, ERBB3, ESR1, FGFR1, FOXM1, GSTT1, NRG1, APC, HRAS, IFNG, FAS, IL1B, MIR146A, MIR155, MIR21, MIR31, MCL1, MMP1, MPO, MTHFR, MYC, PRKN, PCNA, SERPINA1, MAPK3, MAP2K7, RAF1, RARB, CCND1, ACTB, SOX2, SOX9, SPP1, TGFB1, TNF, TP73, TYMS, XPC, CAV1, RUNX3, BECN1, BIRC5, HMOX1, MIR193A, TLR4, DNMT3A, EGR1, GPX1, IL2, MIR30A, TGFBR2, PRDX1, ROBO1, SFTPD, DAPK1, DPYD, ANXA2, JUN, MIR34B, MIR98, NOS2, MIR4435-2HG, CCN2, GJA1, PDCD4, GCLC, APOE, PON1, SFTPB, DDR1, CASC1, USP18, EFEMP1, FGFR2, GAST, GATA6, HES1, MIR222, MARCKS, MMP10, ERGIC3, RTEL1, MIR410, STAT5A, TFRC, WNT5A, CXCL14, PRDX6, PTGIS, BCL2L1, TTR, ADA, JAG1, FOS, RICTOR, GPX3, CCN1, MIR10A, MIR34C, UGT2B17, SLC7A5, RIOX2, SOX30, CEACAM1, RAMP2, CDKN1C, DAB2IP, FOSB, JUNB, JUND, NFYA, PTMA, SLC3A2, CHRNA7, FGF9, GJB1, APOA1, HTRA1, TEP1, BHLHE41, DOK3, CALML3, CCND2, SELENBP1, DOK2, COX17, ADAM28, PTPRT, EEF2, EMX2, HEY1, ANKRD18A, GC, SND1, SLCO1B3, PYCARD, APOC3, LECT2, MIRLET7BHG, MIR127, MIR154, MIR224, MIR370, ATOX1, NOTCH2, NPPA, PPBP, AZGP1, MIR432, MIR494, RAD21, ALX4, CCL18, SELENOP, MIR487B, CA12, CCNG1, CLCA2, MIR938, CCAT1, SPRY2, SMC2, CES1, EHMT2, CHRNA2, ACSM1, CLTB, COL6A1, CPE, LMNTD1, DNASE1L3, DOK1, EFNB2, A2M, FOSL2, RCHY1, ANK3, GRB7, HILPDA, ID3, IDS, IGBP1, MIR136, MPP1, MIR302D, MIR369, SIDT2, CHST15, PGGT1B, TMEM45A, EAF2, MIR409, MIR511, RIT1, AVPI1, ATG101, SMARCC1, STIM1, SERPING1, TSHR, TYRP1, IL1R2, STN1, CWH43, IKBKG, PDLIM4, RNASET2, MAP4K4, IER2, SECISBP2L, IQSEC1, MIR6808, VTI1A, MIR3164, ACVR1B, KRT8, FRY, RAD51B, HNF1B, BPTF, BABAM1, RPS2P1, NEK10, RPL26P19, BAG6, APOM, BTNL2, RPL32P1, LINC01276, FOXP4-AS1, UBA52P6, SLC17A8, MXRA8, MYEOV, XPOTP1, MAP3K1, HLA-DPA2, DMRTA1, NR1H4
**LIVCA**	TP53, AXIN1, MTUS1, CDKN3, PIK3CA, CASP8, PDGFRL, CTNNB1, MET, APC, IGF2R, HNF1A, CDKN2A, IGF2, KRAS, HTATIP2, ARID2,	TP53, AXIN1, MTUS1, CDKN3, PIK3CA, CASP8, PDGFRL, CTNNB1, MET, APC, IGF2R, HNF1A, CDKN2A, IGF2, KRAS, HTATIP2, ARID2,

	FOXM1, GPC3, GNMT, MYC, ABCB1, PTEN, PTGS2, PTK2, HAMP, SKP2, TERT, TGFA, CCNE1, CDK1, KIF1B, MICA, LOC101929072, HLA-DRB1, HLA-DQB1, HLA-DQA1, MTCO3P1	FOXM1, GPC3, GNMT, MYC, ABCB1, PTEN, PTGS2, PTK2, HAMP, SKP2, TERT, TGFA, CCNE1, CDK1, ABCB4, IQGAP2, MECP2, NR1H4, ARID1B, A2M, HRAS, NFE2L2, SERPINA1, STAT4, DEPDC5, IDH1, CDK14, SPRTN, HOTAIR, MICA, CDK4, CDKN1B, YAP1, CEBPA, UBD, HPSE, COPS5, CRP, CYP1A1, CYP1A2, CYP2B6, CYP2E1, ACE, AFP, E2F1, EGF, EGFR, EGR1, EPHX1, EPO, ESR1, EZH2, F2, FASN, FBP1, FOS, MTOR, GAPDH, GJB1, GLI1, GLUL, CD274, GSTM1, GSTP1, HGF, APEX1, HSPA5, HSPB1, BIRC5, APOA1, IFNA1, IGF1, IGF1R, IL6, CXCL8, IRS1, AR, JUN, STMN1, MIR122, MAGEA1, MAT1A, MKI67, MMP2, MMP9, NEK2, NME1, NOTCH3, PKM, PLK1, PPARG, AKR1B10, RAC1, RB1, CCND1, ACTB, CXCL12, SFRP1, SLC2A1, SLC5A5, SREBF1, STAT1, AURKA, TGFB1, THY1, TLR4, TNF, ARID1A, NR0B2, RUNX3, TNFSF10, CCNB1, SOCS3, CD34, FGF19, NAT2, CEBPB, ATG7, LPA, MAGEA3, MAGEC2, SCD, SOD2, TFPI2, IGF2BP1, FOSB, UHRF1, HSPA9, CCN1, PCK1, BCL2L1, SLC22A1, BRAF, VIPR1, BTG2, AURKB, GDF15, KMT2B, PCLAF, CYLD, ENO1, MAD2L1, MCM2, ATM, PRDX2, TOP2A, TYMS, PTTG1, CCR1, CYP17A1, DPYD, ACSL4, TPX2, KIF4A, RACGAP1, KIFC1, KMT2A, PGK1, RARA, ROBO1, E2F8, MELK, CDH13, CDCA5, ADRA1A, GHR, ACACA, HOXA13, MPO, MYBL2, NFKBIA, PRDX1, ACOX1, DEPDC1, PBK, RRM2, CA2, CD276, AXIN2, IRS2, IQGAP1, PRC1, CD5L, CD163, ISG15, CDC20, CDC25C, CDKN2C, NDC80, CEBPD, IGF2BP3, ESM1, CYP2C8, DCN, FGF4, PDIA3, HMGCR, IL1RN, IRF2, MT1A, MLXIPL, NUSAP1, YTHDF2, PDK4, PHB, CEP55, NCAPG, CSMD1, MIR539, SSX1, MIR615, TTK, ZFP36, ARHGEF39, FCN3, SOCS2, EXO1, TRIP13, DLGAP5, KIF14, MIR885, TROAP, FST, CAP2, CENPF, LYVE1, UBE2C, ADAMTS13, ADRA2B, RCAN1, ECT2, SKA1, FCN2, FGF3, ASPM, MTBP, UBE2T, HMGB2, HOXA10, MT1E, MT1F, GMNN, ANLN, HJURP, RPS6KA3, SLC2A2, SREBF2, BUB1, BUB1B, UCHL1, KIF18A, BCO2, TAGLN2, TRIM24, APLN, CCNB2, SLIT2, ADAMTS1, CDC6, CENPA, CENPE, VASN, ADH4, IQGAP3, COMT, CTSD, CYP4A11, EGR2, FABP5, FANCD2, CPEB3, GDF2, GNAO1, SFN, FLVCR1, PYCARD, GYS2, ANXA4, HGFAC, HOXD9, IL1RAP, ASPG, LIFR, ME1, MVK, NNMT, HERC5, ZDHHC2, CLEC1B, GTSE1, DTL, PDGFB, PITX1, TCIM, KIF15, KMT2C, CCL14, BID, HHIP, SHH, TALDO1, TCF19, TSC1, NR1H2, WDR76, FAM83D, CDCA3, NUF2, MARCO, CBR1, INPP4B, DIRAS3, PKMYT1, KIF23, CXCL14, KIF20A, ERP29, ZWINT, PARK7, GPR182, VSIG4, THEM4, JDP2, COL15A1, UROC1, CP, CRHBP, TTC36, CSPG4, BMPER, OIT3, ECM1, CELSR3, NLRC3, SKA3, SCAP, CEP131, NCAPH, GNAZ, SLC25A47, HMMR, APOF, APCS, LINC00221, RND3, MAGEA6, MAPT, MT2A, ACLY, TONSL, ACO2, HAO2, CFP, MED1, LRRC1, MCM10, STAB2, DEPDC1B, PTH1R, RPS6,

		CCL3, TGM3, TH, TST, C9, UMPS, VCAM1, ZIC2, ZNF23, CENPM, EPS8L3, KDM8, CHAF1B, RTP3, TATDN1, MFSD2A, ZIC5, EIF3H, CCNA1, ARHGAP11A, DNAJC6, TRAIP, CLEC4M, TACC3, ATP5PD, NKILA, COLEC10, CETP, LILRB5, KIF2C, INMT, RNF157, ADD1, DCAF4L2, PPP4R3C, KIF18B, CCBE1, CKAP2L, CDCA2, DBH, AKR1C2, OLFML2A, COX7B2, DNASE1L3, ETFA, FDFT1, PHLDA1, GABRD, PAMR1, OLFML2B, GPM6A, WDR62, EHD3, HSD3B2, CLEC4G, IGBP1, IGFALS, ITIH3, FAM111B, KCNN2, KIF11, CENPW, SLC22A10, FAM180A, LCAT, LETM1, C14orf180, MSH5, ORC1, AADAT, CYP39A1, PLAC8, MBTPS2, PGD, SERPINA4, PKP1, TMEM70, PPP1R1A, CDCA8, FANCI, NEIL3, LRRC59, ASF1B, EAF2, PLXDC1, CLTRN, SPC25, MIR520B, GPR158, GBA3, PYGL, PZP, RRM1, CSRNP1, SLC26A6, XAGE1B, FAM72B, TK1, INS-IGF2, UBE2E2, CENPU, DIPK2B, FBXL18, MOGAT2, TEDC2, CDT1, CDC45, PITPNM3, PLVAP, ANGPTL6, MRO, SRPX, ZIC4, RAD54L, CNDP1, RSPO3, ADGRG7, CNTNAP4, TSLP, PNPT1, MBTPS1, FATE1, CCNF, TICRR, USP2, TIMD4, LRAT, PRDX6, GINS1, KBTBD11, KIF1B, GRIK1, LOC101929072, HLA-DRB1, HLA-DQB1, MTCO3P1, HLA-DQA1, HLA-DQA2
**OVCA**	BRCA1, PIK3CA, RRAS2, PARK2, CTNNB1, AKT1, SEPT9, CDH1, OPCML, BRCA2, ERBB2, KRAS, MLH1, MSH2, PTEN, TP53, RAD51C, BRIP1, PMS2, ESR1, CCNE1, RAD51D, BRAF, RSPO1, FGFR2, MIR3164, KRT8, HNF1B, NSF, SKAP1, BABAM1, HAGLR, HAGLROS, TIPARP, RPL26P19, LINC00824, LSM1P1, IFNL3P1, HOXD3, MYEOV, MAP3K1, LINC00977, RPL31P42, IFNL3	BRCA1, PIK3CA, RRAS2, PARK2, CTNNB1, AKT1, SEPT9, CDH1, OPCML, BRCA2, ERBB2, KRAS, MLH1, MSH2, PTEN, TP53, RAD51C, BRIP1, PMS2, ESR1, CCNE1, RAD51D, BRAF, MSH6, PRKN, STK11, SMARCA4, TP63, EPCAM, BNC2, RNF43, BABAM1, CHMP4C, MSLN, EGFR, FOLR1, MTOR, TIPARP, IL6, CXCL8, MYC, PMS1, MAPK1, STAT3, SKAP1, MUC16, TERT, CDKN1B, TUBB3, FASN, MET, ZEB1, AKT2, SPARC, TNFSF10, BIRC5, SULF1, YAP1, XIAP, CYP1B1, MAPK3, CAV1, EDNRA, FGF1, AREG, SKP2, WNT7A, GADD45A, MKI67, KLK10, SLC5A5, DPH1, MECOM, MAP2K1, SOD2, TYMS, HDAC6, CDK12, ATP7B, TP53BP1, ATG7, ARL11, NR5A1, SPDEF, NME2, DESI2, ATR, BCL9, SLC2A1, CCND2, SELENBP1, ATG5, MRE11, NECTIN2, RBL2, BAP1, DLC1, CAMKK2, EREG, ERCC4, JMJD6, ALOX5, ANXA3, HOXD1, IL6ST, SOD1, TLR4, URI1, DYRK1B, GPR150, HOXD11, PIK3R1, PRTFDC1, ITGA8, SRSF10, POP4, DOK1, ERCC6, ALOX12B, KANSL1, GRIK2, HOXB9, HOXD9, IL11RA, AQP3, SLC22A10, ATF3, HAUS6, PPP1CC, LRRC59, TRMT11, WDR77, PLEKHF1, C19orf12, CCNH, MACIR, LRRC46, PRC1, FAM107A, MIR6808, RSPO1, MLLT10, FGFR2, MIR3164, KRT8, FRY, RAD51B, HNF1B, NSF, HAGLR, HAGLROS, RPS2P1, NEK10, RPL26P19, LINC00824, LSM1P1, IFNL3P1, HOXD3, MXRA8, MYEOV, XPOTP1, MAP3K1, LINC00977, RPL31P42, IFNL3
**PACA**	RBBP8, TP53, ACVR1B, SMAD4, STK11, KRAS, PALLD, BRCA2, PALB2, BRCA1, CDKN2A, RPL23AP16, LINC00867, PDX1-AS1, RNY1P8, LINC00673, BACH1, TFF2, TERT, CLPTM1L, LINC-PINT, ABO, NR5A2, PRLHR, MARK2P12, TFF1	RBBP8, TP53, ACVR1B, SMAD4, STK11, KRAS, PALLD, BRCA2, PALB2, BRCA1, CDKN2A, EP300, NR5A2, ABO, PTEN, TERT, APC, SPINK1, TSC2, BAP1, EGFR, HIF1A, STAT3, WT1, MSLN, CTNNB1, AKT2, CXCL8, PDX1, KDR, MMP2, MMP9, MYC, ATM, PTGS2, SST,

		TGFB1, TNF, TYMS, TNFSF10, CD44, CD24, PPARG, MEN1, LDHA, SLC29A1, PLAU, SOD2, SSTR2, IL24, DPYD, IFNA1, AXL, MAP2K4, TGM2, CDH1, NR4A1, EPCAM, PTCH1, TFPI2, NRP1, LINC00673, GADD45A, RNF43, TYMP, RAP1GAP, RB1, KLF5, VHL, AHR, EFEMP1, FGF13, PTHLH, CCL20, SSTR1, TNFRSF10B, CFLAR, DAXX, FXYD3, MIR1179, ANXA10, FOXP1, IFNA2, NFKBIA, SSTR3, BUB1, VEGFB, TP63, CCAT1, CLPTM1, CNR1, CNR2, GAGE1, RABL3, IFNA5, LY6E, ETAA1, ATRX, BCL2L1, SYCP1, SUGCT, ZNRF3, BCAR1, RPL23AP16, LINC00867, PDX1-AS1, RNY1P8, BACH1, TFF2, FAM19A5, CLPTM1L, DAB2, LINC-PINT, CASC8, PRLHR, MARK2P12, C1D, TFF1, CASC11
**PRCA**	EPHB2, BRCA2, PTEN, MAD1L1, HIP1, CD82, ZFHX3, KLF6, AR, MXI1, CDH1, FGFR4, MSR1, CHEK2, HOXB13, RNASEL, MSMB, ELAC2, EHBP1, APC, SPOP, CTNNB1, IGF1, NKX3-1, KRAS, CCND1, TGFBR2, KCND3, GOLPH3L, MDM4, RPL23AP61, FGFR2, MIR4686, RPS2P38, MIR3164, MMP7, TUBA1C, KRT8, GLULP5, LINC00421, FERMT2, VPS53, HNF1B, ZNF652, LOC102724596, CASC17, DPF1, PCAT19, KLK15, KLK3, MIR4752, GGCX, TANC1, ITGA6, ADNP, ZGPAT, RPS25P10, CXorf67, XAGE3, TEX11, CHL1-AS1, PPATP1, SIDT1, PRKCI, SKIL, AFM, PDLIM5, TERT, CTD-2194D22.4, RPL26P19, RFX6, RGS17, SLC22A1, SLC22A3, RPS26P30, JAZF1, FAM60DP, EBF2, FAM84B, PCAT1, PRNCR1, SRRM1P1, CCAT2, CASC8, RAD23B, AGAP7P, ASCL2, LUZP2, MYEOV, PRPH, TBX5, PARP4P2, PPP1R14A, KLK2, LILRA5, VAMP8, BIK, NUDT11, SLC7A3, LINC01266, LINC00506, CLDN11, MAP3K1, SLC22A2, ASS1P11, CCAT1, CASC11, HMGN2P32	EPHB2, BRCA2, PTEN, MAD1L1, HIP1, CD82, ZFHX3, KLF6, AR, MXI1, CDH1, FGFR4, MSR1, CHEK2, HOXB13, RNASEL, MSMB, ELAC2, EHBP1, APC, SPOP, CTNNB1, IGF1, NKX3-1, KRAS, CCND1, TGFBR2, SRD5A1, RXRA, EGFR, PCAT1, KLK3, NBN, PIK3CA, BRCA1, HNF1B, TP53, SLC22A3, CTBP2, RFX6, LDAH, FOXP4, LMTK2, PALB2, FGF10, POLK, CDKN1B, CLU, EGF, EGR1, ERBB2, AKT1, ERG, ESR2, EZH2, FOLH1, AMACR, GSTP1, HIF1A, IGFBP3, IL6, CXCL8, MMP9, MYC, PTGS2, BCL2, CXCL12, SRD5A2, STAT3, TGFB1, TMPRSS2, VDR, VEGFA, CAV1, ZBED1, NAT2, CDKN1A, NDRG1, VAV3, AGR2, CENPF, IL24, CHD1, COMT, CREBBP, PARP1, DAB2IP, CYP1A1, CYP1B1, CYP3A4, CYP17A1, CYP19A1, ACE, NQO1, DNMT1, DNMT3B, AHR, ERCC2, ESR1, ETV1, ETV4, FGF2, FOXO1, SIRT1, BRD4, GJA1, SETD2, NR3C1, GRPR, GSK3B, GSTM1, GSTT1, HMOX1, FOXA1, HPN, APEX1, HSD3B1, IL10, GSTK1, KLK2, LEP, MIR21, EPCAM, MDM2, MET, MLH1, MME, MTHFR, ATM, NFE2L2, NOS3, OGG1, SERPINE1, PAWR, PIK3CB, PIK3CD, PLAU, CCHCR1, RNF43, MAPK3, PTHLH, BGLAP, SHBG, SLC5A5, SOD2, SOX2, SPINK1, ST14, AURKA, TERT, TLR4, TPD52, UGT2B15, UGT2B17, VIM, VIP, XRCC1, CALCA, PSCA, CALR, NCOA3, CASP9, AKR1C3, TNFSF10, MBD2, CCND2, GDF15, CAMKK2, CTSB, CYP3A5, ETV5, TMEFF2, ANXA1, ICAM1, IL18, JAK2, EAF2, AZGP1, NCOA1, SCHLAP1, NCOA2, JAZF1, HOXD3, SERPINF1, PML, SELENOP, TGFA, NRP1, IL27, SLC39A1, HSD17B3, IDH1, IGFBP7, IL2, KMT2A, MPO, TRPM4, DCAF6, PRKDC, BAX, CYP3A43, BMP7, ADAM9, ITGB3, ERBB3, NEAT1, GPX3, HSD3B2, STMN1, PON1, PPARA, PAK6, BRAF, TOP2A, HMGN5, UXT, NAT1, BAD, TLR6, ADRB2, ALOX5, KLK5, IRS1, ATF3, NGFR, PIK3R1, TET2, SULT1E1, SULT2A1, TPT1, DDR1, CD276, EIF3H, TNFRSF10A, GREB1, EHF, BIRC5, MIF, ABCC4, TXNRD2, SERPINA3, CTSD, CYP2E1, DEFB1, LPAR1, EPHX1, FHIT, GHR, GNMT, AOX1, LPL, MIR106A, MMP13, MSH3, MT2A, NUCB2, ZBTB7A, PIK3R2, ADI1, PRKCZ, RB1, RRAS, CX3CL1, SMARCA1, SPOCK1, SSX2, SULT1A1, PRDX2, EZR, XPO1,

	USP7, STEAP4, RUNX1, MED12, PLAUR, RLN2, HDAC6, CDK2AP2, BAZ2A, LZTS1, CLDN3, CLDN7, DAG1, GADD45A, FOXC1, ABO, GSTA1, GSTM3, ANXA3, IGF2R, FASLG, IL16, LIFR, MAP3K1, PDHA1, CDK12, PODXL, B2M, VPS52, SLC7A1, MAP3K7, VCP, KMT2D, H4C8, IL17RC, ONECUT2, CREB3L4, GCNT1, JAK1, MUC4, PPP2R2A, TIMP4, UMPS, CYP7B1, MYBBP1A, WASF3, STARD3, CSRP1, EMP1, ACSL4, FBLN1, GGT1, PDIA3, HMGB2, HPGD, HSD17B1, HSPA1A, IL1RN, ITPR1, ARG2, LAMC1, LDHB, LRP2, MBD1, PCDH8, PDE4D, PENK, PMS1, ATR, IL17RD, PARD3, PVR, RAD23B, RAG1, KLKP1, BNIP3, TERC, KDM6A, ZIC2, ZBTB16, TBL1XR1, TXNDC5, CAPNS1, PPFIBP2, EIF3A, ALDH1A2, CCNH, LITAF, BCAR1, NCOR1, SETDB1, CDH13, CTCF, ADAM28, CST6, CYP2C19, ERF, SSBP2, ABR, EFEMP2, HNRNPH1, HRAS, ID3, INS, JUP, DCXR, PDP1, PRKACB, EIF2AK2, PRNP, BMPR1B, TMF1, HSP90B1, TYMS, PCDH11Y, USO1, CPNE3, CD9, SPON2, PGRMC1, RALBP1, CBX1, AKAP13, SLC31A1, CRYAB, ALAD, ETV3, EWSR1, SPEN, SF3B1, GALNT3, SENP6, PHGDH, ANXA4, IGFBP6, M6PR, MTAP, PAX6, IRAK4, LRP1B, CASZ1, PPL, NOL8, ATAD3A, PPP3CA, PRSS8, PTPRC, PTPRK, NECTIN2, ARID4A, ALOXE3, ROBO1, RPL10, RPS19, ABCG5, SULT2B1, TAP1, TRAF1, WNT10B, EPS8L3, ARHGEF5, BAP1, MBTPS1, CBR1, MACIR, HERPUD1, CDC27, TOM1L1, CDH12, PDZK1IP1, PATJ, OLFM1, FBLN5, IVNS1ABP, CELF2, STARD10, KDELR1, SERINC3, ERP29, SPINK5, CHD3, ADAMTS8, PKP3, CHST14, TIRAP, ANTXR2, TPP1, PAQR4, CNN3, COL5A1, COL15A1, SPATA18, NCOA7, ASZ1, TAF1L, SESN3, CST1, PYHIN1, IGSF5, CYP2A6, CYP2C18, CYP11B2, DNASE1L2, EHHADH, ARID2, UNC13D, EMP3, DHX30, ZNF292, ERP44, FAF2, ARL6IP1, JMJD6, PDS5A, RPRD2, MGA, JADE2, AHCYL2, SDF2L1, ALOX12B, CIZ1, CLIC4, ZBTB20, LCE2B, PLEK2, TJP3, TNFRSF21, DHDH, GNG5, GOLGA4, TTC9C, RGMB, GRB7, KLF15, THYN1, PSMC3IP, HBG1, HBG2, FOXA3, HOXB9, HSP90AB1, IL6ST, SP5, LAMB2, MC2R, ACHE, MYCL, NAGLU, NFIC, CNOT3, NPPA, NPR3, P4HB, NOX3, COL5A3, ITSN2, CRYL1, RLIM, PIGP, WAC, PTRH2, RASD1, SLC26A4, PDZK1, PGAM2, SERPINB10, PITX3, RAB4B, PRRX1, ATP7B, WNT4, HAO1, DNAJC10, SAMD9, RNF31, APPL2, RFK, TBC1D2, STAB2, NAGK, CHD7, RNF130, SELENOS, ASH1L, DNAJC3, DIABLO, TCEAL7, SMARCAD1, NIPAL3, USP28, KMT2C, ENPP5, RNASE4, ROBO2, RPL11, RPL12, RPN2, ACSM3, SIL1, ITSN1, NDST4, SLC4A2, SLC12A2, SSR2, ZFP36L2, SURF4, TBXAS1, TBX3, TCN2, TCP1, TLR5, TMOD1, TMSB4X, TPBG, TPD52L1, TST, U2AF1, UCP3, UCK2, ZFAND5, BRPF1, MYH14, GSTCD, KAT6A, AAAS, CLPTM1L, ARID1A, H2BC8, GUCD1, CHD6, ACRBP, CUL3, NDST2, BCAS1, DEGS1, CAV2, B4GALT4, MATN4, CREG1, ZNF160, CLDN9, ZMYM3, INTS4, FBXO44, GRHPR,

		GSTO1, EI24, KEAP1, REC8, MIR6808, KCND3, GOLPH3L, MDM4, USH2A, PIP4K2A, MARCH8, RPL23AP61, TRIM8, LINC01153, FGFR2, MIR4686, RPS2P38, MS4A5, MIR3164, MMP7, LOC728114, TUBA1C, KRT78, KRT8, GLULP5, LINC00421, FRY, RNU6-66P, FERMT2, SIX1, RAD51B, UBE3A, VPS53, FLJ40194, ZNF652, LOC102724596, CASC17, SALL3, BABAM1, DPF1, PCAT19, KLK15, MIR4752, GGCX, VAMP8, TANC1, ITGA6, COL6A3, FARP2, RPS2P1, ADNP, ZGPAT, RPS25P10, SHROOM2, CXorf67, NUDT11, XAGE3, TEX11, NLGN3, CHL1-AS1, NEK10, PPATP1, LINC00506, SIDT1, EEFSEC, PRKCI, SKIL, HNRNPA1P67, AFM, PDLIM5, LOC101929468, CTD-2194D22.4, RPL26P19, RASA1, NEDD9, CASC15, NOTCH4, ARMC2, RGS17, SLC22A1, RPS26P30, TNS3, SGCZ, FAM60DP, EBF2, FAM84B, PRNCR1, SRRM1P1, CCAT2, CASC8, DMRT1, BMS1P9, MXRA8, AGAP7P, ASCL2, LUZP2, MYEOV, PRPH, RPL7P41, TBX5, PARP4P2, RNU7-89P, SIX4, MIR6129, ATP9B, PPP1R14A, LILRA5, MLPH, XPOTP1, BIK, CENPVP3, SLC7A3, GJB1, LINC01266, CLDN11, RNU6ATAC5P, SLC22A2, ASS1P11, CCAT1, CASC11, AQP7P4, HMGN2P32
**SKCM**	MC1R, CDKN2A, STK11, POT1, PTEN, CDK4, BRAF, MITF, XRCC3, TERT, TYR, HRAS, ERBB4, GNA11, GNAQ, NRAS, MAP2K1, TP53, BAP1, MAP2K2, NF1, RAC1, LOC100996521, YWHAZP5, IFITM9P, OCA2, FTO, CDK10, AFG3L1P, RALY, PIGU, MYH7B, MIR499A, SLC45A2, IRF4, MTAP, SLC25A6P5, RPL23AP59, CCND1, EXOC2, MIR8081	MC1R, CDKN2A, STK11, POT1, PTEN, CDK4, BRAF, MITF, XRCC3, TERT, TYR, HRAS, ERBB4, GNA11, GNAQ, NRAS, MAP2K1, TP53, BAP1, MAP2K2, NF1, RAC1, PPP6C, PARP1, CTNNB1, ERCC2, AKT1, KIT, MDM2, MTAP, ATM, SLC45A2, PIK3CA, RAF1, BRCA2, XPC, TRRAP, PDGFRA, CASP8, ERCC4, FTO, ERCC5, SDHD, DDB2, ERCC3, MX2, XPA, AKT3, CDK2, HPSE, CSF2, CTLA4, E2F1, MLANA, FLT1, PRAME, ABCB5, IFNA1, IFNA2, IFNB1, IFNG, IL2, LGALS3, MAGEA1, MET, MELTF, PDCD1, POMC, PPARG, PTGS2, CCND1, RET, S100A4, PMEL, TLR4, TNF, TYRP1, VEGFC, KLRK1, ALK, TNC, CRYBG1, FASLG, ASIP, AURKA, HDAC6, ALDH1A1, ARNT, CUX1, MMP8, GDF15, ERCC1, APEX1, SETDB1, EPO, PTK2B, FASN, PHGDH, PAK1, LXN, RAP1GAP, ROS1, NDRG1, STAG2, CSF3, EPOR, GRIN2A, RASA2, SLC5A5, SYK, TFPI2, GAB2, EP300, JMJD6, SUZ12, GLO1, GRM3, MIR340, NF2, PPARD, STX17, PRDX2, FAT4, CAPN1, HOXB13, STIP1, IL1RAPL1, BATF2, CLU, CST6, CTH, ALDH1A3, KDM1A, ABL1, ASF1A, SLC17A5, HSPA1A, MAP3K5, MAP3K9, MYO7A, PAX4, PLCB4, TNFRSF19, WFDC1, RAC2, RPS3, SIPA1, SYT4, CASP10, CUL3, ACVR1, RIN1, MAGEC1, CDKN1C, RPP38, DRAP1, RAI1, PRSS23, TMEM132B, DDIT4L, NUS1, CLNS1A, CPT1C, COL2A1, ZNF831, CP, CYP1B1, CCDC63, TMEM30B, DCC, GPHA2, DLG2, DSC1, EDA, KHDRBS2, EPB41, F2RL1, ZNF438, SLC39A12, ARL5B, NT5DC1, HS3ST5, RFX6, KDM4B, KDM4C, TSPYL4, UBR4, NPAP1, VGLL2, QPCT, PPP1R14B, CNNM1, SLC25A45, CD163L1, DCBLD1, DNAJC15, SAC3D1, RAX, HDAC2, HOXA9, HOXD12, TAS2R60, IFNA5, INCENP, KLRB1, ARL2, MPST, MYO1B, NFKBIE, NPPA, PAX6, HIKESHI, PIP4K2A, LRP1B, WDR74,

		MTPAP, ACER3, ECHDC1, PCDHB8, AKR1B10, GOPC, GPAM, FAM160B1, PTPRK, PTPRO, TRAPPC1, DNAJC1, IQCH, SLC6A11, SLC15A2, UGT2B10, MRPL49, SF1, EPHX3, ADAMTS20, MLLT10, CUBN, SLC35G2, C11orf68, EIF1AD, CBS, PKMYT1, PTER, KDM4A, BZW1, USP6NL, JAKMIP2, REC8, LOC100996521, OBFC1, YWHAZP5, IFITM9P, OCA2, CDK10, AFG3L1P, RMDN2, ALS2CR12, RALY, PIGU, MYH7B, MIR499A, PLA2G6, IRF4, RAD17P1, SLC25A6P5, RPL23AP59, EXOC2, AHR, MIR8081
**STCA**	ERBB2, MUTYH, IRF1, PIK3CA, CASP10, KLF6, APC, FGFR2, KRAS, CDH1, IL1RN, IL1B, MET, ATM, MUC1, ASH1L, PRKAA1, MEF2C-AS1, LRFN2, DNAH11, PSCA, MIR3660	ERBB2, MUTYH, IRF1, PIK3CA, CASP10, KLF6, APC, FGFR2, KRAS, CDH1, IL1RN, IL1B, MET, ATM, CDKN1A, PRKAA1, TP53, CHEK2, CDKN2A, ZBTB20, ERBB3, DLC1, AXIN2, HOTAIR, AFP, DNMT1, DNMT3B, DPYD, EGFR, ERCC1, ERCC2, ALB, FHIT, GAST, GSTP1, IL6, CXCL8, SMAD4, MLH1, MMP7, MSH2, MTHFR, MUC1, MYC, SERPINE1, PLCE1, PPARG, CHFR, MAPK1, MAPK3, GKN1, PTGS2, CCND1, SNAI1, STAT3, TNF, TWIST1, TYMS, XRCC1, XRCC3, PSCA, ARID1A, CAV1, RUNX3, CD44, CDK4, BAP1, RHOA, REG4, APEX1, PLAU, KRT20, BRAF, TIMP3, CCAT1, ACE, ENO1, IGFBP3, LGALS3, PRKAB1, PRNP, KLK10, FAT4, CASP8, BIRC5, CDKN1B, AHR, ZNRD1, HMOX1, AREG, NOTCH2, KMT2C, SOD2, IL32, ARL6IP5, JUN, MT2A, NPM1, XAF1, MAPK8, BMP2, TGFA, AURKB, ADRB2, TYMP, F2R, HRAS, HSPB1, IGFBP7, ITGA5, MUC6, PHB, PTPA, FSD1, MTSS1, CTNNA1, FBP1, PYCARD, KISS1, KMT2A, SERPINB2, PAX6, WWOX, PRKCB, MSLN, WIF1, CLCN3, ECM1, ING1, NOS3, UBR5, PDHA1, ATR, RNF43, RARRES1, BCL2L1, RORA, BDNF, UMPS, MIA, SCRN1, EBI3, CKB, LRRC3B, DCBLD2, CLDN3, CST1, HBEGF, ECHS1, EEF1A2, FGG, ALOX5, PRDX5, GLI3, ANXA5, HSPA8, HSPD1, ICAM2, TNFRSF9, KRT8, FADS1, MLF1, MMP10, MSX1, NT5E, COPS7A, HIKESHI, NBAS, SERPINA1, PLAGL1, POLE, PPIA, PTPRG, RARB, RGS2, RPS6, BID, SLC1A2, BNIP3, TBX3, THBD, KISS1R, SPZ1, MIR22HG, AKR1C3, SLC16A3, CDH2, BIRC2, RPL15, MAP3K6, ZNF667-AS1, TRAP1, CDKN2D, CNPY2, FST, NOP56, CCT7, CTSC, PTPRT, CLN3, PLIN2, MBD3L2, CTNNA2, CTSL, ADRB1, CYP2A6, DDB1, GADD45A, DES, DPAGT1, AGTR2, EEF1A1, ALDH1A3, FKBP2, ALDOB, FAM168A, BOP1, ATP6V0D2, FYN, SERBP1, GREM1, ACAD8, SNX5, FILIP1, PRPF19, MRPS18B, MRPL13, ABT1, CPSF1, HNRNPL, HOXA2, APOA1, HTR1A, ID4, IDH3B, IL6R, M6PR, MARK1, MX1, NDUFA2, NDUFS1, NDUFV1, ALDH7A1, PA2G4, UBXN1, ZNF593, GMPR2, POLR3K, PGAM1, PTOV1, PPIC, PPP2R1A, PRR5-ARHGAP8, PREP, NAXD, EXOSC5, RANBP10, PTPRF, RAD23A, HRH4, RBP1, RBP4, RPL13, RPL18, RPS15, RPS19, RPS21, RPS26, RXRB, BLVRB, MRPS11, BMP7, SNRPB, SPRR2A, SREBF2, ACTC1, TFAP2C, TPM3, CA1, CA2, ZNF177, EPHX3, PUS1, ULBP2, TAF15, URM1,

		SYMPK, ARFGAP2, ZNF559, TUBA1C, ITGA8, CST7, PLPP1, IRS2, TNFSF9, SUCLG1, FCGBP, SELENBP1, ZNF160, RRP9, WDR46, TMEM63A, MTX1, THBS3, ASH1L, MEF2C-AS1, LRFN2, DNAH11, MIR3660
**THCA**	NKX2-1, FOXE1, BRAF, HABP2, RET, KRAS, TSHR, TP53, PCNXL2, OBFC1, ILF2P2, PTCSC3, DIRC3, NRG1, KRT18P13, SLK, LINC00609, MBIP, C9orf156	NKX2-1, FOXE1, BRAF, HABP2, RET, KRAS, TSHR, TP53, HRAS, PTEN, NRAS, DICER1, PRKAR1A, MSH6, CXCL8, PPARG, CCND1, SLC5A5, TERT, PIK3CA, NCOA4, MTOR, HIF1A, PTGS2, TPM3, TNF, RAP1GAP, IL6, CCL2, CDKN1B, ENPP2, IL1B, TSC2, CDH1, EPO, EPOR, CXCL10, PRDM2, CSF2, RXRA, BRD4, HPGD, IFNA2, PDGFA, TPR, PTGES2, TCF7L1, PCNXL2, OBFC1, ILF2P2, PTCSC3, SMAD3, DIRC3, EPB41L4A, NRG1, KRT18P13, SLK, LINC00609, MBIP, C9orf156

Table S26. Interactome average intra-disease distance localization measure under the six settings tested in the analysis. The first column of each group of two columns shows the number of genes associated with a particular disorder represented in the interactome under analysis. The second column provides the intra-disease distance values for each list of disease-associated genes and gene variants and their associated p-values.

	Interactome 1 stringent		Interactome 2 stringent		Interactome 3 stringent		Interactome 1 relaxed		Interactome 2 relaxed		Interactome 3 relaxed	
**Disease**	**Nº of genes**	**(p-val)**	**Nº of genes**	**(p-val)**	**Nº of genes**	**(p-val)**	**Nº of genes**	**(p-val)**	**Nº of genes**	**(p-val)**	**Nº of genes**	**(p-val)**
**AD**	55	1.8 (2.35e-07)	57	1.54 (1.02e-04)	55	1.35 (3.86e-09)	138	1.7 (4.50e-04)	142	1.43 (3.88e-06)	137	1.3 (1.31e-15)
**PD**	69	1.7 (1.24e-05)	77	1.69 (1.05e-01)	67	1.34 (4.46e-14)	138	1.72 (4.70e-03)	148	1.57 (4.44e-02)	137	1.24 (5.90e-13)
**ALL**	16	1.56 (4.94e-03)	17	1.71 (5.86e-02)	18	1.39 (8.90e-11)	71	1.39 (1.03e-03)	74	1.31 (5.42e-05)	73	1.3 (4.09e-10)
**AML**	34	1.47 (2.17e-02)	34	1.24 (3.27e-03)	31	1.13 (1.17e-09)	167	1.3 (7.39e-03)	172	1.22 (2.20e-02)	163	1.23 (5.65e-11)
**BLCA**	22	2.05 (9.08e-01)	24	1.42 (3.85e-02)	23	1.48 (1.53e-03)	134	1.54 (2.48e-01)	142	1.3 (5.94e-03)	136	1.31 (1.04e-05)
**BRCA**	126	1.47 (1.47e-02)	135	1.28 (4.91e-03)	125	1.34 (8.60e-05)	1044	1.15 (1.57e-03)	1094	1.16 (6.70e-02)	1029	1.24 (2.01e-03)
**BRNCA**	17	1.82 (1.27e-01)	19	1.26 (6.97e-03)	17	1.41 (4.02e-04)	52	1.69 (1.75e-02)	57	1.28 (4.57e-02)	53	1.3 (7.93e-06)

**CERV**	3	1.67 (2.08e-02)	4	2.5 (7.48e-01)	4	1.75 (3.33e-02)	5	1.8 (2.42e-02)	7	2.71 (9.99e-01)	7	1.57 (2.86e-05)
**CHLCA**	3	2 (2.71e-01)	3	2 (8.45e-01)	3	1 (1.39e-02)	26	1.65 (1.16e-01)	26	1.31 (2.11e-02)	26	1.15 (9.74e-06)
**CLL**	32	1.56 (3.60e-05)	33	1.73 (2.93e-02)	29	1.79 (1.22e-03)	85	1.45 (4.56e-05)	90	1.4 (1.45e-03)	82	1.48 (5.16e-04)
**CML**	2	3 (5.47e-01)	2	2 (8.94e-02)	NA	NA	2	3 (5.49e-01)	2	2 (8.72e-02)	NA	NA
**CRCA**	67	1.54 (2.08e-02)	68	1.18 (1.21e-04)	68	1.26 (1.10e-03)	672	1.27 (3.48e-04)	700	1.25 (6.90e-03)	680	1.36 (2.22e-05)
**DLBCL**	7	2.43 (5.42e-01)	8	1.75 (1.16e-01)	7	1.29 (1.86e-04)	57	1.44 (8.87e-04)	59	1.24 (1.68e-03)	55	1.22 (5.61e-06)
**FLYMPH**	7	2.29 (2.11e-01)	7	2.29 (7.97e-01)	7	1.86 (8.09e-02)	26	2 (8.78e-02)	28	1.82 (3.27e-01)	23	1.3 (2.09e-07)
**HANC**	7	1.86 (5.21e-02)	7	1.57 (1.05e-01)	7	1.71 (1.95e-02)	51	1.39 (3.82e-04)	52	1.25 (3.28e-03)	51	1.24 (3.11e-05)
**KDNCA**	17	1.71 (1.17e-01)	19	1.47 (1.46e-03)	17	1.41 (1.57e-05)	135	1.59 (1.33e-02)	146	1.4 (2.87e-02)	141	1.47 (4.63e-04)
**LGCA**	33	1.64 (8.21e-02)	37	1.38 (8.68e-02)	34	1.35 (1.27e-04)	245	1.36 (1.91e-03)	261	1.27 (3.16e-02)	241	1.24 (3.40e-07)

**LIVCA**	35	1.46 (6.05e-04)	35	1.34 (1.56e-02)	35	1.29 (1.93e-03)	469	1.29 (5.52e-02)	486	1.23 (2.38e-01)	480	1.21 (9.31e-07)
**OVCA**	34	1.56 (3.51e-03)	35	1.17 (2.51e-03)	31	1.23 (2.75e-04)	144	1.38 (5.05e-03)	147	1.19 (3.62e-04)	141	1.3 (7.51e-04)
**PACA**	19	1.63 (8.37e-03)	20	1.55 (1.16e-01)	19	1.26 (6.79e-09)	103	1.54 (4.57e-03)	110	1.32 (6.77e-03)	104	1.18 (8.12e-11)
**PRCA**	75	1.83 (4.89e-02)	79	1.53 (4.77e-02)	73	1.73 (2.41e-05)	654	1.26 (3.45e-02)	679	1.15 (1.57e-03)	649	1.25 (5.69e-07)
**SKCM**	32	1.44 (1.07e-04)	34	1.38 (7.64e-02)	32	1.19 (6.06e-07)	234	1.47 (1.74e-01)	249	1.33 (1.95e-01)	240	1.4 (1.62e-02)
**STCA**	19	1.79 (1.92e-01)	20	1.6 (1.79e-01)	20	1.45 (1.04e-03)	289	1.33 (3.39e-03)	299	1.16 (5.51e-03)	287	1.18 (5.23e-07)
**THCA**	13	1.69 (7.99e-02)	14	1.86 (7.17e-01)	11	1.27 (4.23e-07)	53	1.7 (1.74e-01)	55	1.42 (1.13e-01)	52	1.13 (5.47e-09)

Table S27. Sizes of the largest connected components under the six tested settings. The first column of each group of two columns shows the number of genes associated with a particular disorder represented in the interactome under analysis. The second column provides the size of the largest connected component and its associated p-value.

	**Interactome 1 stringent**		**Interactome 2 stringent**		**Interactome 3 stringent**		**Interactome 1 relaxed**		**Interactome 2 relaxed**		**Interactome 3 relaxed**	
**Disease**	**Nº of genes**	**S (p-val)**	**Nº of genes**	**S (p-val)**	**Nº of genes**	**S (p-val)**	**Nº of genes**	**S (p-val)**	**Nº of genes**	**S (p-val)**	**Nº of genes**	**S (p-val)**

**AD**	55	6 (3.27e-11)	57	28 (7.50e-09)	55	37 (0.00e+00)	138	15 (1.71e-01)	142	75 (6.06e-05)	137	94 (6.36e-13)
**ASD**	19	1 (7.12e-01)	19	4 (8.85e-02)	17	6 (1.98e-12)	93	10 (4.06e-01)	98	17 (2.06e-02)	96	29 (0.00e+00)
**BD**	22	5 (1.41e-02)	24	1 (8.56e-01)	23	12 (0.00e+00)	454	228 (1.37e-02)	465	316 (3.06e-08)	465	358 (9.76e-09)
**HD**	NA	NA	NA	NA	NA	NA	15	1 (6.10e-01)	17	2 (1.23e-01)	17	3 (4.37e-03)
**MD**	23	2 (5.60e-01)	26	3 (3.47e-01)	24	15 (0.00e+00)	432	260 (5.33e-03)	436	317 (5.05e-09)	440	376 (2.01e-11)
**PD**	69	5 (2.58e-01)	77	24 (1.31e-02)	67	40 (0.00e+00)	138	12 (1.10e-01)	148	59 (2.77e-02)	137	101 (1.24e-09)
**SCZ**	38	2 (7.35e-01)	43	5 (1.31e-01)	41	28 (0.00e+00)	888	594 (1.20e-02)	923	684 (3.08e-03)	890	759 (3.11e-14)
**ALL**	16	7 (3.47e-06)	17	3 (8.11e-02)	18	14 (0.00e+00)	71	40 (1.18e-03)	74	51 (5.86e-05)	73	53 (9.54e-13)
**AML**	34	16 (2.18e-02)	34	21 (5.04e-02)	31	22 (8.31e-13)	167	113 (9.89e-03)	172	133 (1.97e-02)	163	121 (2.56e-05)
**BLCA**	22	2 (7.63e-01)	24	14 (2.69e-02)	23	14 (3.15e-09)	134	61 (1.07e-01)	142	99 (4.82e-03)	136	96 (5.68e-04)

**BRCA**	126	61 (2.21e-02)	135	95 (1.04e-02)	125	86 (1.27e-03)	1044	882 (1.53e-03)	1094	922 (2.98e-02)	1029	777 (8.34e-04)
**BRNCA**	17	4 (4.14e-02)	19	14 (8.51e-03)	17	12 (5.59e-10)	52	17 (1.36e-05)	57	42 (1.89e-02)	53	39 (7.80e-08)
**CERV**	3	2 (0.00e+00)	4	1 (5.45e-01)	4	2 (4.69e-04)	5	2 (0.00e+00)	7	1 (5.79e-01)	7	2 (1.36e-03)
**CHLCA**	3	1 (6.03e-01)	3	1 (8.29e-01)	3	3 (6.99e-04)	26	11 (3.46e-03)	26	18 (1.47e-02)	26	22 (6.46e-10)
**CLL**	32	5 (1.89e-01)	33	11 (2.26e-05)	29	9 (2.92e-07)	85	42 (1.31e-03)	90	53 (1.33e-03)	82	41 (2.77e-04)
**CML**	2	1 (5.06e-01)	2	1 (5.17e-01)	NA	NA	2	1 (5.04e-01)	2	1 (5.20e-01)	NA	NA
**CRCA**	67	26 (4.04e-03)	68	56 (1.11e-04)	68	51 (1.47e-03)	672	489 (2.27e-04)	700	518 (7.43e-03)	680	415 (3.31e-04)
**DLBCL**	7	1 (5.30e-01)	8	2 (2.02e-01)	7	3 (2.35e-04)	57	30 (1.44e-03)	59	41 (1.04e-02)	55	45 (1.18e-07)
**FLYMPH**	7	1 (5.46e-01)	7	1 (6.87e-01)	7	2 (2.26e-01)	26	3 (2.71e-03)	28	3 (5.97e-01)	23	6 (5.00e-03)
**HANC**	7	2 (8.07e-04)	7	3 (1.01e-01)	7	2 (8.68e-02)	51	34 (2.80e-06)	52	38 (4.60e-03)	51	40 (4.35e-09)
**KDNCA**	17	5 (2.90e-02)	19	9 (5.55e-05)	17	5 (1.96e-05)	135	42 (2.49e-02)	146	85 (2.04e-02)	141	77 (1.68e-05)

**LGCA**	33	12 (2.95e-02)	37	24 (5.48e-02)	34	24 (8.91e-11)	245	146 (6.02e-03)	261	191 (1.44e-02)	241	184 (1.35e-05)
**LIVCA**	35	15 (6.34e-03)	35	21 (4.41e-02)	35	28 (1.69e-05)	469	329 (4.62e-02)	486	371 (2.28e-01)	480	378 (6.14e-07)
**OVCA**	34	15 (1.29e-04)	35	29 (3.01e-03)	31	24 (8.87e-07)	144	86 (5.07e-03)	147	117 (8.41e-04)	141	97 (4.94e-03)
**PACA**	19	4 (2.17e-01)	20	10 (3.98e-02)	19	13 (0.00e+00)	103	42 (2.09e-02)	110	70 (4.10e-02)	104	86 (1.88e-05)
**PRCA**	75	7 (3.57e-01)	79	38 (1.08e-02)	73	27 (0.00e+00)	654	480 (3.17e-02)	679	574 (2.92e-03)	649	491 (2.71e-09)
**SKCM**	32	14 (1.84e-04)	34	22 (2.65e-02)	32	24 (3.11e-11)	234	121 (1.02e-01)	249	163 (2.15e-01)	240	142 (9.98e-03)
**STCA**	19	3 (4.40e-01)	20	8 (9.28e-02)	20	11 (3.06e-11)	289	185 (4.79e-03)	299	248 (6.99e-03)	287	233 (7.93e-07)
**THCA**	13	4 (8.82e-02)	14	2 (7.93e-01)	11	8 (0.00e+00)	53	7 (6.22e-01)	55	32 (6.99e-02)	52	43 (2.30e-06)

Table S28. Significant interactome-based overlaps identified under all the tested settings. I1S (Interactome 1 stringent disease associated sets of genes and variant genes), I2S (Interactome 2 stringent disease associated sets of genes and variant genes), I3S (Interactome 3 stringent disease associated sets of genes and variant genes), I1R (Interactome 1 relaxed disease associated sets of genes and variant genes), I2R (Interactome 2 relaxed disease associated sets of genes and variant genes), I3R (Interactome 3 stringent relaxed associated sets of genes and variant genes).

Disease A	Disease B	S_{AB}	P-value_form
		**I1S**	
**LGCA**	**OVCA**	-0.54	8.98e-09
**LGCA**	**STCA**	-0.41	3.02e-03
**CRCA**	**LGCA**	-0.28	6.30e-03
**CRCA**	**OVCA**	-0.28	7.00e-03
**BRCA**	**PRCA**	-0.18	8.70e-03
**BRNCA**	**LGCA**	-0.35	2.27e-02
**BRCA**	**LGCA**	-0.17	4.31e-02
		**I2S**	
**LGCA**	**OVCA**	-0.39	1.13e-05
**BRCA**	**LGCA**	-0.13	1.06e-02
**CRCA**	**OVCA**	-0.23	1.40e-02
		**I3S**	
**LGCA**	**OVCA**	-0.4	1.68e-02
		**I1R**	
**LIVCA**	**PRCA**	-0.15	3.36e-07
**LGCA**	**PRCA**	-0.15	9.20e-07
**BRCA**	**CRCA**	-0.15	1.43e-06
**LGCA**	**LIVCA**	-0.16	7.59e-06
**LGCA**	**OVCA**	-0.2	1.18e-05
**LGCA**	**STCA**	-0.18	1.40e-05
**LIVCA**	**STCA**	-0.15	3.07e-05
**BLCA**	**PACA**	-0.22	7.63e-05
**PRCA**	**STCA**	-0.12	1.21e-04
**BLCA**	**LGCA**	-0.16	1.93e-03
**OVCA**	**PACA**	-0.2	3.24e-03
**BRCA**	**PRCA**	-0.12	4.94e-03
**CHLCA**	**THCA**	-0.3	9.00e-03
**SKCM**	**THCA**	-0.11	1.43e-02
**PRCA**	**SKCM**	-0.09	1.99e-02
**ALL**	**AML**	-0.15	4.06e-02
		**I2R**	
**LGCA**	**PRCA**	-0.16	1.73e-14
**BRCA**	**CRCA**	-0.17	3.18e-13
**PRCA**	**STCA**	-0.13	1.79e-09
**LGCA**	**OVCA**	-0.21	3.64e-09
**LGCA**	**LIVCA**	-0.14	3.39e-08
**LGCA**	**STCA**	-0.17	1.00e-07
**BRCA**	**PRCA**	-0.14	2.48e-07
**LIVCA**	**PRCA**	-0.14	5.07e-07
**BRCA**	**LGCA**	-0.06	4.54e-06
**CRCA**	**LGCA**	-0.09	2.38e-05
**BRCA**	**LIVCA**	-0.1	1.13e-04
**LIVCA**	**STCA**	-0.12	3.07e-04
**BLCA**	**LGCA**	-0.14	8.12e-04
**LGCA**	**SKCM**	-0.14	1.47e-03
**CRCA**	**PRCA**	-0.12	2.13e-03
**BLCA**	**LIVCA**	-0.08	3.77e-03
**OVCA**	**PRCA**	-0.03	5.34e-03
**BRCA**	**SKCM**	-0.03	9.11e-03
**OVCA**	**PACA**	-0.15	3.62e-02
**OVCA**	**STCA**	-0.1	3.85e-02

	**I3R**		
**LGCA**	**OVCA**	-0.18	1.98e-05
**PRCA**	**STCA**	-0.09	2.80e-05
**LGCA**	**PRCA**	-0.07	4.33e-04
**BRCA**	**CRCA**	-0.15	8.33e-04
**BRCA**	**PRCA**	-0.13	9.54e-04
**OVCA**	**PRCA**	-0.01	1.87e-03
**LIVCA**	**PRCA**	-0.12	3.66e-03
**LIVCA**	**STCA**	-0.1	1.19e-02

Table S29. Significant genetic correlations between dataset pairs. The first and the second column shows the involved datasets. Column three presents the measured genetic correlation between each pair. Column four shows the standard error of the computed genetic correlations. Finally, columns five and six indicate the z-scores and the raw p-values linked to each association.

A) Significant genetic correlations between pairs of studies targeting the same disorder					
**Disease 1**	**Disease 2**	Rg (Cambiar)	**SE**	**z**	**p**
AD 1	AD 2	1.8	0.33	5.54	3.09e-08
AD 2	AD 3	0.92	0.14	6.4	1.60e-10
PD 1	PD 2	0.86	0.06	13.44	3.51e-41
BRCA 1	BRCA 2	1.02	0	233.42	0.00e+00
BRCA 1	BRCA 3	0.93	0.08	11.89	1.26e-32
BRCA 2	BRCA 3	1	0.06	17.17	4.47e-66
PRCA 2	PRCA 3	1.04	0.05	20.58	4.59e-94
SKCM 1	SKCM 2	1.24	0.28	4.48	7.49e-06
**B) Significant genetic correlations between pairs of CNS disorders**					
**Disease 1**	**Disease 2**	Rg (Cambiar)	**SE**	**z**	**p**
AD 3	PD 1	0.21	0.08	2.51	1.21e-02
**C) Significant genetic correlations between pairs of cancers**					
**Disease 1**	**Disease 2**	Rg (Cambiar)	**SE**	**z**	**p**
BRCA 2	OVCA 1	0.23	0.06	3.68	2.00e-04
BRCA 2	SKCM 2	0.12	0.04	2.68	7.40e-03
BRCA 3	CRCA 1	-0.22	0.13	-1.74	8.11e-02
PRCA 2	BRCA 1	0.11	0.05	2.32	2.01e-02
PRCA 2	BRCA 2	0.07	0.03	2.41	1.59e-02
PRCA 3	BRCA 2	0.11	0.04	2.56	1.05e-02
**D) Significant genetic correlations between CNS disorders and cancers**					
**Disease 1**	**Disease 2**	Rg (Cambiar)	**SE**	**z**	**p**
PD 1	PRCA 2	0.09	0.04	2.15	3.16e-02
PD 1	SKCM 2	0.14	0.07	2.01	4.41e-02
PD 2	PRCA 3	0.16	0.08	2.01	4.44e-02

Table S30. Drugs indicated for the treatment of AD, PD and the included cancer types with available LINCS L1000 gene expression signatures.

Disorder	DB_ID	LINCS perturbation ID	name
AD	DB00674	BRD-K49481516	Galantamine
AD	DB01043	BRD-A79803969	Memantine
AD	DB01037	BRD-K86434416	Selegiline
AD	DB00382	BRD-K81473089	Tacrine
AD	DB00313	BRD-K41260949	Valproic acid
AD	DB00163		Vitamin E
AD	DB14003		alpha-Tocopherol acetate
AD	DB00843	BRD-A49160188	Donepezil
AD	DB00989	BRD-K10706131	Rivastigmine
AD	DB14477		DL-alpha tocopheryl acetate
PD	DB00915	BRD-K70330367	Amantadine
PD	DB00714	BRD-K76022557	Apomorphine
PD	DB00245		Benzatropine
PD	DB00810	BRD-A00546892	Biperiden
PD	DB01200		Bromocriptine
PD	DB00190	BRD-K78712176	Carbidopa
PD	DB01235	BRD-K34730807	Levodopa
PD	DB01186	BRD-K60770992	Pergolide
PD	DB01037	BRD-K86434416	Selegiline
PD	DB00376	BRD-A48180038	Trihexyphenidyl
PD	DB00248	BRD-K86882815	Cabergoline
PD	DB00494	BRD-K83636919	Entacapone
PD	DB00268	BRD-K15933101	Ropinirole
PD	DB00323	BRD-K10852020	Tolcapone
PD	DB01367	BRD-K58114536	Rasagiline
PD	DB00424		Hyoscyamine
PD	DB00989	BRD-K10706131	Rivastigmine
PD	DB05271	BRD-K91111634	Rotigotine
PD	DB00413	BRD-K06388322	Pramipexole
ALL	DB01033	BRD-K91601245	Mercaptopurine
ALL	DB00023		Asparaginase Escherichia coli
ALL	DB00987	BRD-K33106058	Cytarabine
ALL	DB00694	BRD-K43389675	Daunorubicin
ALL	DB00997	BRD-K92093830	Doxorubicin
ALL	DB00444		Teniposide
ALL	DB00059		Pegaspargase
ALL	DB00631	BRD-K34022604	Clofarabine
ALL	DB00619	BRD-K92723993	Imatinib
AML	DB00023		Asparaginase Escherichia coli
AML	DB00987	BRD-K33106058	Cytarabine
AML	DB00694	BRD-K43389675	Daunorubicin
AML	DB00997	BRD-K92093830	Doxorubicin
AML	DB00773	BRD-K37798499	Etoposide
AML	DB01177	BRD-K69650333	Idarubicin
AML	DB01204	BRD-K21680192	Mitoxantrone
AML	DB00352	BRD-K49350383	Tioguanine
AML	DB00755	BRD-K71879491	Tretinoin
AML	DB01169		Arsenic trioxide
AML	DB00020		Sargramostim
AML	DB00056		Gemtuzumab ozogamicin
BLCA	DB00305		Mitomycin
BLCA	DB00515		Cisplatin
BLCA	DB00997	BRD-K92093830	Doxorubicin
BLCA	DB00773	BRD-K37798499	Etoposide
BLCA	DB00544	BRD-K24844714	Fluorouracil
BLCA	DB04572	BRD-K09631521	Thiotepa
BLCA	DB00441	BRD-K15108141	Gemcitabine

BLCA	DB00385	BRD-K72951360	Valrubicin
BLCA	DB00958		Carboplatin
BLCA	DB10804		Bacillus calmette-guerin substrain connaught live antigen
BLCA	DB10343		Bacillus calmette-guerin substrain tice live antigen
BRCA	DB00445	BRD-K04548931	Epirubicin
BRCA	DB00783	BRD-K18910433	Estradiol
BRCA	DB00286		Conjugated estrogens
BRCA	DB00544	BRD-K24844714	Fluorouracil
BRCA	DB01185		Fluoxymesterone
BRCA	DB01181	BRD-A67097164	Ifosfamide
BRCA	DB00563	BRD-K59456551	Methotrexate
BRCA	DB06710	BRD-K84036904	Methyltestosterone
BRCA	DB00675	BRD-K93754473	Tamoxifen
BRCA	DB00894		Testolactone
BRCA	DB00624		Testosterone
BRCA	DB04572	BRD-K09631521	Thiotepa
BRCA	DB00282		Pamidronic acid
BRCA	DB00441	BRD-K15108141	Gemcitabine
BRCA	DB00351	BRD-K19507340	Megestrol acetate
BRCA	DB00539	BRD-K51350053	Toremifene
BRCA	DB00361		Vinorelbine
BRCA	DB00380	BRD-K07265709	Dexrazoxane
BRCA	DB00014	BRD-K99504665	Goserelin
BRCA	DB01229		Paclitaxel
BRCA	DB00481	BRD-K63828191	Raloxifene
BRCA	DB01006	BRD-K88789588	Letrozole
BRCA	DB01217	BRD-K52172416	Anastrozole
BRCA	DB01101	BRD-K61192372	Capecitabine
BRCA	DB09381		Esterified estrogens
BRCA	DB00072		Trastuzumab
BRCA	DB00112		Bevacizumab
BRCA	DB00990	BRD-K33425534	Exemestane
BRCA	DB00947		Fulvestrant
BRCA	DB04845	BRD-K03601870	Ixabepilone
BRCA	DB01259	BRD-M07438658	Lapatinib
BRCA	DB01259	BRD-K19687926	Lapatinib
BRCA	DB08871		Eribulin
BRNCA	DB00262		Carmustine
BRNCA	DB00515		Cisplatin
BRNCA	DB00853	BRD-K32107296	Temozolomide
CERV	DB00515		Cisplatin
CERV	DB01181	BRD-A67097164	Ifosfamide
CERV	DB01030	BRD-K55696337	Topotecan
CLL	DB01033	BRD-K91601245	Mercaptopurine
CLL	DB00291	BRD-K29458283	Chlorambucil
CLL	DB00773	BRD-K37798499	Etoposide
CLL	DB01181	BRD-A67097164	Ifosfamide
CLL	DB00888		Mechlorethamine
CLL	DB00552		Pentostatin
CLL	DB01073	BRD-K66788707	Fludarabine
CLL	DB00059		Pegaspargase
CLL	DB00242	BRD-K93034159	Cladribine
CLL	DB00087		Alemtuzumab
CLL	DB06769	BRD-K17068645	Bendamustine
CLL	DB06650		Ofatumumab
CML	DB01008	BRD-K23204545	Busulfan
CML	DB00987	BRD-K33106058	Cytarabine
CML	DB01005	BRD-K51747290	Hydroxyurea
CML	DB01177	BRD-K69650333	Idarubicin

CML	DB00105		Interferon alfa-2b
CML	DB06810		Plicamycin
CML	DB00352	BRD-K49350383	Tioguanine
CML	DB00619	BRD-K92723993	Imatinib
CRCA	DB00544	BRD-K24844714	Fluorouracil
CRCA	DB00526		Oxaliplatin
CRCA	DB00762	BRD-K08547377	Irinotecan
CRCA	DB01101	BRD-K61192372	Capecitabine
CRCA	DB00112		Bevacizumab
CRCA	DB00002		Cetuximab
HANC	DB00290		Bleomycin
HANC	DB00515		Cisplatin
HANC	DB00570	BRD-K01188359	Vinblastine
HANC	DB00958		Carboplatin
HANC	DB01229		Paclitaxel
KDNCA	DB00970	BRD-K70578146	Dactinomycin
KDNCA	DB01005	BRD-K51747290	Hydroxyurea
KDNCA	DB00034		Interferon alfa-2a, Recombinant
KDNCA	DB00105		Interferon alfa-2b
KDNCA	DB00033		Interferon gamma-1b
KDNCA	DB01041	BRD-A93255169	Thalidomide
KDNCA	DB00541	BRD-K82109576	Vincristine
KDNCA	DB00069		Interferon alfacon-1
KDNCA	DB00041		Aldesleukin
KDNCA	DB00008		Peginterferon alfa-2a
KDNCA	DB01590	BRD-K13514097	Everolimus
KDNCA	DB00112		Bevacizumab
KDNCA	DB00022		Peginterferon alfa-2b
KDNCA	DB01268	BRD-M64432851	Sunitinib
KDNCA	DB01268	BRD-K42828737	Sunitinib
KDNCA	DB00398	BRD-K23984367	Sorafenib
KDNCA	DB06287		Temsirolimus
KDNCA	DB06589	BRD-K74514084	Pazopanib
KDNCA	DB00603	BRD-K82216340	Medroxyprogesterone acetate
LGCA	DB00515		Cisplatin
LGCA	DB00445	BRD-K04548931	Epirubicin
LGCA	DB00773	BRD-K37798499	Etoposide
LGCA	DB01005	BRD-K51747290	Hydroxyurea
LGCA	DB01181	BRD-A67097164	Ifosfamide
LGCA	DB00563	BRD-K59456551	Methotrexate
LGCA	DB01168	BRD-K13032584	Procarbazine
LGCA	DB00570	BRD-K01188359	Vinblastine
LGCA	DB00441	BRD-K15108141	Gemcitabine
LGCA	DB00361		Vinorelbine
LGCA	DB00958		Carboplatin
LGCA	DB00762	BRD-K08547377	Irinotecan
LGCA	DB01229		Paclitaxel
LGCA	DB01030	BRD-K55696337	Topotecan
LGCA	DB00642		Pemetrexed
LGCA	DB00112		Bevacizumab
LGCA	DB00317	BRD-K64052750	Gefitinib
LGCA	DB00530	BRD-K70401845	Erlotinib
LGCA	DB08865	BRD-K78431006	Crizotinib
LIVCA	DB00034		Interferon alfa-2a, Recombinant
LIVCA	DB00105		Interferon alfa-2b
LIVCA	DB00033		Interferon gamma-1b
LIVCA	DB00069		Interferon alfacon-1
LIVCA	DB00008		Peginterferon alfa-2a
LIVCA	DB00022		Peginterferon alfa-2b
LIVCA	DB00398	BRD-K23984367	Sorafenib
OVCA	DB00515		Cisplatin

OVCA	DB01005	BRD-K51747290	Hydroxyurea
PACA	DB00997	BRD-K92093830	Doxorubicin
PACA	DB00544	BRD-K24844714	Fluorouracil
PACA	DB00441	BRD-K15108141	Gemcitabine
PACA	DB00530	BRD-K70401845	Erlotinib
PRCA	DB00513	BRD-K64931368	Aminocaproic acid
PRCA	DB00997	BRD-K92093830	Doxorubicin
PRCA	DB00783	BRD-K18910433	Estradiol
PRCA	DB01196		Estramustine
PRCA	DB00286		Conjugated estrogens
PRCA	DB00499	BRD-K28307902	Flutamide
PRCA	DB01026	BRD-A76019558	Ketoconazole
PRCA	DB01204	BRD-K21680192	Mitoxantrone
PRCA	DB01041	BRD-A93255169	Thalidomide
PRCA	DB00351	BRD-K19507340	Megestrol acetate
PRCA	DB00665	BRD-K23566484	Nilutamide
PRCA	DB06825	BRD-K62685538	Triptorelin
PRCA	DB00007		Leuprolide
PRCA	DB00014	BRD-K99504665	Goserelin
PRCA	DB06788		Histrelin
PRCA	DB01128	BRD-A29485665	Bicalutamide
PRCA	DB09381		Esterified estrogens
PRCA	DB00106		Abarelix
PRCA	DB06699		Degarelix
PRCA	DB06643		Denosumab
PRCA	DB06772		Cabazitaxel
PRCA	DB06688		Sipuleucel-T
PRCA	DB05812		Abiraterone
SKCM	DB00290		Bleomycin
SKCM	DB00262		Carmustine
SKCM	DB00851		Dacarbazine
SKCM	DB00970	BRD-K70578146	Dactinomycin
SKCM	DB01005	BRD-K51747290	Hydroxyurea
SKCM	DB00034		Interferon alfa-2a, Recombinant
SKCM	DB00105		Interferon alfa-2b
SKCM	DB00033		Interferon gamma-1b
SKCM	DB01206		Lomustine
SKCM	DB01042		Melphalan
SKCM	DB01168	BRD-K13032584	Procarbazine
SKCM	DB00853	BRD-K32107296	Temozolomide
SKCM	DB00069		Interferon alfacon-1
SKCM	DB00041		Aldesleukin
SKCM	DB00008		Peginterferon alfa-2a
SKCM	DB00022		Peginterferon alfa-2b
SKCM	DB06186		Ipilimumab
SKCM	DB08881	BRD-K56343971	Vemurafenib
STCA	DB00305		Mitomycin
STCA	DB00445	BRD-K04548931	Epirubicin
STCA	DB00544	BRD-K24844714	Fluorouracil
STCA	DB01101	BRD-K61192372	Capecitabine
THCA	DB05294	BRD-K77625799	Vandetanib
LYMPH	DB01033	BRD-K91601245	Mercaptopurine
LYMPH	DB00023		Asparaginase Escherichia coli
LYMPH	DB00290		Bleomycin
LYMPH	DB00262		Carmustine
LYMPH	DB00291	BRD-K29458283	Chlorambucil
LYMPH	DB00515		Cisplatin
LYMPH	DB00987	BRD-K33106058	Cytarabine
LYMPH	DB00851		Dacarbazine
LYMPH	DB00997	BRD-K92093830	Doxorubicin
LYMPH	DB00445	BRD-K04548931	Epirubicin

LYMPH	DB00773	BRD-K37798499	Etoposide
LYMPH	DB01181	BRD-A67097164	Ifosfamide
LYMPH	DB00105		Interferon alfa-2b
LYMPH	DB01206		Lomustine
LYMPH	DB00888		Mechlorethamine
LYMPH	DB00563	BRD-K59456551	Methotrexate
LYMPH	DB01204	BRD-K21680192	Mitoxantrone
LYMPH	DB00860		Prednisolone
LYMPH	DB00635		Prednisone
LYMPH	DB01168	BRD-K13032584	Procarbazine
LYMPH	DB00428	BRD-K34411947	Streptozocin
LYMPH	DB04572	BRD-K09631521	Thiotepa
LYMPH	DB00570	BRD-K01188359	Vinblastine
LYMPH	DB00541	BRD-K82109576	Vincristine
LYMPH	DB00441	BRD-K15108141	Gemcitabine
LYMPH	DB01073	BRD-K66788707	Fludarabine
LYMPH	DB00059		Pegaspargase
LYMPH	DB00361		Vinorelbine
LYMPH	DB00242	BRD-K93034159	Cladribine
LYMPH	DB00762	BRD-K08547377	Irinotecan
LYMPH	DB00020		Sargramostim
LYMPH	DB00041		Aldesleukin
LYMPH	DB00087		Alemtuzumab
LYMPH	DB00073		Rituximab
LYMPH	DB06769	BRD-K17068645	Bendamustine
LYMPH	DB02546	BRD-K81418486	Vorinostat
LYMPH	DB00004		Denileukin diftitox
LYMPH	DB00307	BRD-K92441787	Bexarotene
LYMPH	DB00081		Tositumomab
LYMPH	DB01280	BRD-K84466663	Nelarabine
LYMPH	DB00049		Rasburicase
LYMPH	DB00188	BRD-K88510285	Bortezomib
LYMPH	DB06813	BRD-A74914197	Pralatrexate
LYMPH	DB06809	BRD-K33240821	Plerixafor
LYMPH	DB06176	BRD-K61397605	Romidepsin
LYMPH	DB08870		Brentuximab vedotin

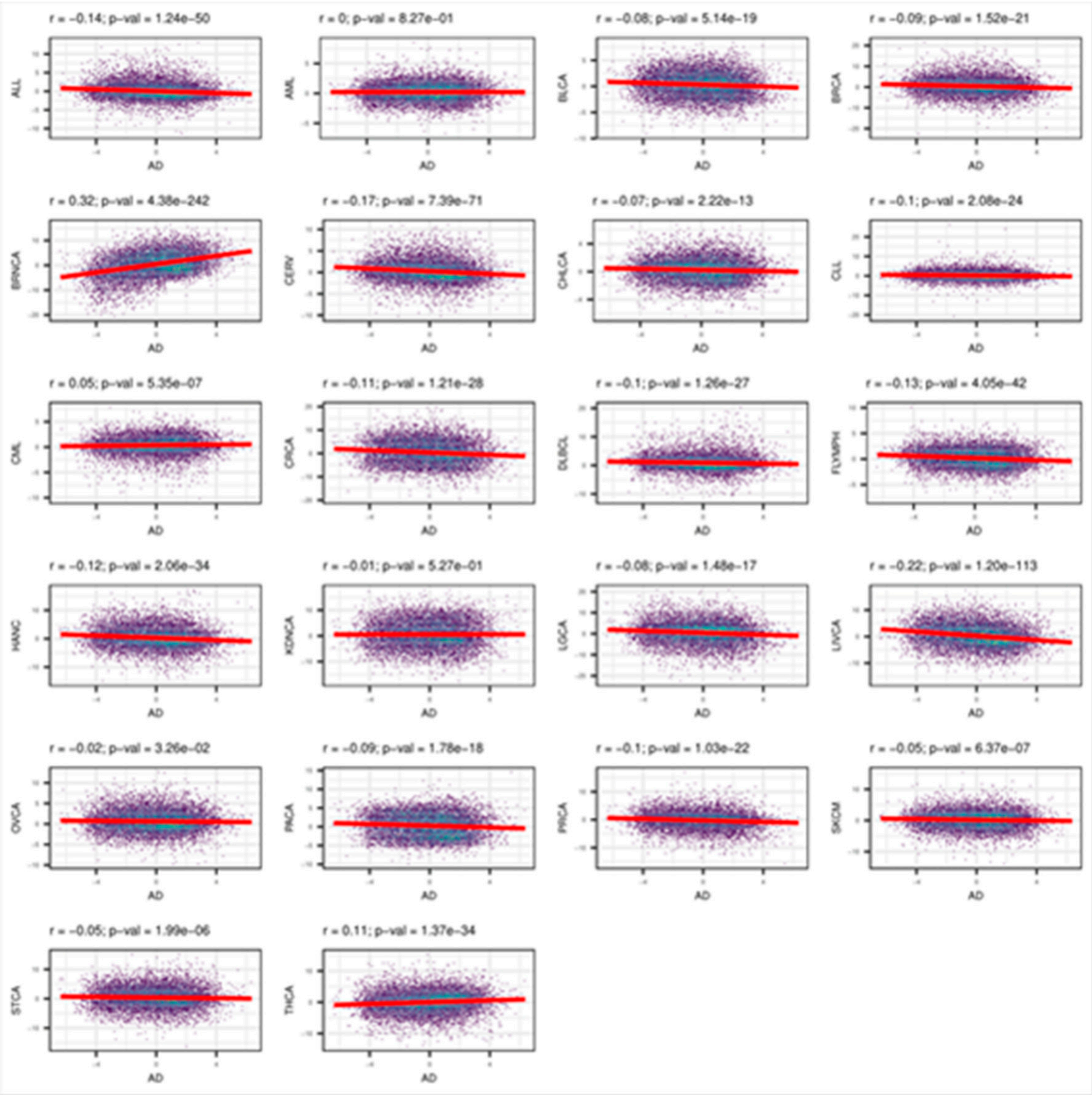

Figure S1. Pearson's correlations of the $\hat{\mu}$ values derived from the differential expression profiles of AD and all the included cancers.

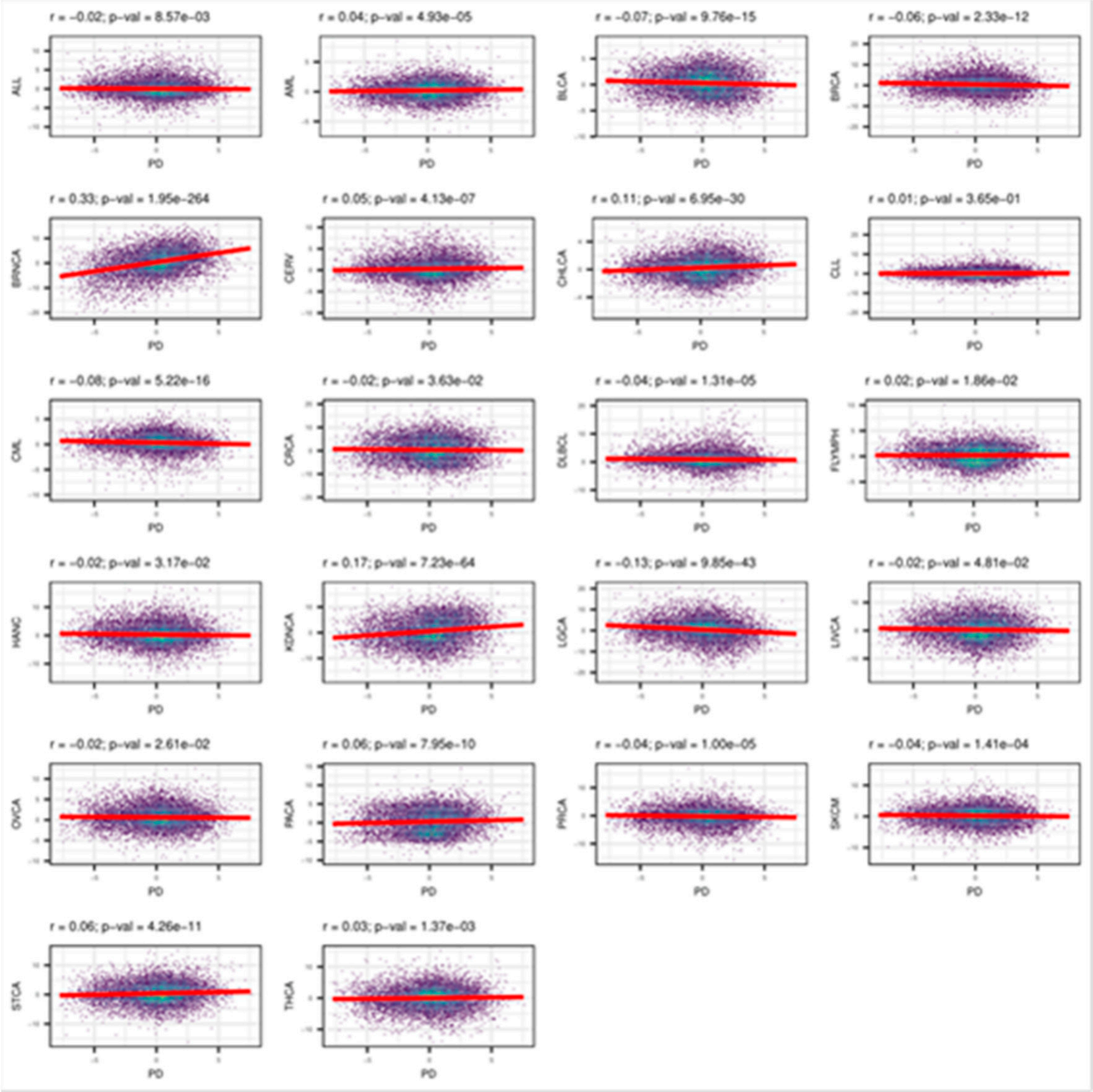

Figure S2. Pearson's correlations of the $\hat{\mu}$ values derived from the differential expression profiles of PD and all the included cancers.

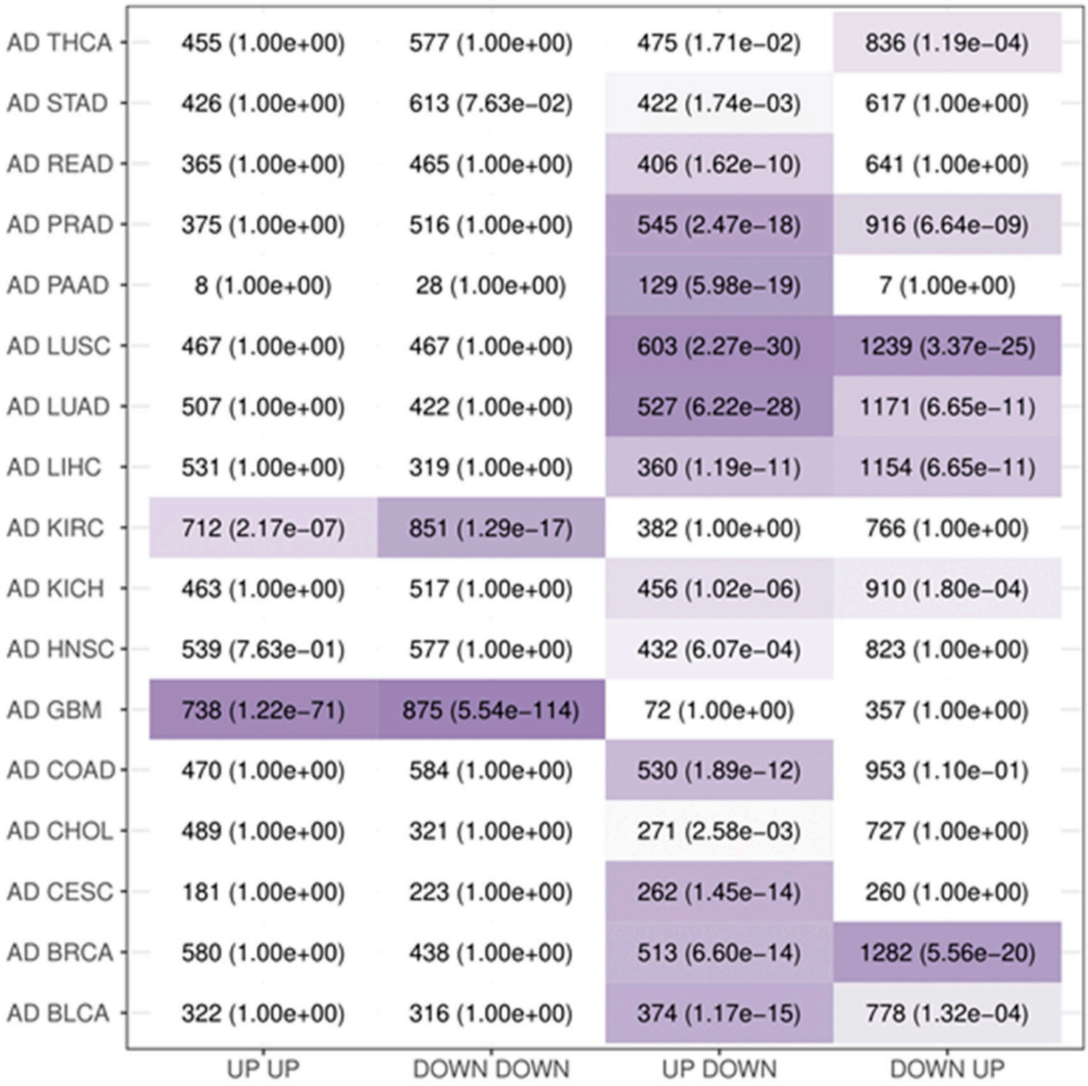

Figure S3. Intersection analysis results for AD and the TCGA-derived cancer data. Bladder Urothelial Carcinoma (BLCA), Breast Invasive Carcinoma (BRCA), Glioblastoma Multiforme (GBM), Cervical Squamous Cell Carcinoma and Endocervical Adenocarcinoma (CESC), Cholangiocarcinoma (CHOL), Colon Adenocarcinoma (COAD), Rectum Adenocarcinoma (READ), Head and Neck Squamous Cell Carcinoma (HNSC), Kidney Chromophobe (KICH), Kidney Renal Clear Cell Carcinoma (KIRC), Lung Adenocarcinoma (LUAD), Lung Squamous Cell Carcinoma (LUSC), Liver Hepatocellular Carcinoma (LIHC), Pancreatic Adenocarcinoma (PAAD), Prostate Adenocarcinoma (PRAD), Stomach Adenocarcinoma (STAD), Thyroid Carcinoma (THCA).

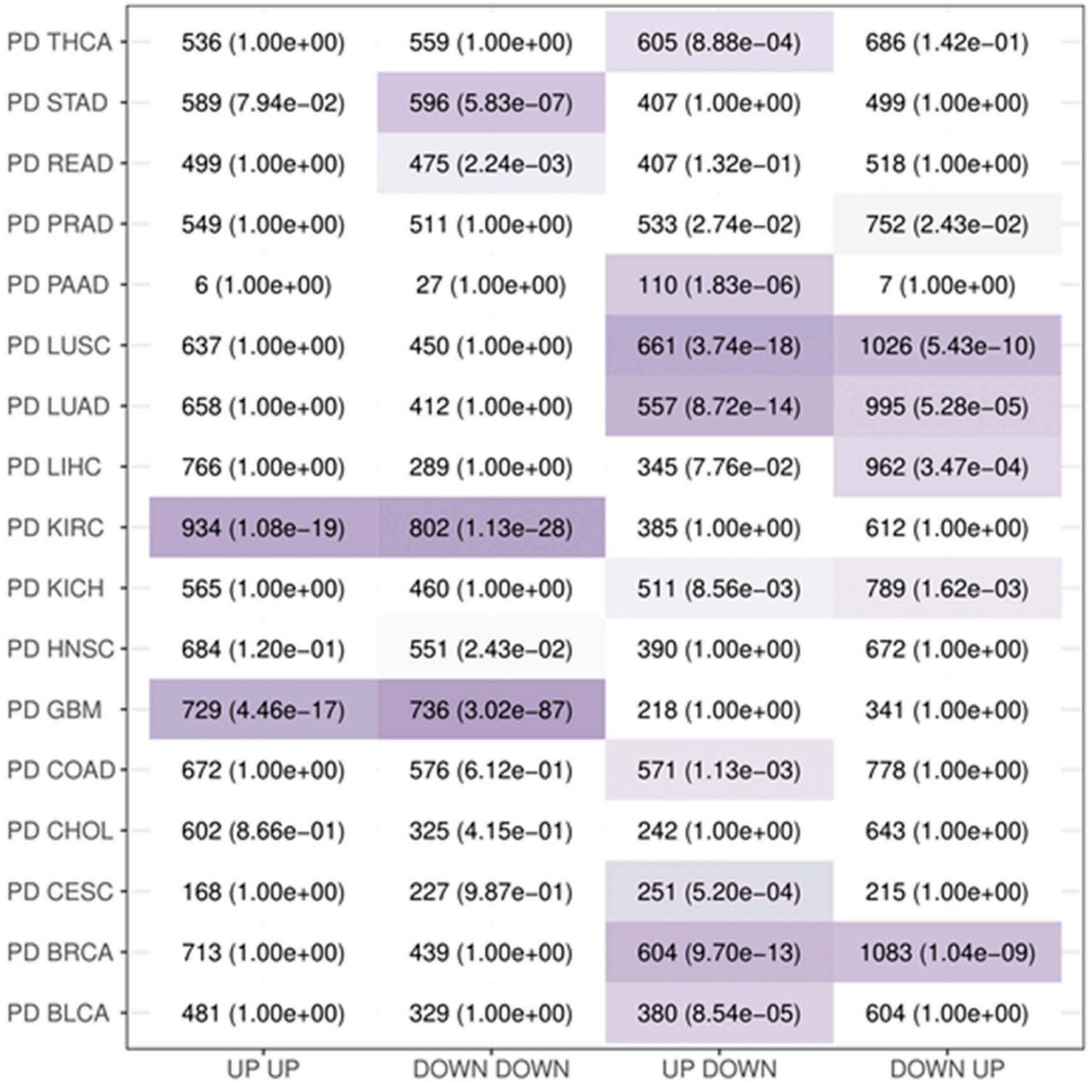

Figure S4. Intersection analysis results for PD and the TCGA-derived cancer data. Bladder Urothelial Carcinoma (BLCA), Breast Invasive Carcinoma (BRCA), Glioblastoma Multiforme (GBM), Cervical Squamous Cell Carcinoma and Endocervical Adenocarcinoma (CESC), Cholangiocarcinoma (CHOL), Colon Adenocarcinoma (COAD), Rectum Adenocarcinoma (READ), Head and Neck Squamous Cell Carcinoma (HNSC), Kidney Chromophobe (KICH), Kidney Renal Clear Cell Carcinoma (KIRC), Lung Adenocarcinoma (LUAD), Lung Squamous Cell Carcinoma (LUSC), Liver Hepatocellular Carcinoma (LIHC), Pancreatic Adenocarcinoma (PAAD), Prostate Adenocarcinoma (PRAD), Stomach Adenocarcinoma (STAD), Thyroid Carcinoma (THCA).

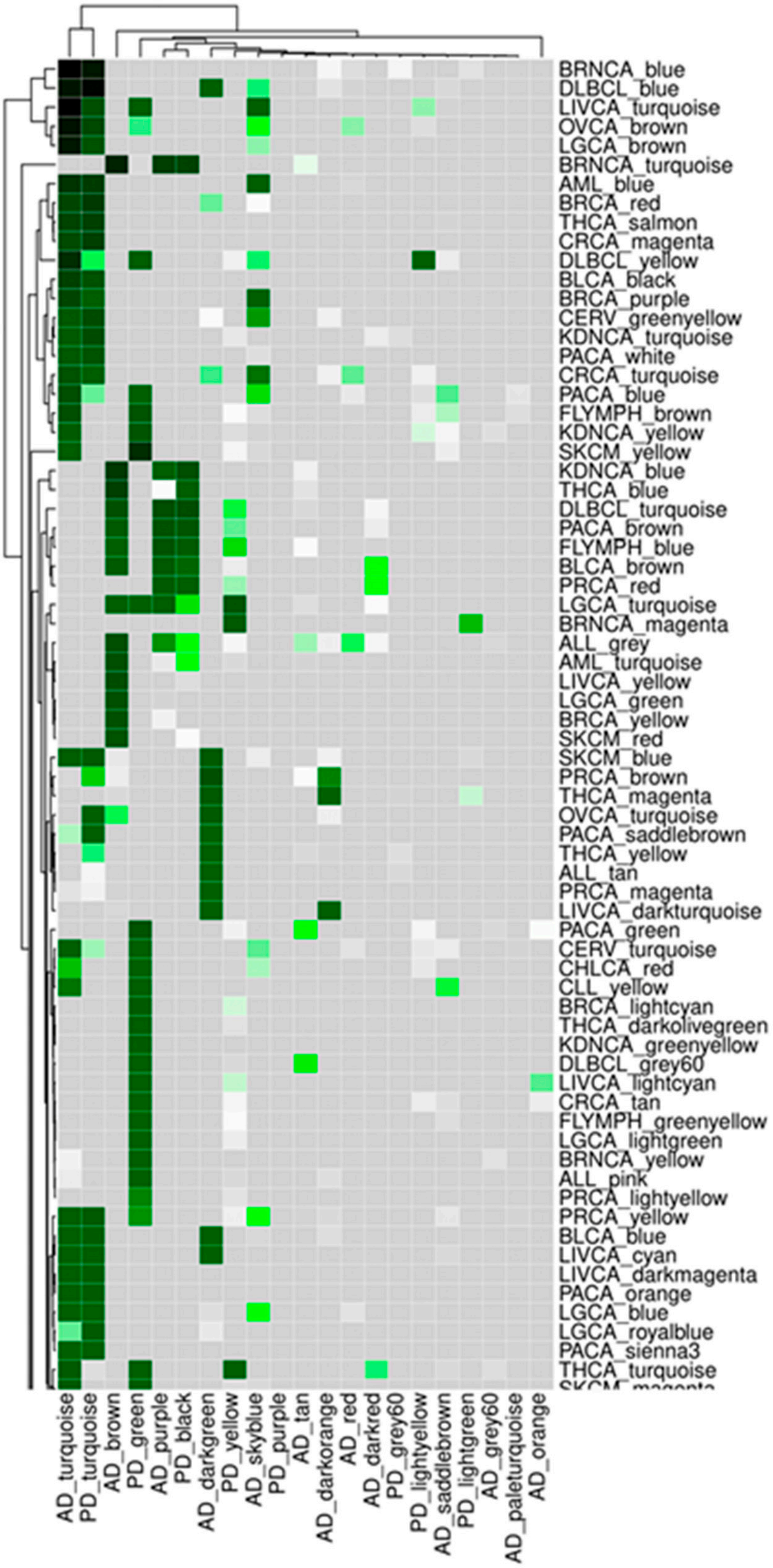
BRNCA_blue
DLBCL_blue
LIVCA_turquoise
OVCA_brown
LGCA_brown
BRNCA_turquoise
AML_blue
BRCA_red
THCA_salmon
CRCA_magenta
DLBCL_yellow
BLCA_black
BRCA_purple
CERV_greenyellow
KDNCA_turquoise
PACA_white
CRCA_turquoise
PACA_blue
FLYMPH_brown
KDNCA_yellow
SKCM_yellow
KDNCA_blue
THCA_blue
DLBCL_turquoise
PACA_brown
FLYMPH_blue
BLCA_brown
PRCA_red
LGCA_turquoise
BRNCA_magenta
ALL_grey
AML_turquoise
LIVCA_yellow
LGCA_green
BRCA_yellow
SKCM_red
SKCM_blue
PRCA_brown
THCA_magenta
OVCA_turquoise
PACA_saddlebrown
THCA_yellow
ALL_tan
PRCA_magenta
LIVCA_darkturquoise
PACA_green
CERV_turquoise
CHLCA_red
CLL_yellow
BRCA_lightcyan
THCA_darkolivegreen
KDNCA_greenyellow
DLBCL_grey60
LIVCA_lightcyan
CRCA_tan
FLYMPH_greenyellow
LGCA_lightgreen
BRNCA_yellow
ALL_pink
PRCA_lightyellow
PRCA_yellow
BLCA_blue
LIVCA_cyan
LIVCA_darkmagenta
PACA_orange
LGCA_blue
LGCA_royalblue
PACA_sienna3
THCA_turquoise
AD_turquoise
PD_turquoise
AD_brown
PD_green
AD_purple
PD_black
AD_darkgreen
PD_yellow
AD_skyblue
PD_purple
AD_tan
AD_darkorange
AD_red
AD_darkred
PD_grey60
PD_lightyellow
AD_saddlebrown
PD_lightgreen
AD_grey60
AD_paleturquoise
AD_orange

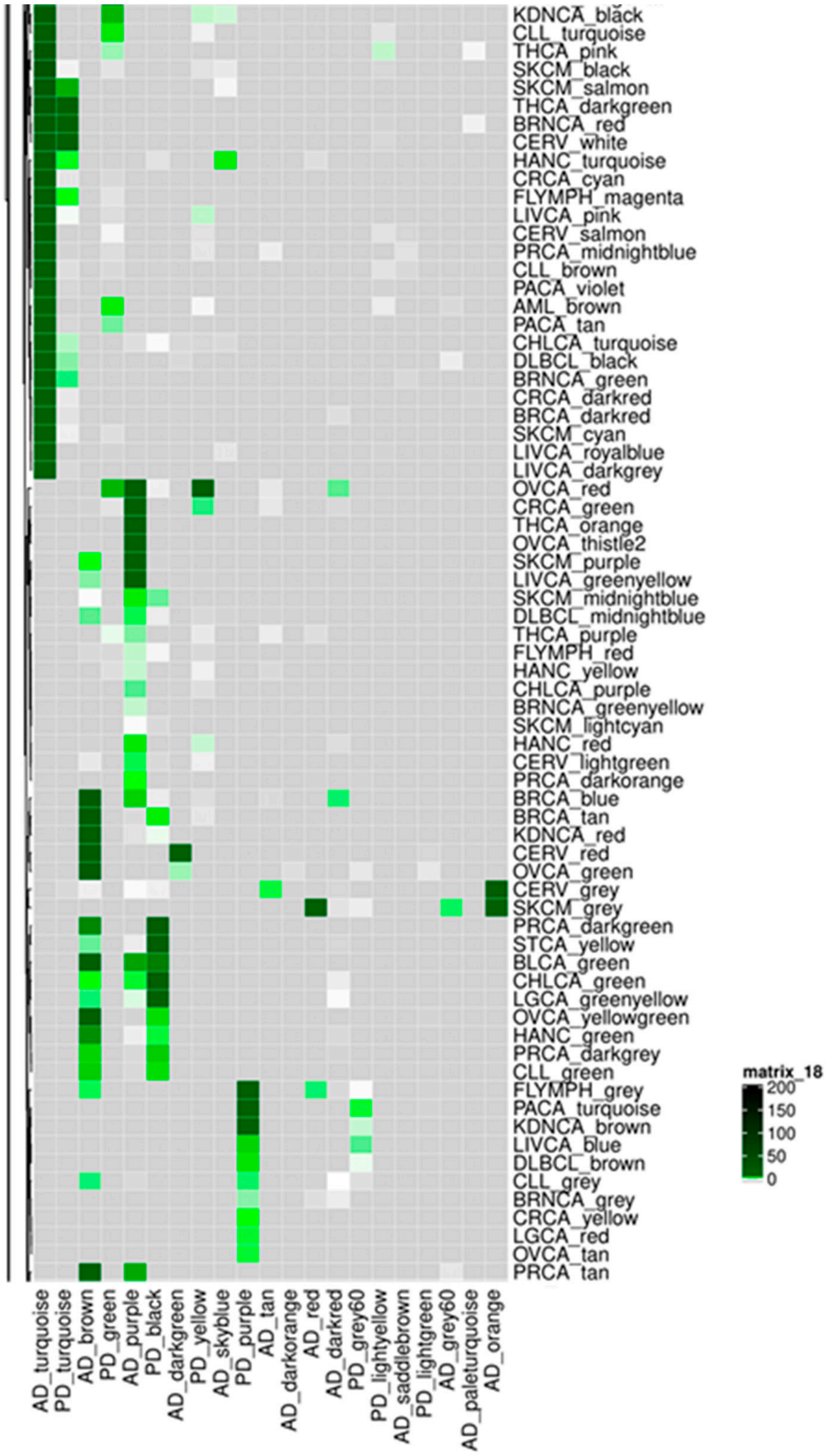
KDNCA_black
CLL_turquoise
THCA_pink
SKCM_black
SKCM_salmon
THCA_darkgreen
BRNCA_red
CERV_white
HANC_turquoise
CRCA_cyan
FLYMPH_magenta
LIVCA_pink
CERV_salmon
PRCA_midnightblue
CLL_brown
PACA_violet
AML_brown
PACA_tan
CHLCA_turquoise
DLBCL_black
BRNCA_green
CRCA_darkred
BRCA_darkred
SKCM_cyan
LIVCA_royalblue
LIVCA_darkgrey
OVCA_red
CRCA_green
THCA_orange
OVCA_thistle2
SKCM_purple
LIVCA_greenyellow
SKCM_midnightblue
DLBCL_midnightblue
THCA_purple
FLYMPH_red
HANC_yellow
CHLCA_purple
BRNCA_greenyellow
SKCM_lightcyan
HANC_red
CERV_lightgreen
PRCA_darkorange
BRCA_blue
BRCA_tan
KDNCA_red
CERV_red
OVCA_green
CERV_grey
SKCM_grey
PRCA_darkgreen
STCA_yellow
BLCA_green
CHLCA_green
LGCA_greenyellow
OVCA_yellowgreen
HANC_green
PRCA_darkgrey
CLL_green
FLYMPH_grey
PACA_turquoise
KDNCA_brown
LIVCA_blue
DLBCL_brown
CLL_grey
BRNCA_grey
CRCA_yellow
LGCA_red
OVCA_tan
PRCA_tan
AD_turquoise
PD_turquoise
AD_brown
PD_green
AD_purple
PD_black
AD_darkgreen
PD_yellow
AD_skyblue
PD_purple
AD_tan
AD_darkorange
AD_red
AD_darkred
PD_grey60
PD_lightyellow
AD_saddlebrown
PD_lightgreen
AD_grey60
AD_paleturquoise
AD_orange
matrix_18
200
150
100
50
0

31

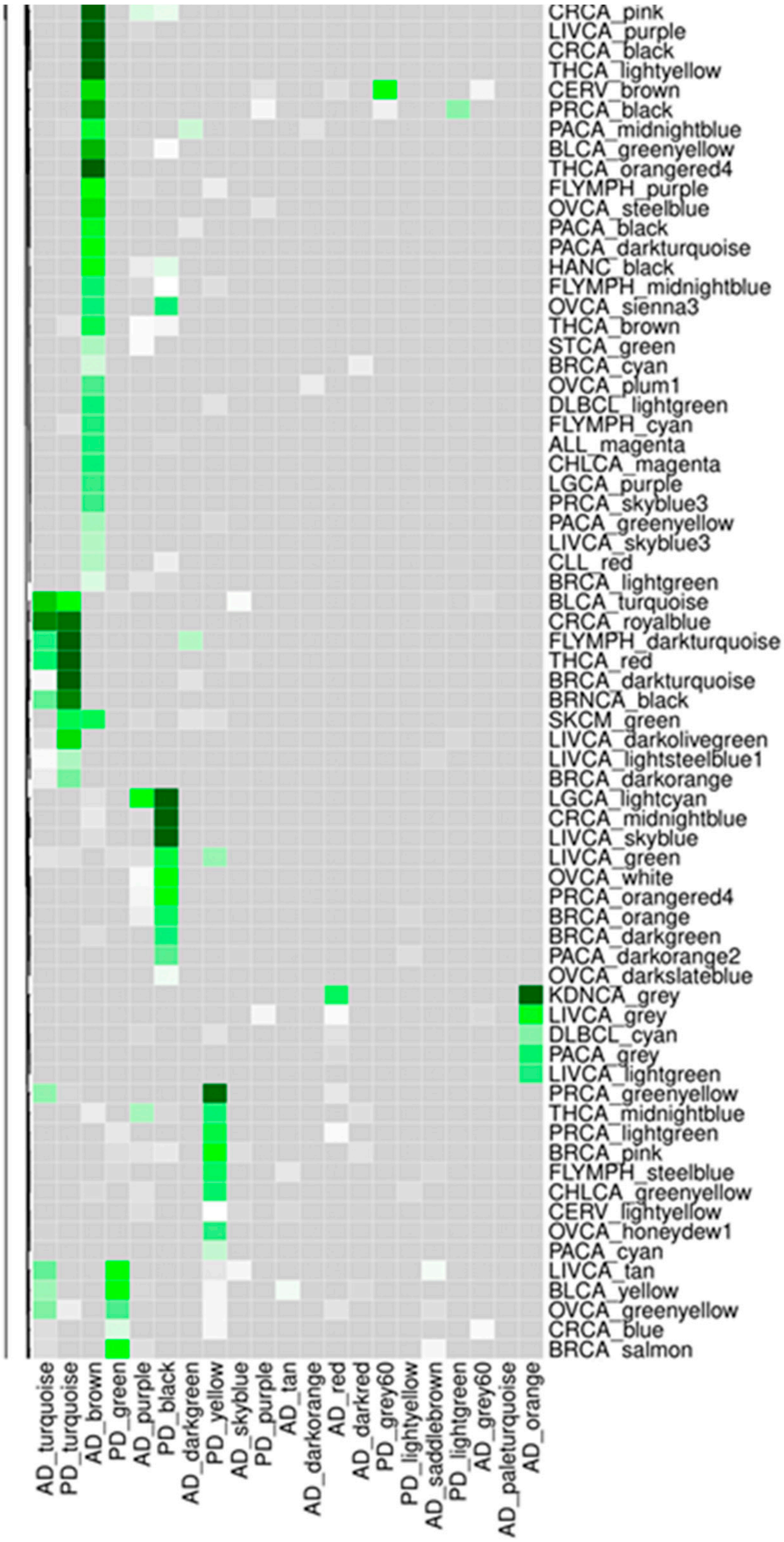
CRCA_pink
LIVCA_purple
CRCA_black
THCA_lightyellow
CERV_brown
PRCA_black
PACA_midnightblue
BLCA_greenyellow
THCA_orangered4
FLYMPH_purple
OVCA_steelblue
PACA_black
PACA_darkturquoise
HANC_black
FLYMPH_midnightblue
OVCA_sienna3
THCA_brown
STCA_green
BRCA_cyan
OVCA_plum1
DLBCL_lightgreen
FLYMPH_cyan
ALL_magenta
CHLCA_magenta
LGCA_purple
PRCA_skyblue3
PACA_greenyellow
LIVCA_skyblue3
CLL_red
BRCA_lightgreen
BLCA_turquoise
CRCA_royalblue
FLYMPH_darkturquoise
THCA_red
BRCA_darkturquoise
BRNCA_black
SKCM_green
LIVCA_darkolivegreen
LIVCA_lightsteelblue1
BRCA_darkorange
LGCA_lightcyan
CRCA_midnightblue
LIVCA_skyblue
LIVCA_green
OVCA_white
PRCA_orangered4
BRCA_orange
BRCA_darkgreen
PACA_darkorange2
OVCA_darkslateblue
KDNCA_grey
LIVCA_grey
DLBCL_cyan
PACA_grey
LIVCA_lightgreen
PRCA_greenyellow
THCA_midnightblue
PRCA_lightgreen
BRCA_pink
FLYMPH_steelblue
CHLCA_greenyellow
CERV_lightyellow
OVCA_honeydew1
PACA_cyan
LIVCA_tan
BLCA_yellow
OVCA_greenyellow
CRCA_blue
BRCA_salmon
AD_turquoise
PD_turquoise
AD_brown
PD_green
AD_purple
PD_black
AD_darkgreen
PD_yellow
AD_skyblue
PD_purple
AD_tan
AD_darkorange
AD_red
AD_darkred
PD_grey60
PD_lightyellow
AD_saddlebrown
PD_lightgreen
AD_grey60
AD_paleturquoise
AD_orange

32

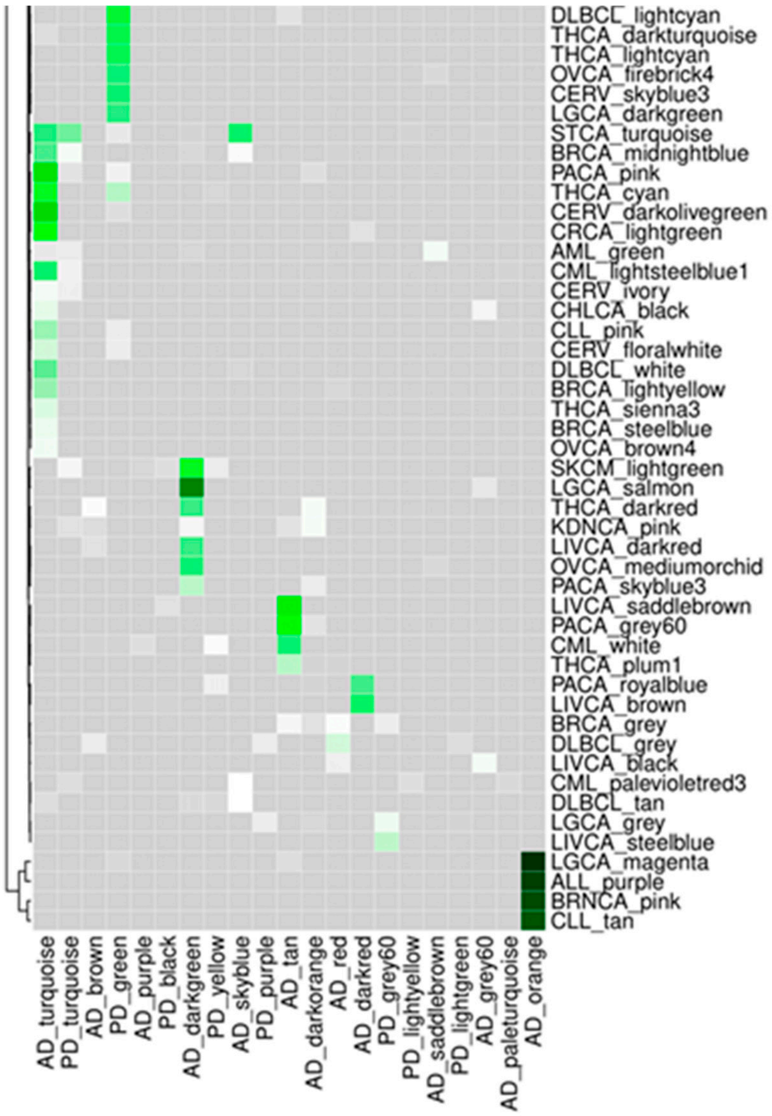

Figure S5. Consensus co-expression module overlap analyses results. Grey cell represent non-significant overlaps whereas the scale from white to green represent significant overlaps of decreasing FDR adjusted p-values.

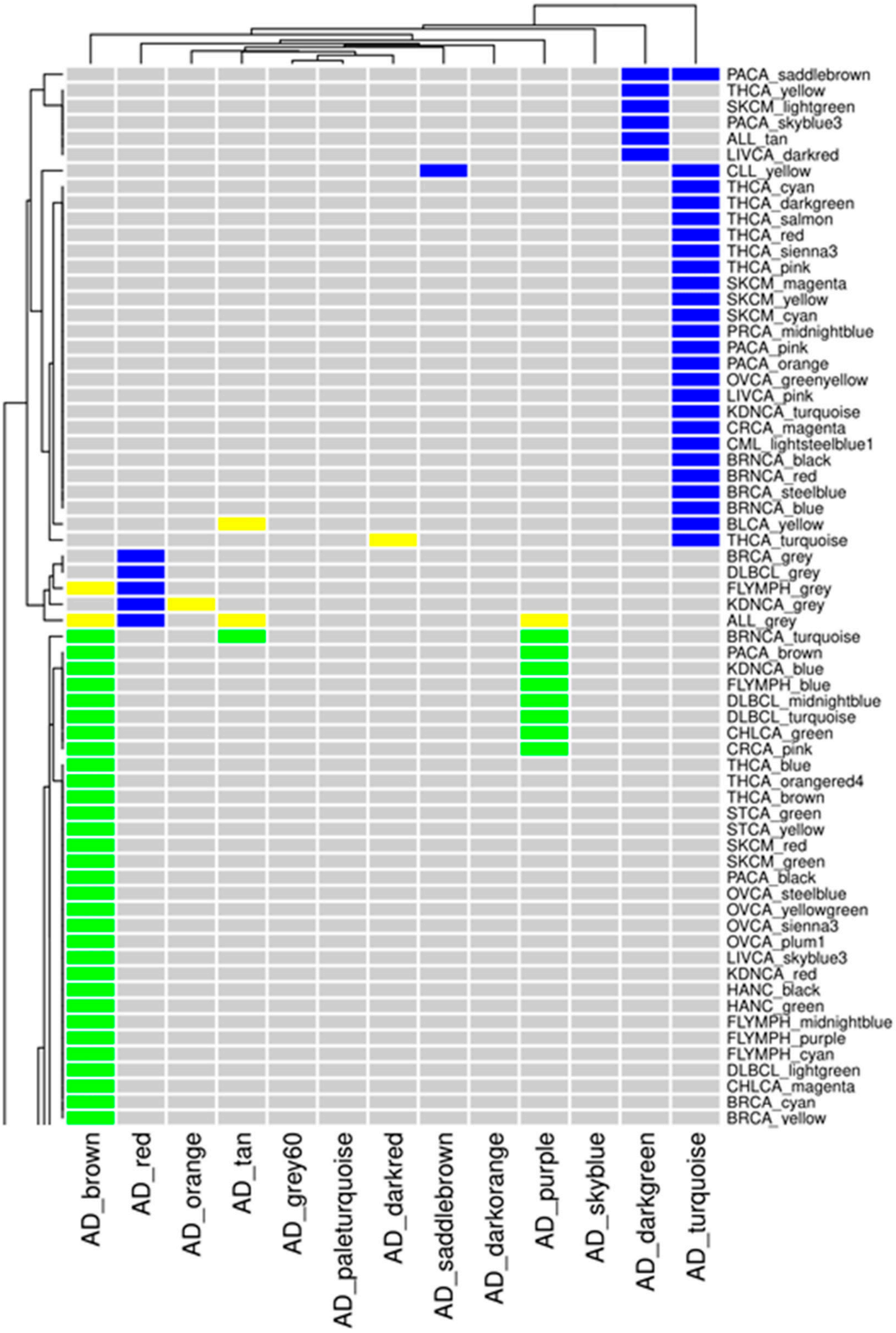

PACA_saddlebrown
THCA_yellow
SKCM_lightgreen
PACA_skyblue3
ALL_tan
LIVCA_darkred
CLL_yellow
THCA_cyan
THCA_darkgreen
THCA_salmon
THCA_red
THCA_sienna3
THCA_pink
SKCM_magenta
SKCM_yellow
SKCM_cyan
PRCA_midnightblue
PACA_pink
PACA_orange
OVCA_greenyellow
LIVCA_pink
KDNCA_turquoise
CRCA_magenta
CML_lightsteelblue1
BRNCA_black
BRNCA_red
BRCA_steelblue
BRNCA_blue
BLCA_yellow
THCA_turquoise
BRCA_grey
DLBCL_grey
FLYMPH_grey
KDNCA_grey
ALL_grey
BRNCA_turquoise
PACA_brown
KDNCA_blue
FLYMPH_blue
DLBCL_midnightblue
DLBCL_turquoise
CHLCA_green
CRCA_pink
THCA_blue
THCA_orangered4
THCA_brown
STCA_green
STCA_yellow
SKCM_red
SKCM_green
PACA_black
OVCA_steelblue
OVCA_yellowgreen
OVCA_sienna3
OVCA_plum1
LIVCA_skyblue3
KDNCA_red
HANC_black
HANC_green
FLYMPH_midnightblue
FLYMPH_purple
FLYMPH_cyan
DLBCL_lightgreen
CHLCA_magenta
BRCA_cyan
BRCA_yellow
AD_brown
AD_red
AD_orange
AD_tan
AD_grey60
AD_paleturquoise
AD_darkred
AD_saddlebrown
AD_darkorange
AD_purple
AD_skyblue
AD_darkgreen
AD_turquoise

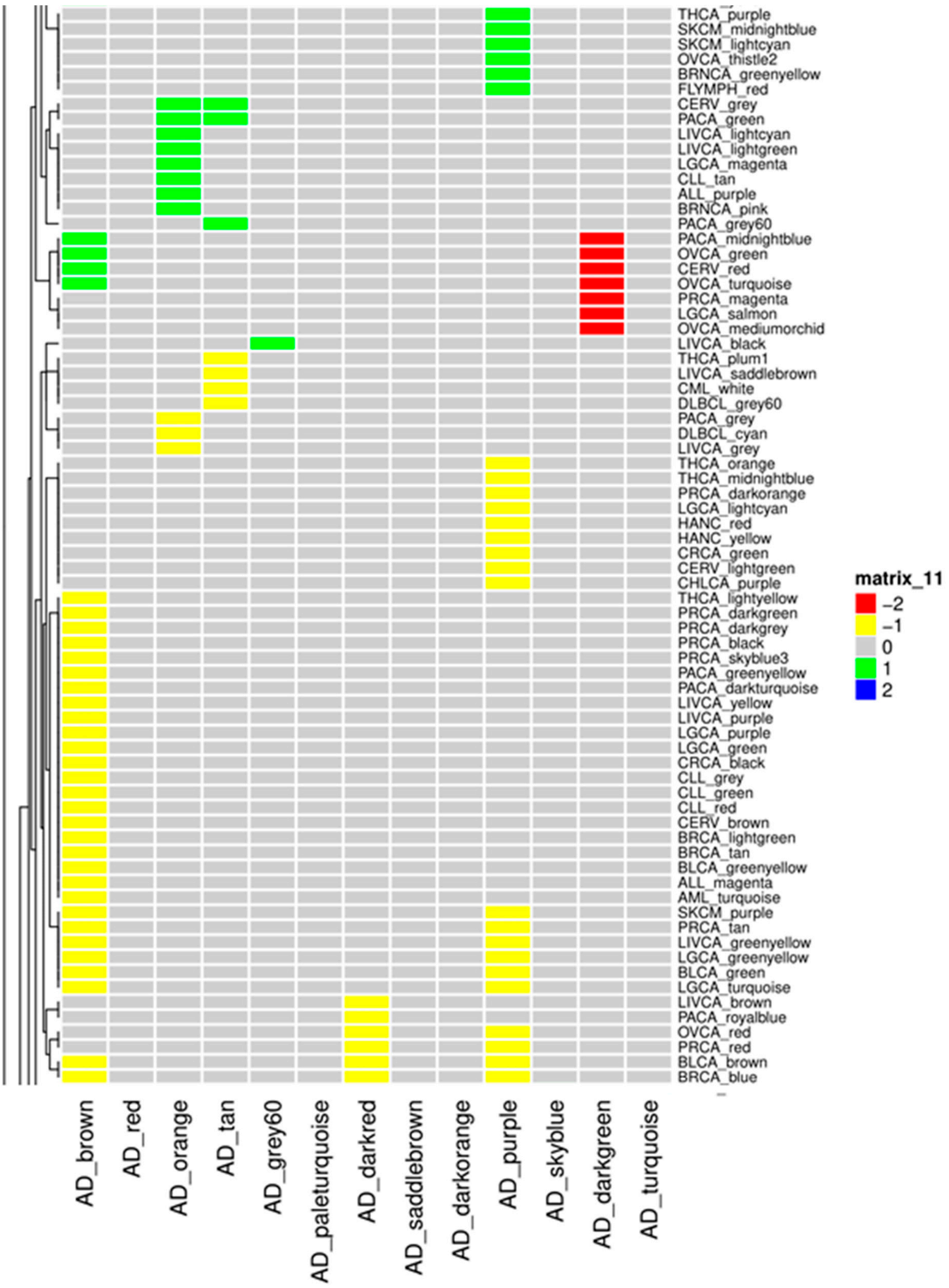
THCA_purple
SKCM_midnightblue
SKCM_lightcyan
OVCA_thistle2
BRNCA_greenyellow
FLYMPH_red
CERV_grey
PACA_green
LIVCA_lightcyan
LIVCA_lightgreen
LGCA_magenta
CLL_tan
ALL_purple
BRNCA_pink
PACA_grey60
PACA_midnightblue
OVCA_green
CERV_red
OVCA_turquoise
PRCA_magenta
LGCA_salmon
OVCA_mediumorchid
LIVCA_black
THCA_plum1
LIVCA_saddlebrown
CML_white
DLBCL_grey60
PACA_grey
DLBCL_cyan
LIVCA_grey
THCA_orange
THCA_midnightblue
PRCA_darkorange
LGCA_lightcyan
HANC_red
HANC_yellow
CRCA_green
CERV_lightgreen
CHLCA_purple
THCA_lightyellow
PRCA_darkgreen
PRCA_darkgrey
PRCA_black
PRCA_skyblue3
PACA_greenyellow
PACA_darkturquoise
LIVCA_yellow
LIVCA_purple
LGCA_purple
LGCA_green
CRCA_black
CLL_grey
CLL_green
CLL_red
CERV_brown
BRCA_lightgreen
BRCA_tan
BLCA_greenyellow
ALL_magenta
AML_turquoise
SKCM_purple
PRCA_tan
LIVCA_greenyellow
LGCA_greenyellow
BLCA_green
LGCA_turquoise
LIVCA_brown
PACA_royalblue
OVCA_red
PRCA_red
BLCA_brown
BRCA_blue
AD_brown
AD_red
AD_orange
AD_tan
AD_grey60
AD_paleturquoise
AD_darkred
AD_saddlebrown
AD_darkorange
AD_purple
AD_skyblue
AD_darkgreen
AD_turquoise
matrix_11
−2
−1
0
1
2

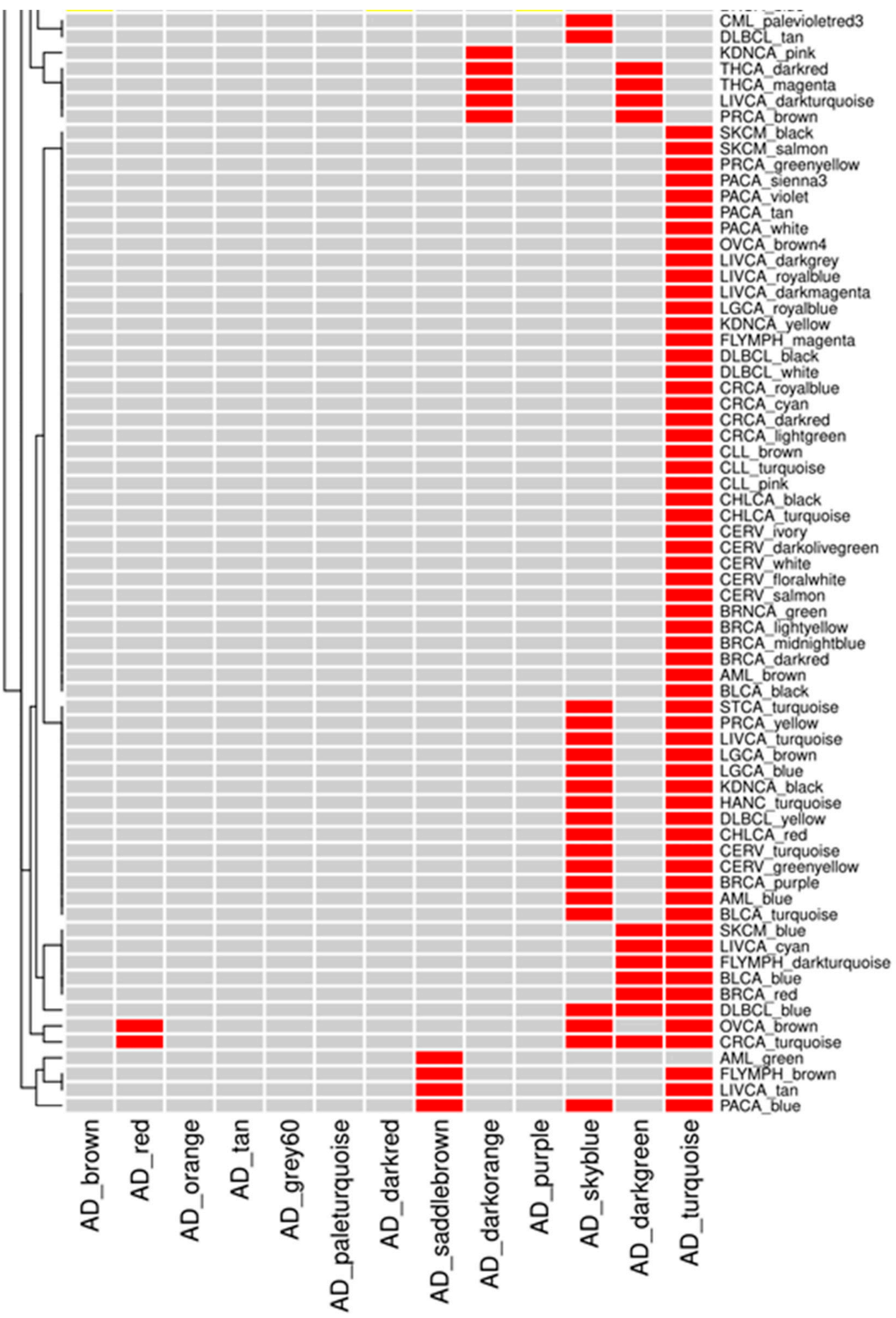

Figure S6. Module overlap analyses between the consensus co-expression modules significantly associated with disease status in AD and cancer analyses. Red cells represent modules presented significant overlaps in gene content which were found to be negatively correlated to disease status in both AD and cancer analyses. Yellow cells show modules presenting positive correlations with AD's disease status and negative correlations with cancer. Green cells show significant module overlaps between modules positively correlated with AD's disease status and positively correlated with cancer disease status. Blue cells show significant overlaps in modules negatively correlated to both AD and cancer disease status. Finally, grey cells show module pairs for which no significant overlap was found.

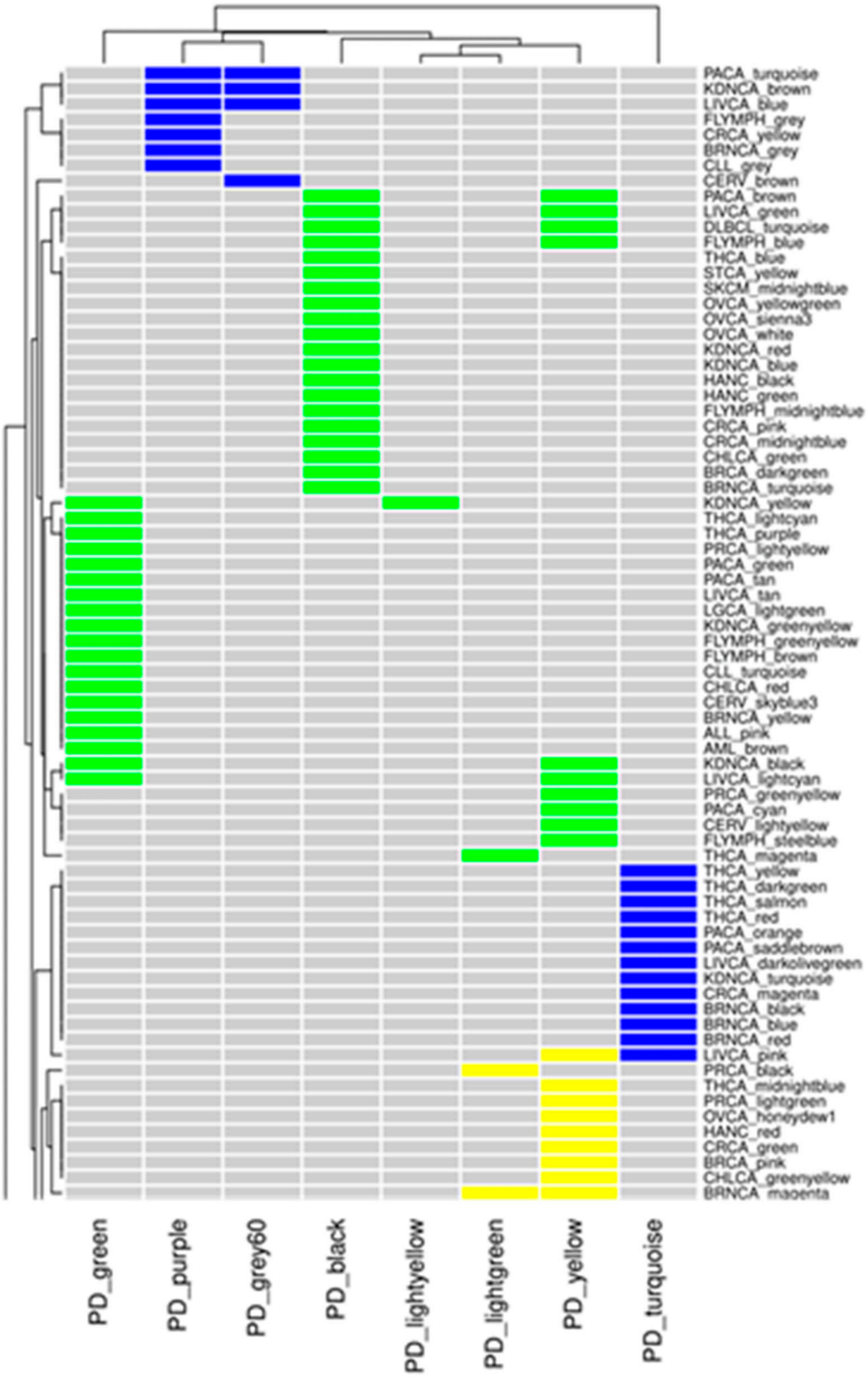
PACA_turquoise
KDNCA_brown
LIVCA_blue
FLYMPH_grey
CRCA_yellow
BRNCA_grey
CLL_grey
CERV_brown
PACA_brown
LIVCA_green
DLBCL_turquoise
FLYMPH_blue
THCA_blue
STCA_yellow
SKCM_midnightblue
OVCA_yellowgreen
OVCA_sienna3
OVCA_white
KDNCA_red
KDNCA_blue
HANC_black
HANC_green
FLYMPH_midnightblue
CRCA_pink
CRCA_midnightblue
CHLCA_green
BRCA_darkgreen
BRNCA_turquoise
KDNCA_yellow
THCA_lightcyan
THCA_purple
PRCA_lightyellow
PACA_green
PACA_tan
LIVCA_tan
LGCA_lightgreen
KDNCA_greenyellow
FLYMPH_greenyellow
FLYMPH_brown
CLL_turquoise
CHLCA_red
CERV_skyblue3
BRNCA_yellow
ALL_pink
AML_brown
KDNCA_black
LIVCA_lightcyan
PRCA_greenyellow
PACA_cyan
CERV_lightyellow
FLYMPH_steelblue
THCA_magenta
THCA_yellow
THCA_darkgreen
THCA_salmon
THCA_red
PACA_orange
PACA_saddlebrown
LIVCA_darkolivegreen
KDNCA_turquoise
CRCA_magenta
BRNCA_black
BRNCA_blue
BRNCA_red
LIVCA_pink
PRCA_black
THCA_midnightblue
PRCA_lightgreen
OVCA_honeydew1
HANC_red
CRCA_green
BRCA_pink
CHLCA_greenyellow
BRNCA_magenta
PD_green
PD_purple
PD_grey60
PD_black
PD_lightyellow
PD_lightgreen
PD_yellow
PD_turquoise

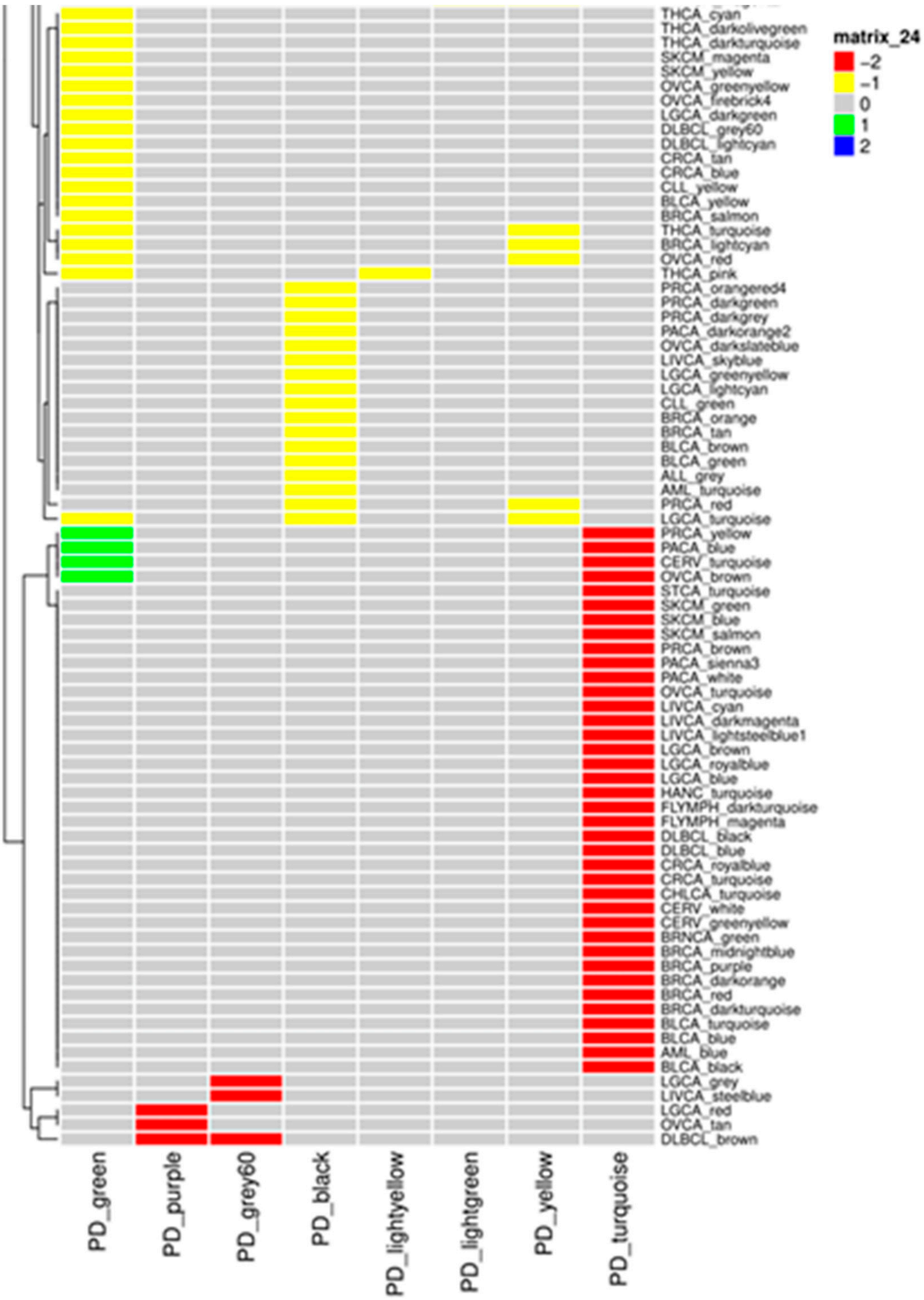

Figure S7. Module overlap analyses between the consensus co-expression modules significantly associated with disease status in AD and cancer analyses. Module overlap analyses between the consensus co-expression modules significantly associated with disease status in PD and cancer analyses. Red cells represent modules presented significant overlaps in gene content which were found to be negatively correlated to disease status in both PD and cancer analyses. Yellow cells show modules presenting positive correlations with PD's disease status and negative correlations with cancer. Green cells show significant module overlaps between modules positively correlated with PD's disease status and positively correlated with cancer disease status. Blue cells show significant overlaps in modules negatively correlated to both PD and cancer disease status. Finally, grey cells show module pairs for which no significant overlap was found.

Reference

1. Dubois, B., et al., *Advancing research diagnostic criteria for Alzheimer's disease: the IWG-2 criteria.* Lancet Neurol, 2014. **13**(6): p. 614-29.
2. Trist, B.G., D.J. Hare, and K.L. Double, *Oxidative stress in the aging substantia nigra and the etiology of Parkinson's disease.* Aging Cell, 2019. **18**(6): p. e13031.
3. Carvalho, B.S. and R.A. Irizarry, *A framework for oligonucleotide microarray preprocessing.* Bioinformatics, 2010. **26**(19): p. 2363-7.
4. Gautier, L., et al., *affy--analysis of Affymetrix GeneChip data at the probe level.* Bioinformatics, 2004. **20**(3): p. 307-15.
5. Ritchie, M.E., et al., *limma powers differential expression analyses for RNA-sequencing and microarray studies.* Nucleic Acids Res, 2015. **43**(7): p. e47.
6. Du, P., W.A. Kibbe, and S.M. Lin, *lumi: a pipeline for processing Illumina microarray.* Bioinformatics, 2008. **24**(13): p. 1547-8.
7. Langfelder, P. and S. Horvath, *WGCNA: an R package for weighted correlation network analysis.* BMC Bioinformatics, 2008. **9**: p. 559.
8. Colaprico, A., et al., *TCGAbiolinks: an R/Bioconductor package for integrative analysis of TCGA data.* Nucleic Acids Res, 2016. **44**(8): p. e71.
9. Kauffmann, A., R. Gentleman, and W. Huber, *arrayQualityMetrics--a bioconductor package for quality assessment of microarray data.* Bioinformatics, 2009. **25**(3): p. 415-6.
10. Wang, X., et al., *An R package suite for microarray meta-analysis in quality control, differentially expressed gene analysis and pathway enrichment detection.* Bioinformatics, 2012. **28**(19): p. 2534-6.
11. Sweeney, T.E., et al., *Methods to increase reproducibility in differential gene expression via meta-analysis.* Nucleic Acids Res, 2017. **45**(1): p. e1.
12. Choi, J.K., et al., *Combining multiple microarray studies and modeling interstudy variation.* Bioinformatics, 2003. **19 Suppl 1**: p. i84-90.
13. Ibanez, K., et al., *Molecular evidence for the inverse comorbidity between central nervous system disorders and cancers detected by transcriptomic meta-analyses.* PLoS Genet, 2014. **10**(2): p. e1004173.
14. Forés-Martos, J., et al., *Transcriptomic metaanalyses of autistic brains reveals shared gene expression and biological pathway abnormalities with cancer.* Mol Autism, 2019. **10**: p. 17.
15. Sanchez-Valle, J., et al., *A molecular hypothesis to explain direct and inverse co-morbidities between Alzheimer's Disease, Glioblastoma and Lung cancer.* Sci Rep, 2017. **7**(1): p. 4474.
16. Love, M.I., W. Huber, and S. Anders, *Moderated estimation of fold change and dispersion for RNA-seq data with DESeq2.* Genome Biol, 2014. **15**(12): p. 550.
17. Langfelder, P., P.S. Mischel, and S. Horvath, *When is hub gene selection better than standard meta-analysis?* PLoS One, 2013. **8**(4): p. e61505.
18. Langfelder, P. and S. Horvath, *Eigengene networks for studying the relationships between co-expression modules.* BMC Syst Biol, 2007. **1**: p. 54.
19. Yu, G., et al., *clusterProfiler: an R package for comparing biological themes among gene clusters.* OMICS, 2012. **16**(5): p. 284-7.
20. Langfelder, P., *anRichment: Collections and annotation data for use with anRichmentMethods*. 2020.
21. Sergushichev, A., *An algorithm for fast preranked gene set enrichment analysis using cumulative statistic calculation.* 2016.
22. *MSigDB*. Available from: https://www.gsea-msigdb.org/gsea/msigd.
23. *The Human Reference Interactome (HuRI)*. Available from: http://www.interactome-atlas.org/.

24. Luck, K., et al., *A reference map of the human binary protein interactome.* Nature, 2020. **580**(7803): p. 402-408.
25. *Genotype-Tissue Expression (GTEx)*. Available from: https://www.gtexportal.org/home/datasets.
26. *The comprehensive resource of mammalian protein complexes (CORUM)*. Available from: http://mips.helmholtz-muenchen.de/corum/.
27. Matys, V., et al., *TRANSFAC and its module TRANSCompel: transcriptional gene regulation in eukaryotes.* Nucleic Acids Res, 2006. **34**(Database issue): p. D108-10.
28. Matys, V., et al., *TRANSFAC: transcriptional regulation, from patterns to profiles.* Nucleic Acids Res, 2003. **31**(1): p. 374-8.
29. *Harmonizome*. Available from: http://amp.pharm.mssm.edu/Harmonizome/dataset/TRANSFAC+Curated+Transcription+Factor+Targets.
30. *PhosphoSitePlus* Available from: https://www.phosphosite.org/homeAction.action.
31. *Kyoto Encyclopedia of Genes and Genomes (KEGG)*. Available from: https://www.genome.jp/kegg/.
32. Zhang, J.D. and S. Wiemann, *KEGGgraph: a graph approach to KEGG PATHWAY in R and bioconductor.* Bioinformatics, 2009. **25**(11): p. 1470-1.
33. *STRING*. Available from: https://string-db.org/.
34. *BioGriD*. Available from: https://thebiogrid.org/.
35. *DisGeNet*. Available from: https://www.disgenet.org/.
36. *The Phenotype Genotype Integrator (PheGenI)*. Available from: https://www.ncbi.nlm.nih.gov/gap/phegeni.
37. *eDGAR*. Available from: http://edgar.biocomp.unibo.it/gene_disease_db/.
38. Menche, J., et al., *Disease networks. Uncovering disease-disease relationships through the incomplete interactome.* Science, 2015. **347**(6224): p. 1257601.
39. Bulik-Sullivan, B., et al., *An atlas of genetic correlations across human diseases and traits.* Nat Genet, 2015. **47**(11): p. 1236-41.
40. *GWAS catalog*. Available from: https://www.ebi.ac.uk/gwas/downloads/summary-statistics.
41. *UK Biobank*. Available from: http://www.nealelab.is/uk-biobank.
42. *MEDI-an*. Available from: https://www.vumc.org/cpm/cpm-blog/medi-ensemble-medication-indication-resource-0.
43. *RxNev RESTful*. Available from: https://rxnav.nlm.nih.gov/RxNormAPIREST.html.
44. Wickham, H., *httr Tools for Working with URLs and HTTP.* 2019.
45. Couture-Beil, A., *rjson: JSON for R.* 2018.
46. *UniChem RESTful*. Available from: https://www.ebi.ac.uk/unichem/rest.
